# Supplementary material for: The ER-SURF pathway uses ER-mitochondria contact sites for protein targeting to mitochondria
Source: EMBO Rep. 2024 Apr 2;25(4):24. doi: 10.1038/s44319-024-00113-w (PMC11014988; doi:10.1038/s44319-024-00113-w)
Supplement: Supplementary file 1 — Appendix [file 44319_2024_113_MOESM1_ESM.pdf]

Table of Content

Appendix Figure S1	Venn Diagram of ER-SURF substrates	Page 2
Appendix Figure S2	The ERMES and the Tom70-Djp1/Lam6 tether cooperate in the ER-to-mitochondria transfer of precursor proteins	Page 3

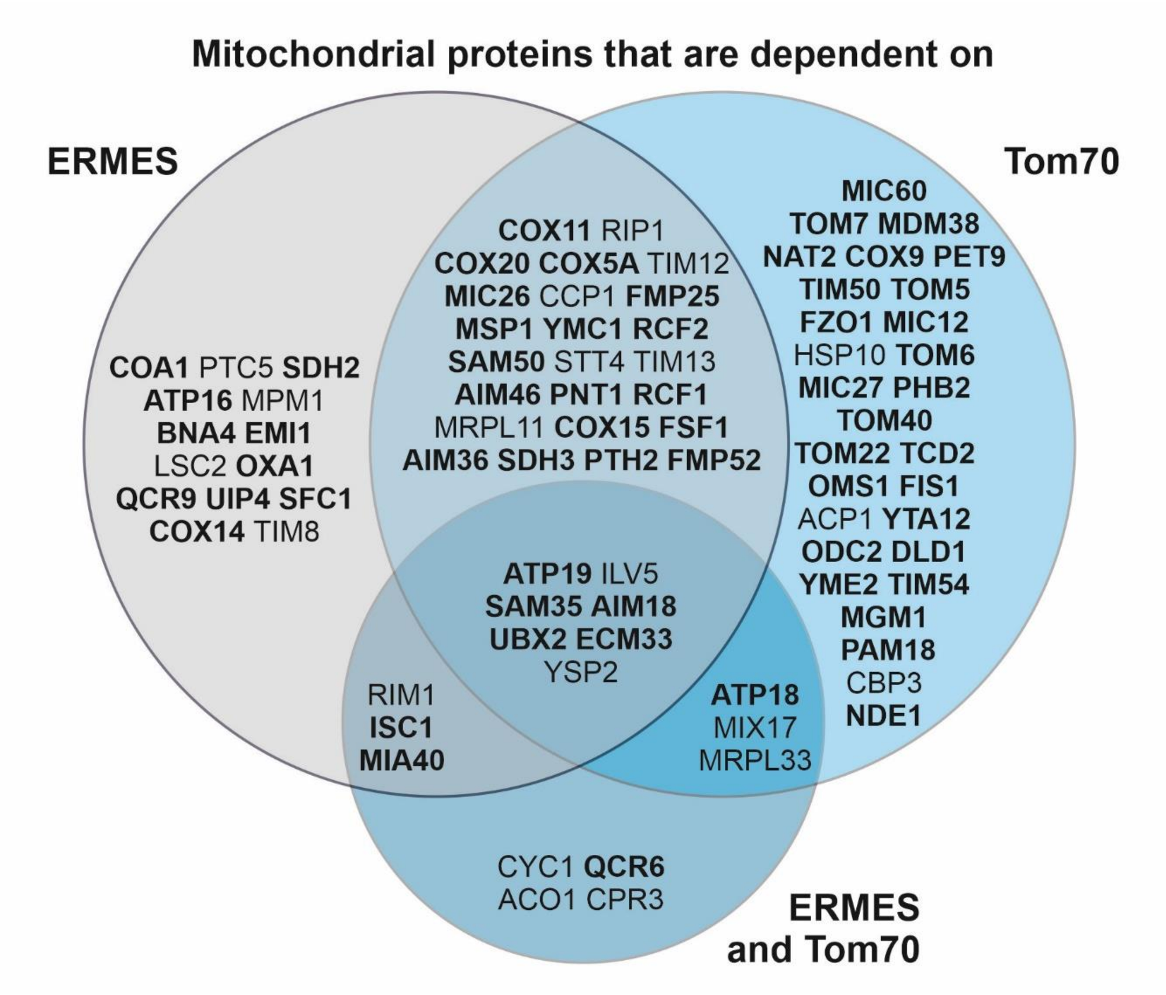

Appendix Figure S1. Venn Diagram of ER-SURF substrates (as defined in Fig.5 E) according to the specific dependance on either ERMES, Tom70 or both. Membrane proteins are highlighted in bold.

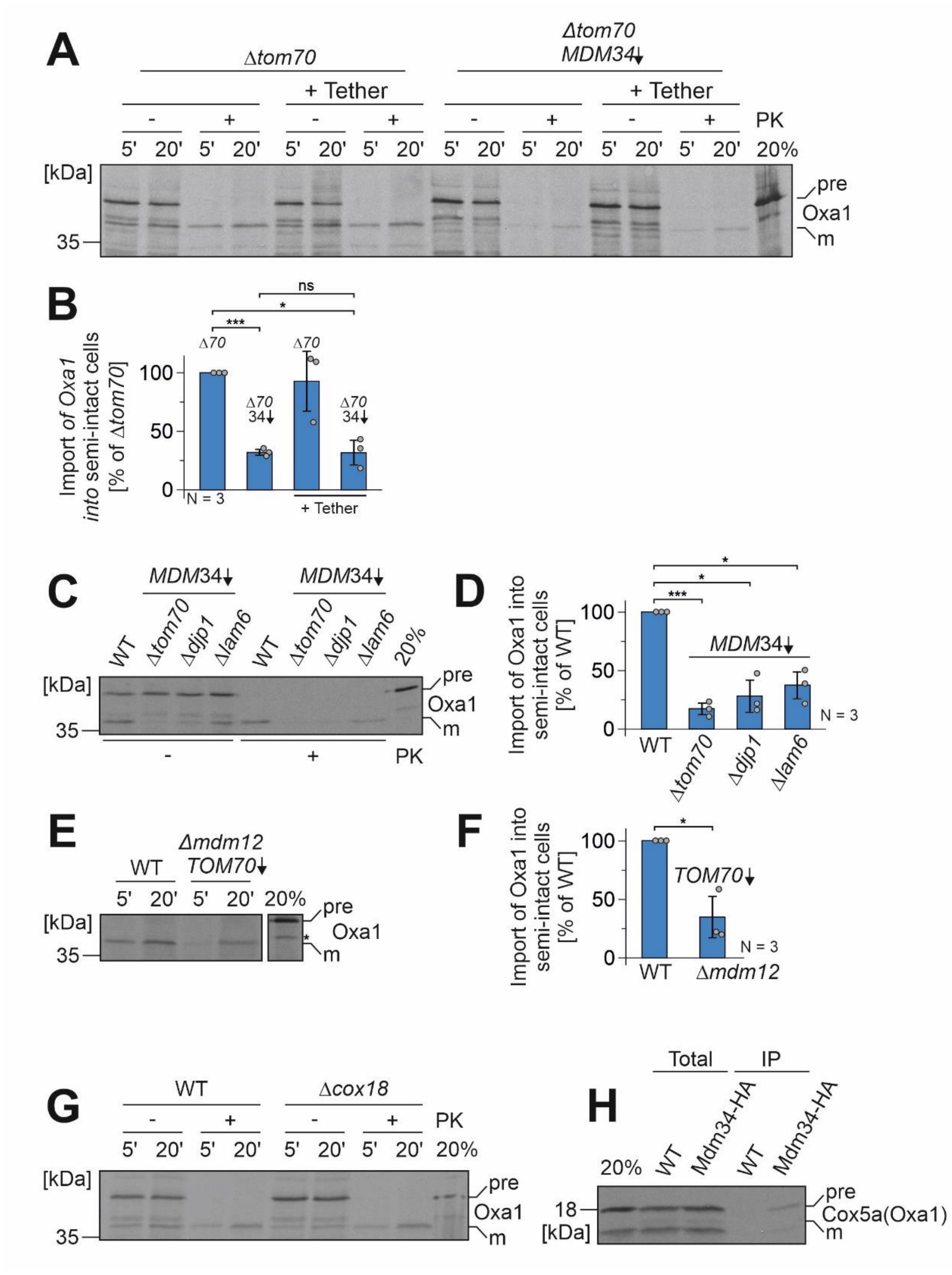

Appendix Figure S2. The ERMES and the Tom70-Djp1/Lam6 tether cooperate in the ER-to-mitochondria transfer of precursor proteins. A-G. Radiolabeled Oxa1 precursor was synthesized in reticulocyte lysate and incubated with semi-intact cells of the indicated strains. After 5 and 20 min (in C only after 20 min), aliquots were taken before samples were treated with proteinase K (PK). The experiments were repeated three times from independent semi-

intact cell preparations (biological replicates) and the signals of the imported proteins were quantified. Shown are mean values and standard deviations. Statistical difference was calculated with a student's t-test. Statistical significance was assigned as follows: p-value < 0.05 = *, p-value < 0.01 = **, p-value < 0.005 = ***. **H.** Radiolabeled Cox5a(Oxa1) precursor was incubated with semi-intact cells of wild type and Mdm34-HA expressing cells. After 5 min, cells were lysed. The extract was incubated with HA-specific antibodies coupled to protein A Sepharose beads. Beads were washed before bound proteins were eluted with sample buffer and subjected to SDS PAGE. The total samples correspond to 25% of what is shown in the IP (immune precipitation) samples.
