# Supplementary figures and images for: The ER-SURF pathway uses ER-mitochondria contact sites for protein targeting to mitochondria
Source: EMBO Rep. 2024 Apr 2;25(4):24. doi: 10.1038/s44319-024-00113-w (PMC11014988; doi:10.1038/s44319-024-00113-w)

Figure 1B

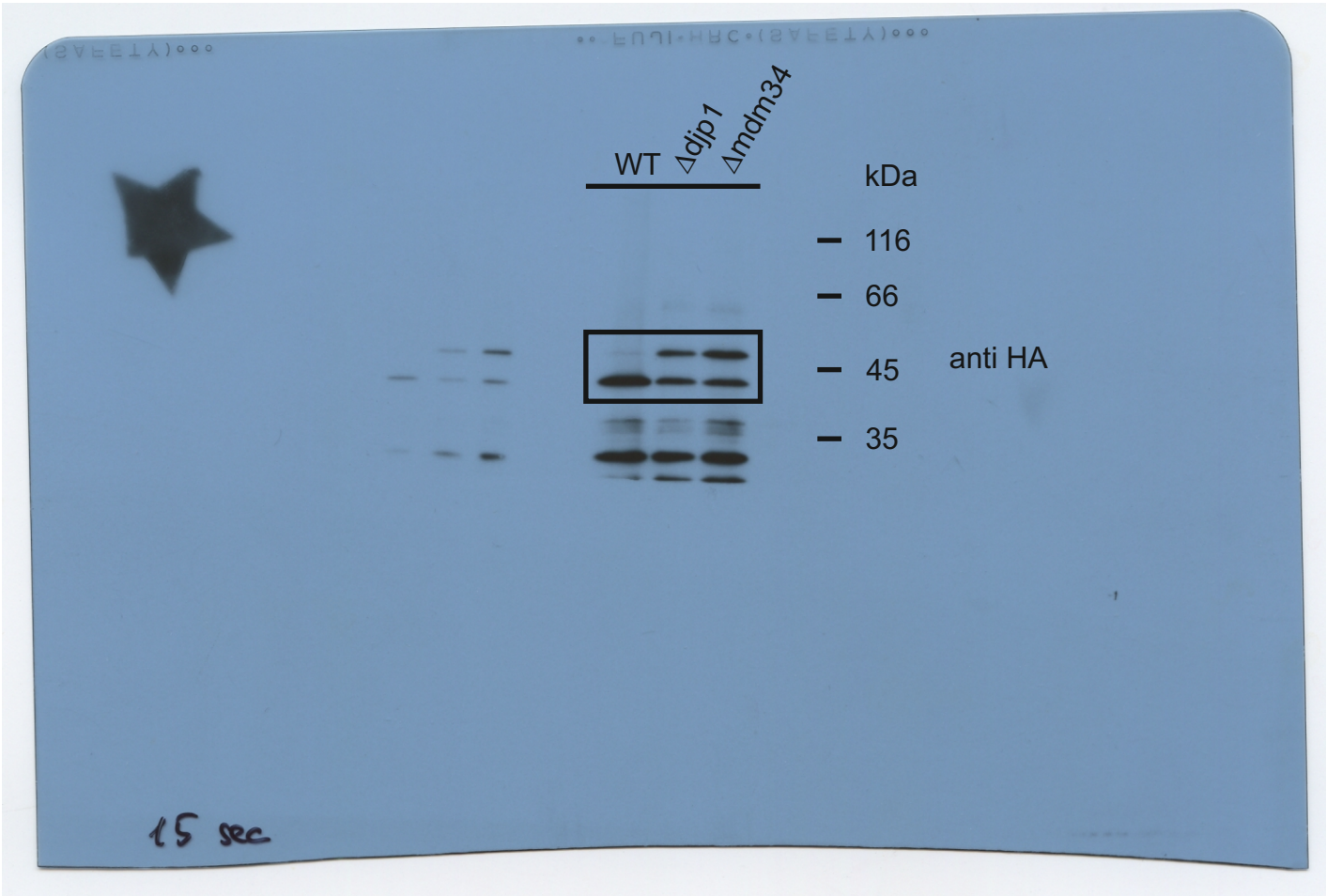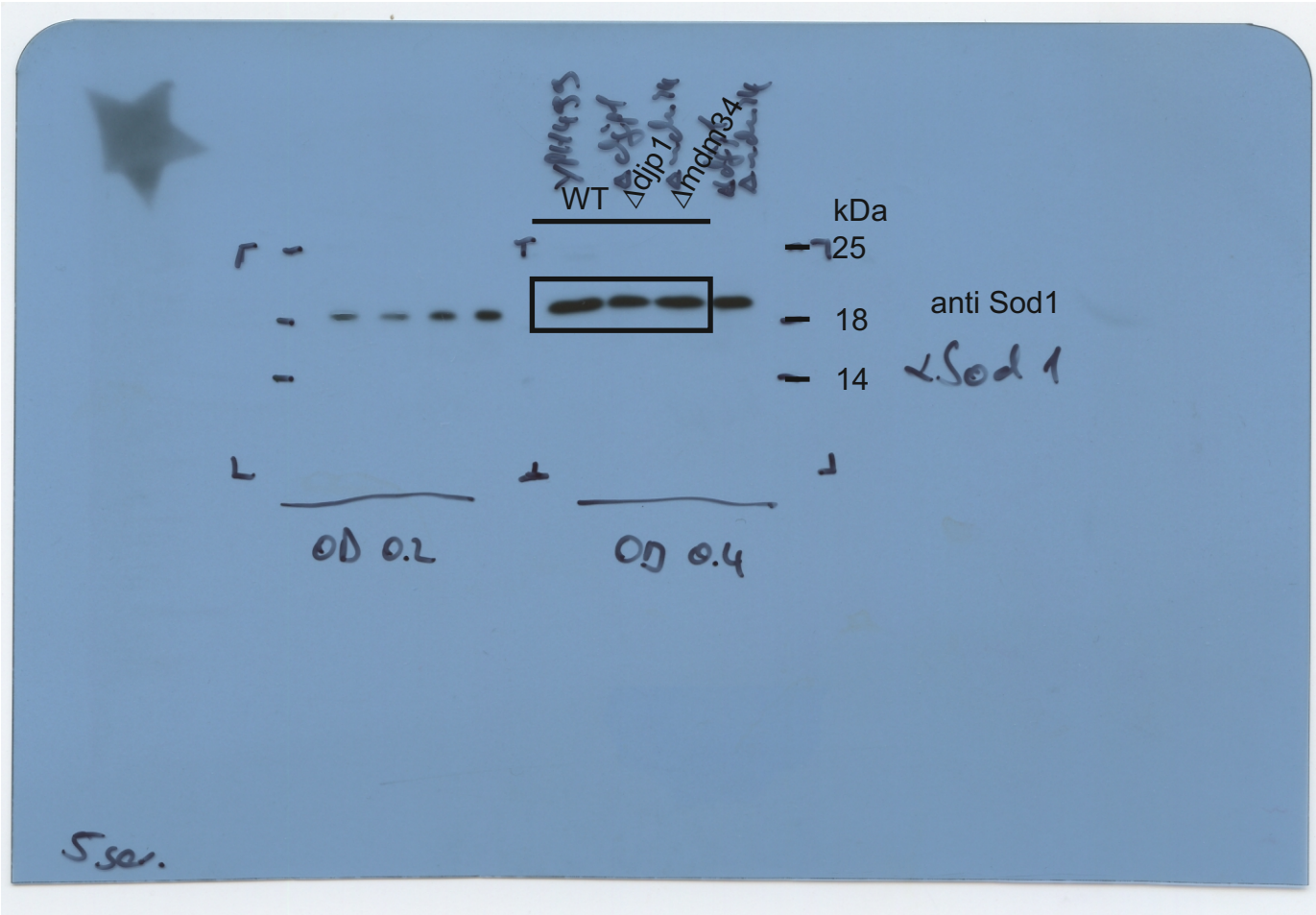

Supplement: Supplementary file 9 — Source data Fig. 1 [file 44319_2024_113_MOESM9_ESM.zip › Koch_SourceData_Fig1/1B/1B.pdf]

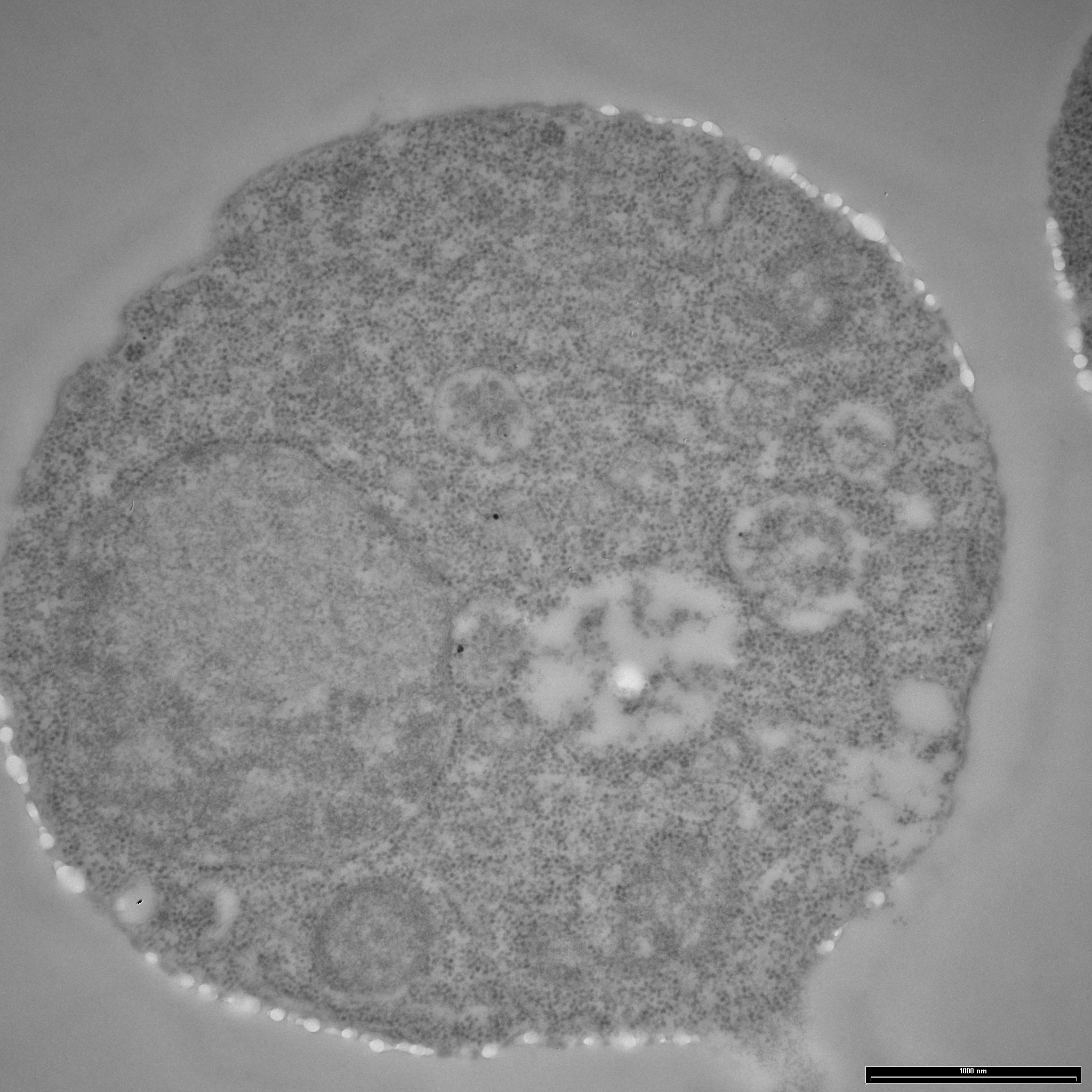

Supplement: Supplementary file 9 — Source data Fig. 1 [file 44319_2024_113_MOESM9_ESM.zip › Koch_SourceData_Fig1/1D/1D_left.tif]

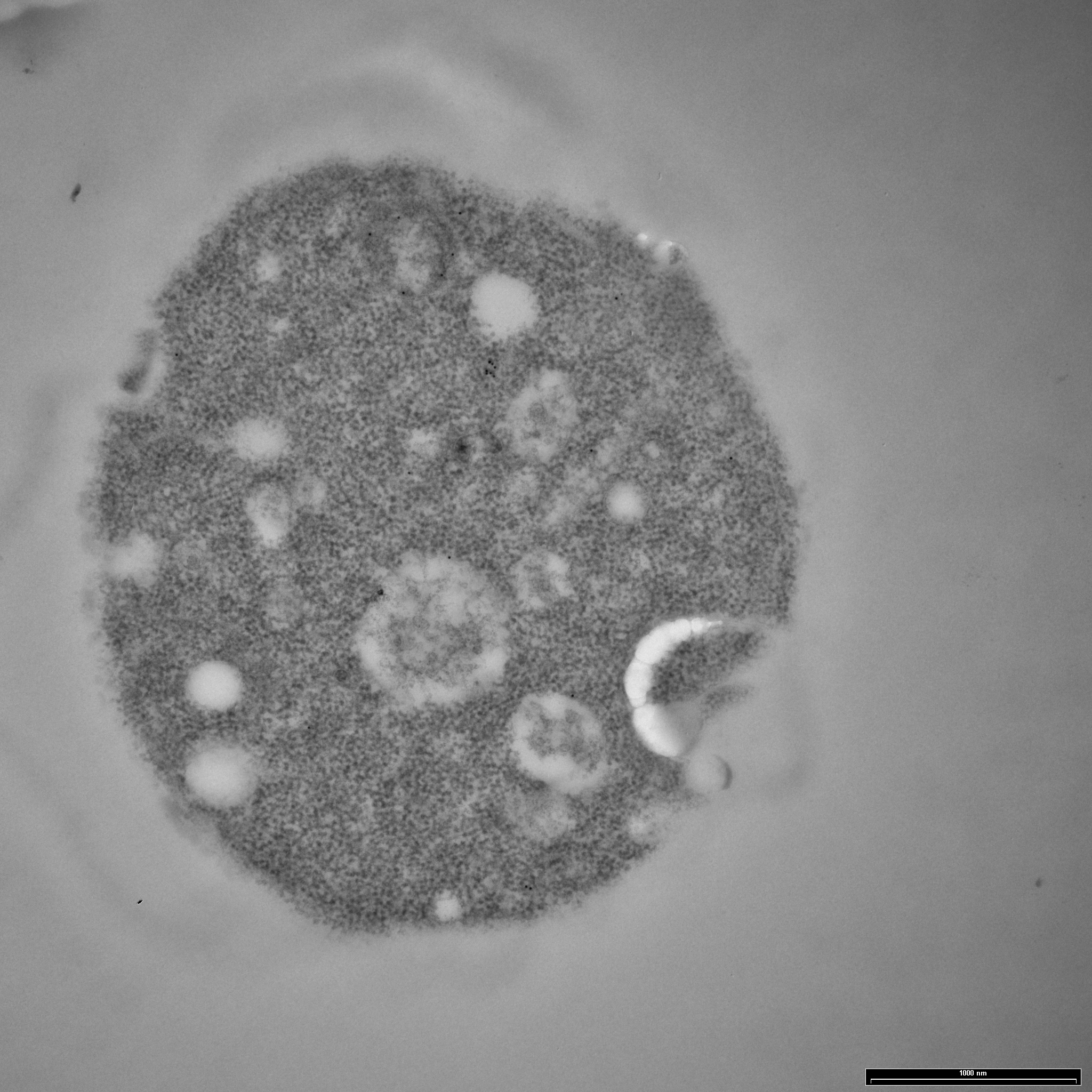

Supplement: Supplementary file 9 — Source data Fig. 1 [file 44319_2024_113_MOESM9_ESM.zip › Koch_SourceData_Fig1/1D/1D_middle.tif]

Figure 1F

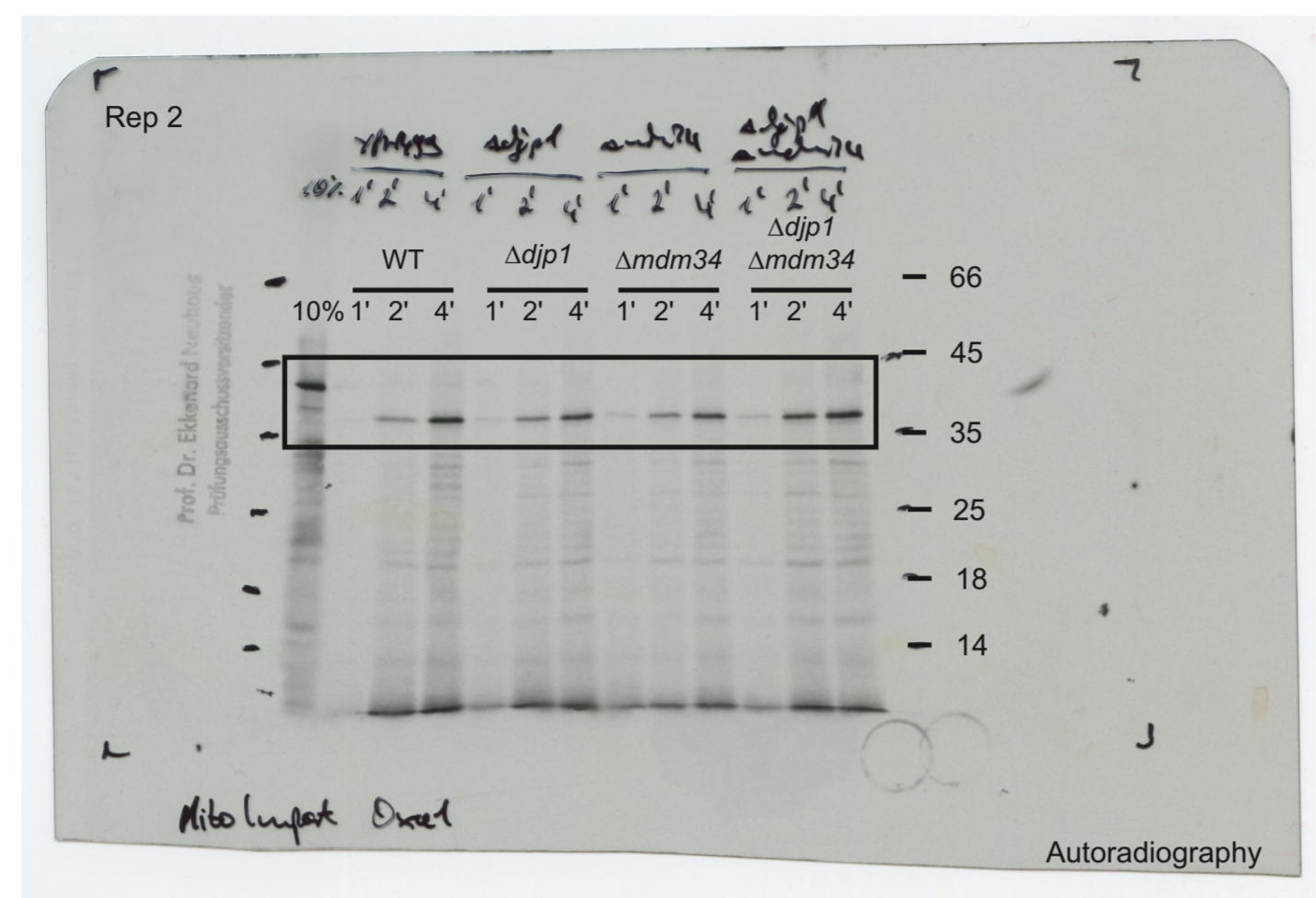

For quantification:

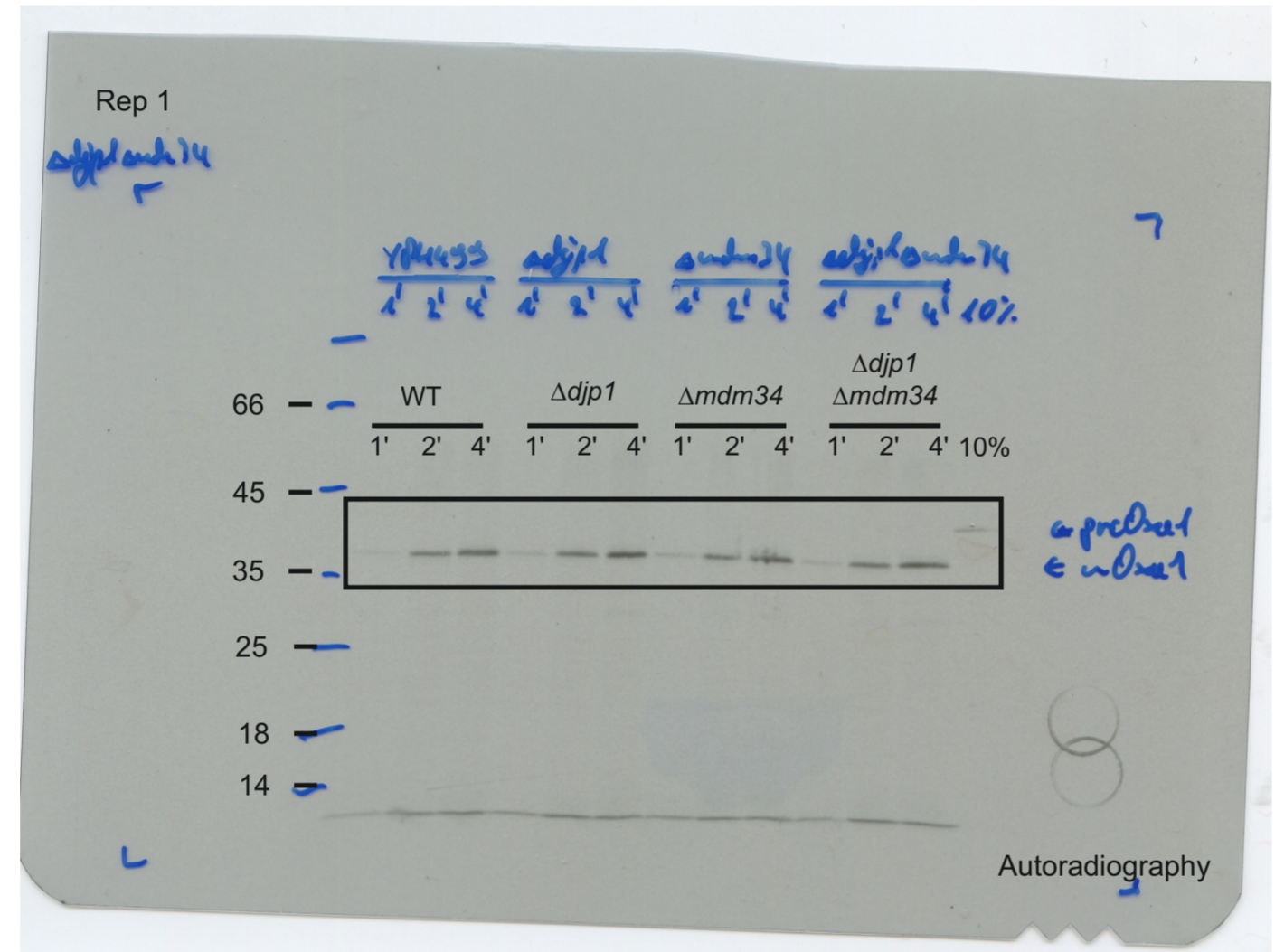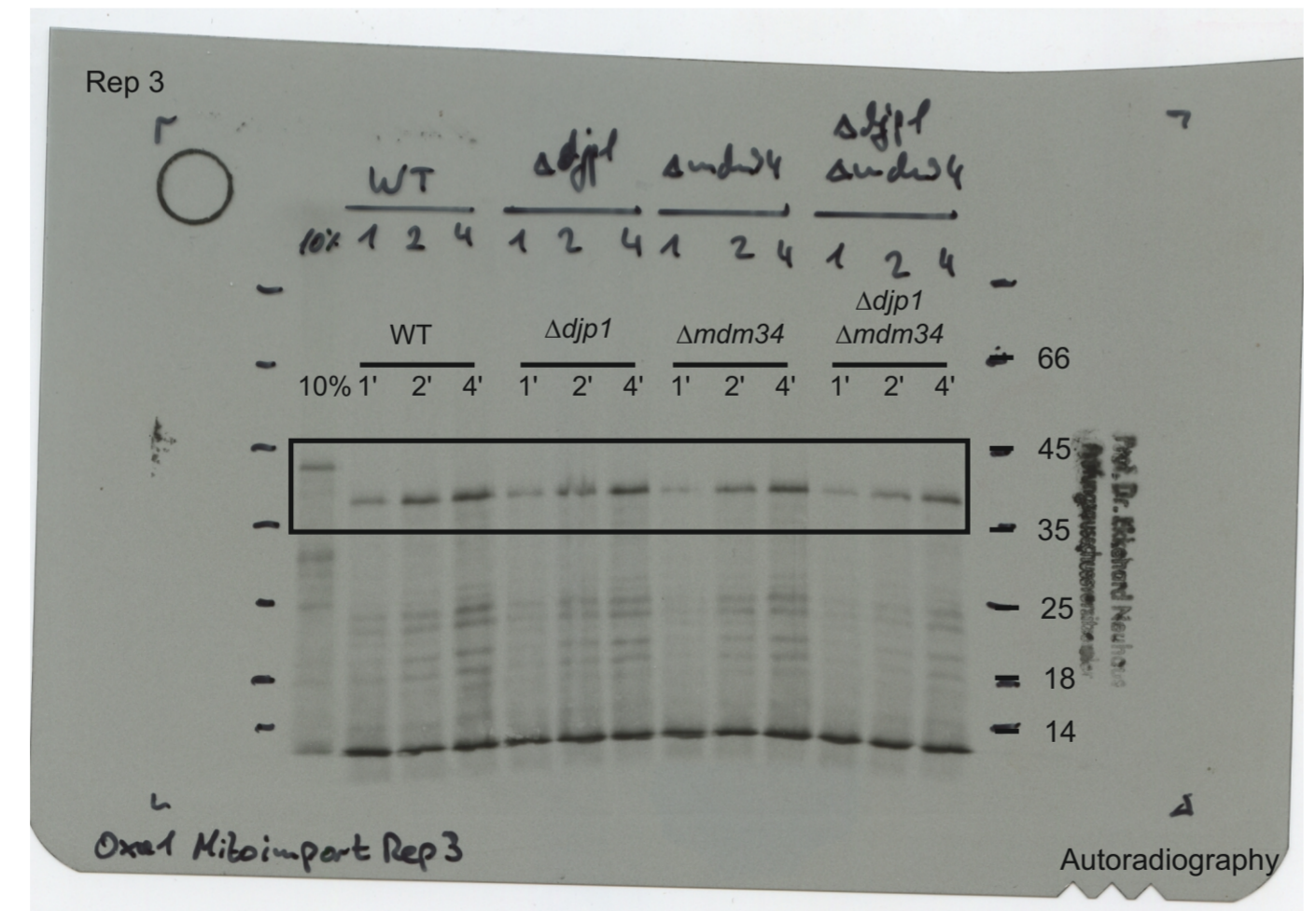

Supplement: Supplementary file 9 — Source data Fig. 1 [file 44319_2024_113_MOESM9_ESM.zip › Koch_SourceData_Fig1/1E-G/1F.pdf]

Figure 1E

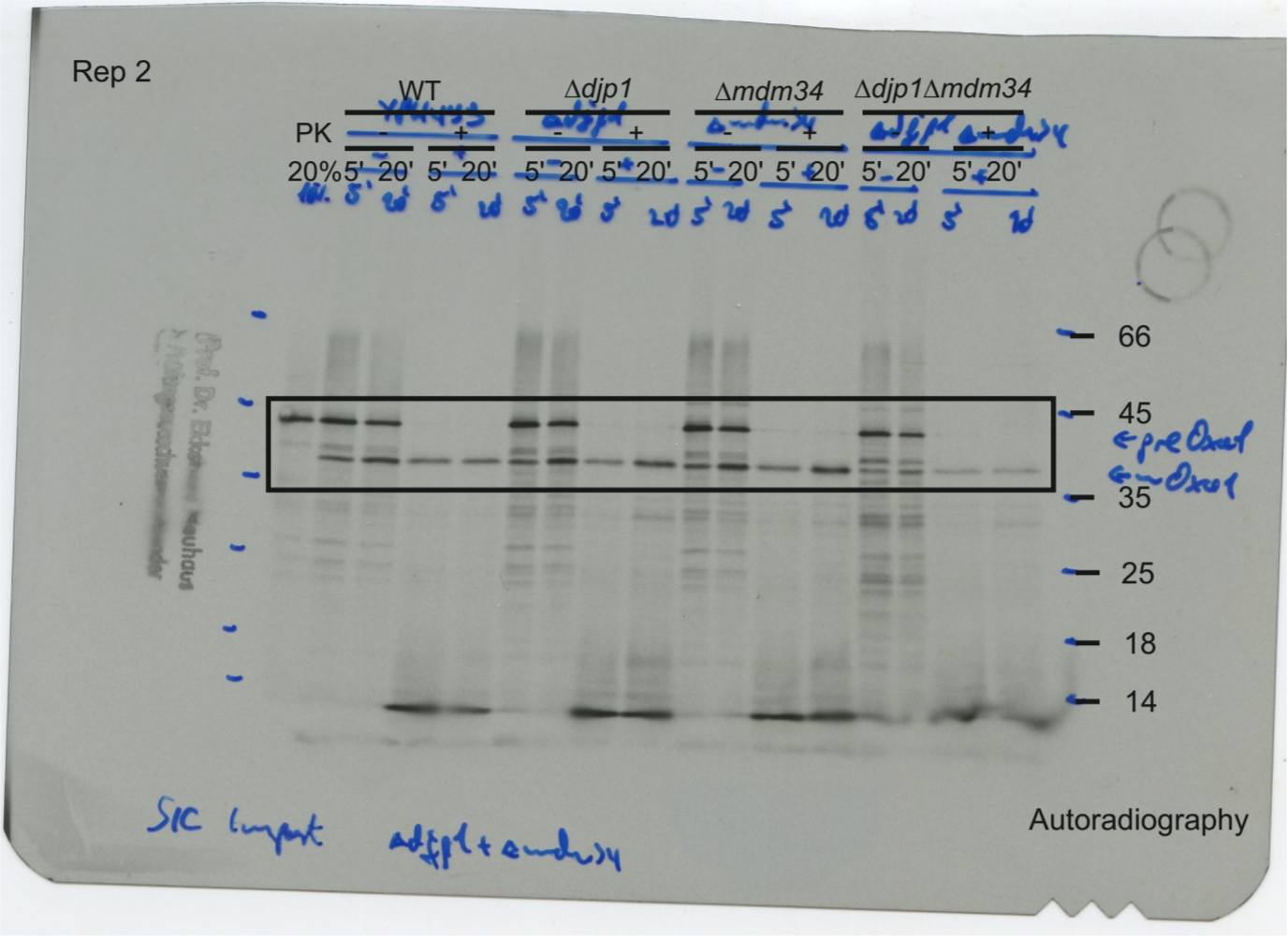

For quantification:

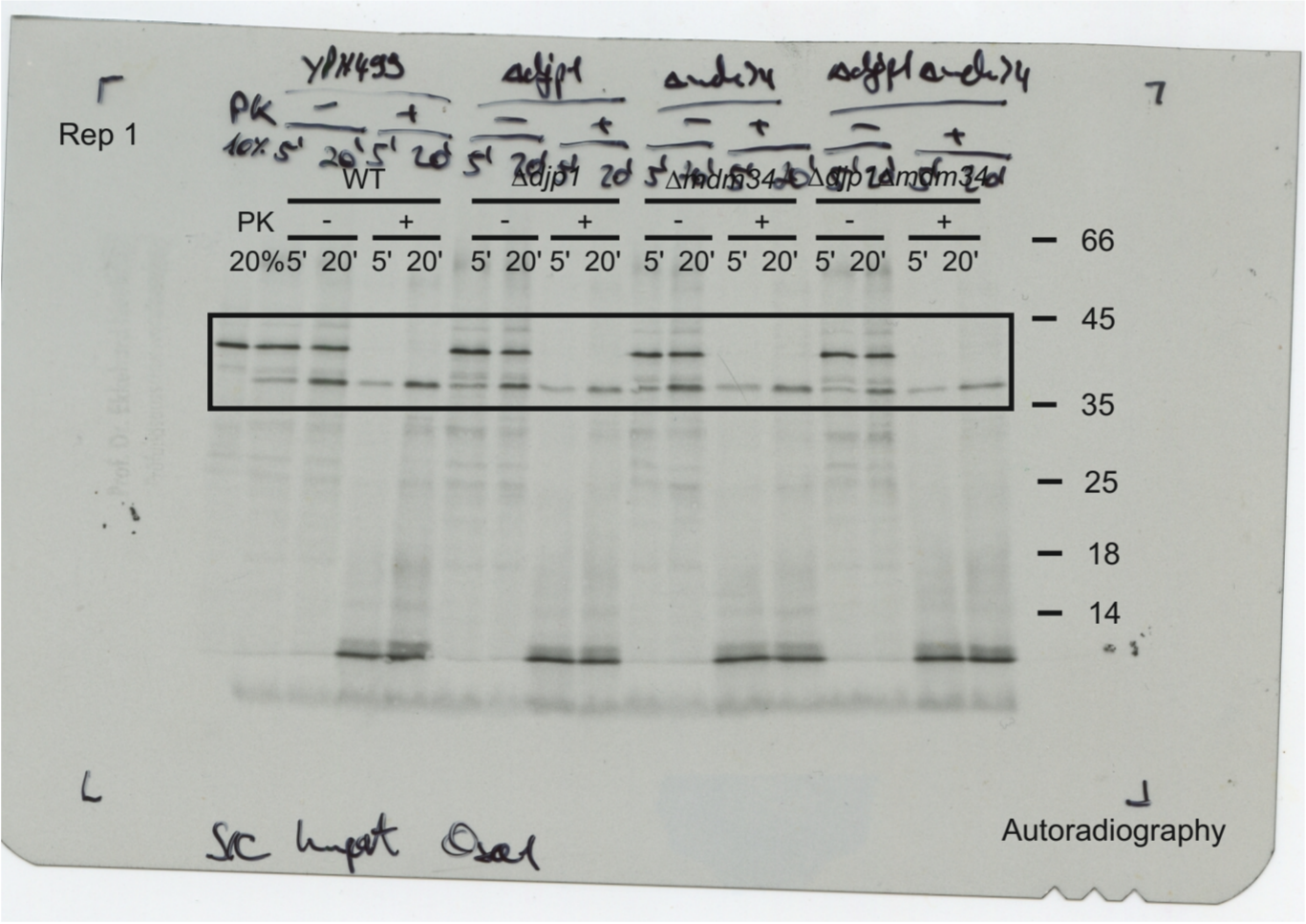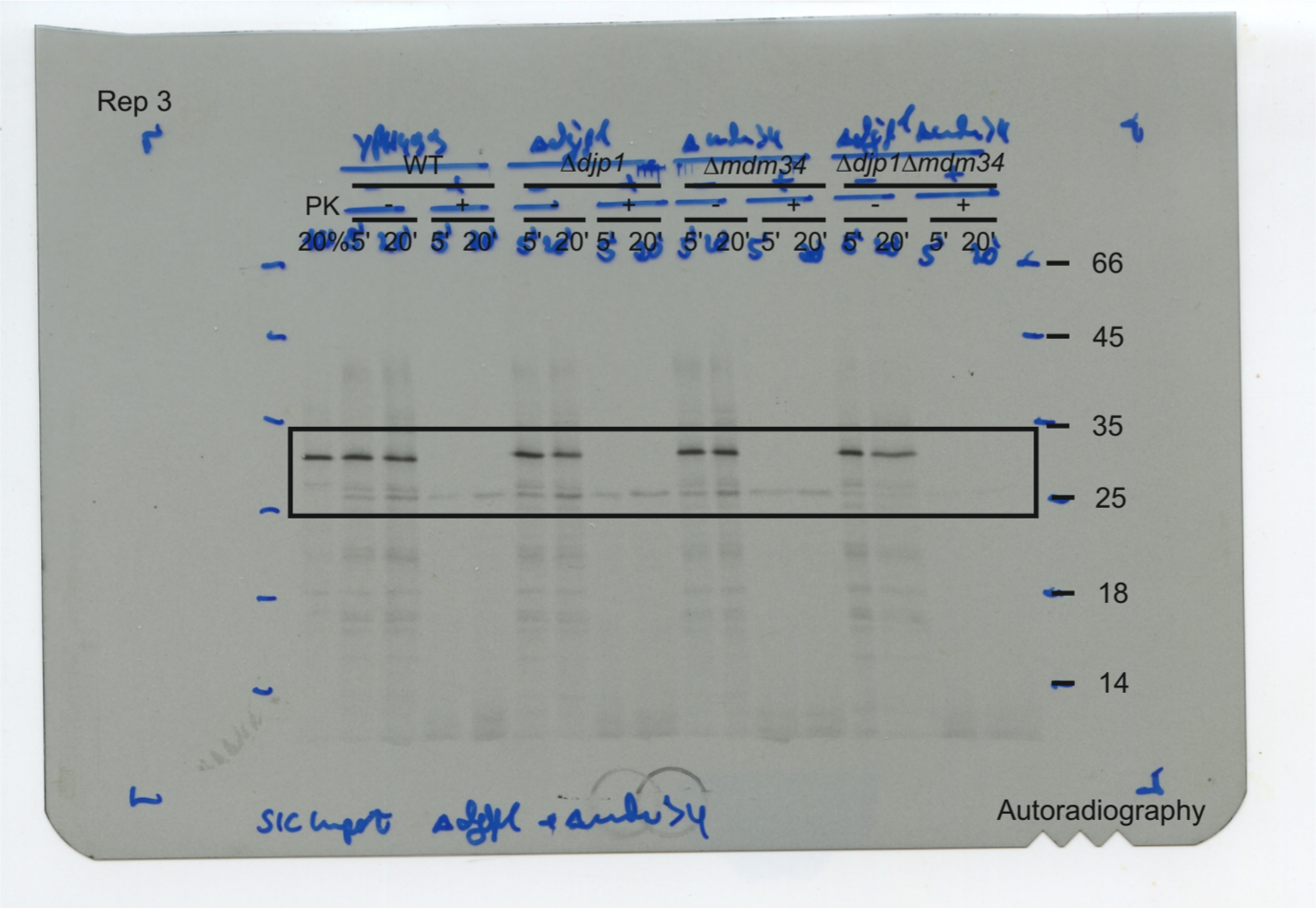

Supplement: Supplementary file 9 — Source data Fig. 1 [file 44319_2024_113_MOESM9_ESM.zip › Koch_SourceData_Fig1/1E-G/1Ê.pdf]

Figure 1I

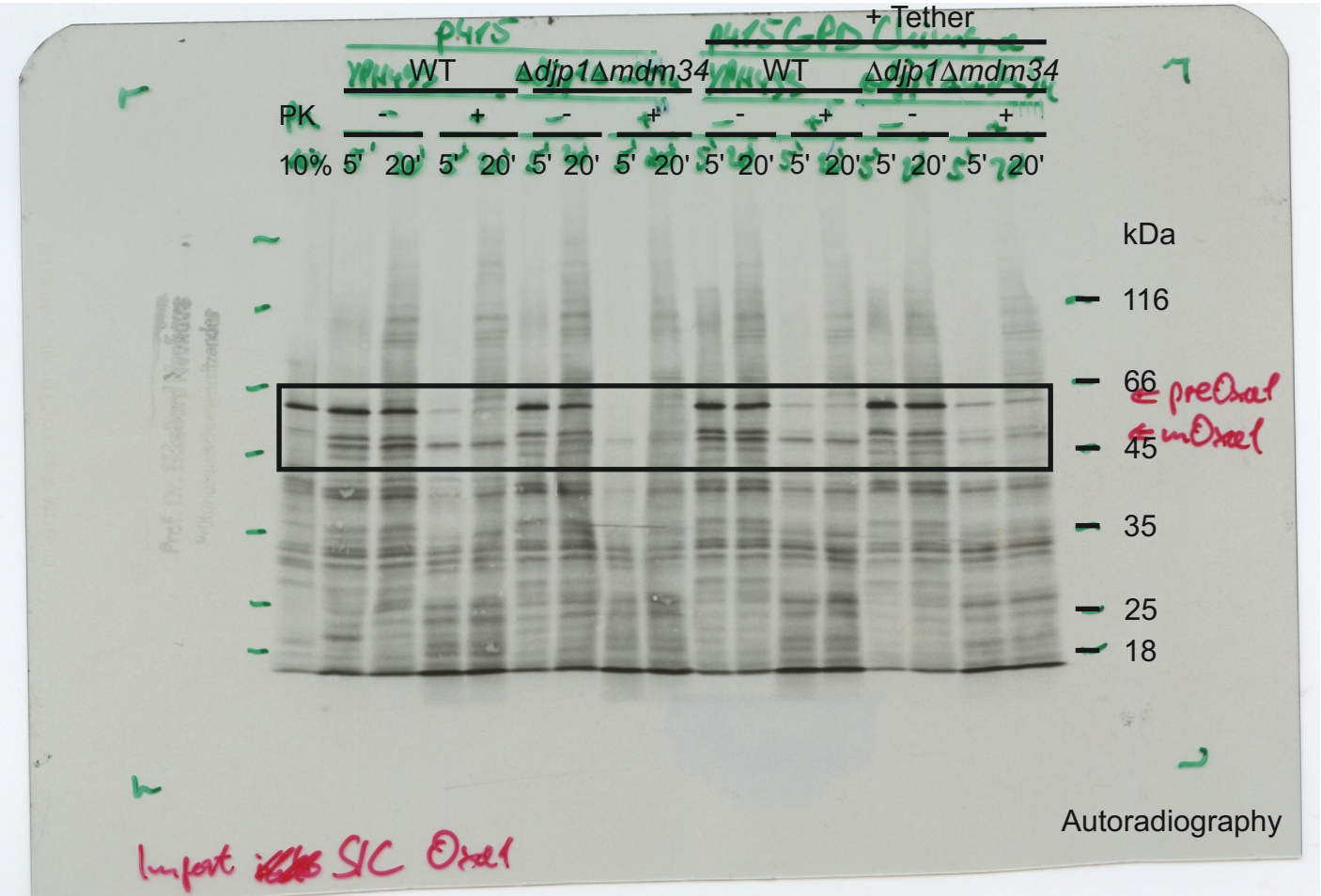

Supplement: Supplementary file 9 — Source data Fig. 1 [file 44319_2024_113_MOESM9_ESM.zip › Koch_SourceData_Fig1/1I/1I.pdf]

Figure 2C

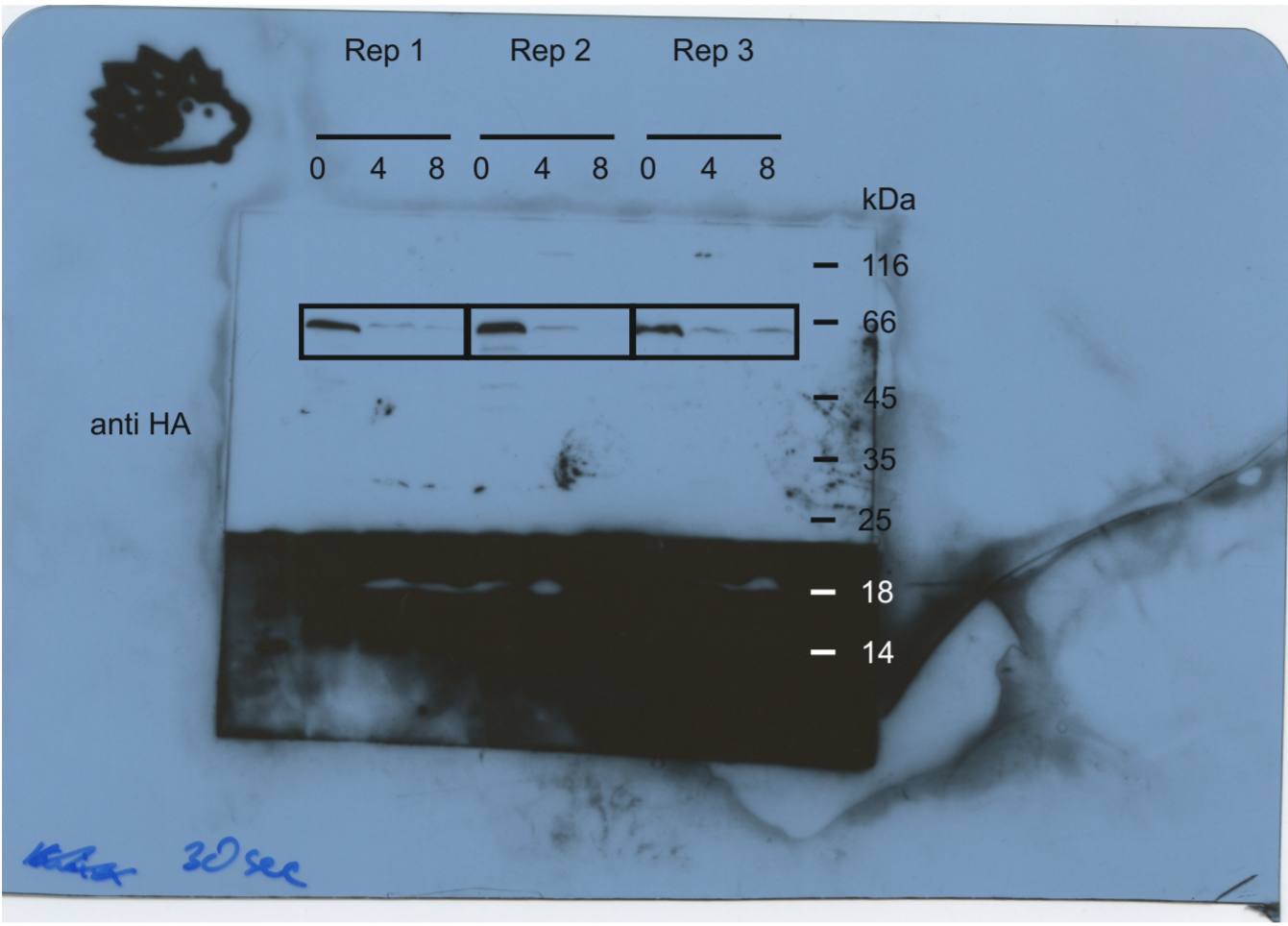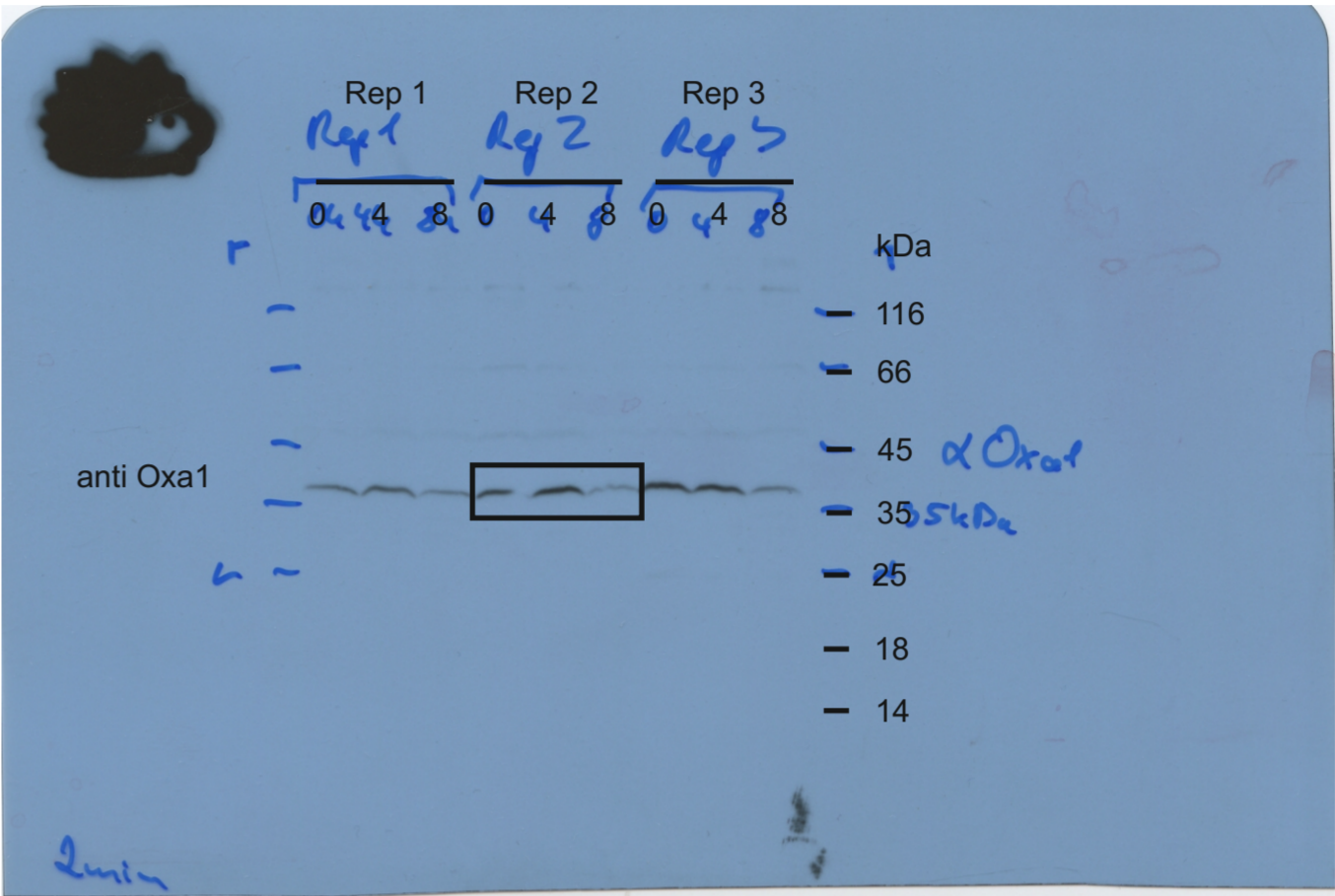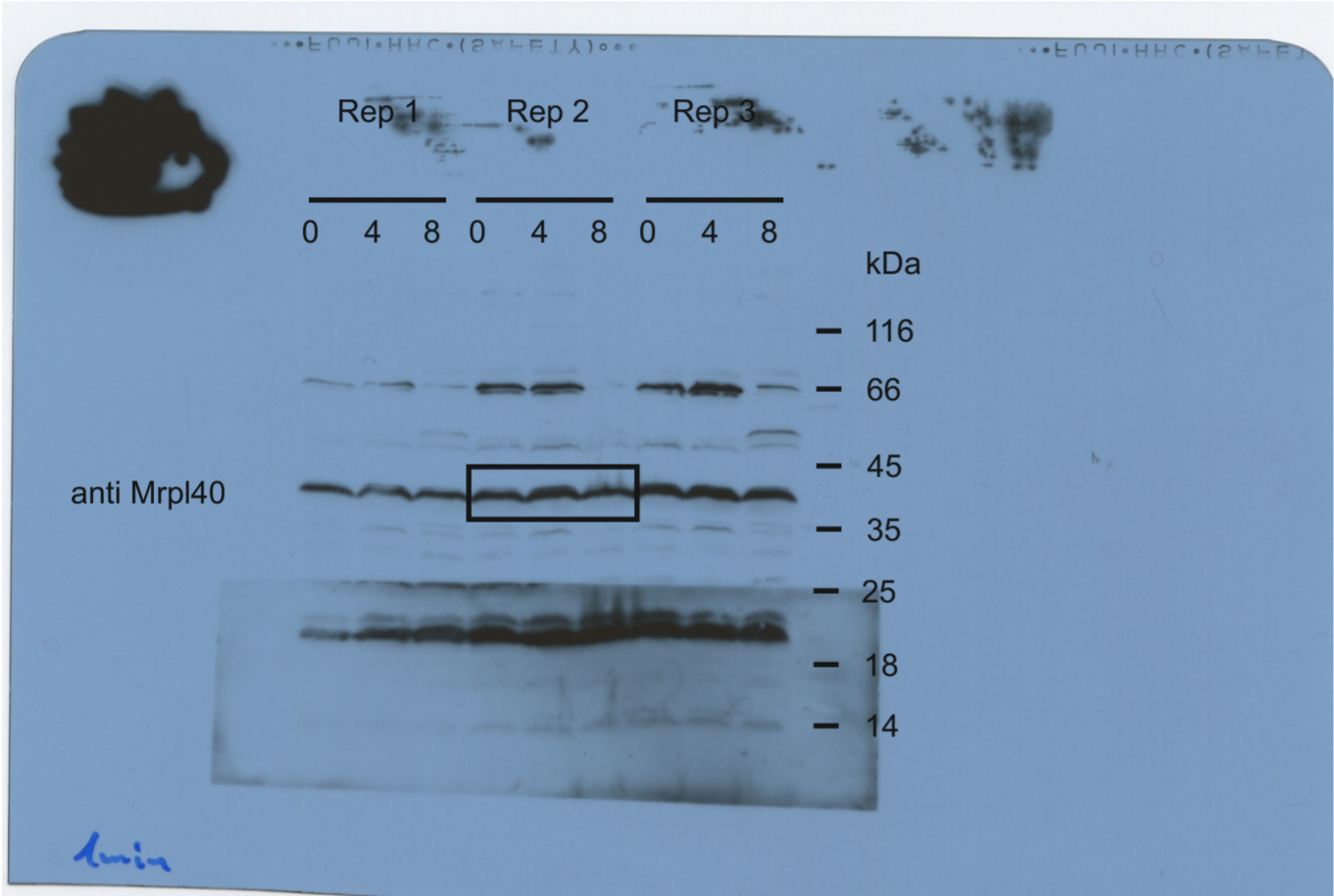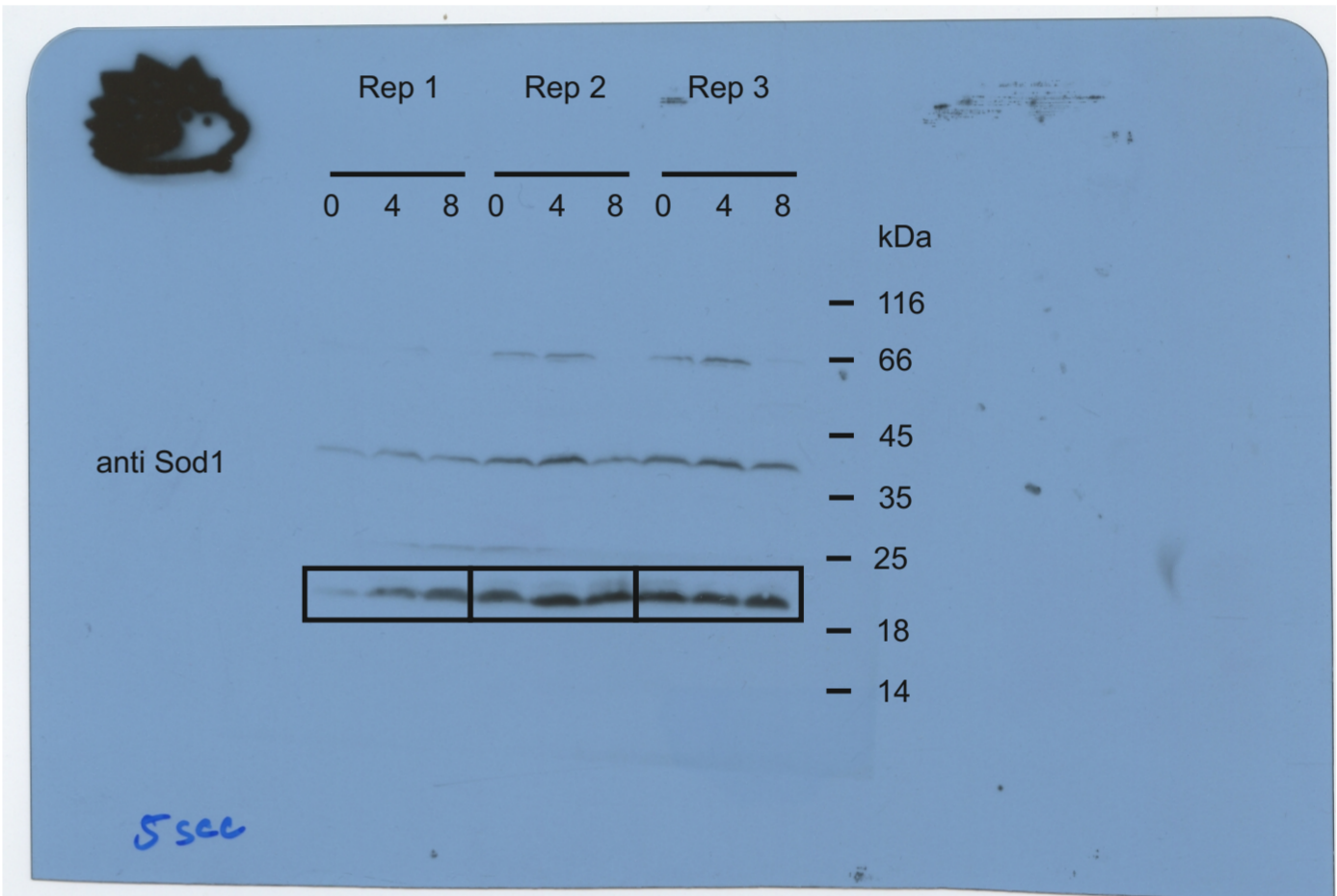

Rep 2 was shown in the Figure. Rep 1 and 3 were only used for quantification

Supplement: Supplementary file 10 — Source data Fig. 2 [file 44319_2024_113_MOESM10_ESM.zip › Koch_SourceData_Fig2/2C-D/2C.pdf]

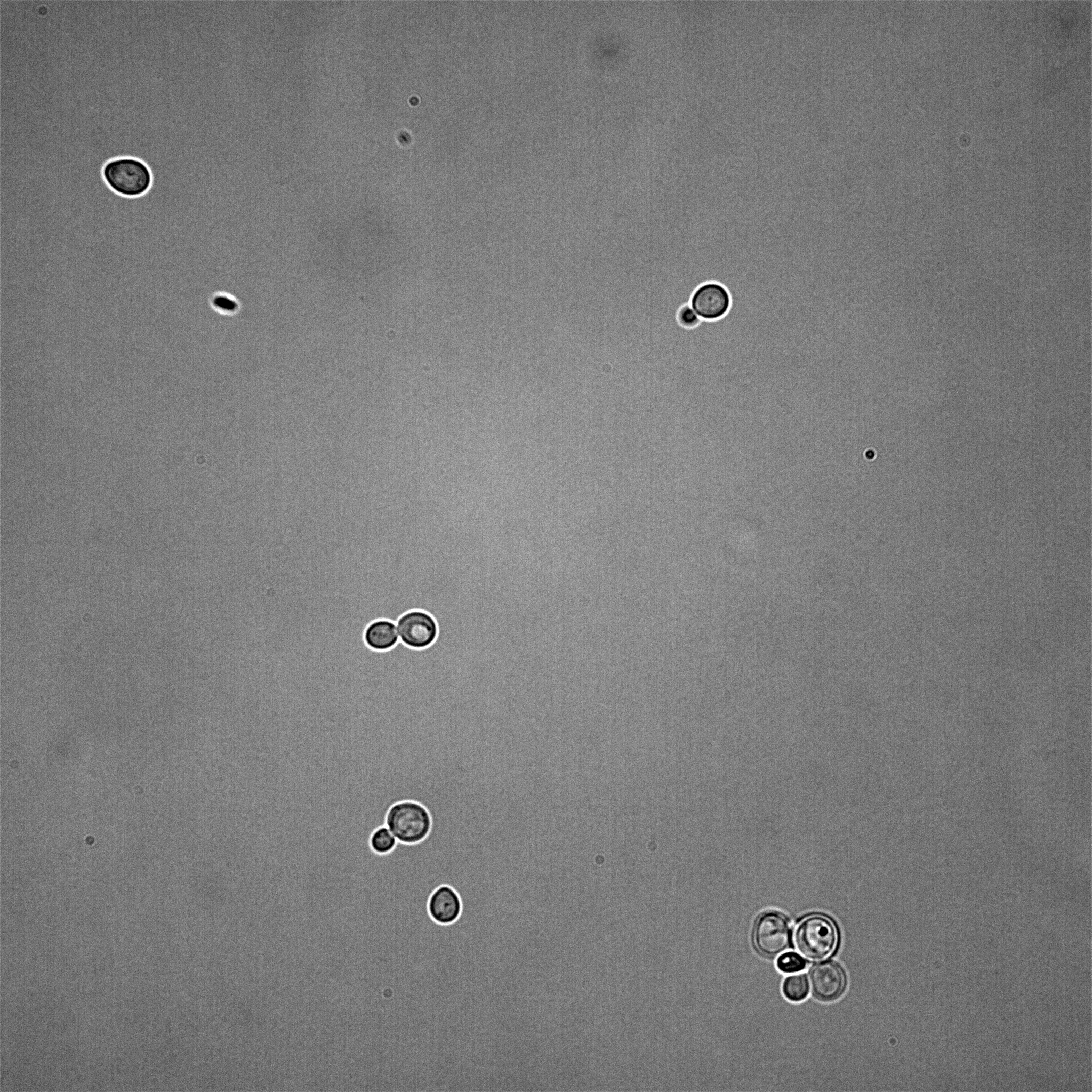

Supplement: Supplementary file 10 — Source data Fig. 2 [file 44319_2024_113_MOESM10_ESM.zip › Koch_SourceData_Fig2/2E/MDM34_0h_BF.tif]

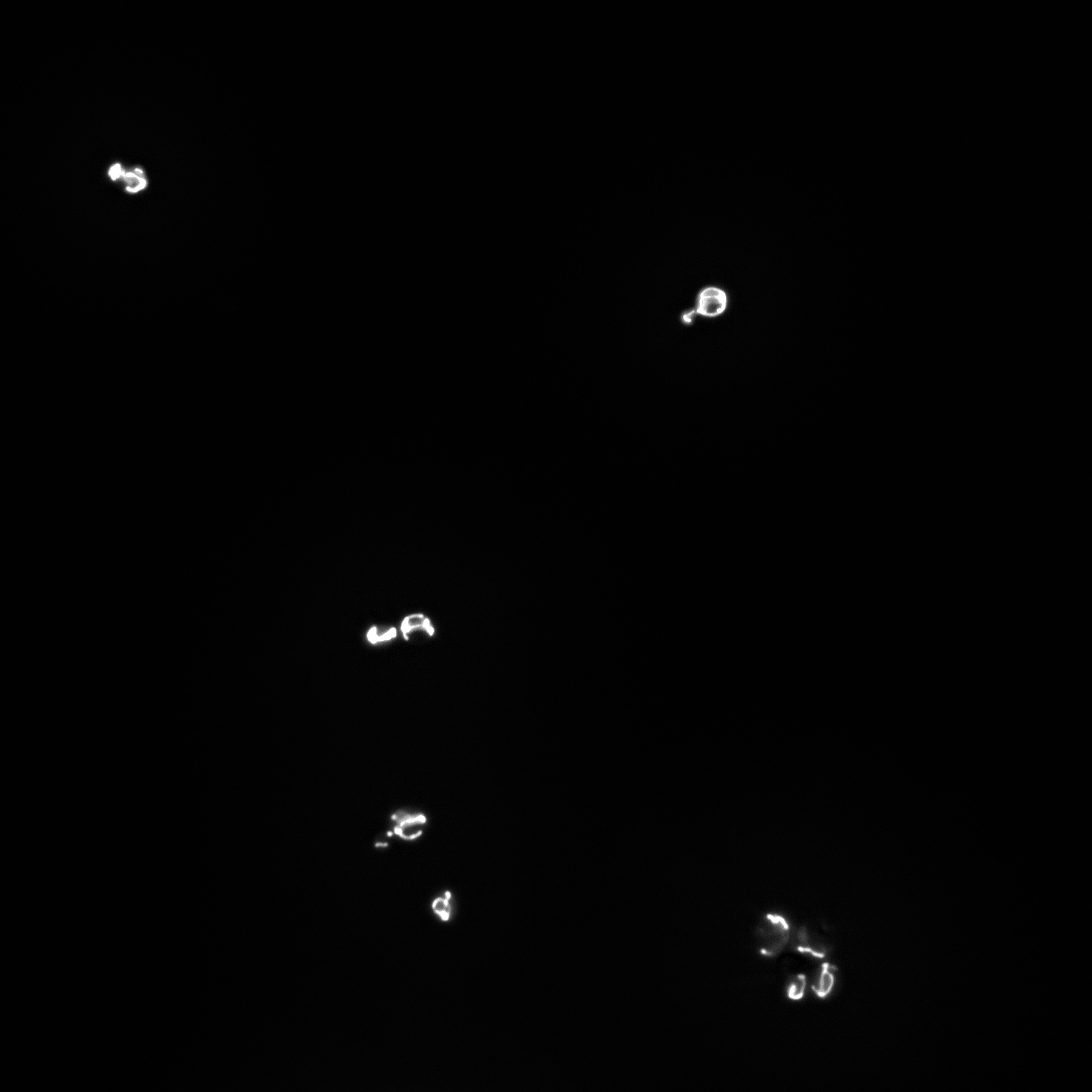

Supplement: Supplementary file 10 — Source data Fig. 2 [file 44319_2024_113_MOESM10_ESM.zip › Koch_SourceData_Fig2/2E/MDM34_0h_NeonGreen.tif]

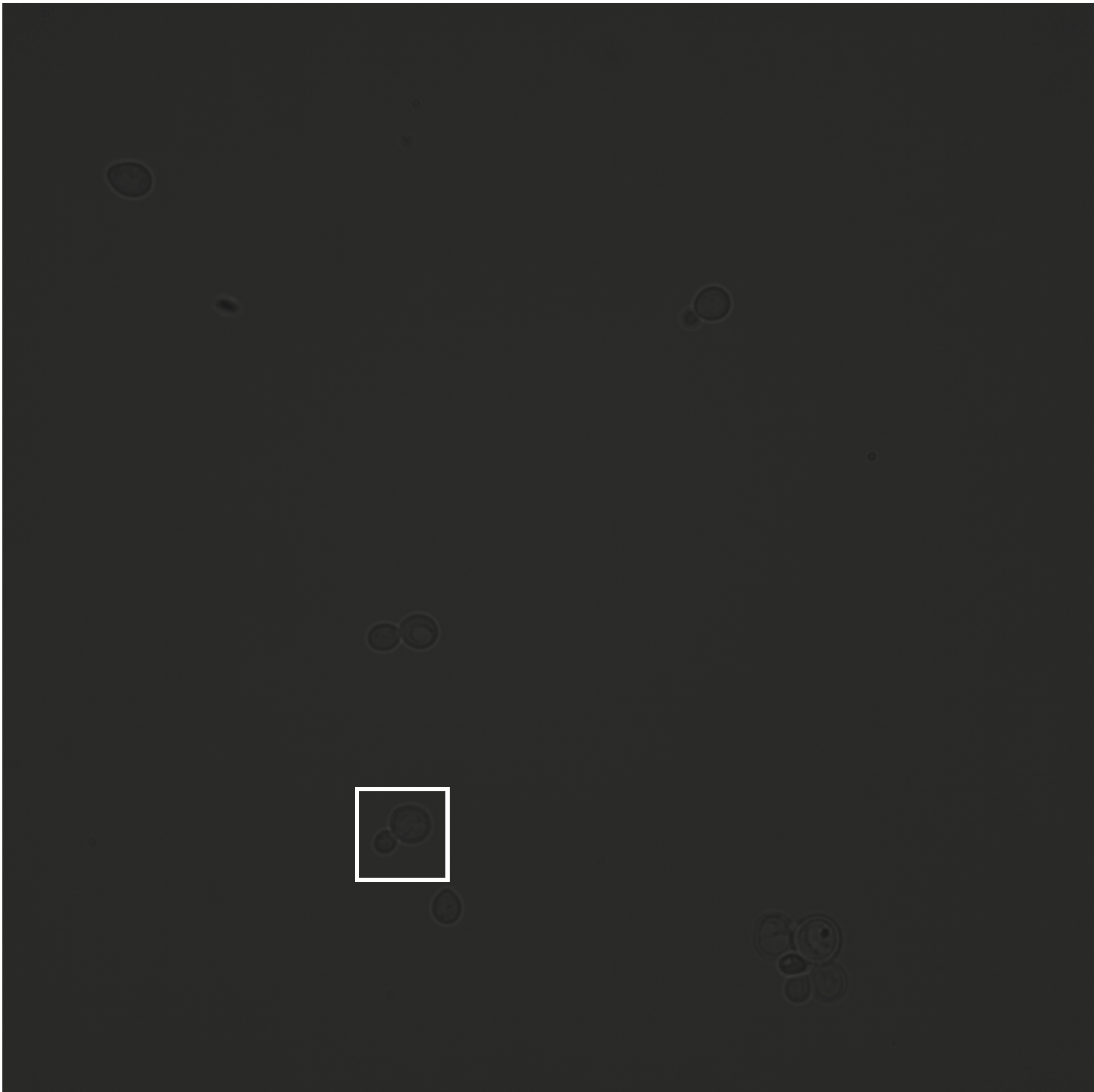

Supplement: Supplementary file 10 — Source data Fig. 2 [file 44319_2024_113_MOESM10_ESM.zip › Koch_SourceData_Fig2/2E/MDM34_0h_reference image.pdf]

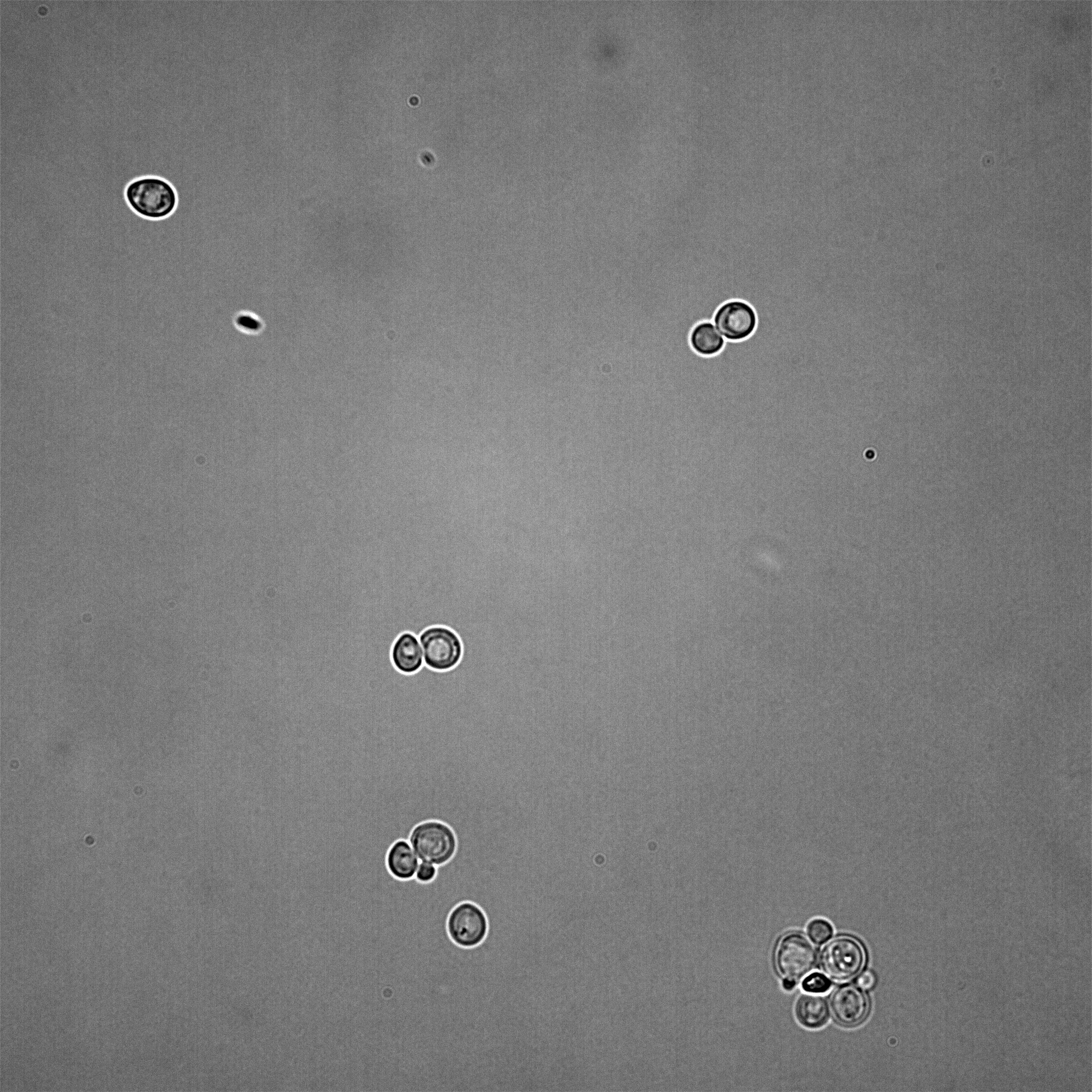

Supplement: Supplementary file 10 — Source data Fig. 2 [file 44319_2024_113_MOESM10_ESM.zip › Koch_SourceData_Fig2/2E/MDM34_4h_BF.tif]

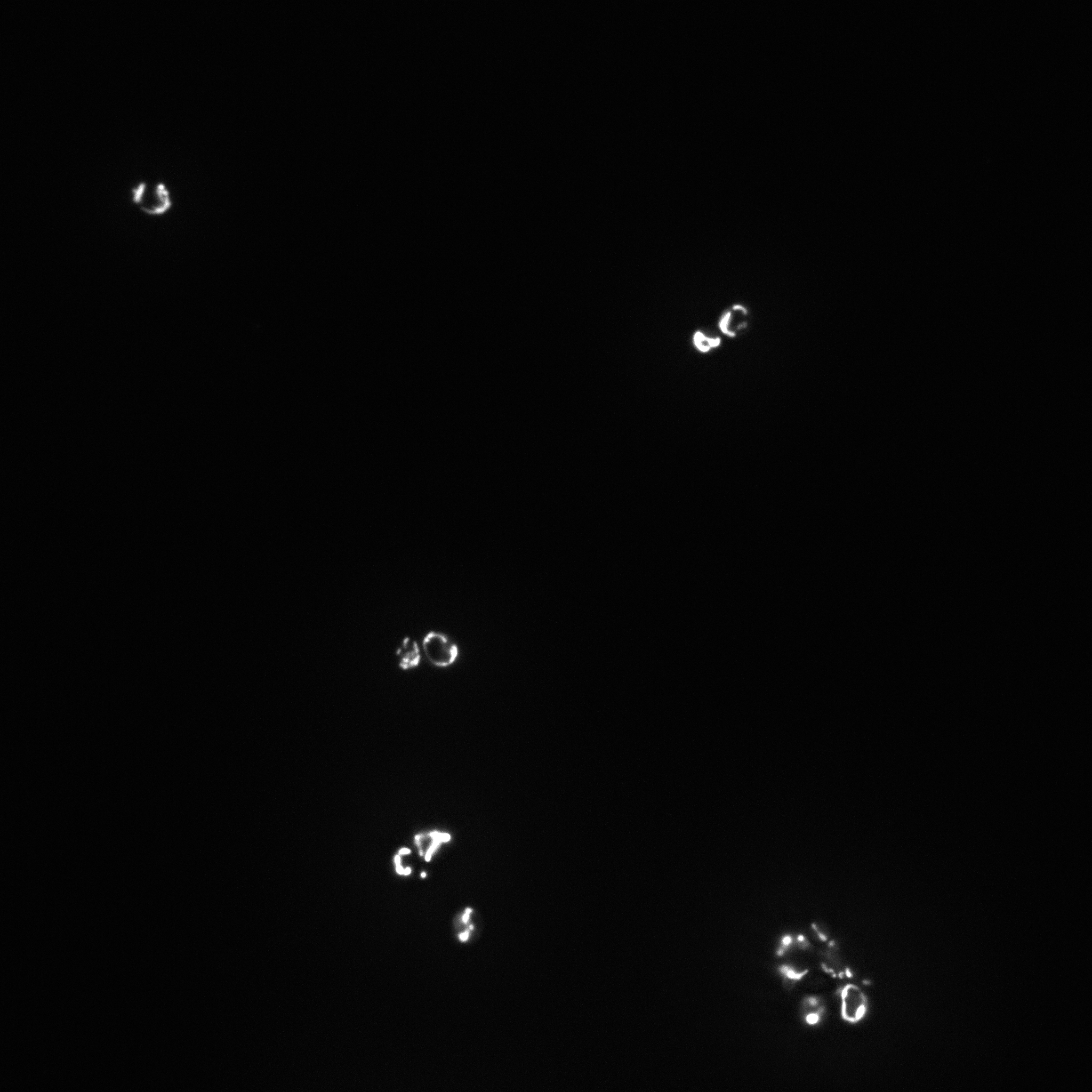

Supplement: Supplementary file 10 — Source data Fig. 2 [file 44319_2024_113_MOESM10_ESM.zip › Koch_SourceData_Fig2/2E/MDM34_4h_NeonGreen.tif]

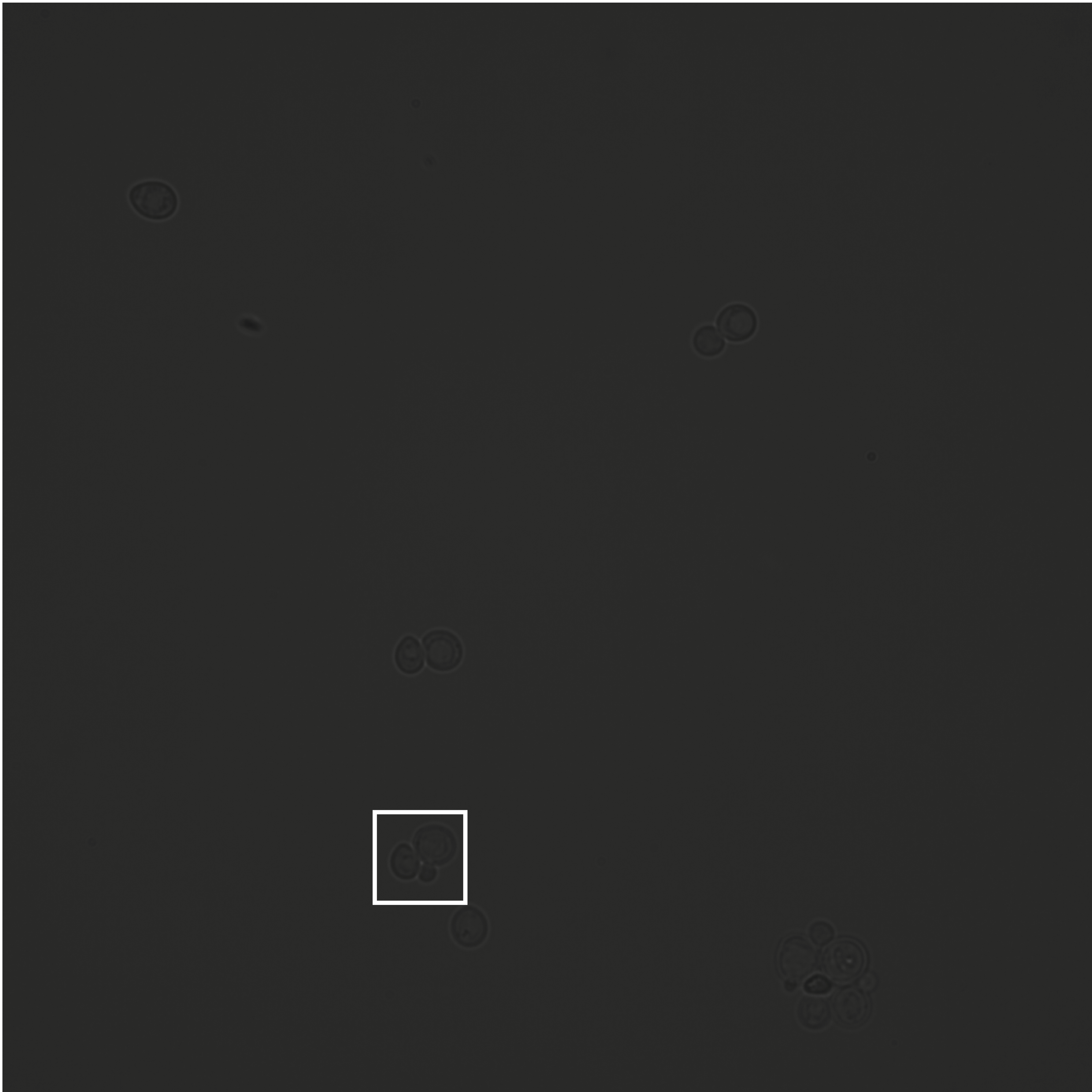

Supplement: Supplementary file 10 — Source data Fig. 2 [file 44319_2024_113_MOESM10_ESM.zip › Koch_SourceData_Fig2/2E/MDM34_4h_reference image.pdf]

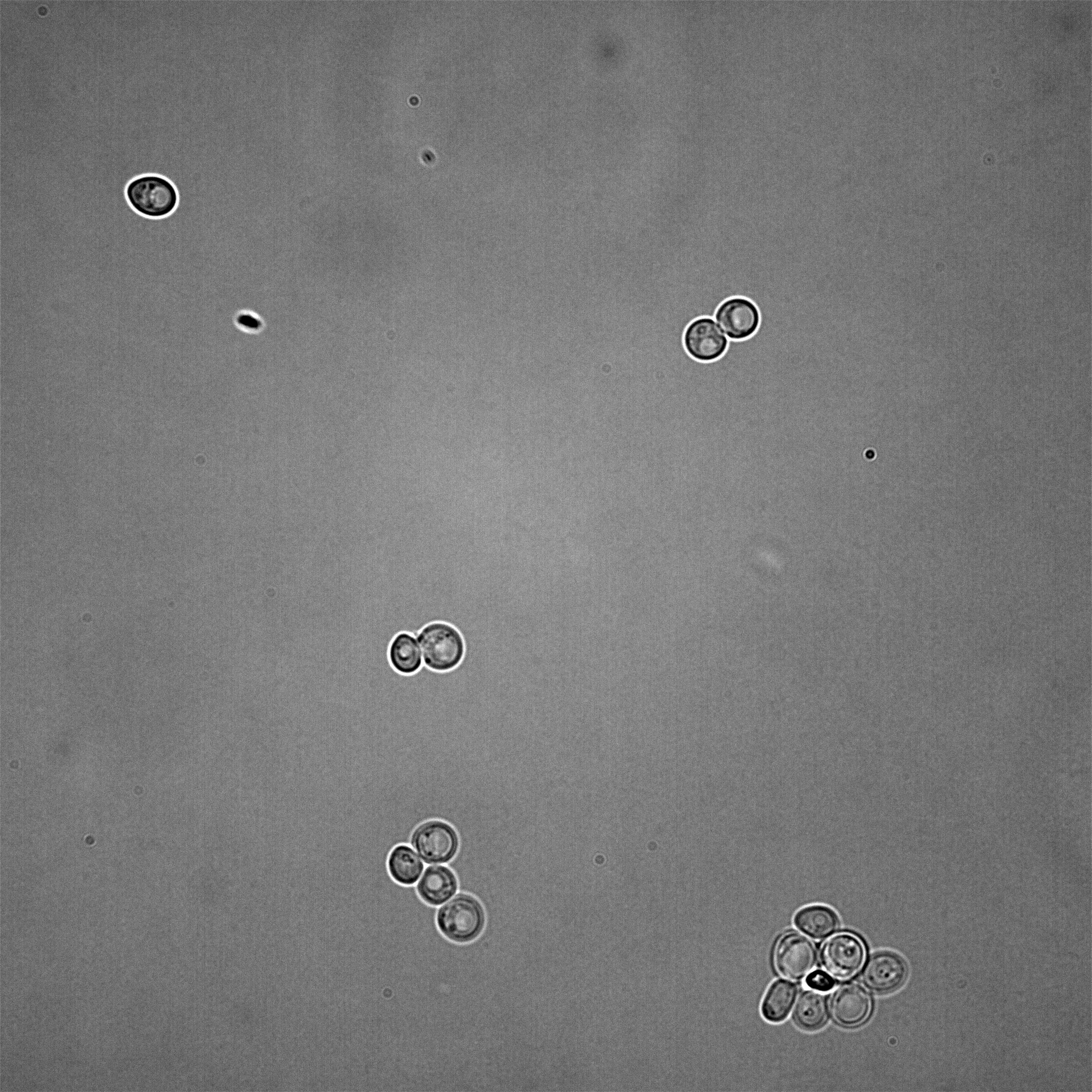

Supplement: Supplementary file 10 — Source data Fig. 2 [file 44319_2024_113_MOESM10_ESM.zip › Koch_SourceData_Fig2/2E/MDM34_8h_BF.tif]

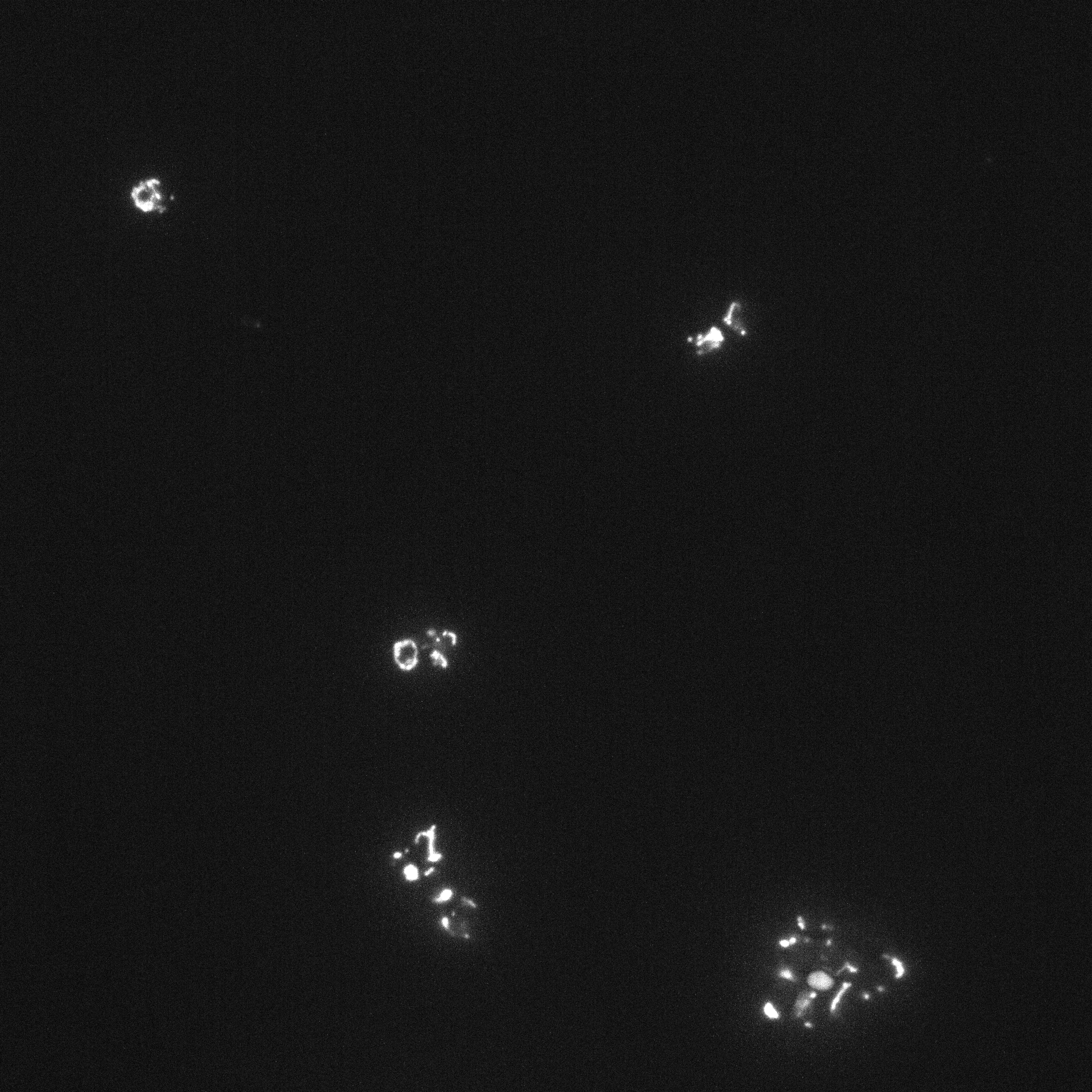

Supplement: Supplementary file 10 — Source data Fig. 2 [file 44319_2024_113_MOESM10_ESM.zip › Koch_SourceData_Fig2/2E/MDM34_8h_NeonGreen.tif]

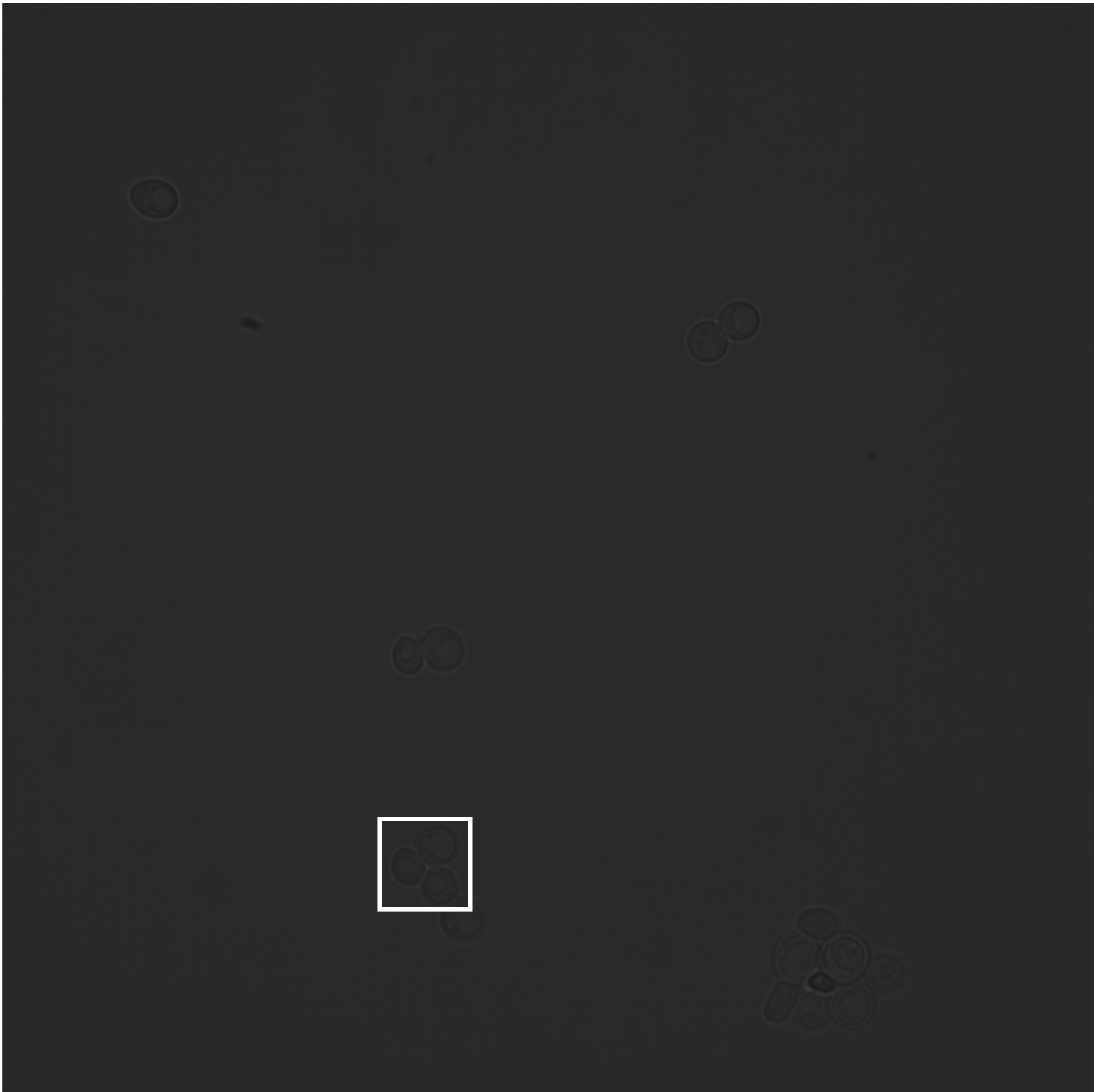

Supplement: Supplementary file 10 — Source data Fig. 2 [file 44319_2024_113_MOESM10_ESM.zip › Koch_SourceData_Fig2/2E/MDM34_8h_reference image.pdf]

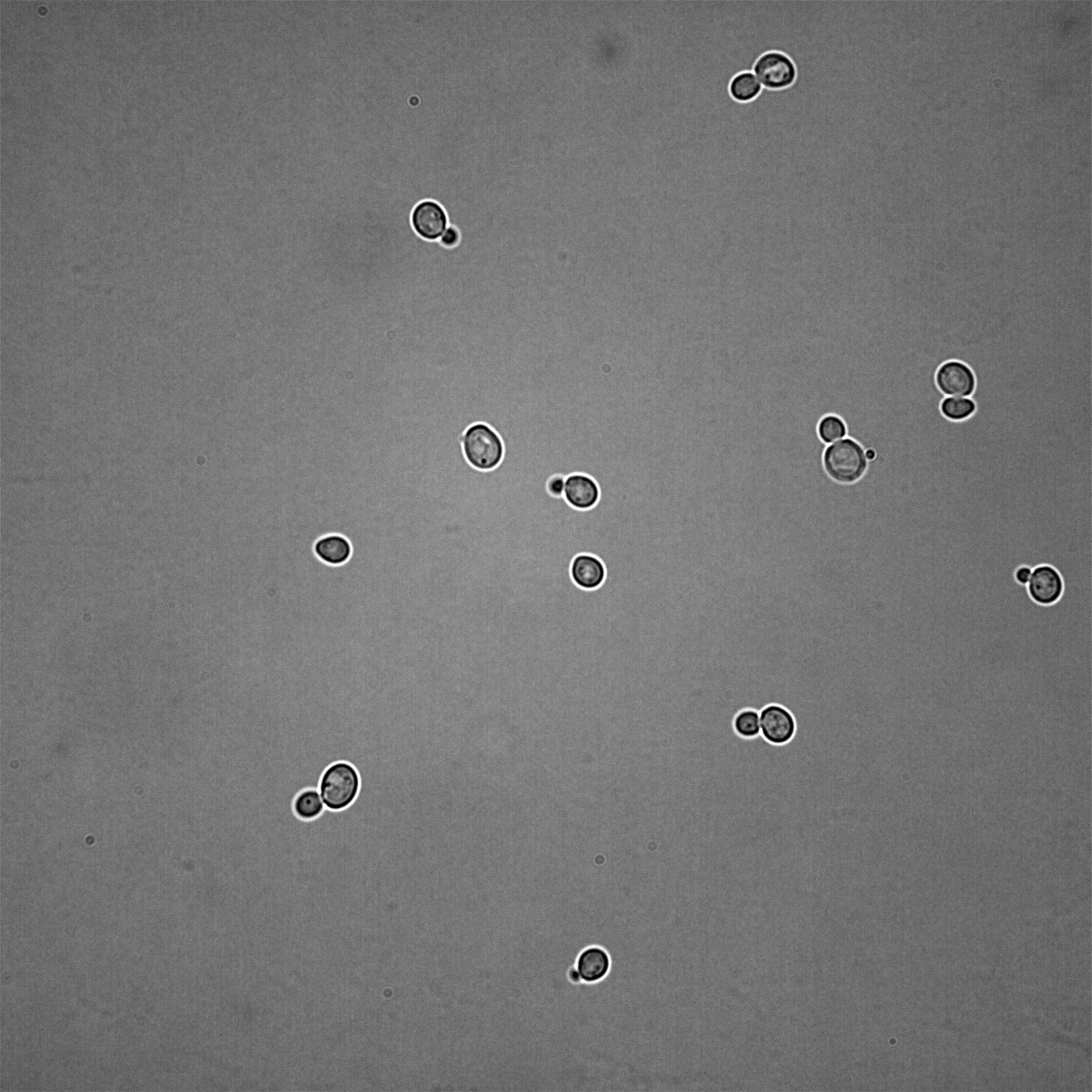

Supplement: Supplementary file 10 — Source data Fig. 2 [file 44319_2024_113_MOESM10_ESM.zip › Koch_SourceData_Fig2/2E/WT_0h_BF.tif]

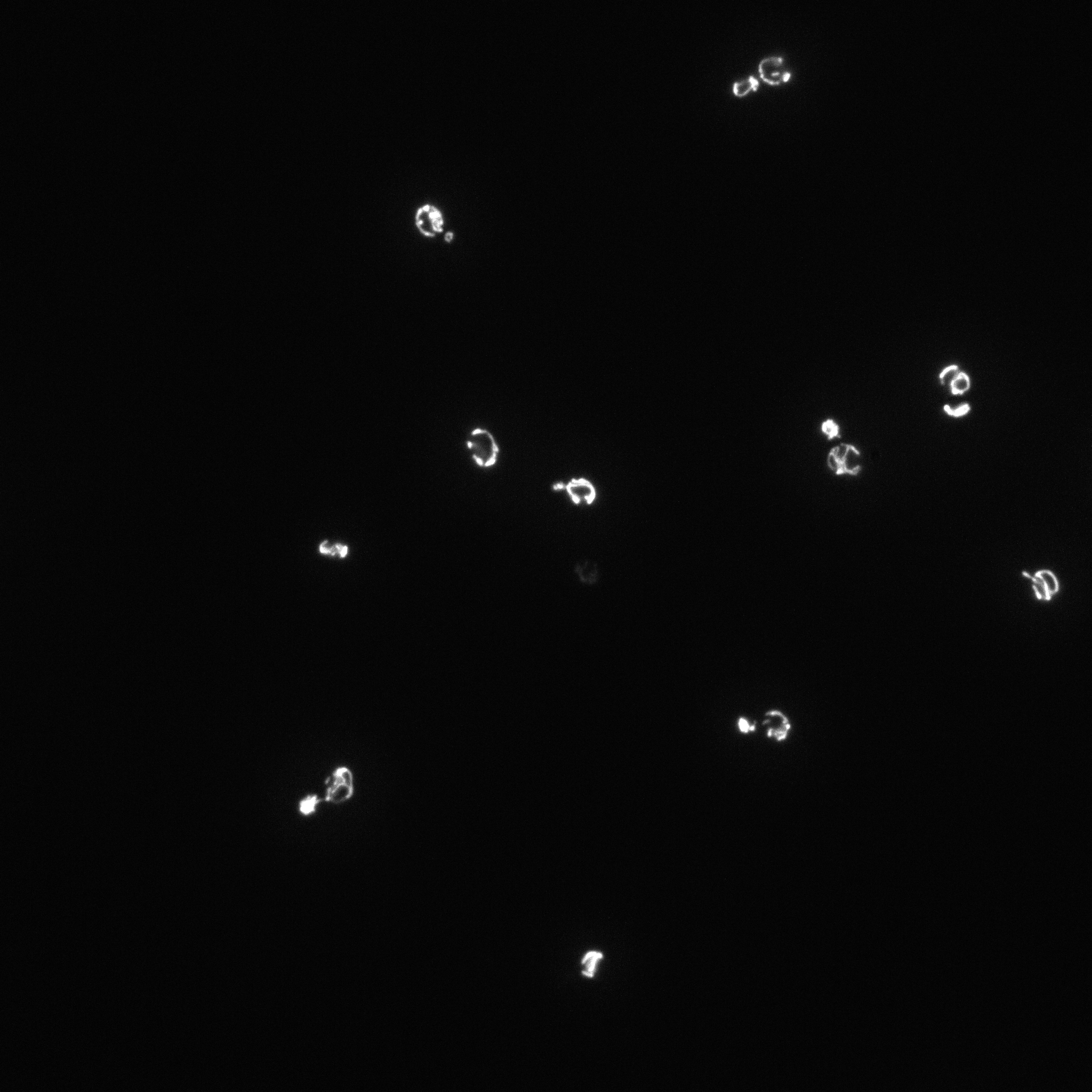

Supplement: Supplementary file 10 — Source data Fig. 2 [file 44319_2024_113_MOESM10_ESM.zip › Koch_SourceData_Fig2/2E/WT_0h_NeonGreen.tif]

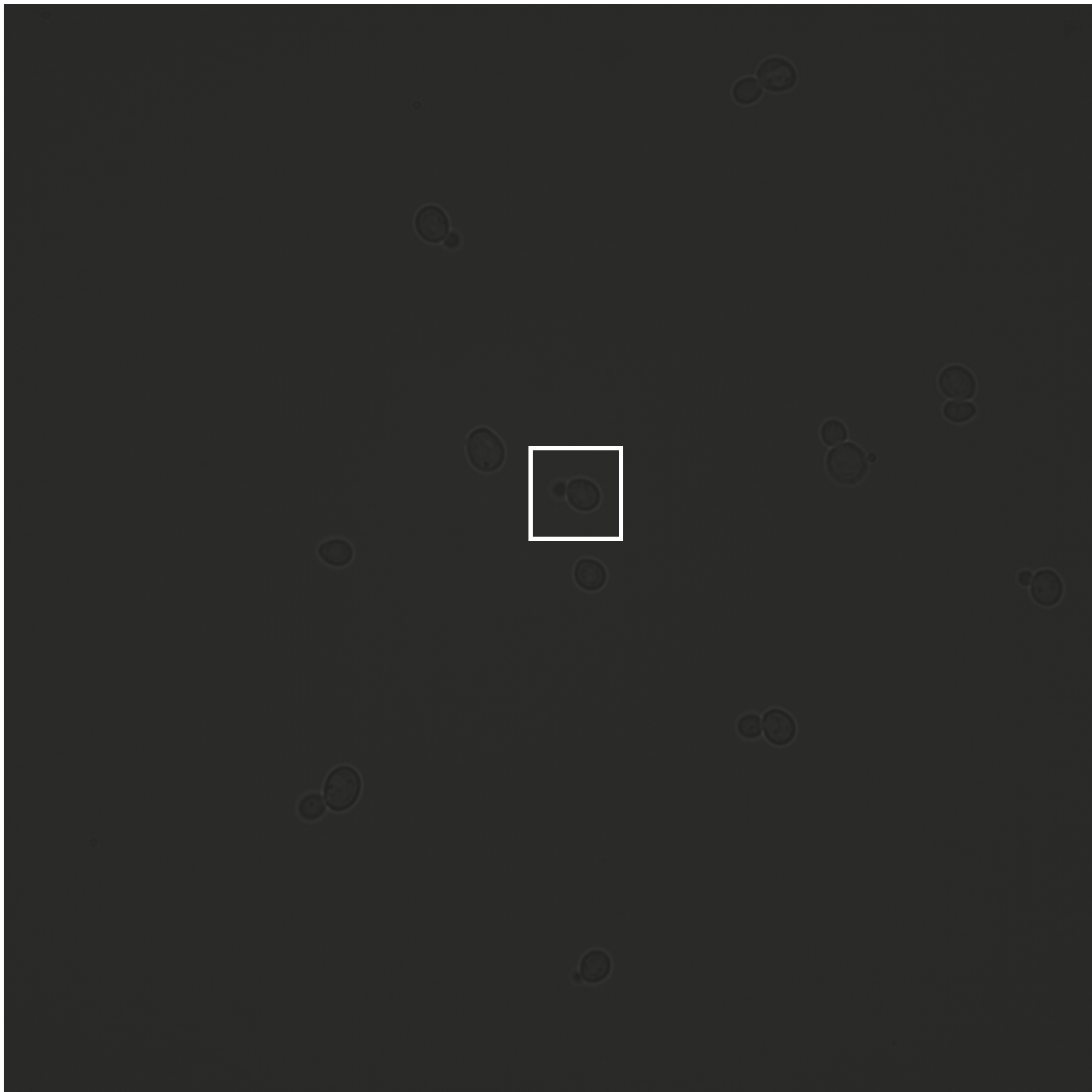

Supplement: Supplementary file 10 — Source data Fig. 2 [file 44319_2024_113_MOESM10_ESM.zip › Koch_SourceData_Fig2/2E/WT_0h_reference image.pdf]

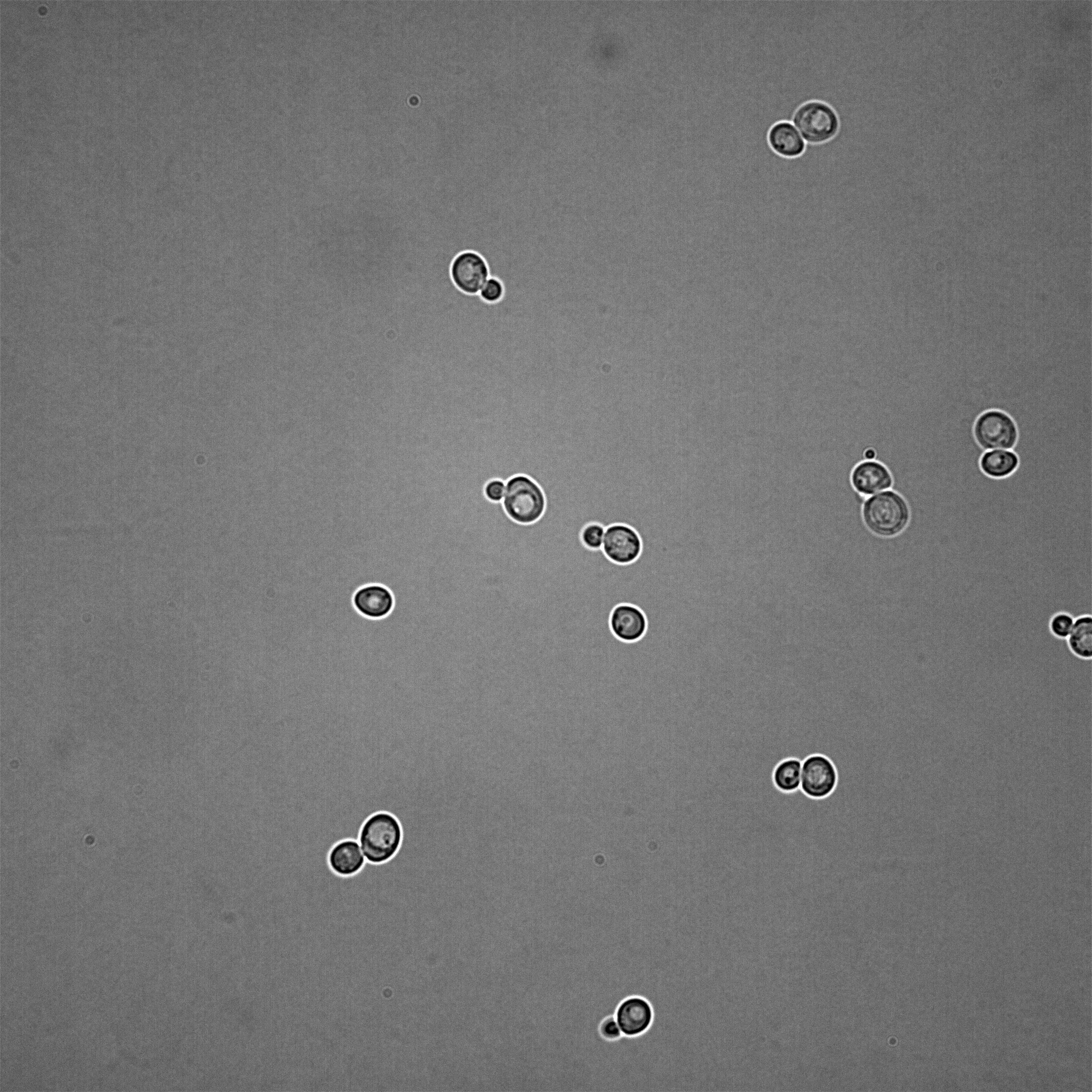

Supplement: Supplementary file 10 — Source data Fig. 2 [file 44319_2024_113_MOESM10_ESM.zip › Koch_SourceData_Fig2/2E/WT_4h_BF.tif]

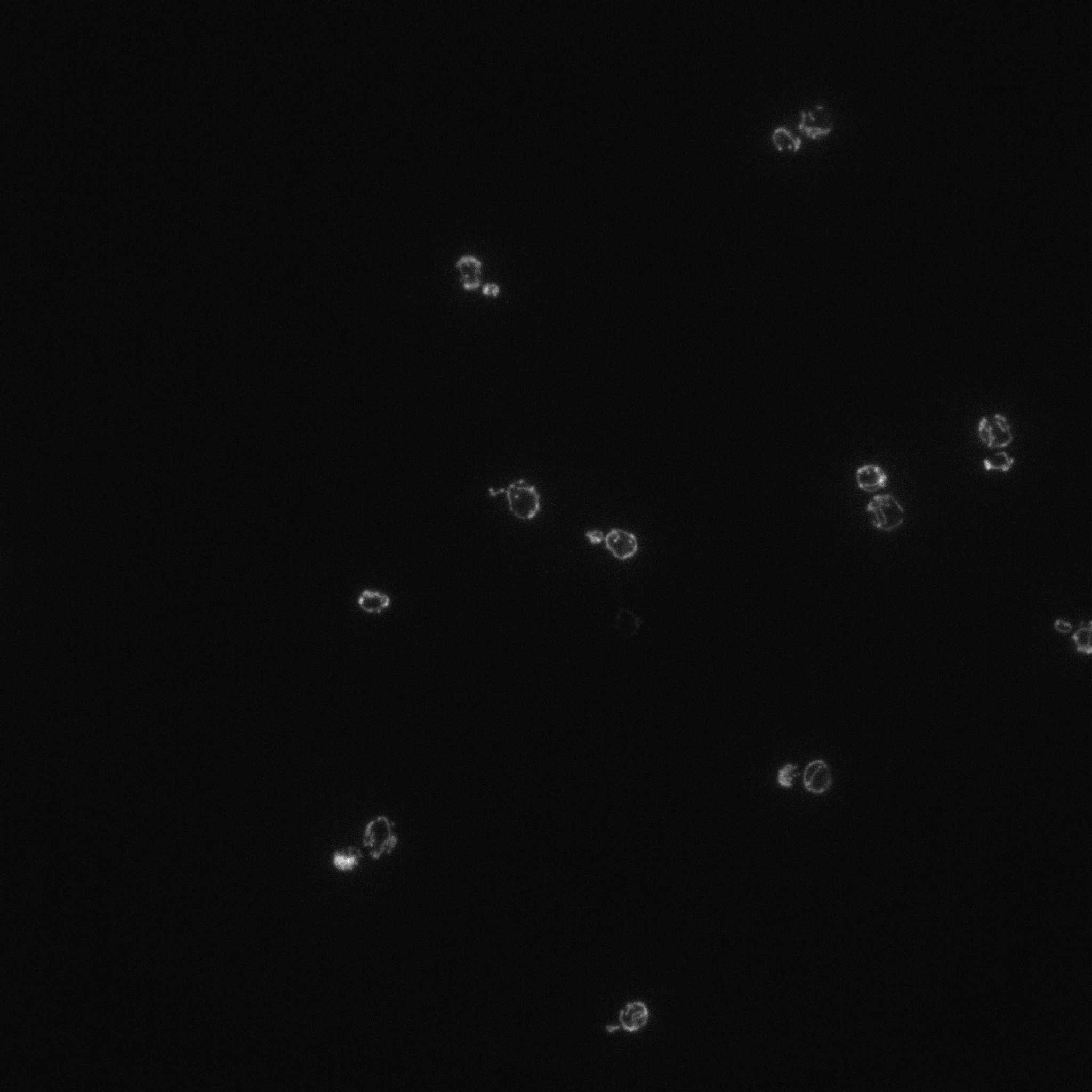

Supplement: Supplementary file 10 — Source data Fig. 2 [file 44319_2024_113_MOESM10_ESM.zip › Koch_SourceData_Fig2/2E/WT_4h_NeonGreen.tif]

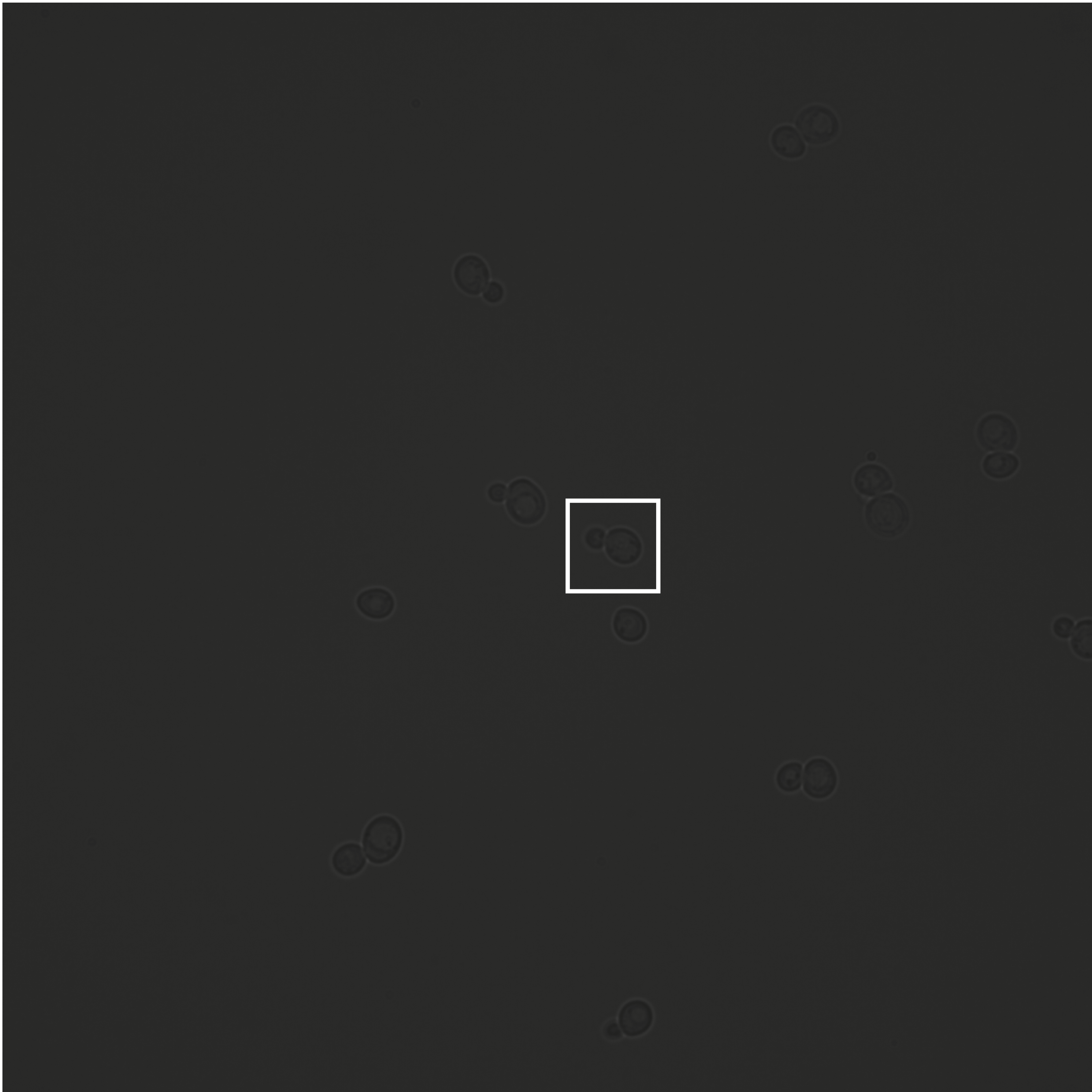

Supplement: Supplementary file 10 — Source data Fig. 2 [file 44319_2024_113_MOESM10_ESM.zip › Koch_SourceData_Fig2/2E/WT_4h_reference image.pdf]

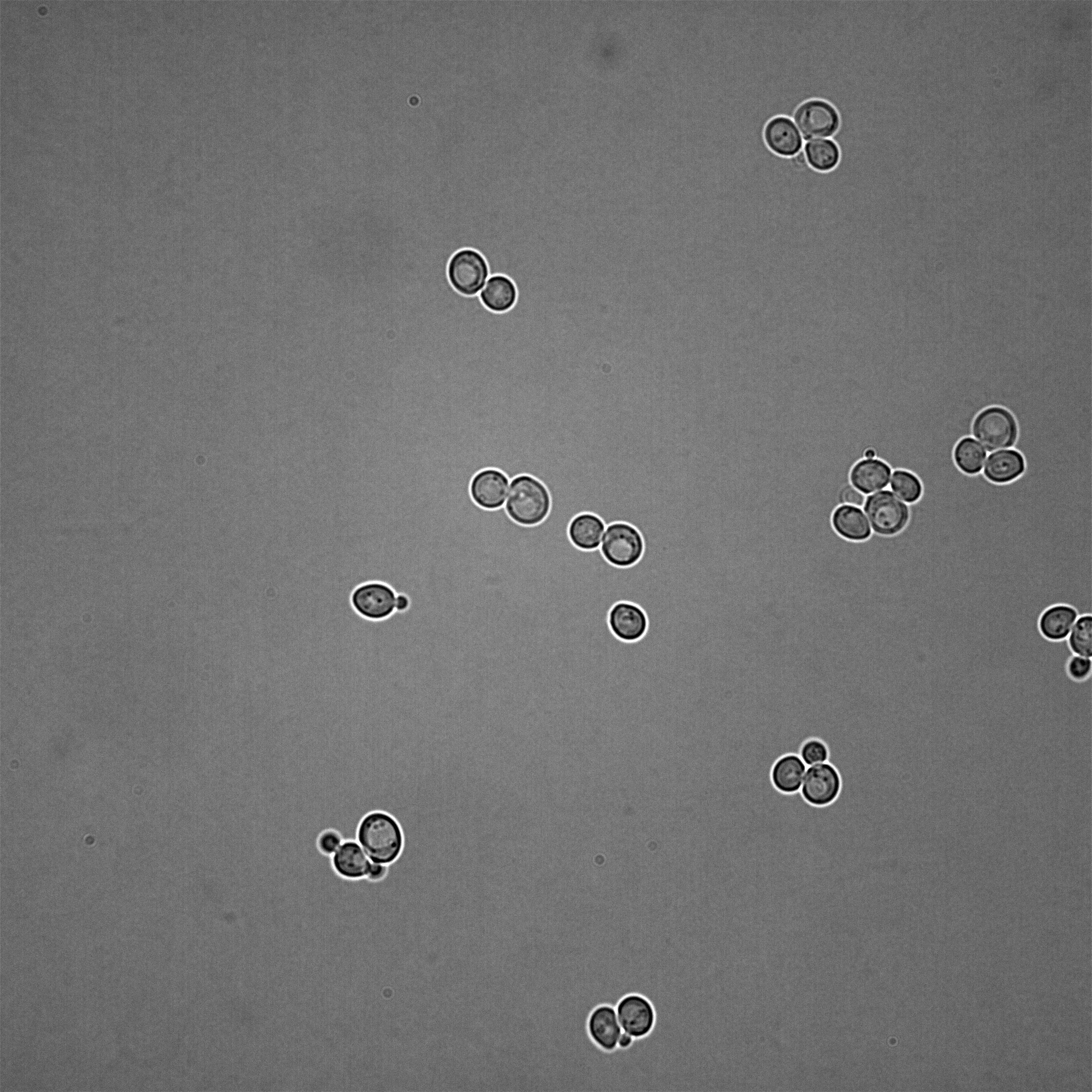

Supplement: Supplementary file 10 — Source data Fig. 2 [file 44319_2024_113_MOESM10_ESM.zip › Koch_SourceData_Fig2/2E/WT_8h_BF.tif]

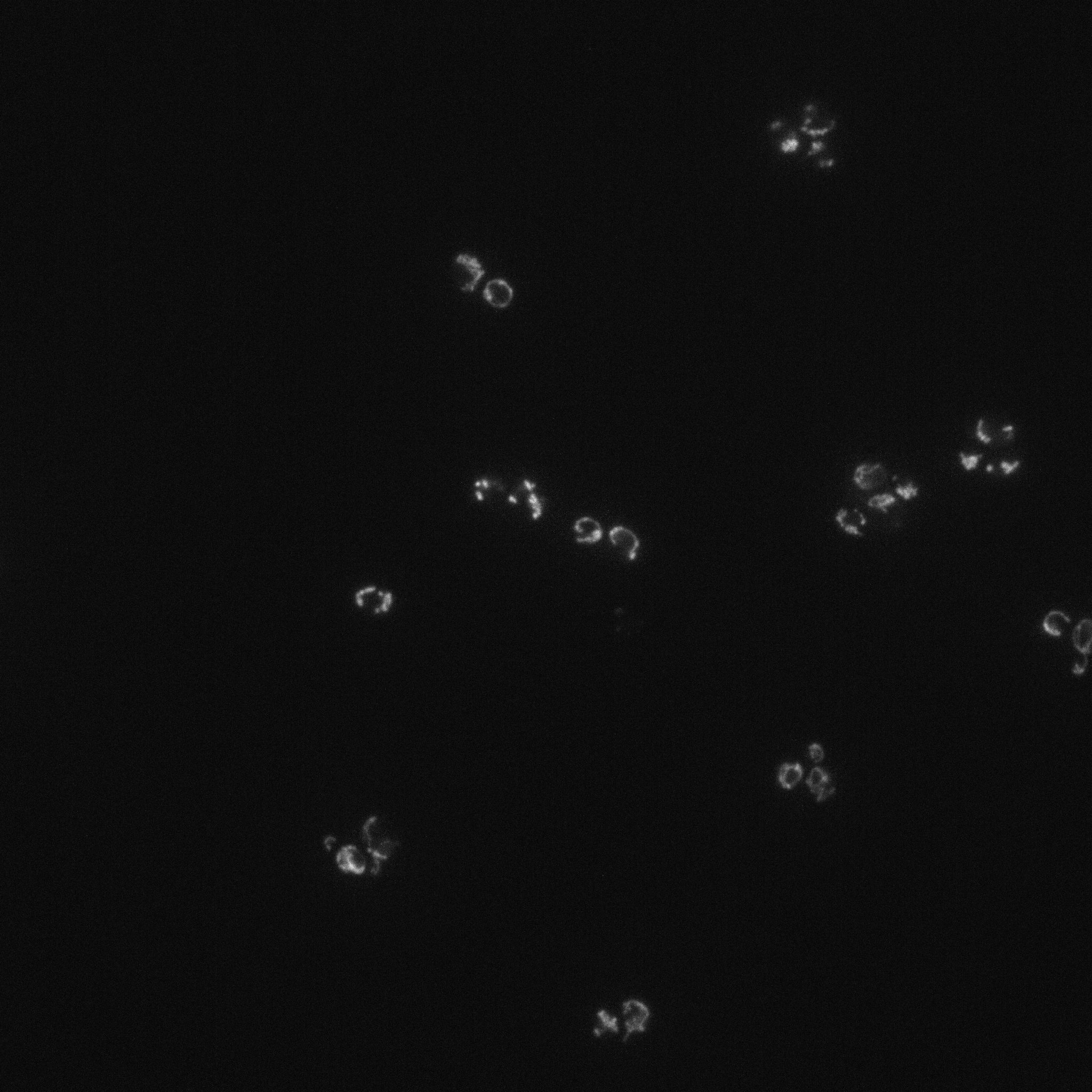

Supplement: Supplementary file 10 — Source data Fig. 2 [file 44319_2024_113_MOESM10_ESM.zip › Koch_SourceData_Fig2/2E/WT_8h_NeonGreen.tif]

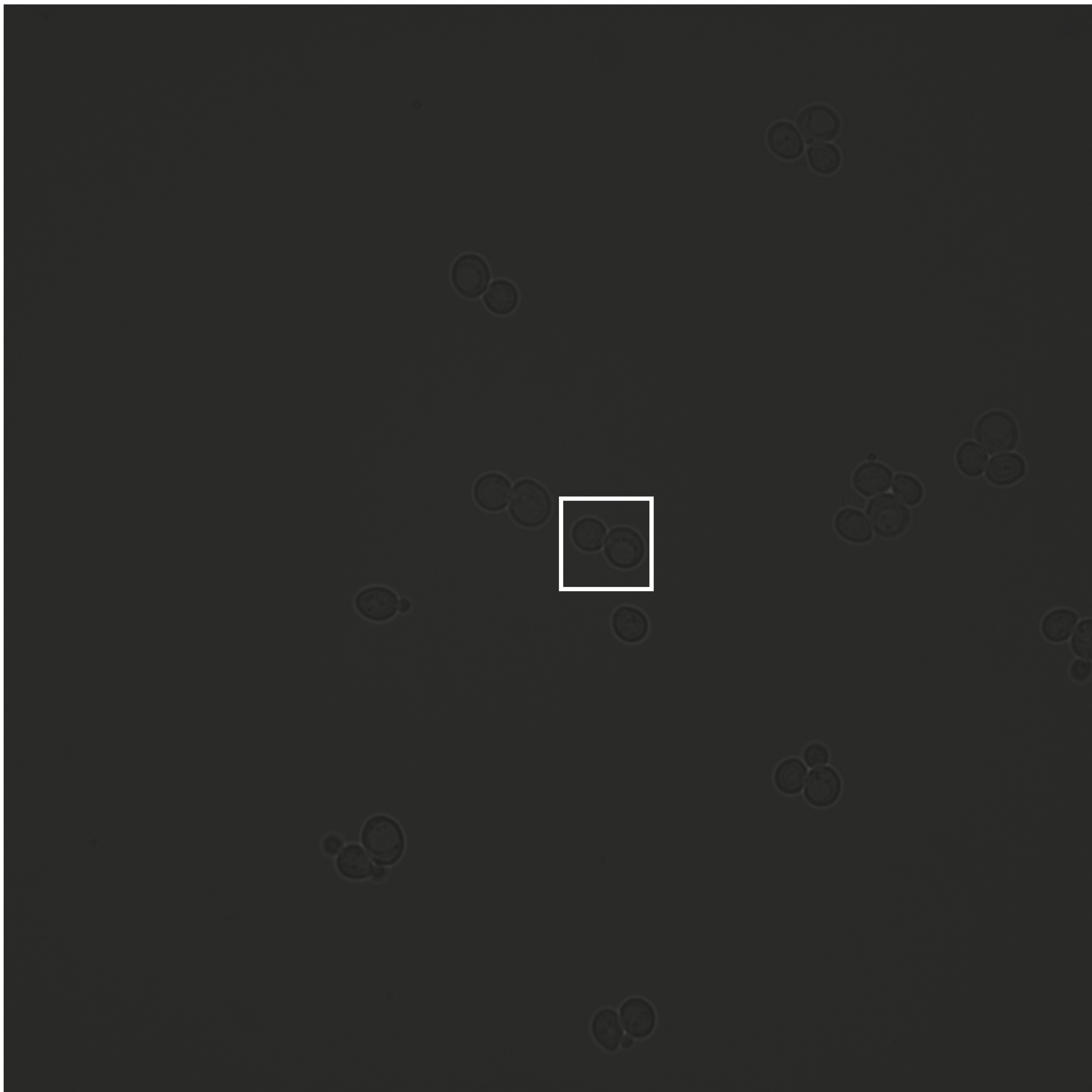

Supplement: Supplementary file 10 — Source data Fig. 2 [file 44319_2024_113_MOESM10_ESM.zip › Koch_SourceData_Fig2/2E/WT_8h_reference image.pdf]

### Figure 3B

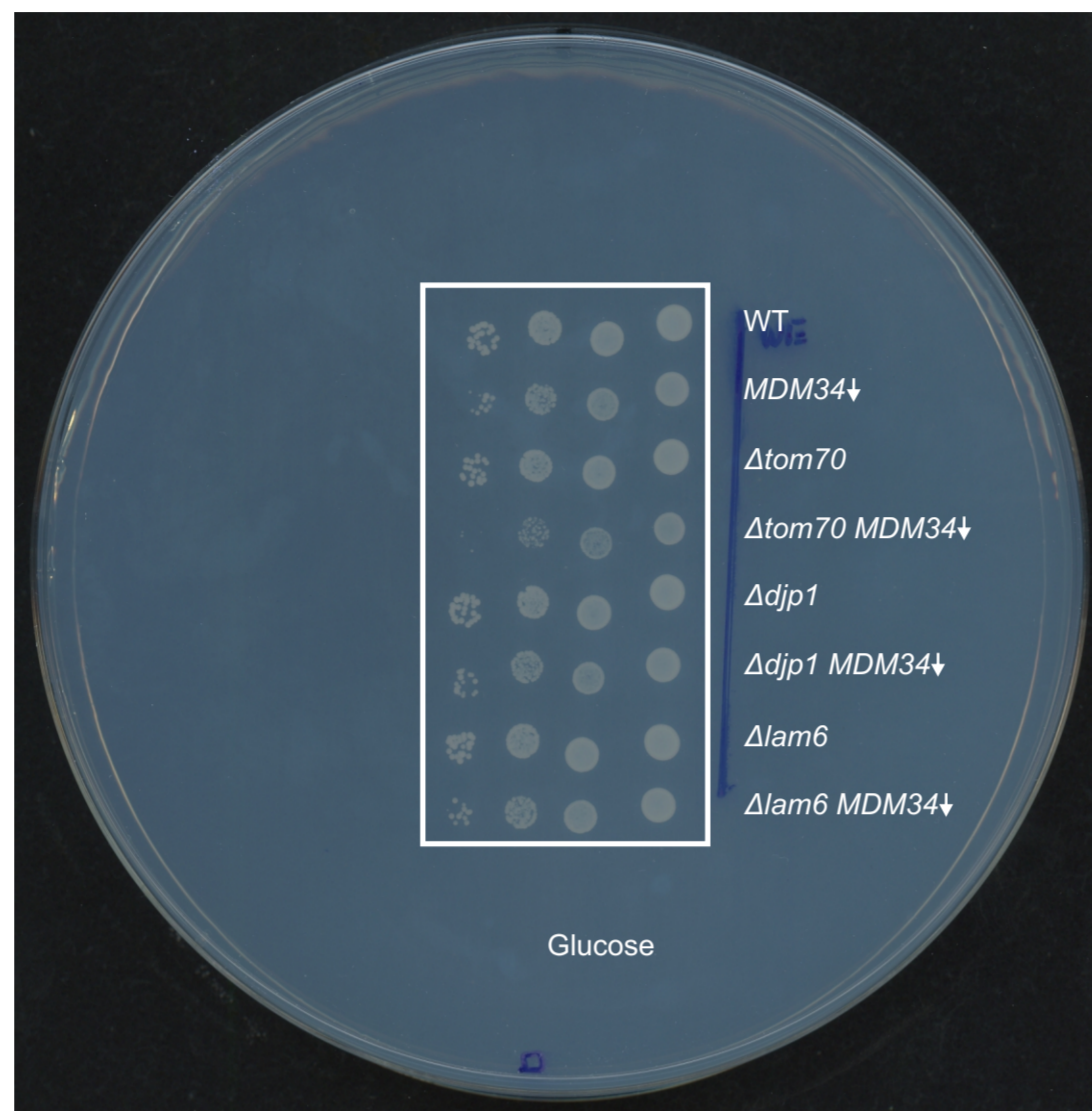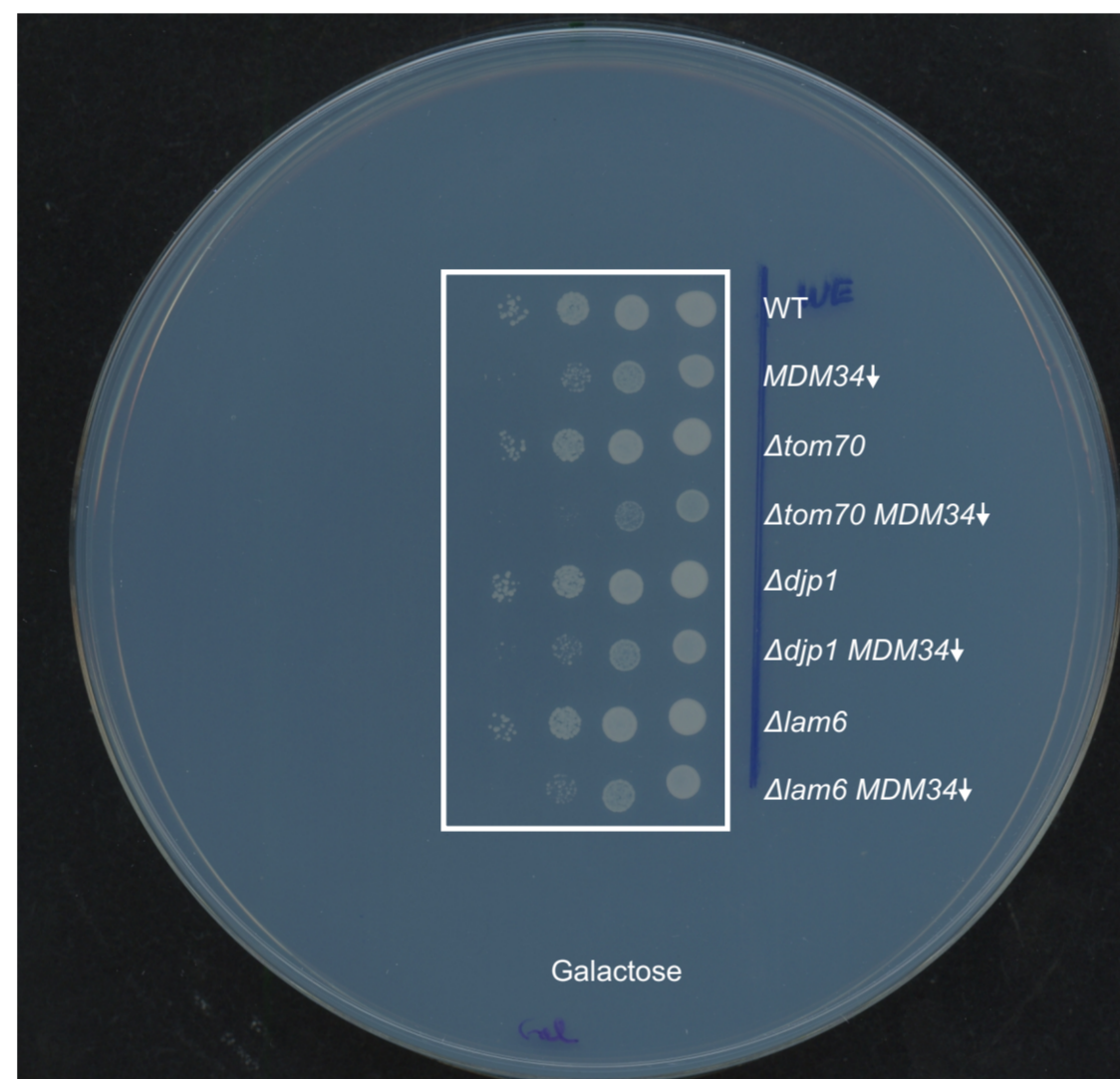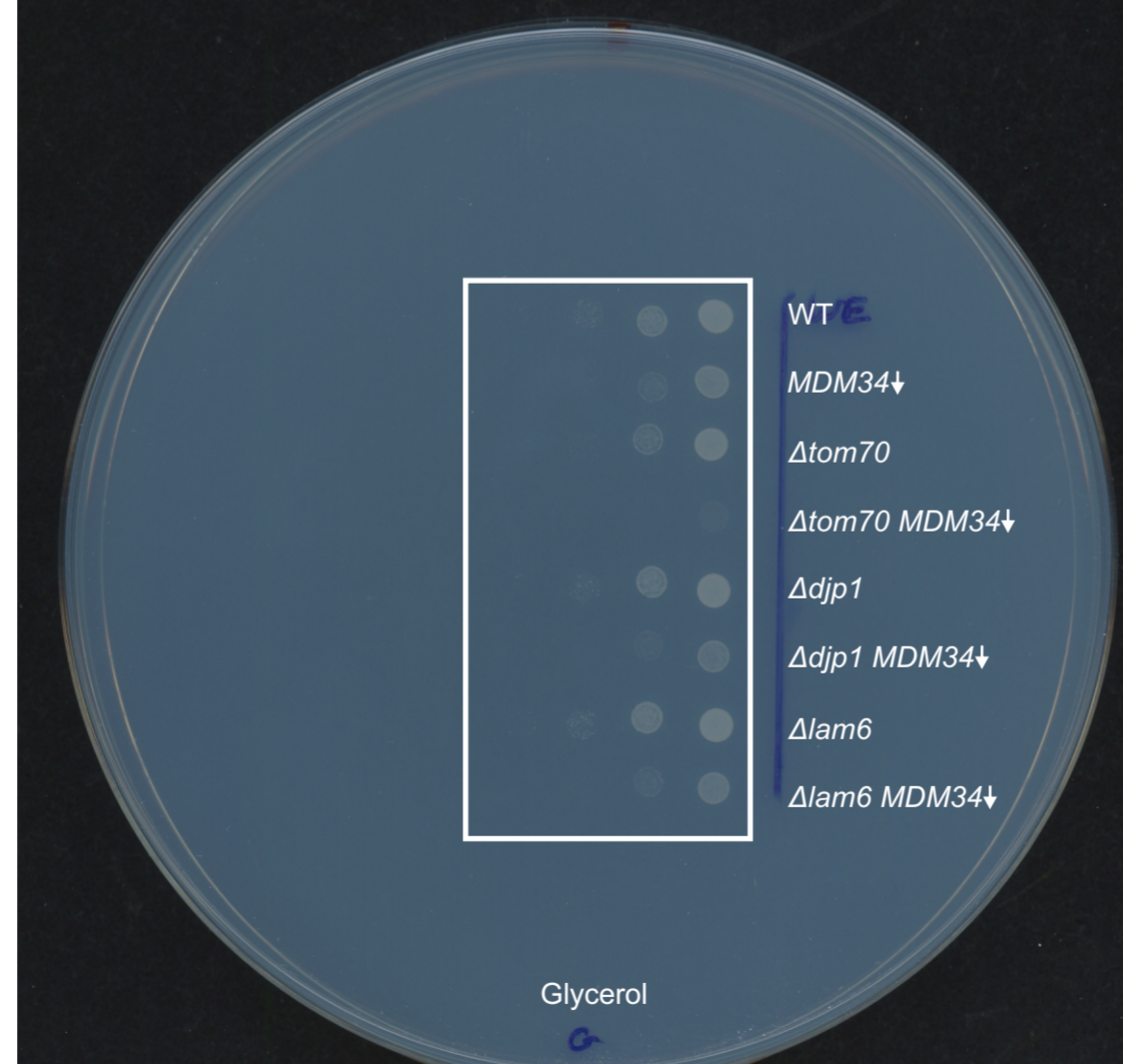

Supplement: Supplementary file 11 — Source data Fig. 3 [file 44319_2024_113_MOESM11_ESM.zip › Koch_SourceData_Fig3/3B/3B.pdf]

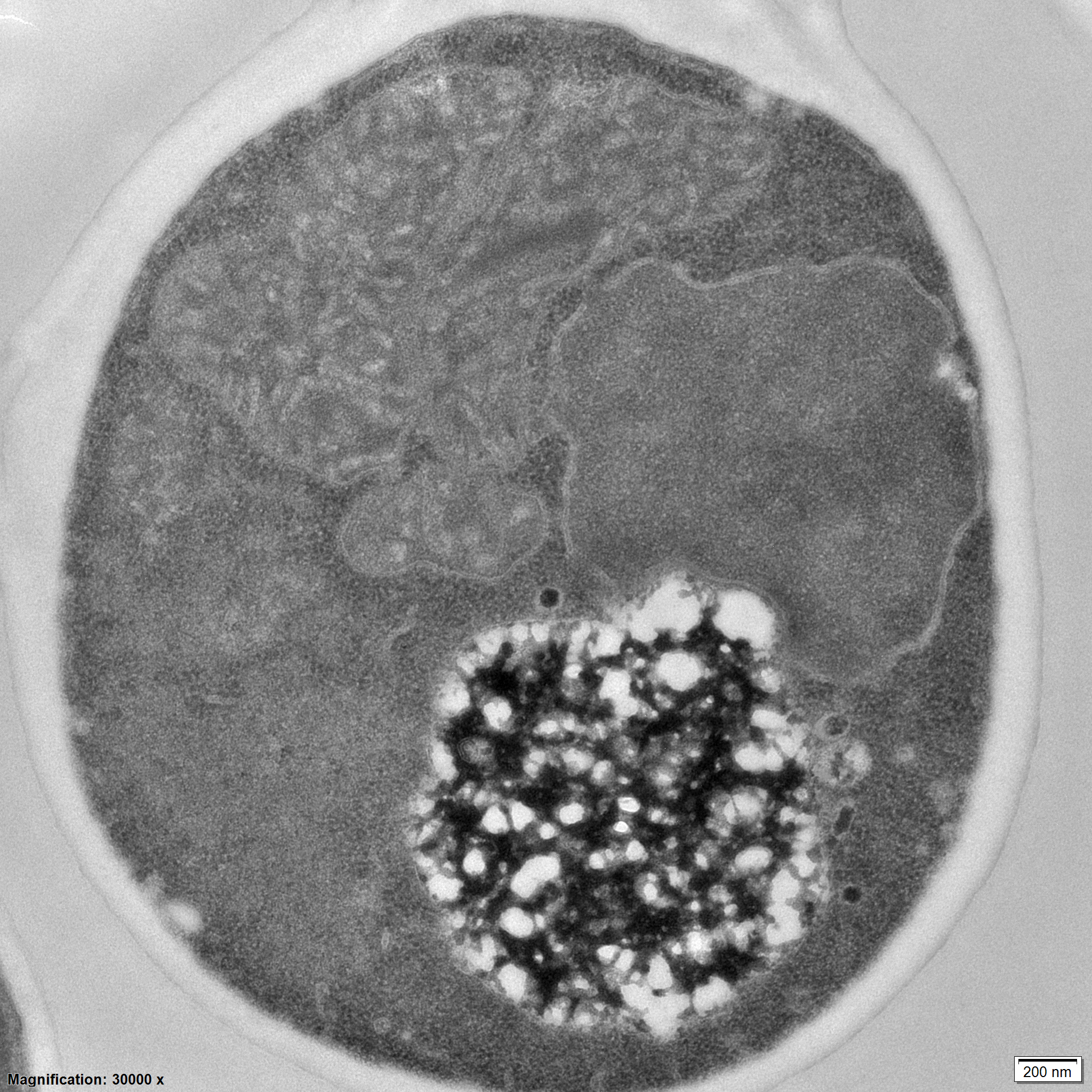

Supplement: Supplementary file 12 — Source data Fig. 4 [file 44319_2024_113_MOESM12_ESM.zip › Koch_SourceData_Fig4/4C/CRISPRi MDM34.tif]

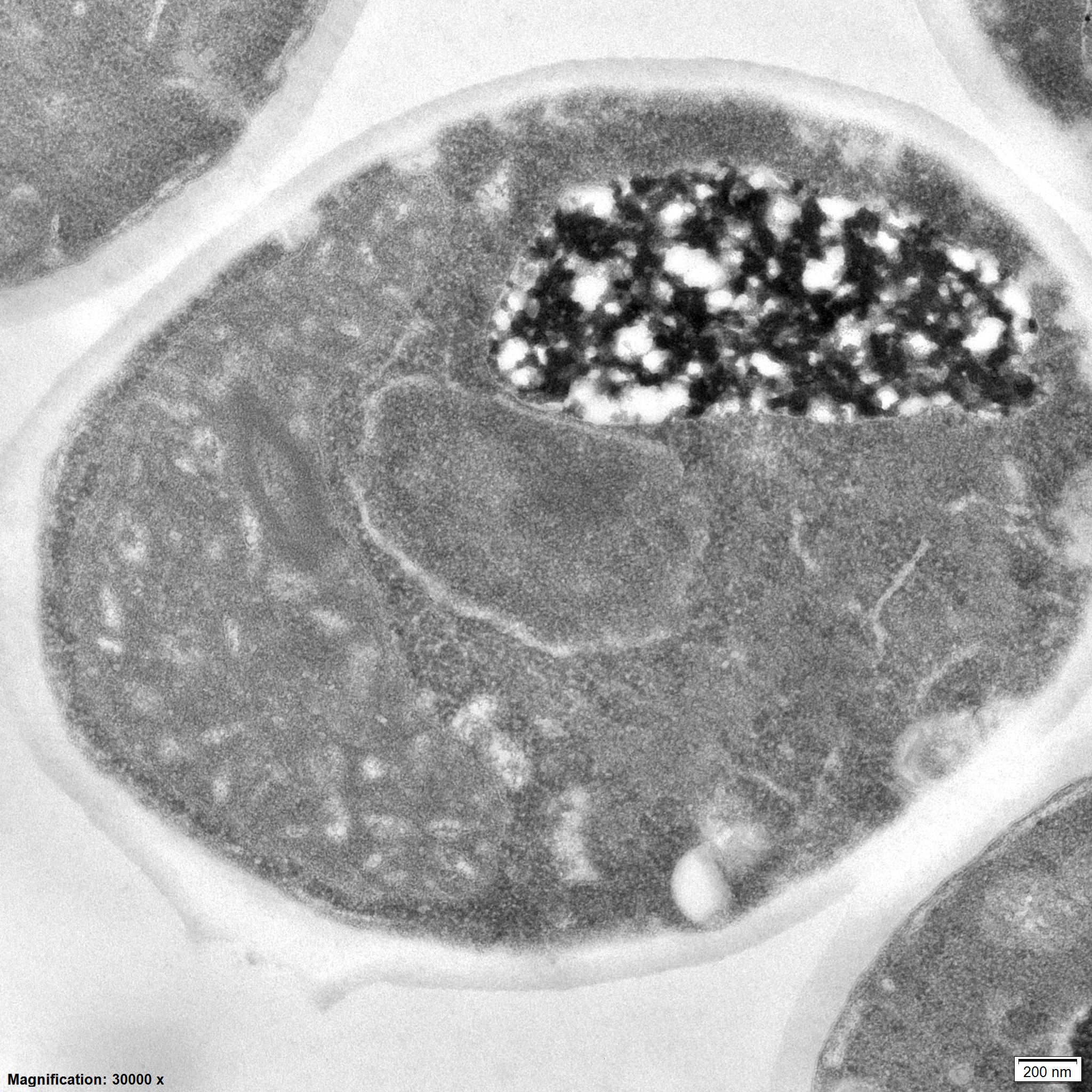

Supplement: Supplementary file 12 — Source data Fig. 4 [file 44319_2024_113_MOESM12_ESM.zip › Koch_SourceData_Fig4/4C/Dtom70 CRISPRi MDM34.tif]

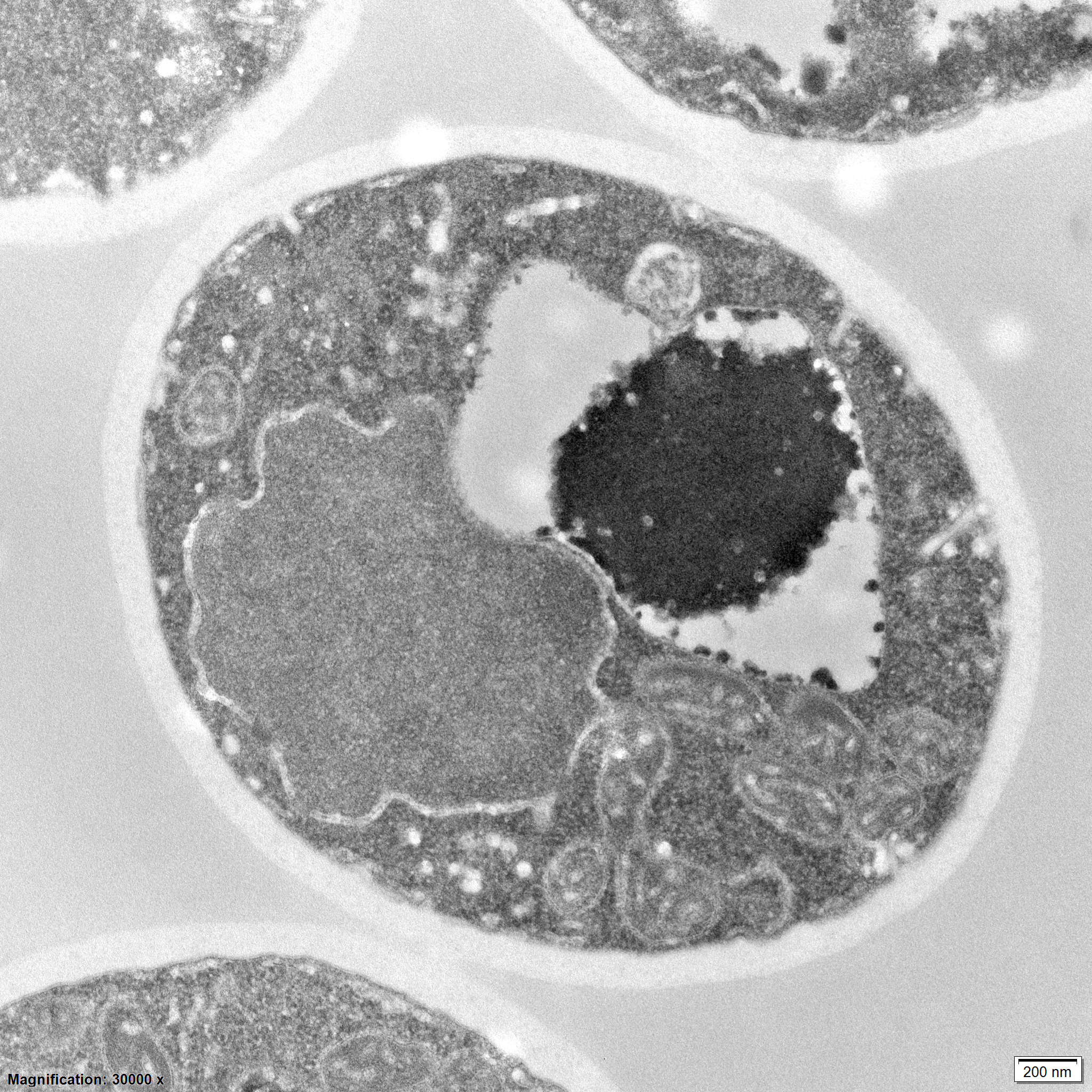

Supplement: Supplementary file 12 — Source data Fig. 4 [file 44319_2024_113_MOESM12_ESM.zip › Koch_SourceData_Fig4/4C/Dtom70.tif]

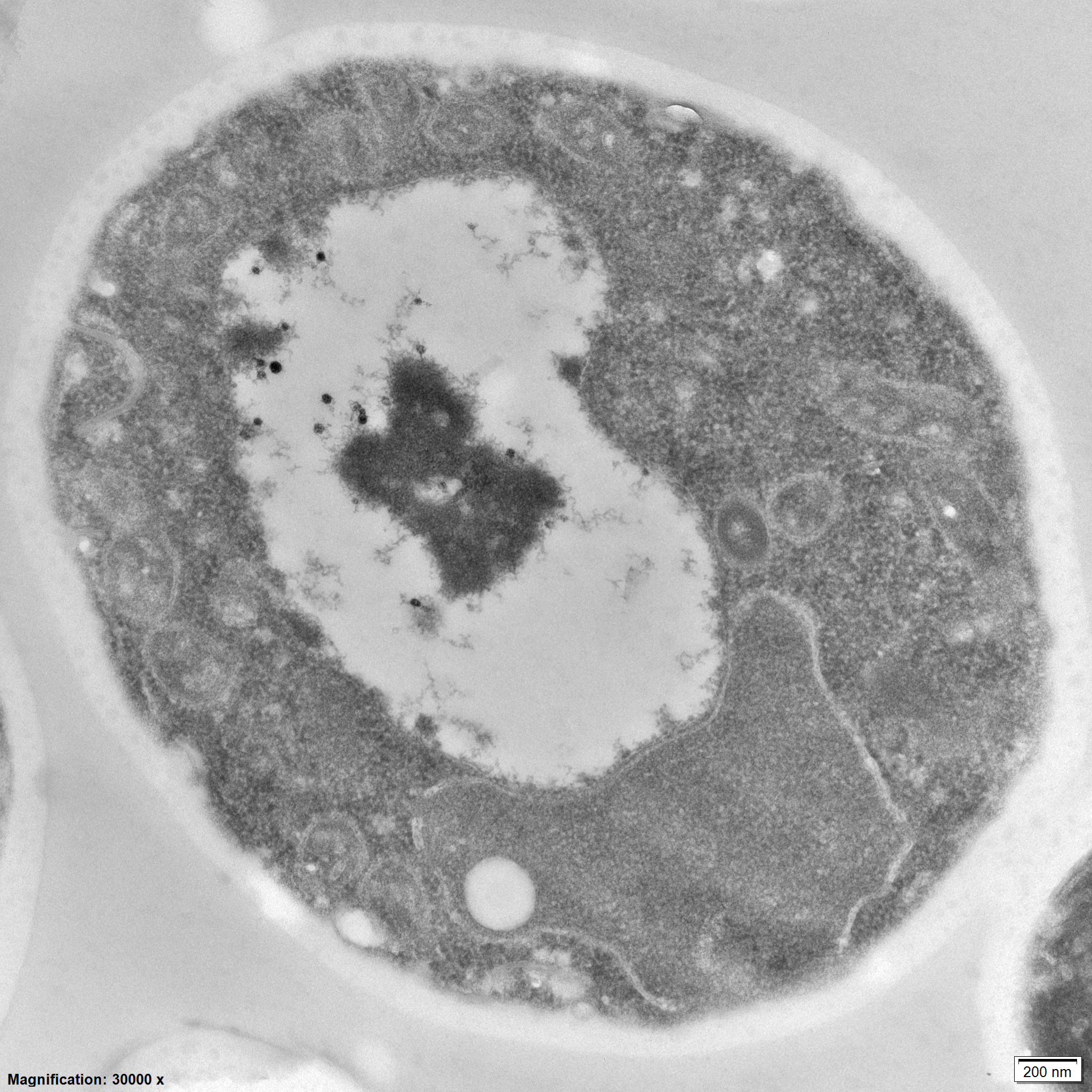

Supplement: Supplementary file 12 — Source data Fig. 4 [file 44319_2024_113_MOESM12_ESM.zip › Koch_SourceData_Fig4/4C/WT.tif]

Figure 6A Cox5A

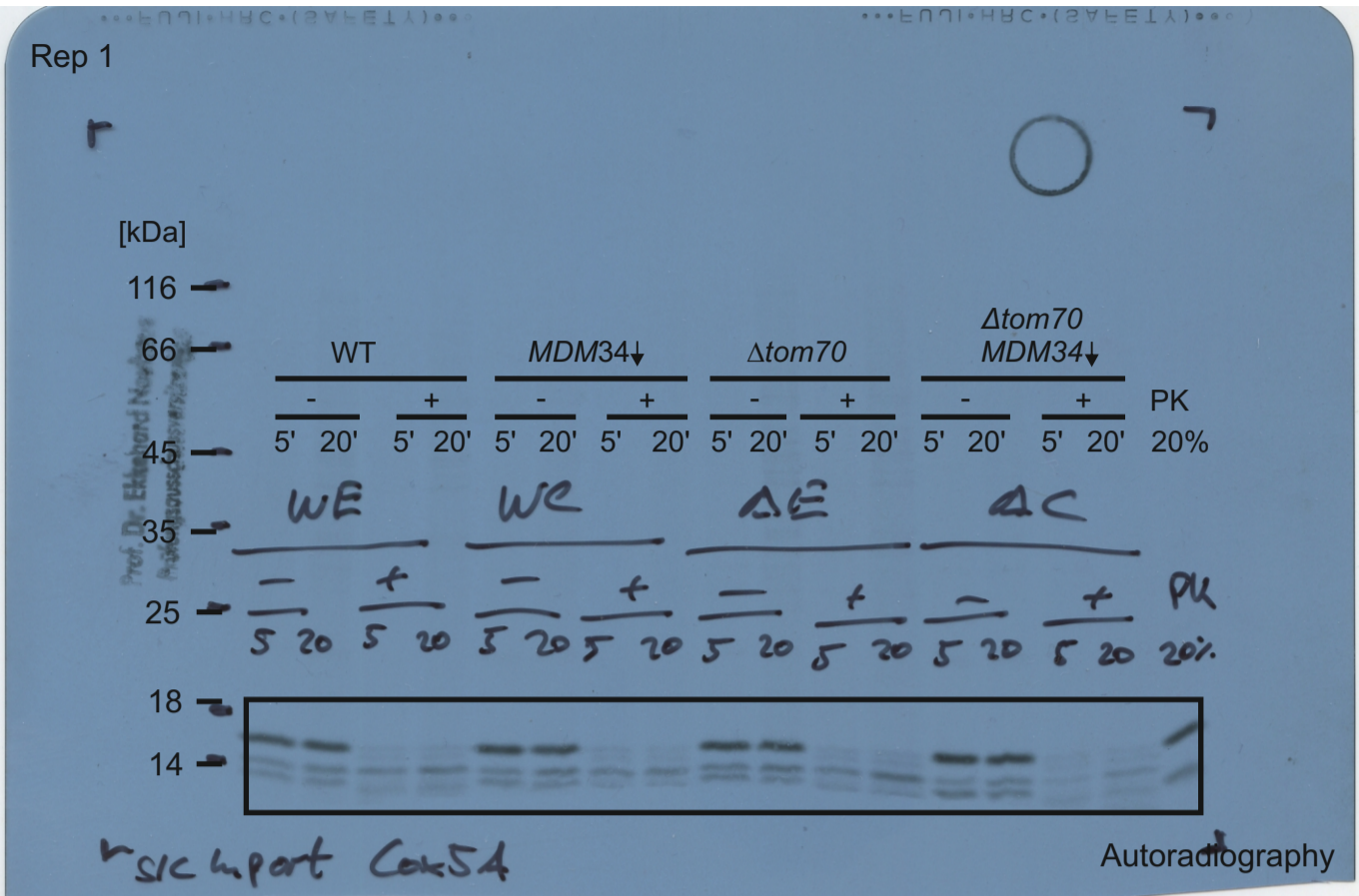

For quantification:

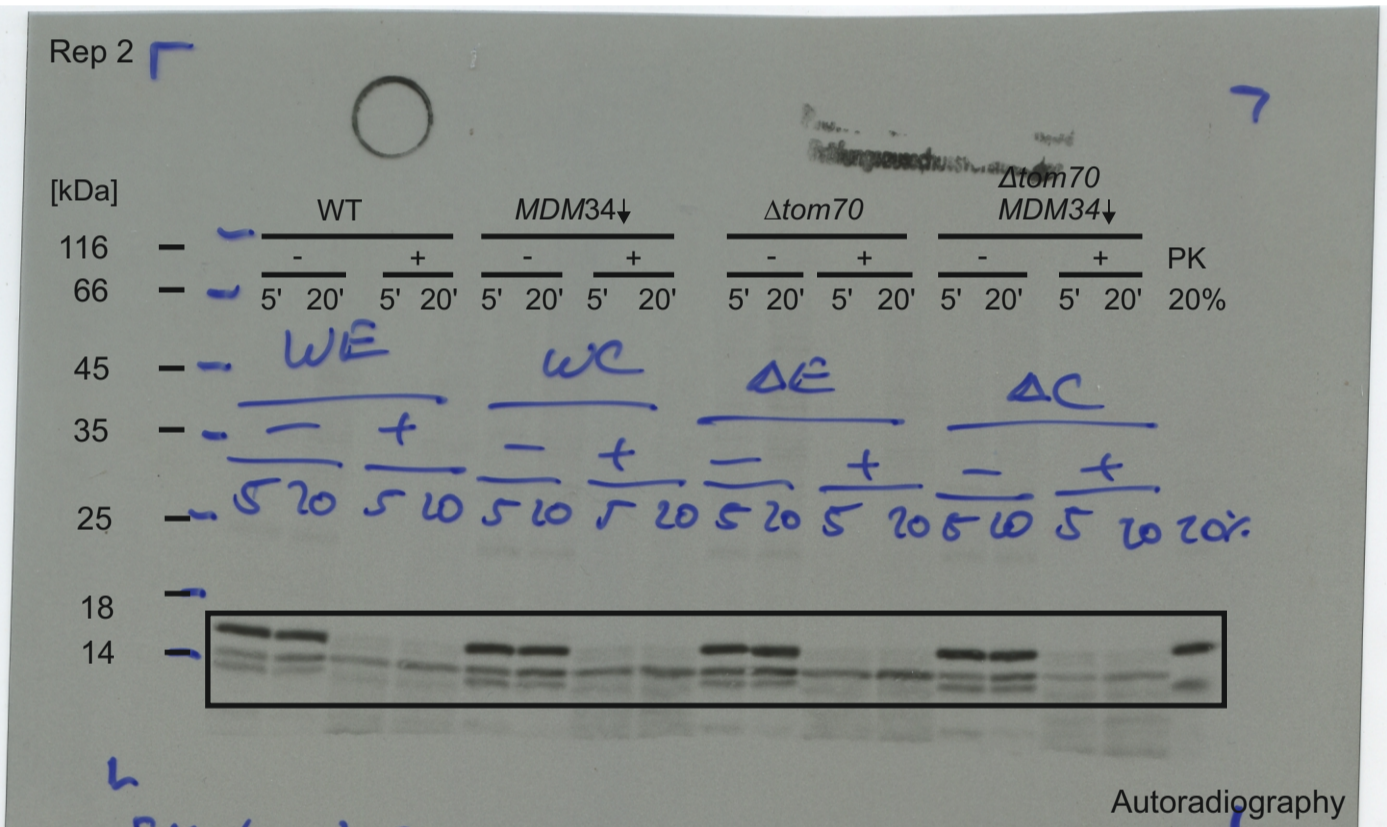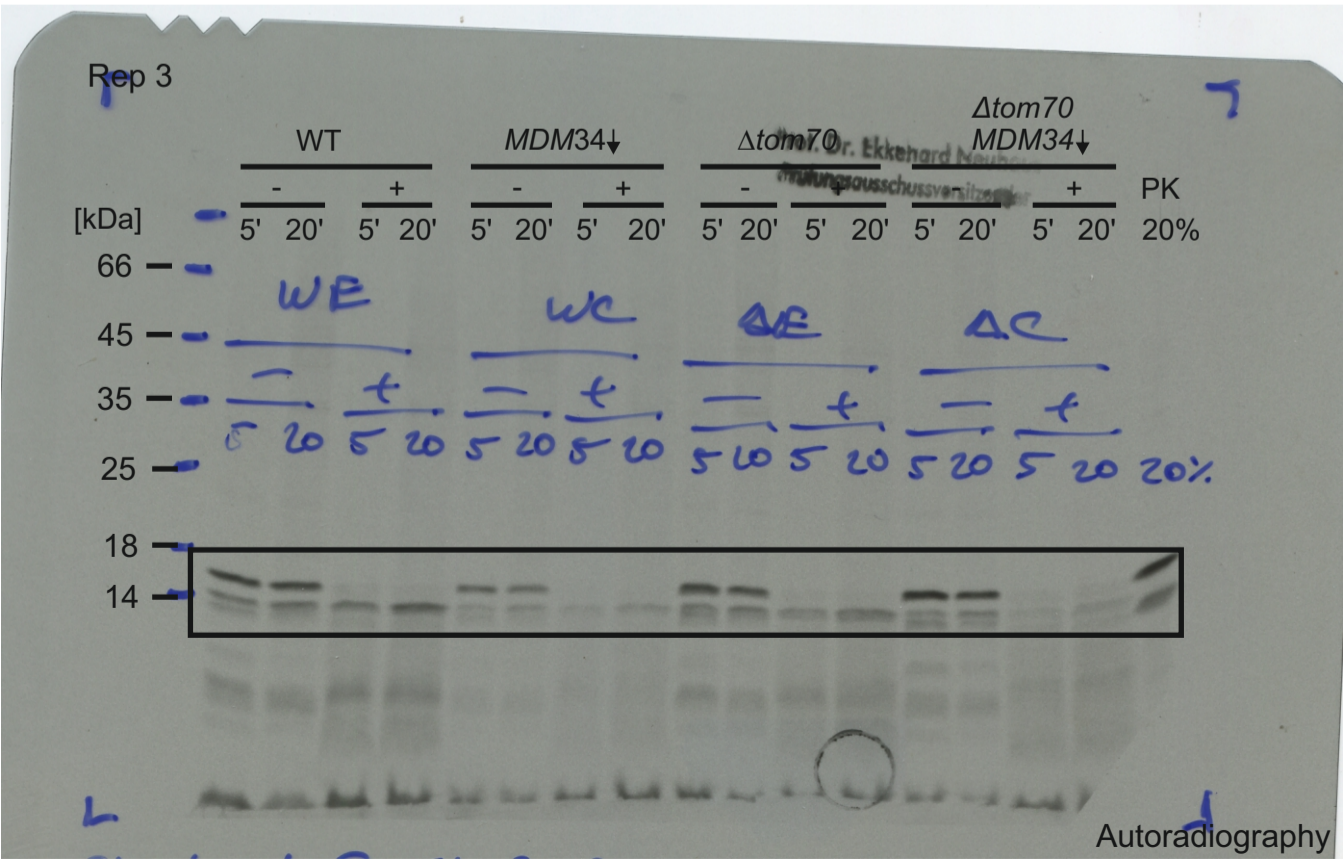

Supplement: Supplementary file 14 — Source data Fig. 6 [file 44319_2024_113_MOESM14_ESM.zip › Koch_SourceData_Fig6/6A-B/6A_Cox5A.pdf]

### Figure 6A Cox5A\_Oxa1TM

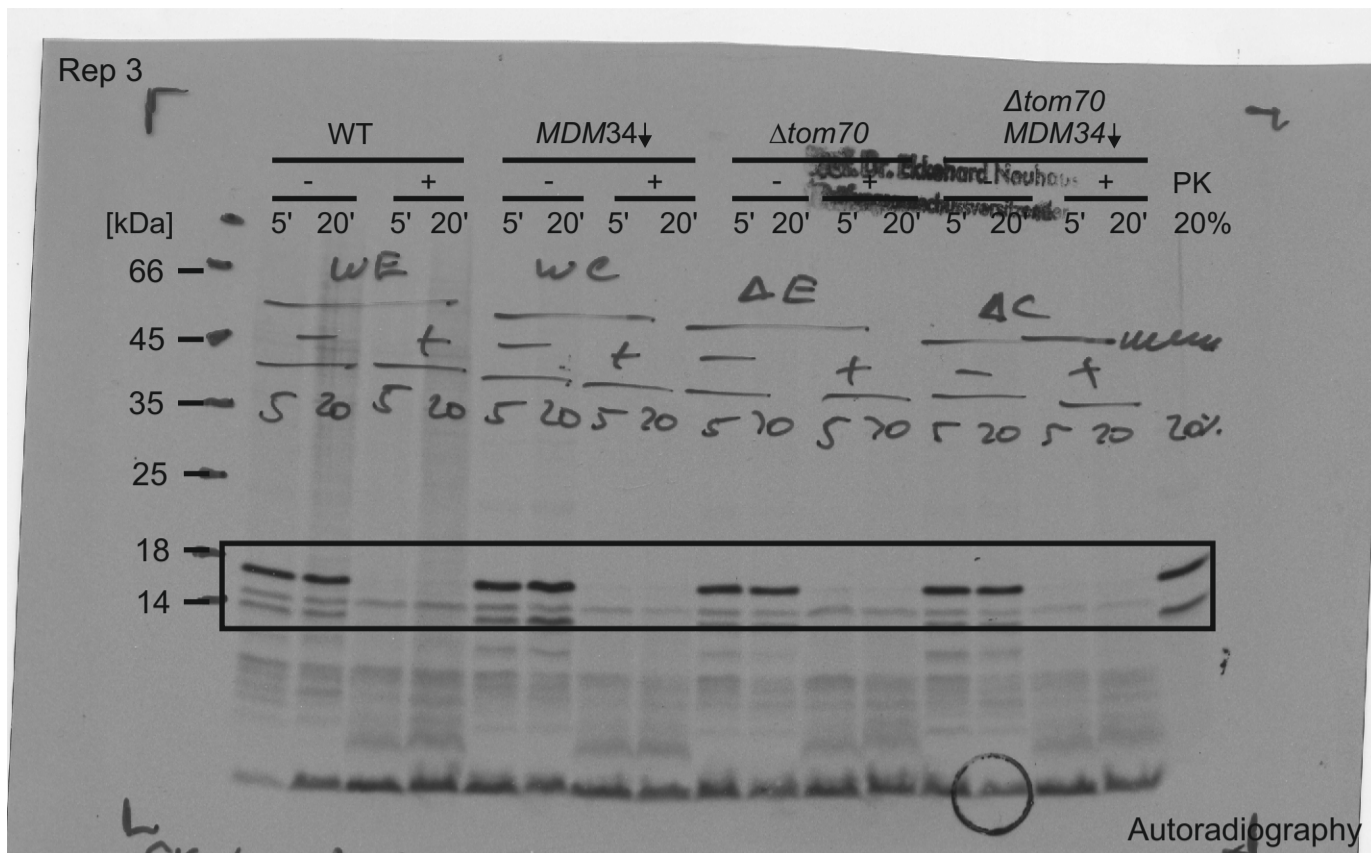

## For quantification:

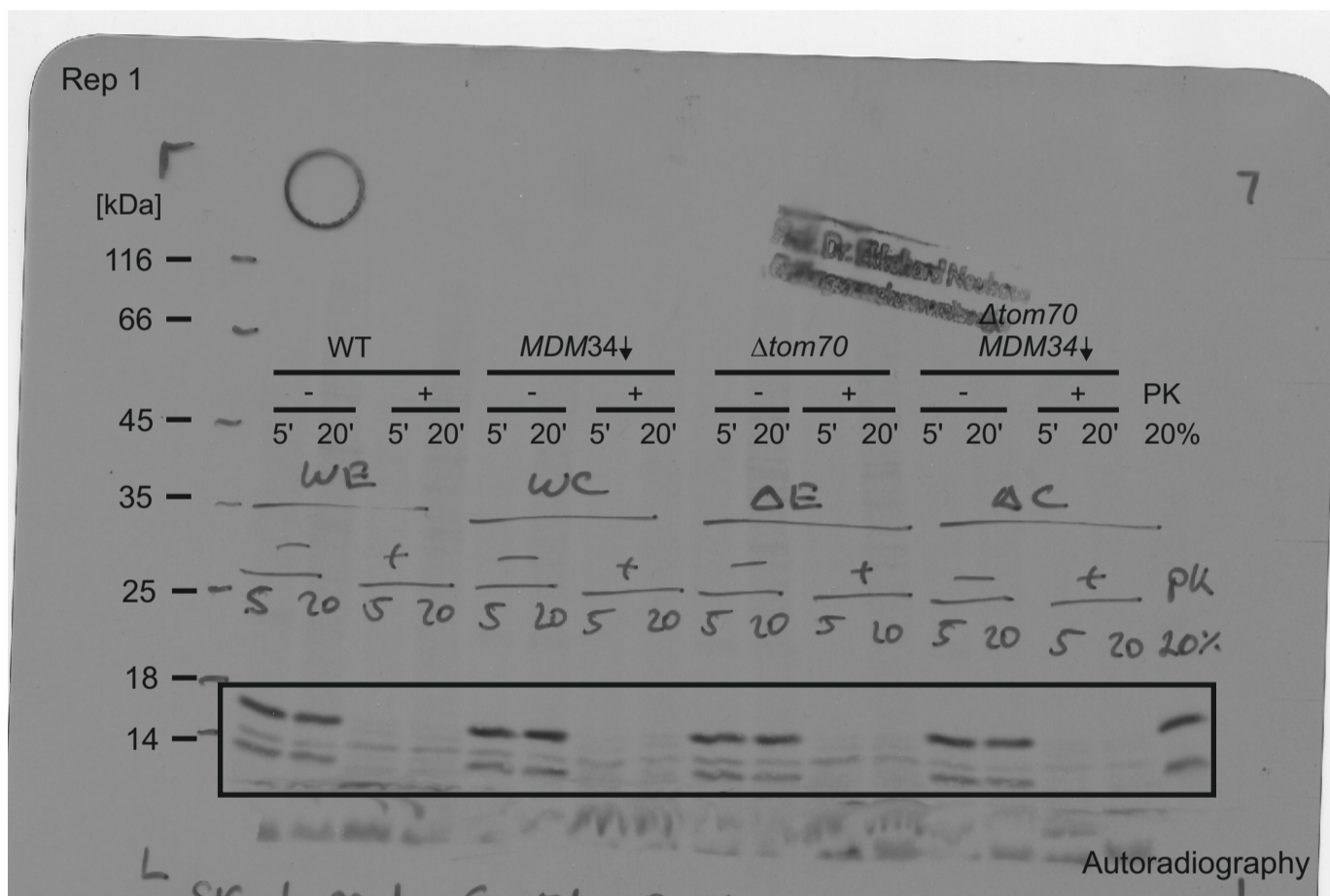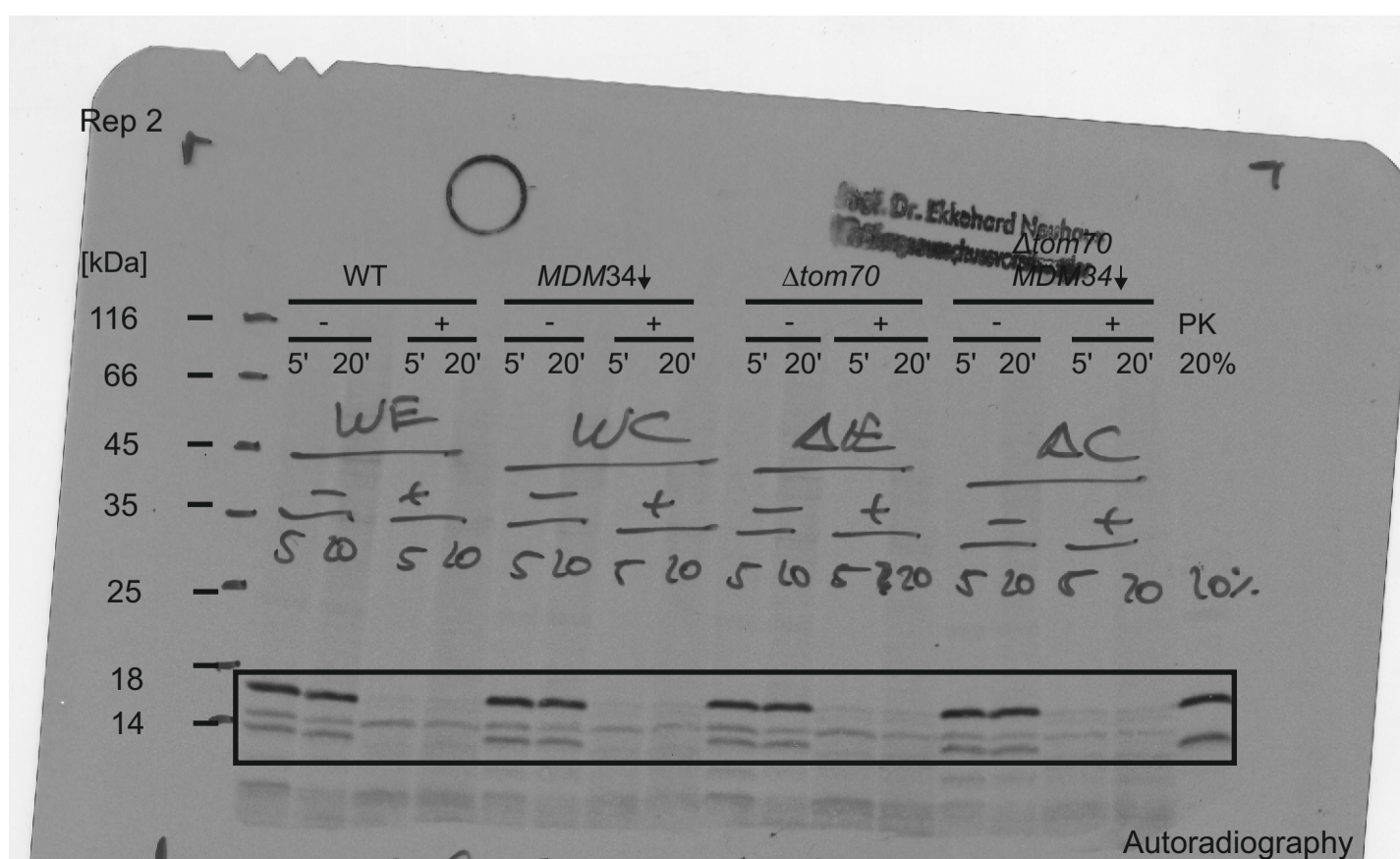

Supplement: Supplementary file 14 — Source data Fig. 6 [file 44319_2024_113_MOESM14_ESM.zip › Koch_SourceData_Fig6/6A-B/6A_Cox5A_Oxa1TM.pdf]

Figure 6A Hsp60

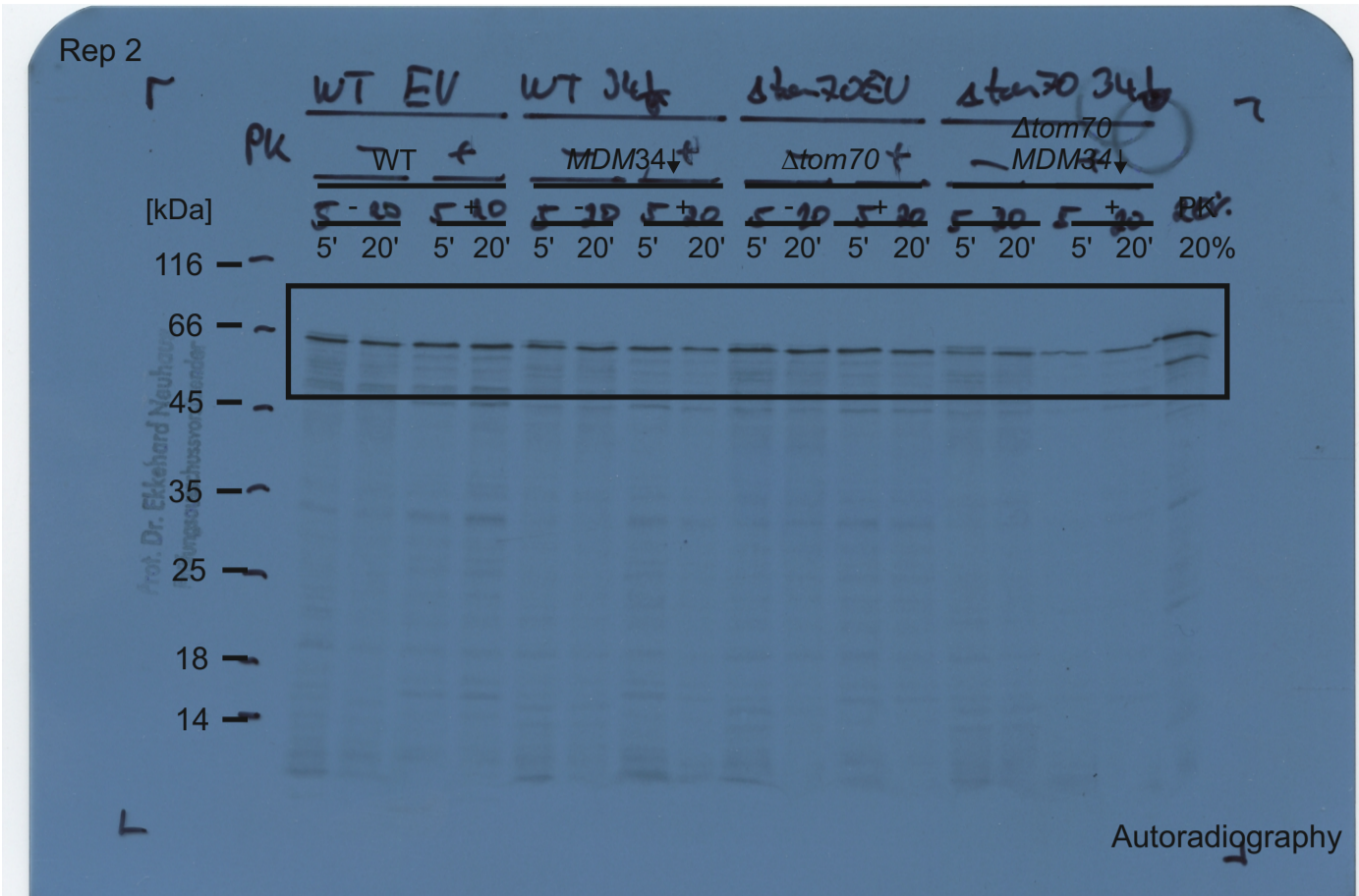

For quantification:

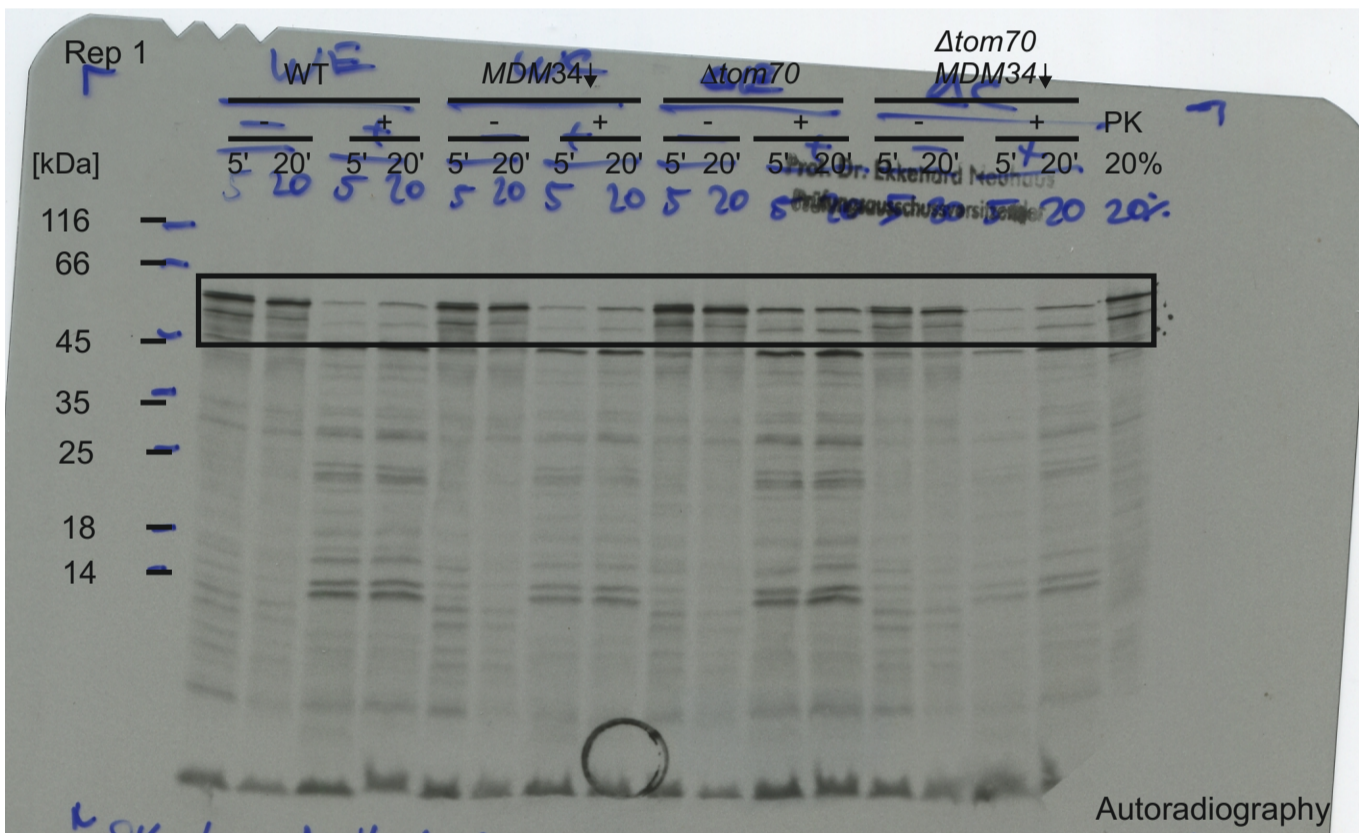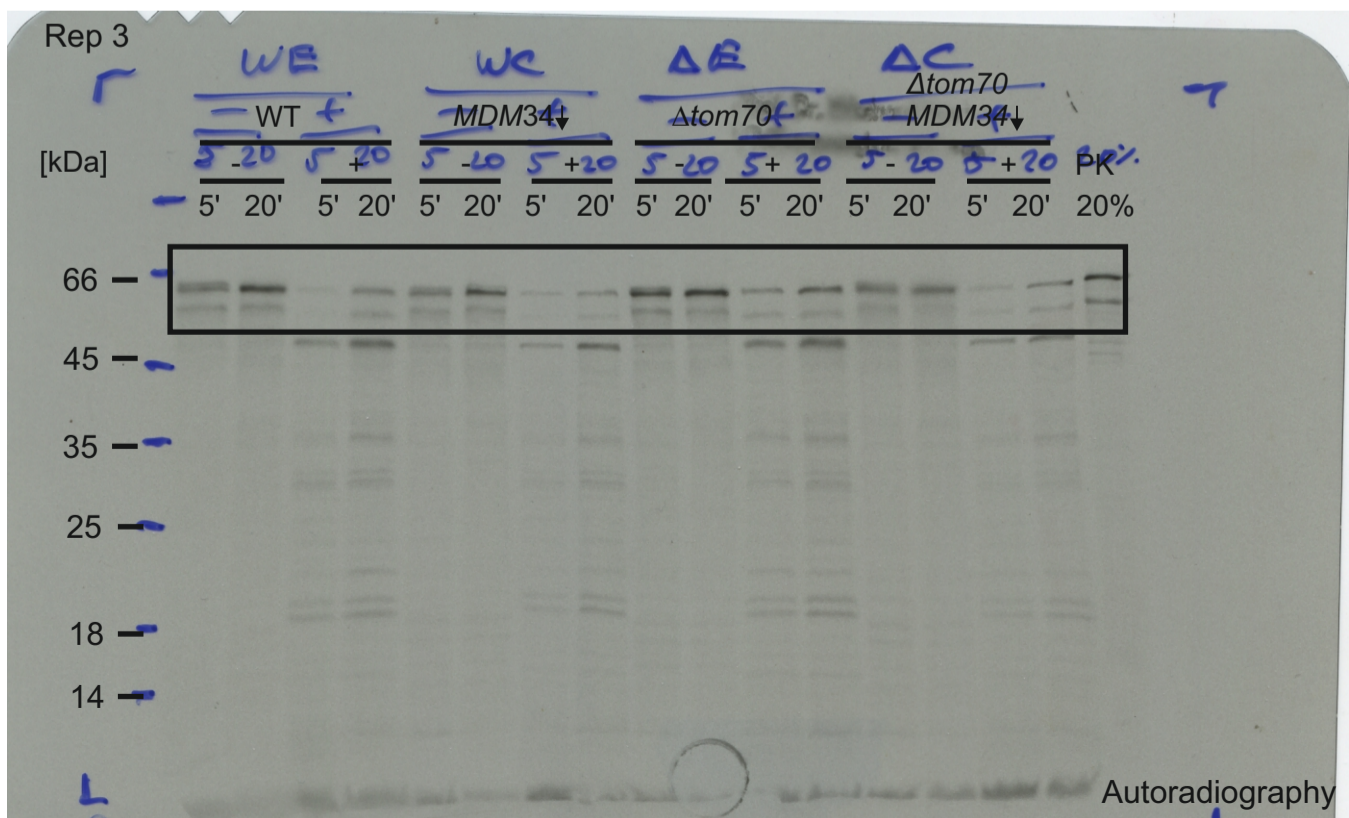

Supplement: Supplementary file 14 — Source data Fig. 6 [file 44319_2024_113_MOESM14_ESM.zip › Koch_SourceData_Fig6/6A-B/6A_Hsp60.pdf]

Figure 6A Oxa1

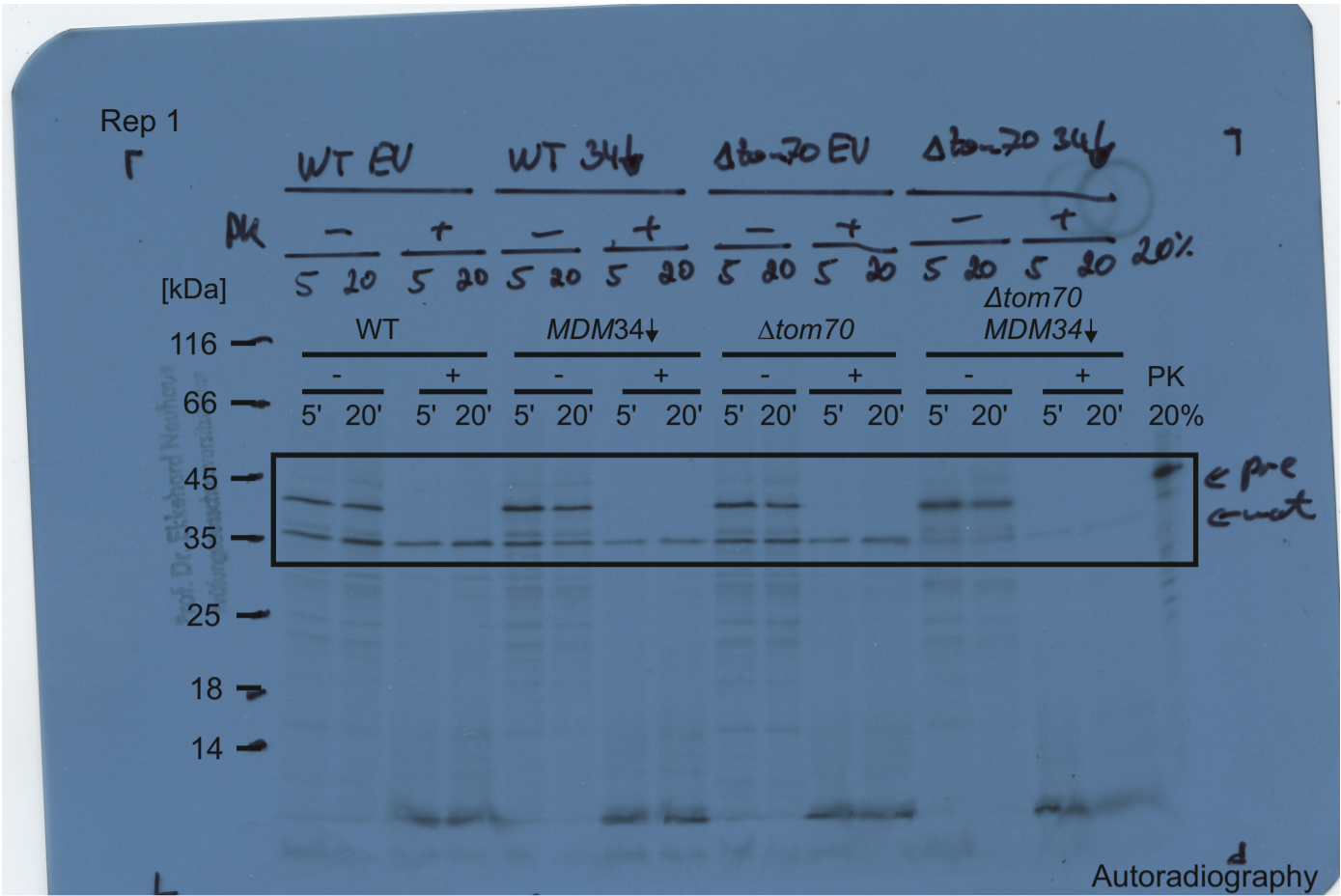

For quantification:

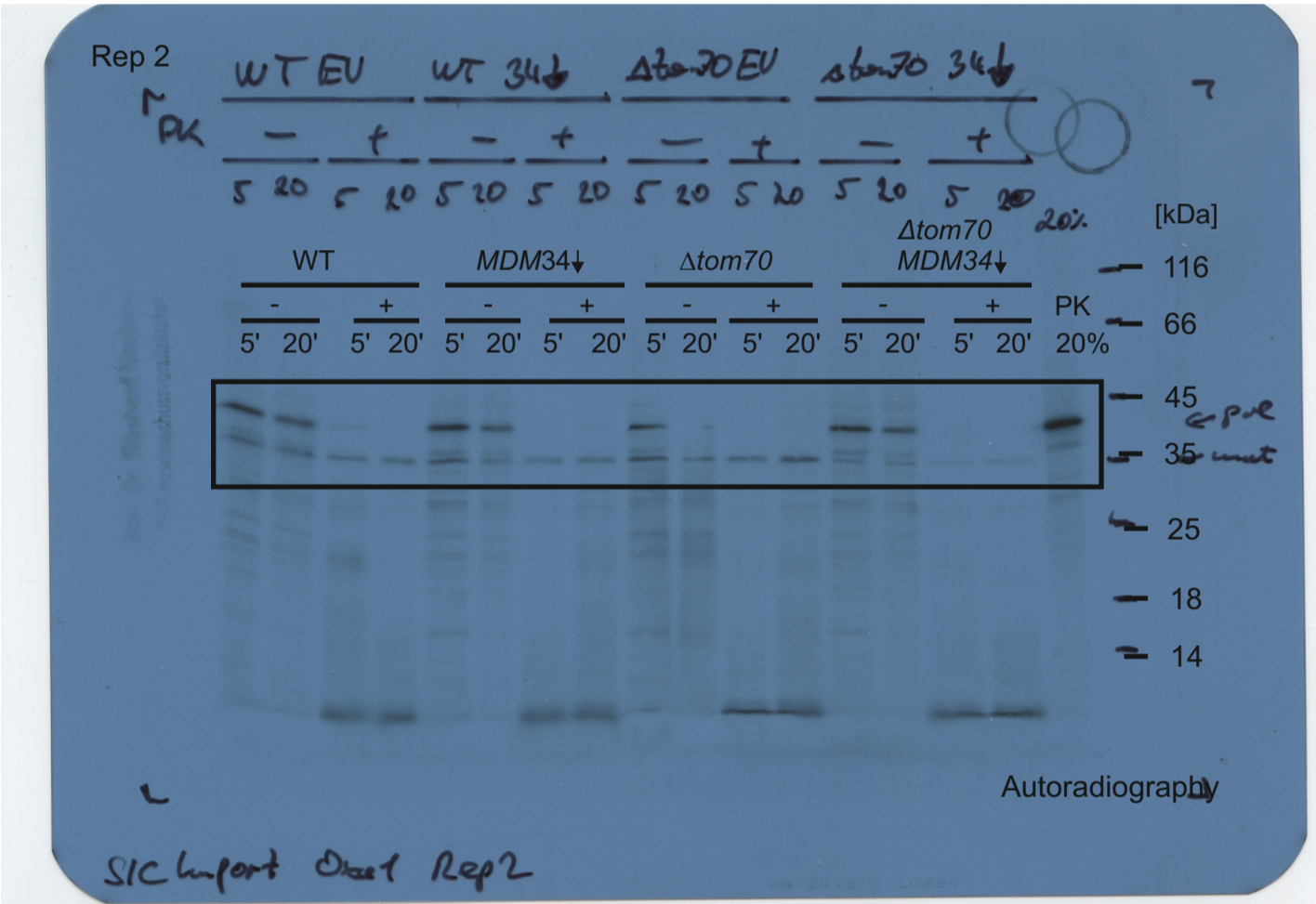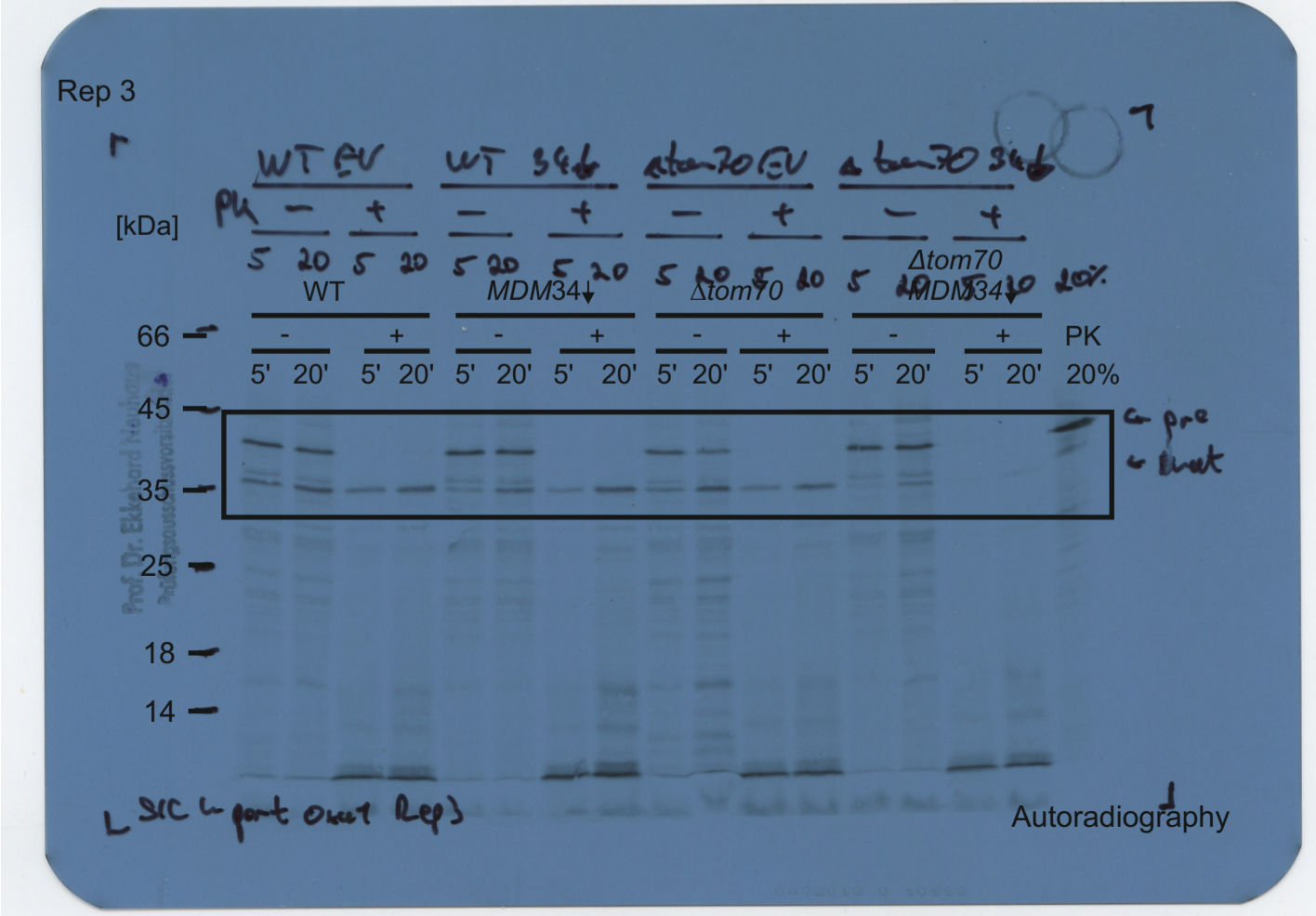

Supplement: Supplementary file 14 — Source data Fig. 6 [file 44319_2024_113_MOESM14_ESM.zip › Koch_SourceData_Fig6/6A-B/6A_Oxa1.pdf]

Figure 6C

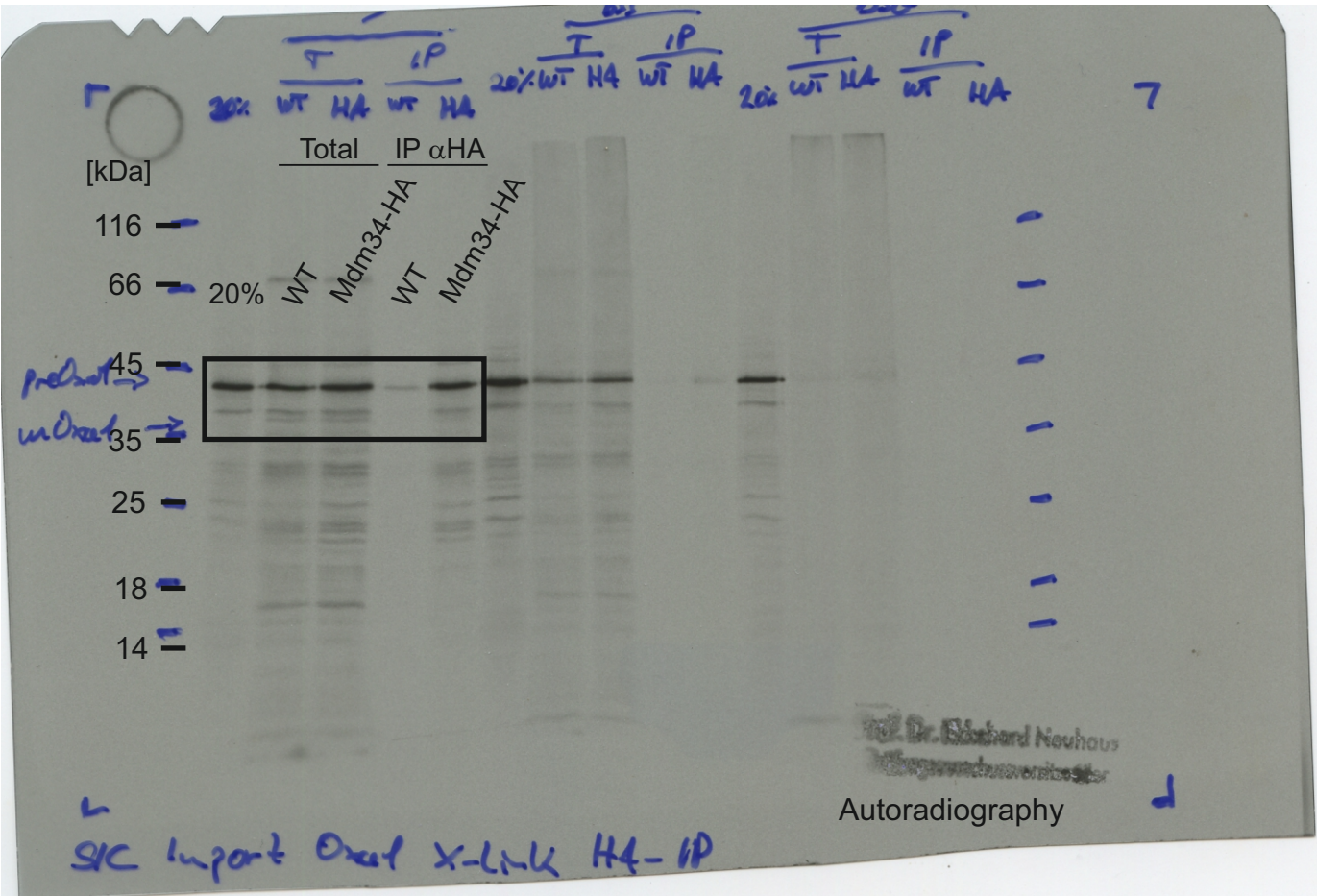

Supplement: Supplementary file 14 — Source data Fig. 6 [file 44319_2024_113_MOESM14_ESM.zip › Koch_SourceData_Fig6/6C/6C.pdf]

Figure 6C

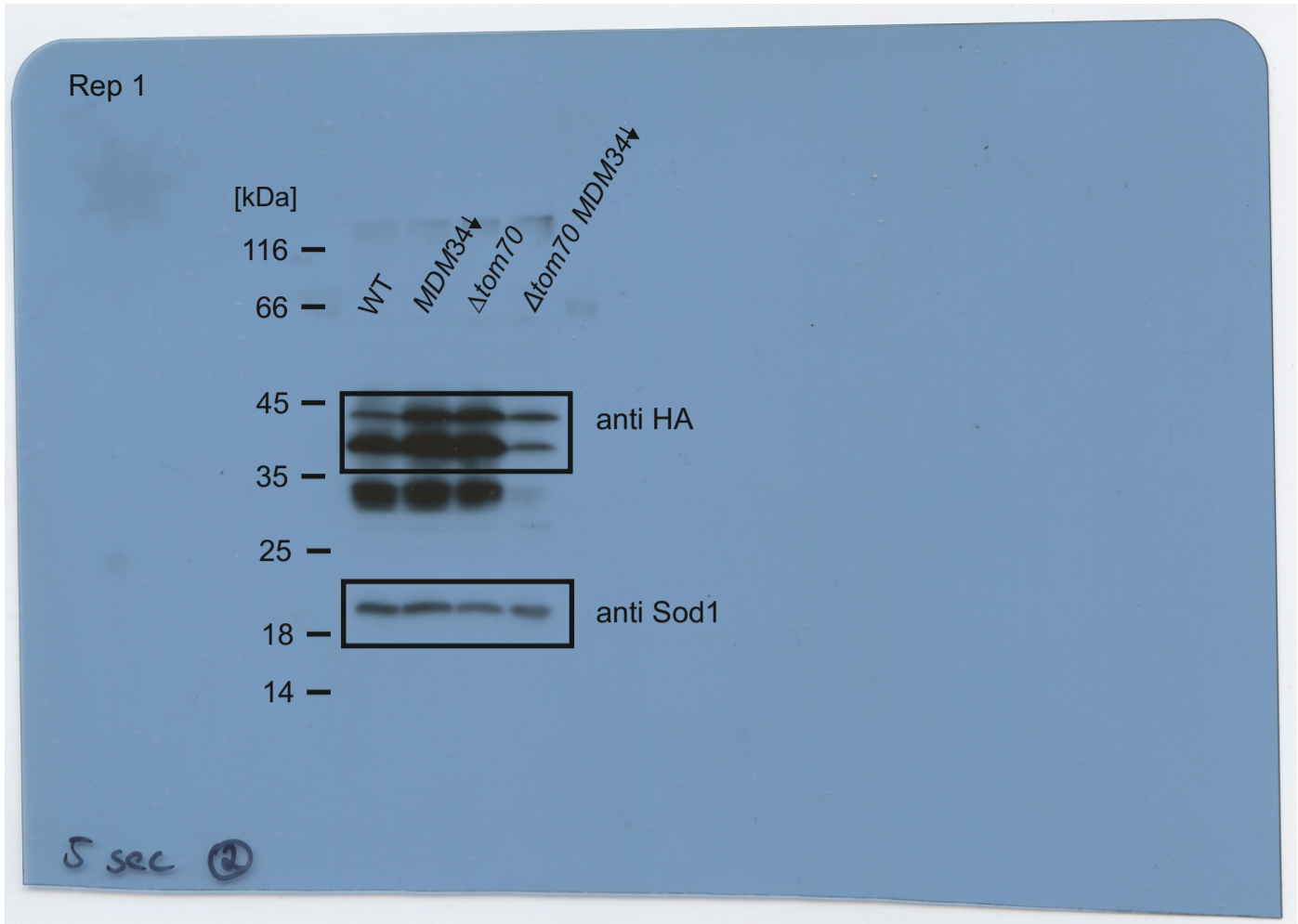

For quantification:

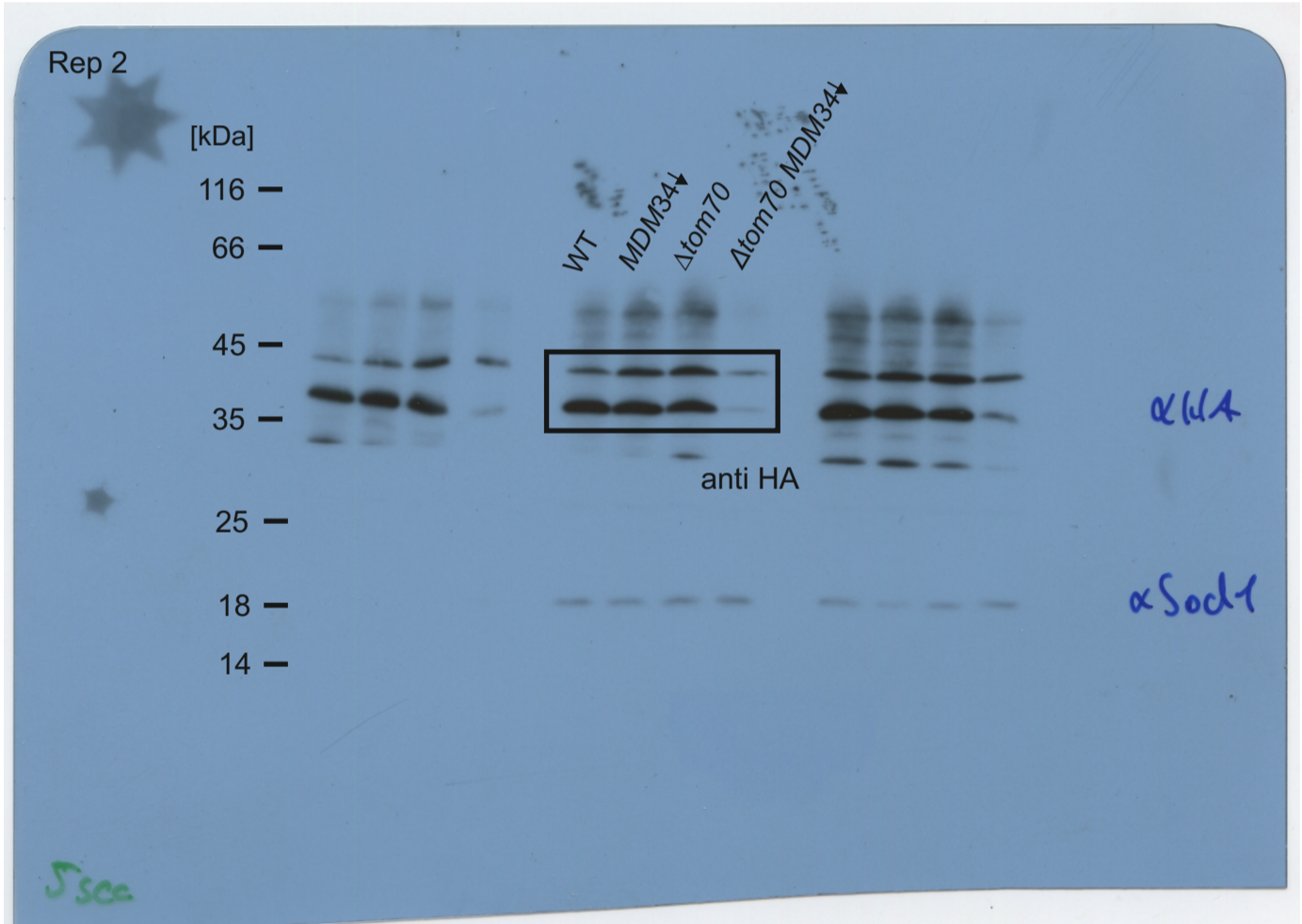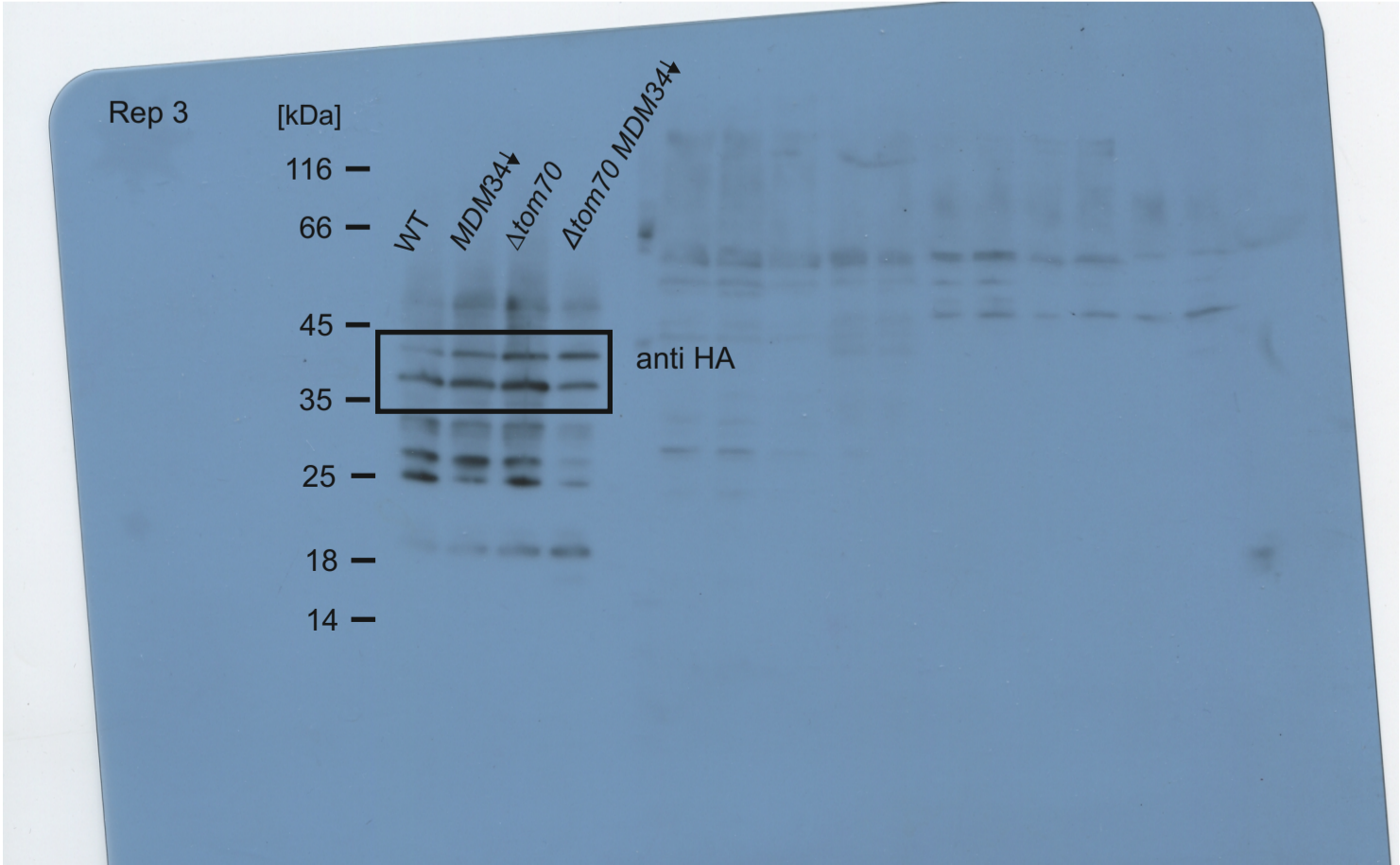

Supplement: Supplementary file 14 — Source data Fig. 6 [file 44319_2024_113_MOESM14_ESM.zip › Koch_SourceData_Fig6/6D-E/6D.pdf]

Appendix Figure 2A

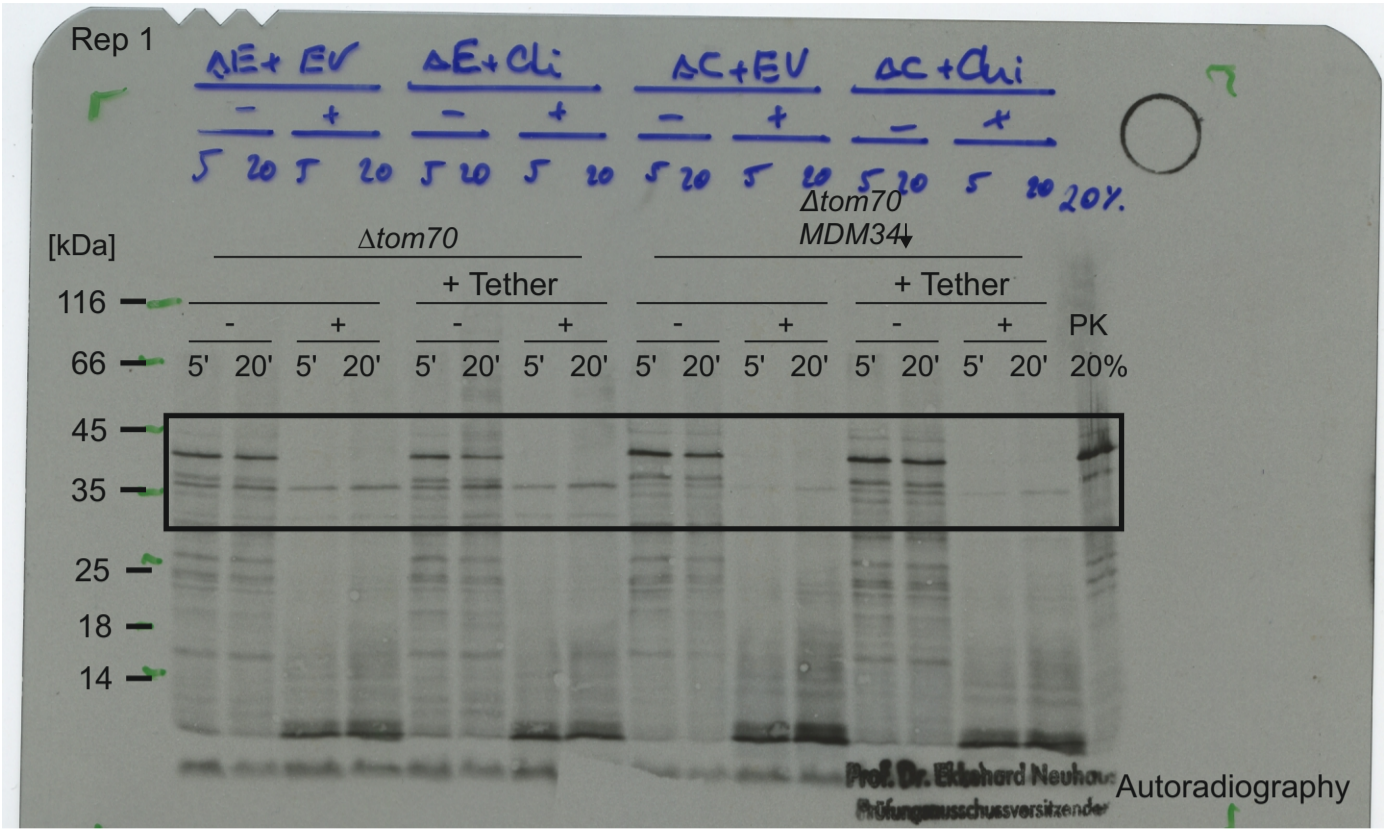

For quantification:

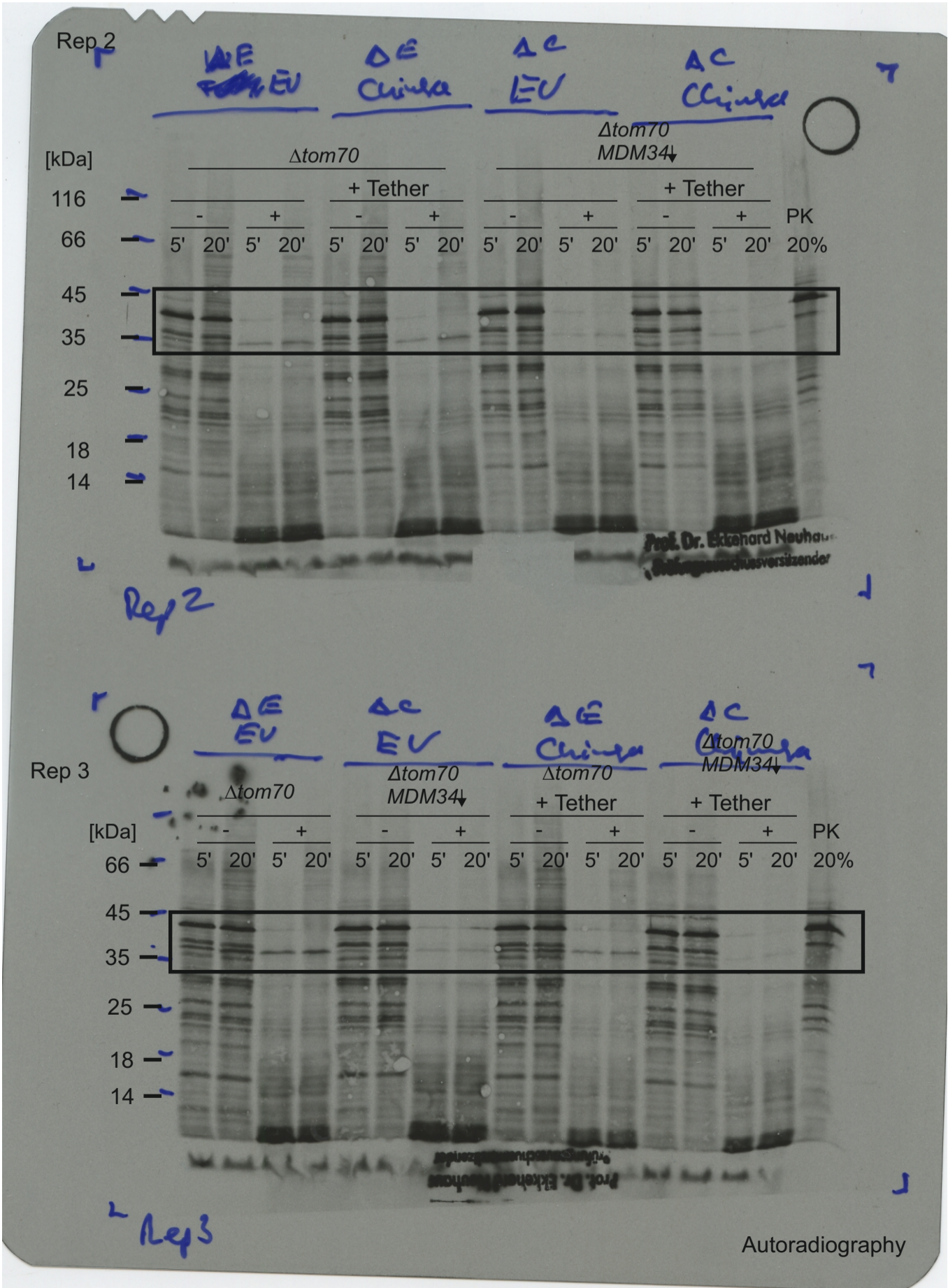

Supplement: Supplementary file 15 — Figure EV1-5 and Appendix S2 Source Data [file 44319_2024_113_MOESM15_ESM.zip › Koch_SourceData_AppendixFigall/Appendix_2A.pdf]

Appendix Figure 2C

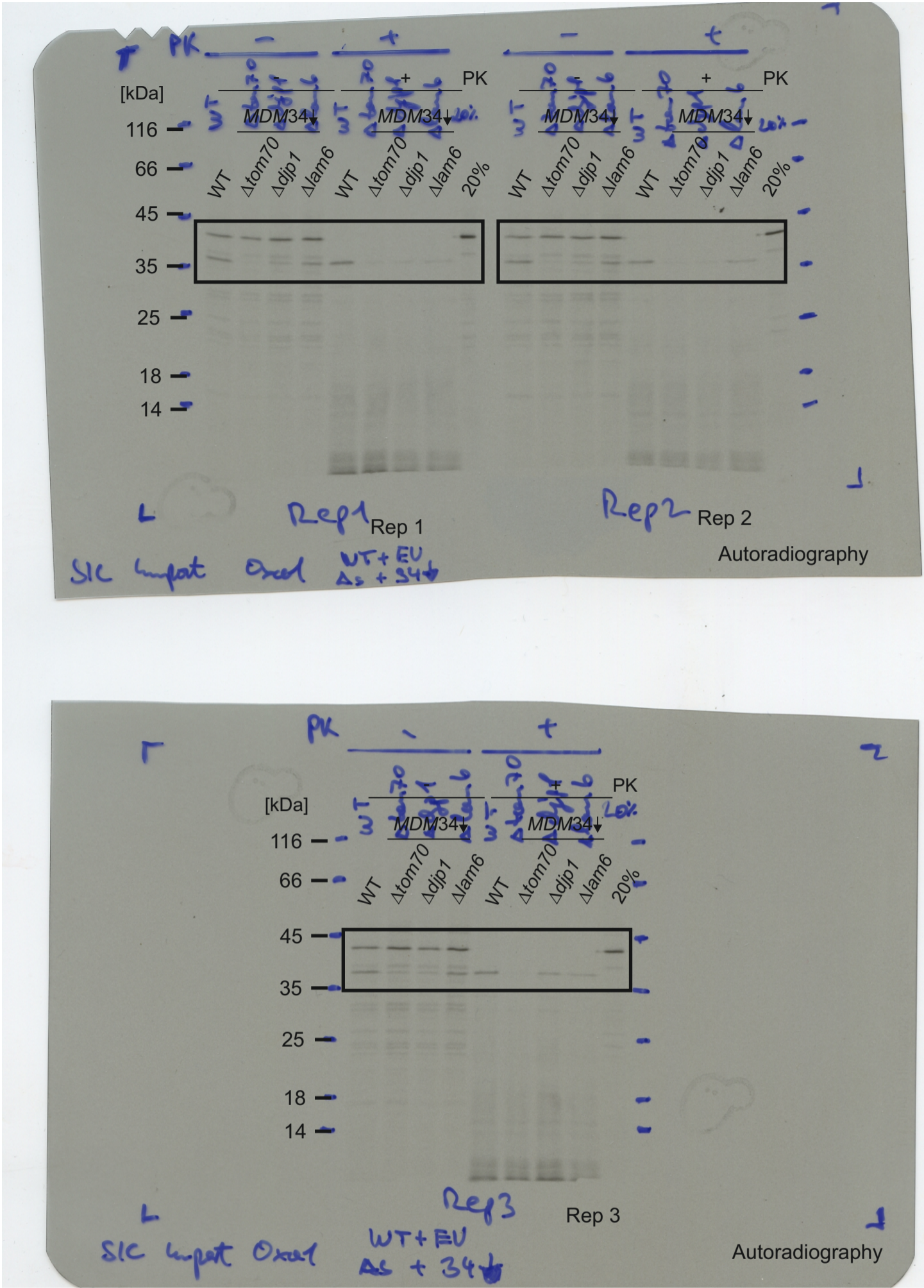

Rep 2 was shown in the Figure, Rep 1 and 3 were used for quantification

Supplement: Supplementary file 15 — Figure EV1-5 and Appendix S2 Source Data [file 44319_2024_113_MOESM15_ESM.zip › Koch_SourceData_AppendixFigall/Appendix_2C.pdf]

Appendix Figure 2E

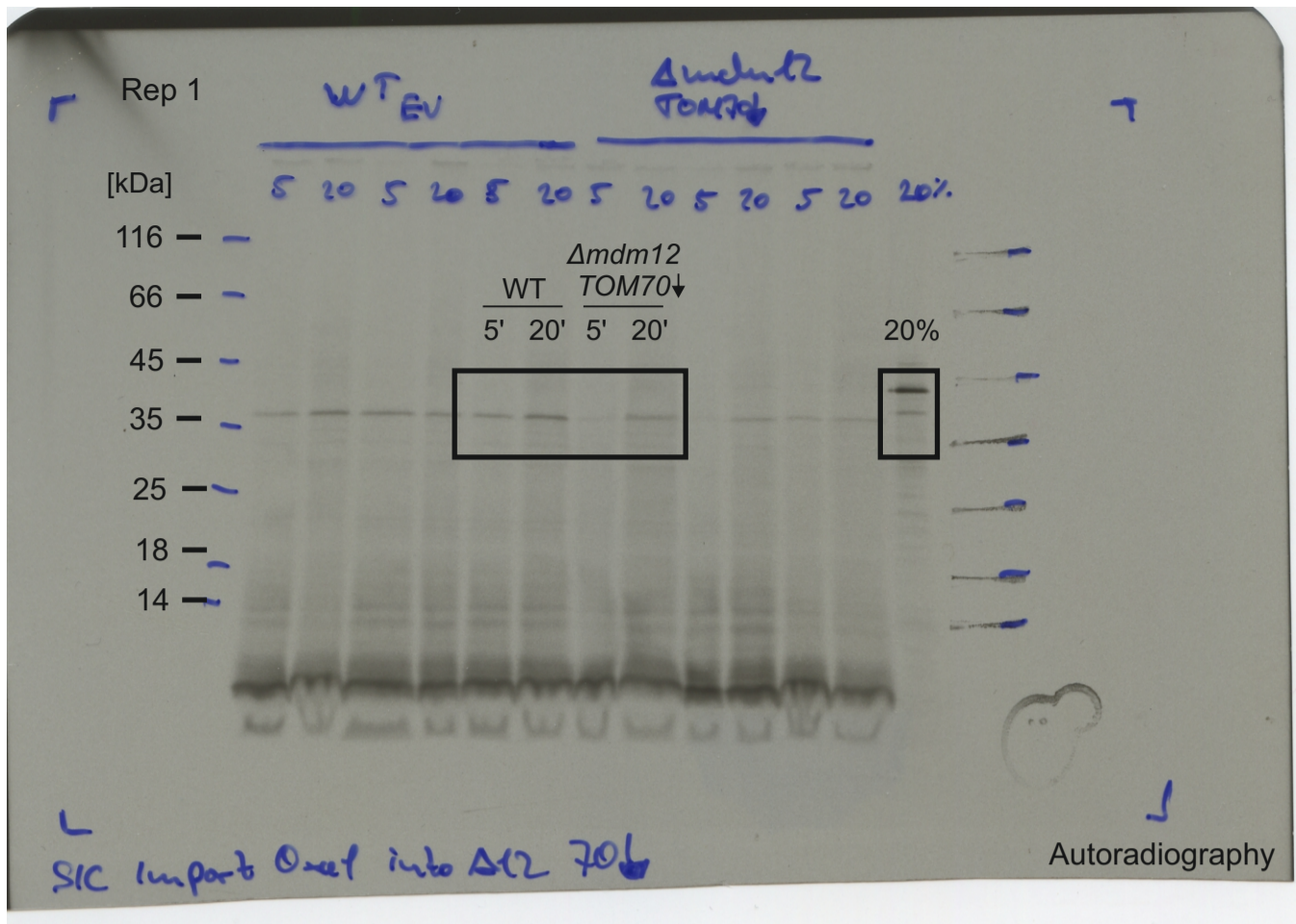

For quantification:

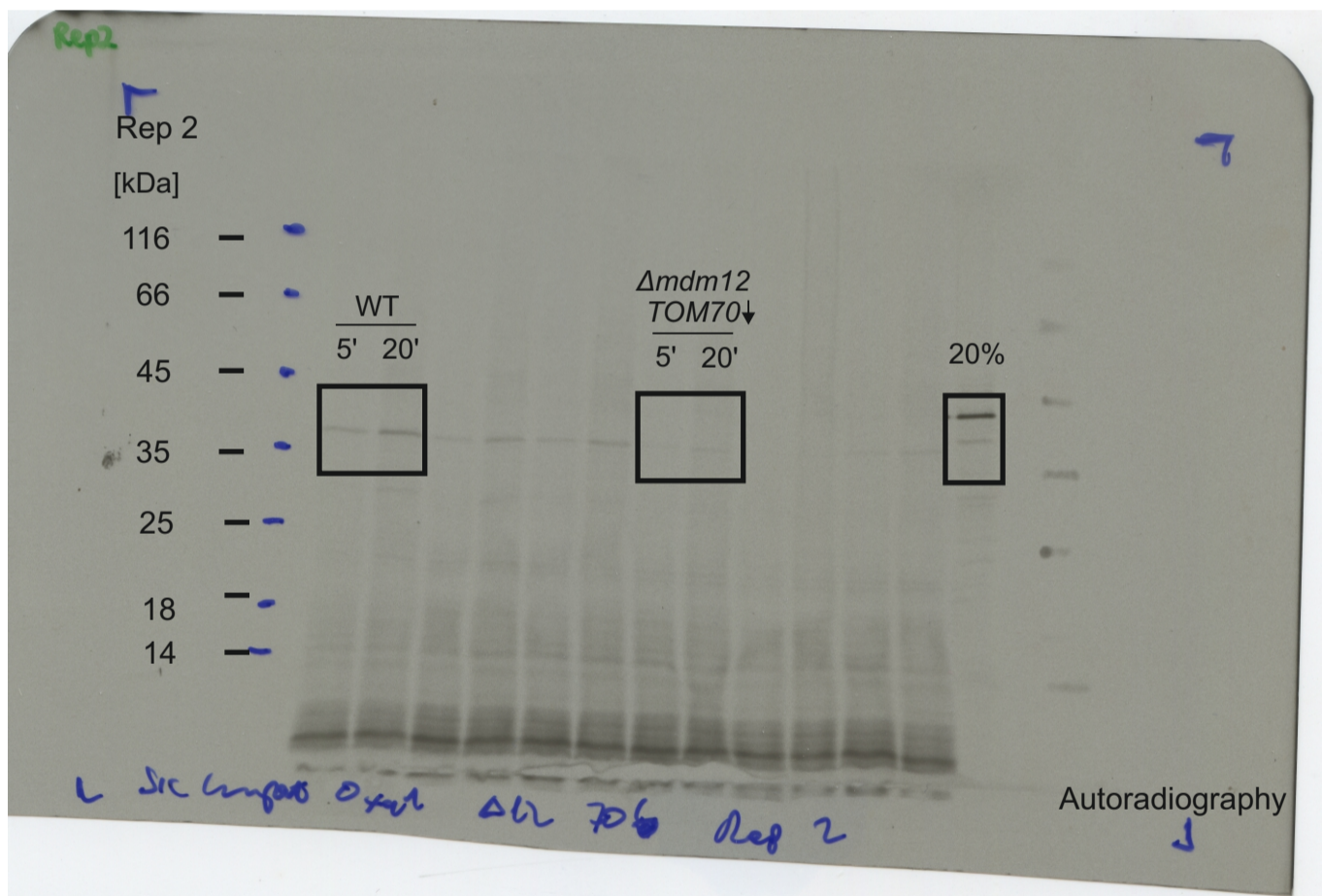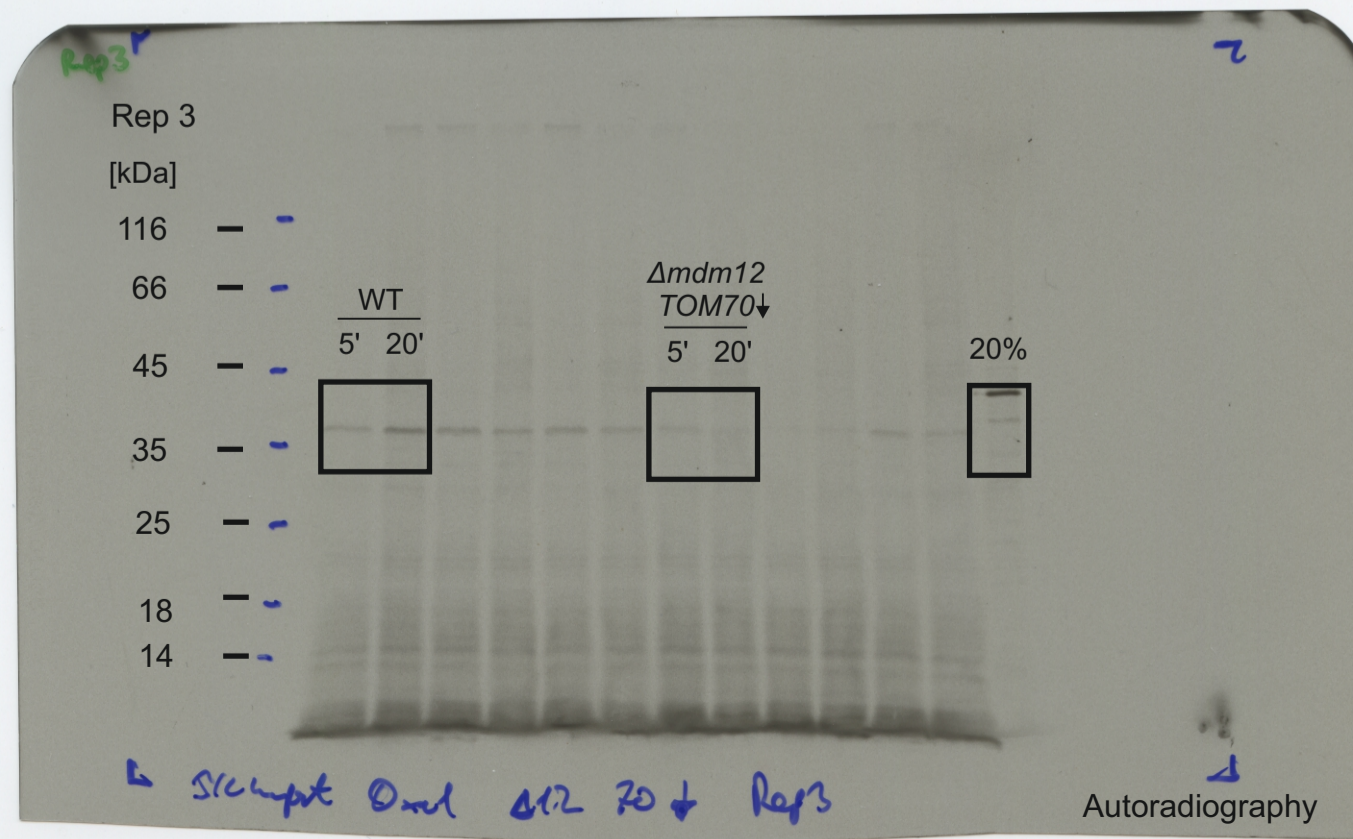

Supplement: Supplementary file 15 — Figure EV1-5 and Appendix S2 Source Data [file 44319_2024_113_MOESM15_ESM.zip › Koch_SourceData_AppendixFigall/Appendix_2E.pdf]

# Appendix Figure 2G

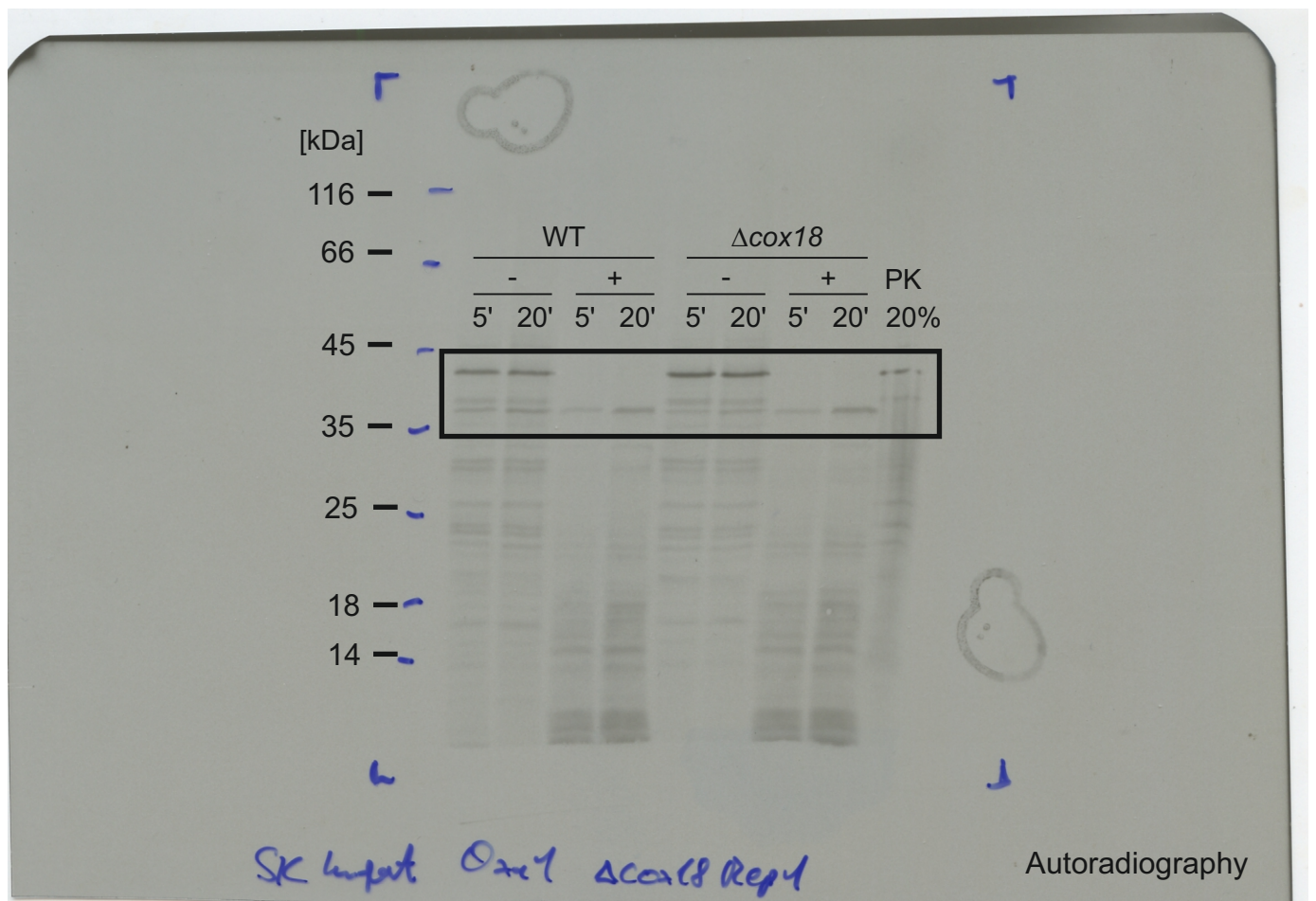

Supplement: Supplementary file 15 — Figure EV1-5 and Appendix S2 Source Data [file 44319_2024_113_MOESM15_ESM.zip › Koch_SourceData_AppendixFigall/Appendix_2G.pdf]

# Appendix Figure 2H

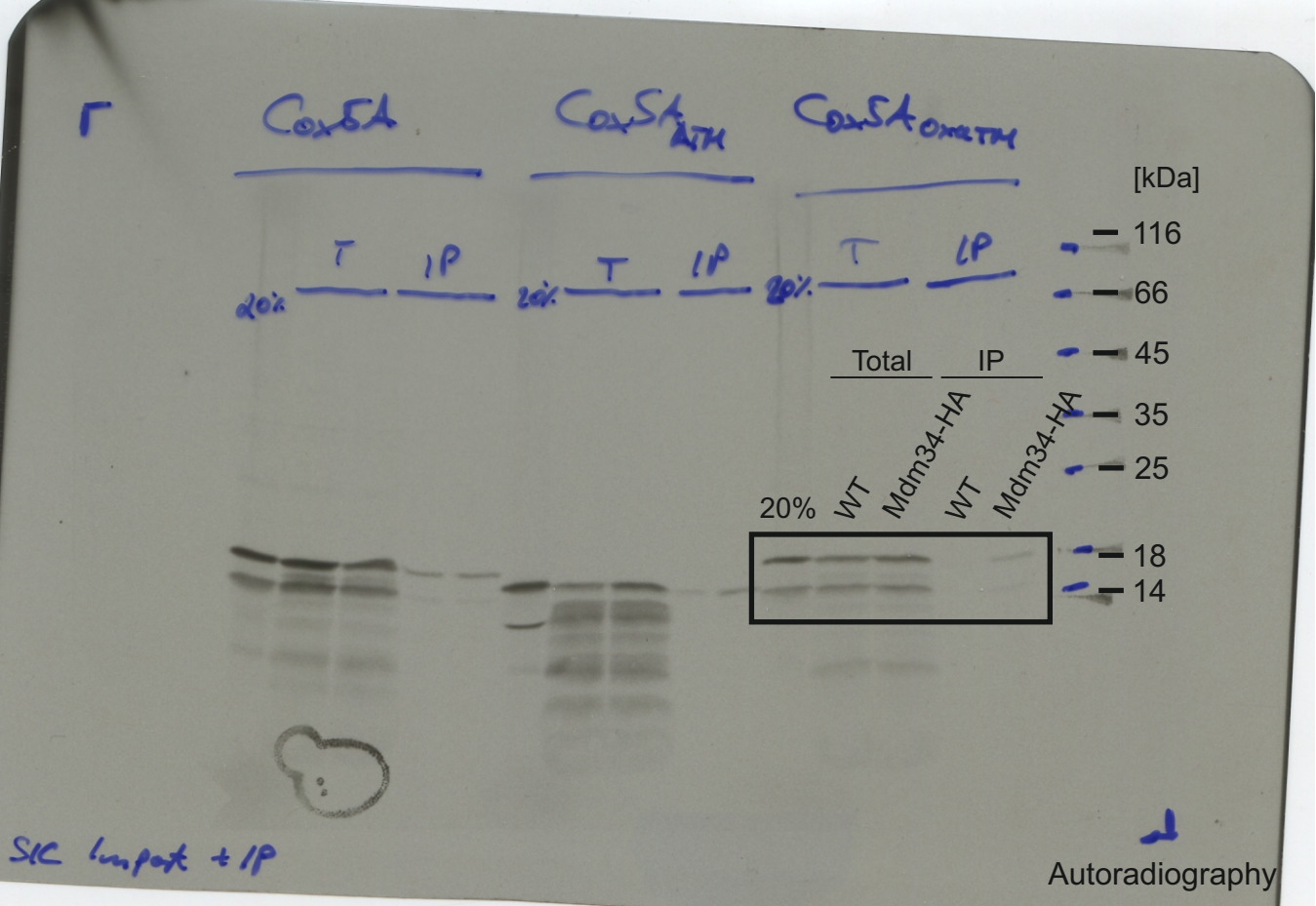

Supplement: Supplementary file 15 — Figure EV1-5 and Appendix S2 Source Data [file 44319_2024_113_MOESM15_ESM.zip › Koch_SourceData_AppendixFigall/Appendix_2H.pdf]

Figure EV1A

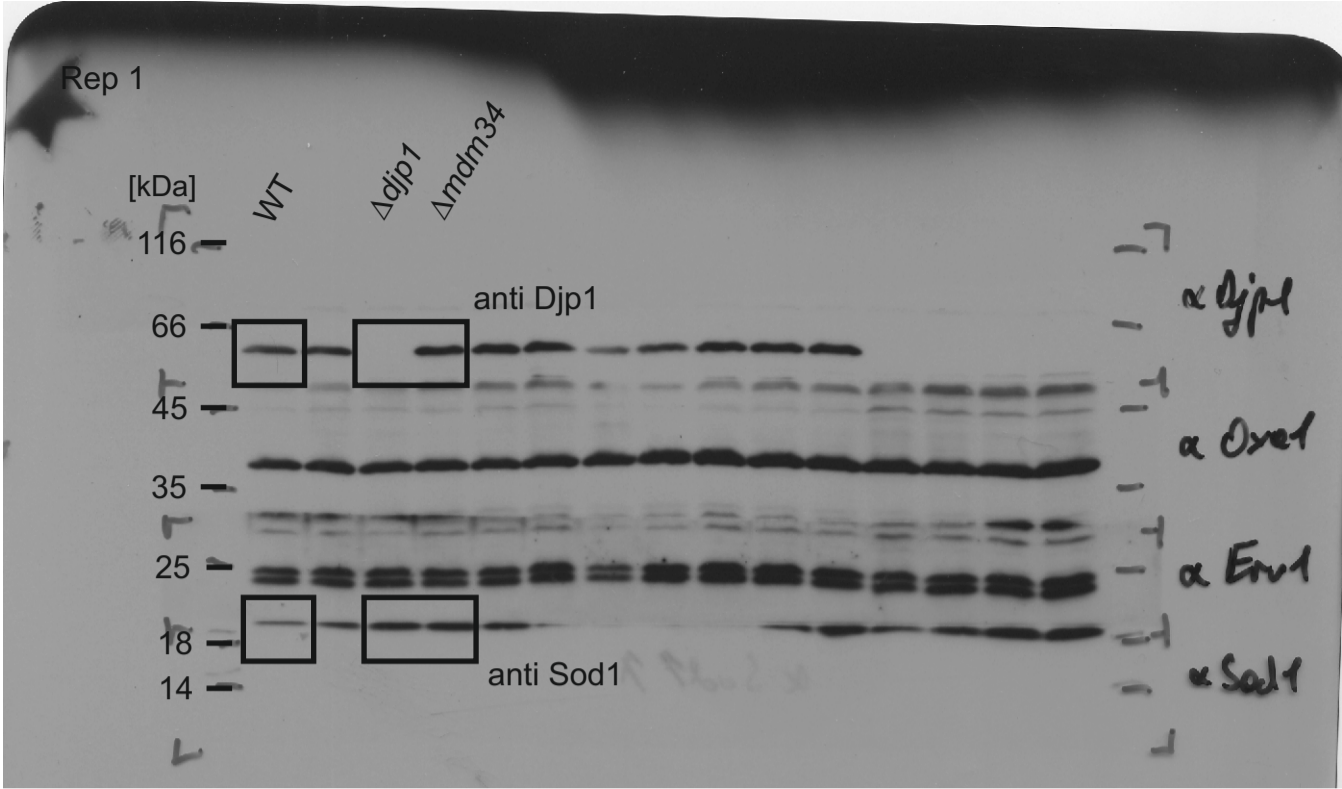

For quantification:

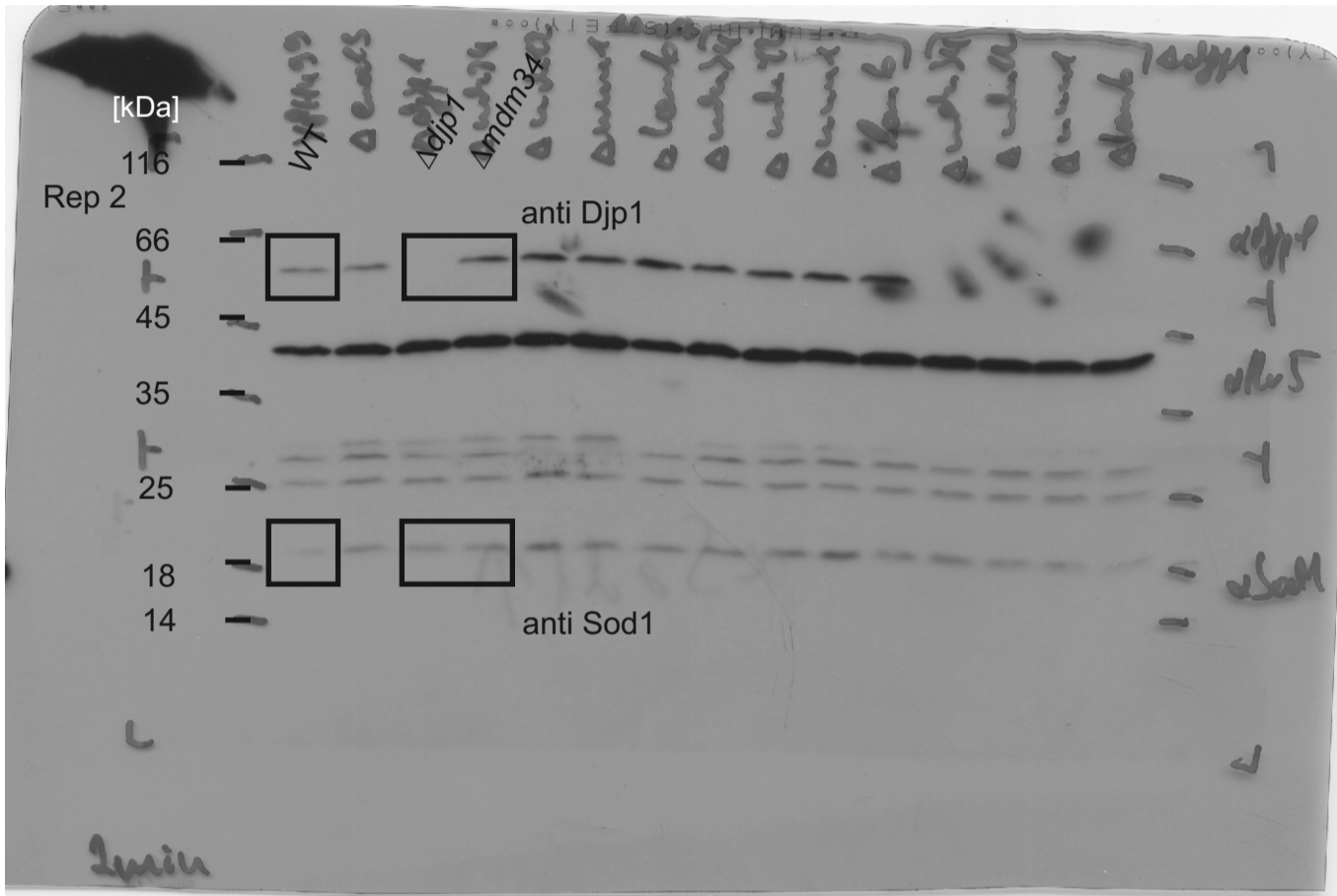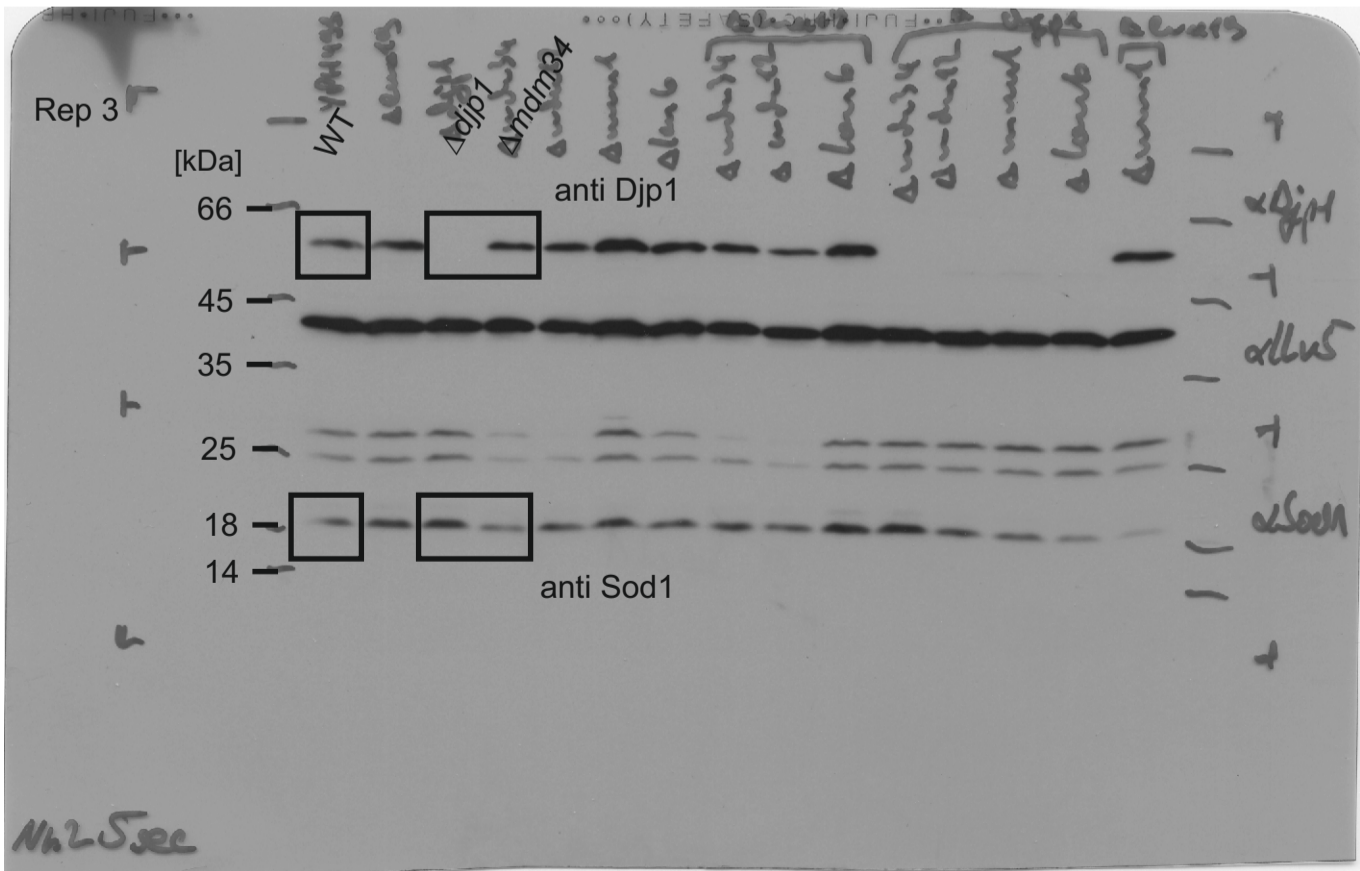

Supplement: Supplementary file 15 — Figure EV1-5 and Appendix S2 Source Data [file 44319_2024_113_MOESM15_ESM.zip › Koch_SourceData_FigEVall/EV1/EV1A.pdf]

Figure EV1D

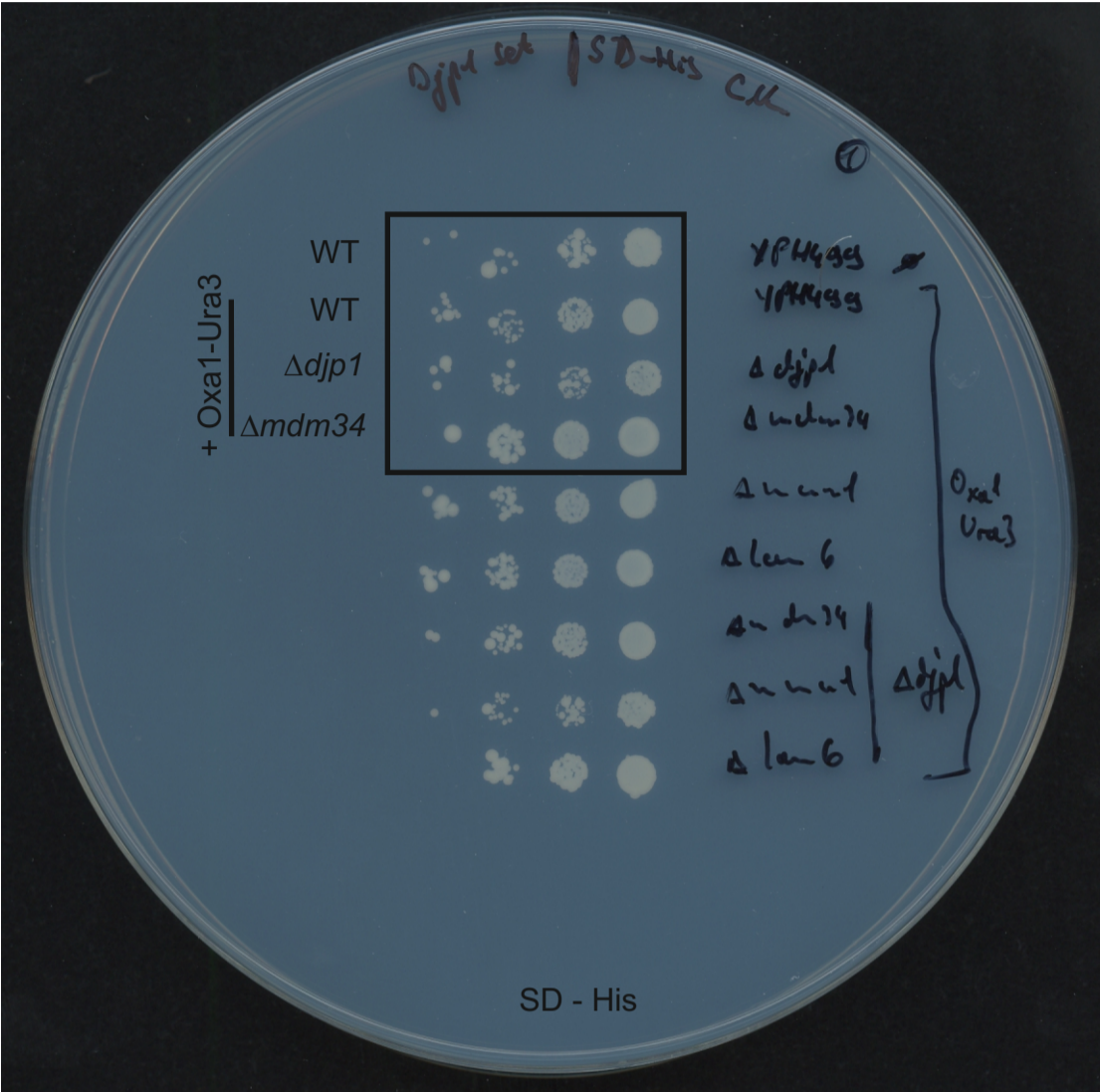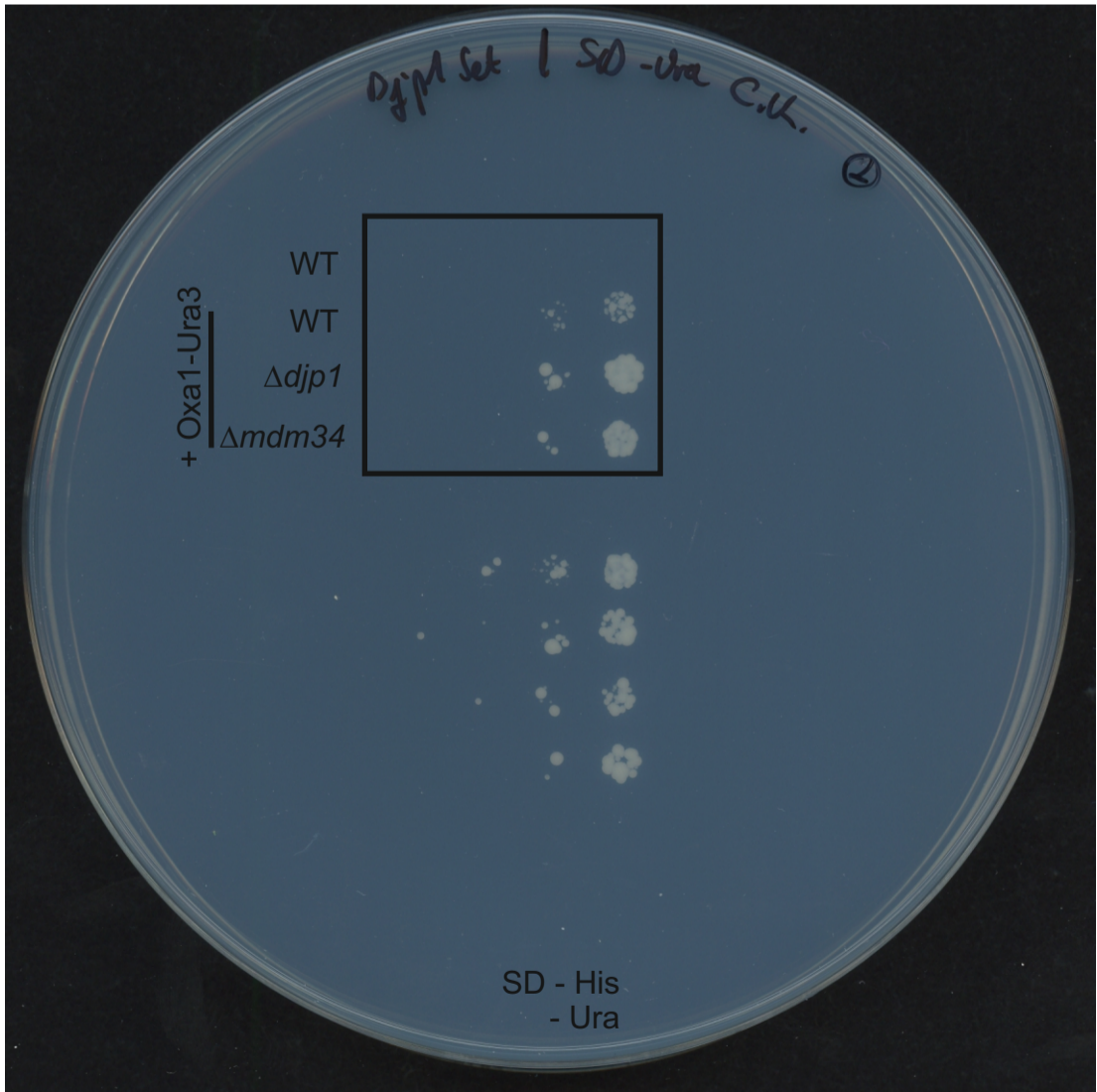

Supplement: Supplementary file 15 — Figure EV1-5 and Appendix S2 Source Data [file 44319_2024_113_MOESM15_ESM.zip › Koch_SourceData_FigEVall/EV1/EV1D.pdf]

Figure EV1E

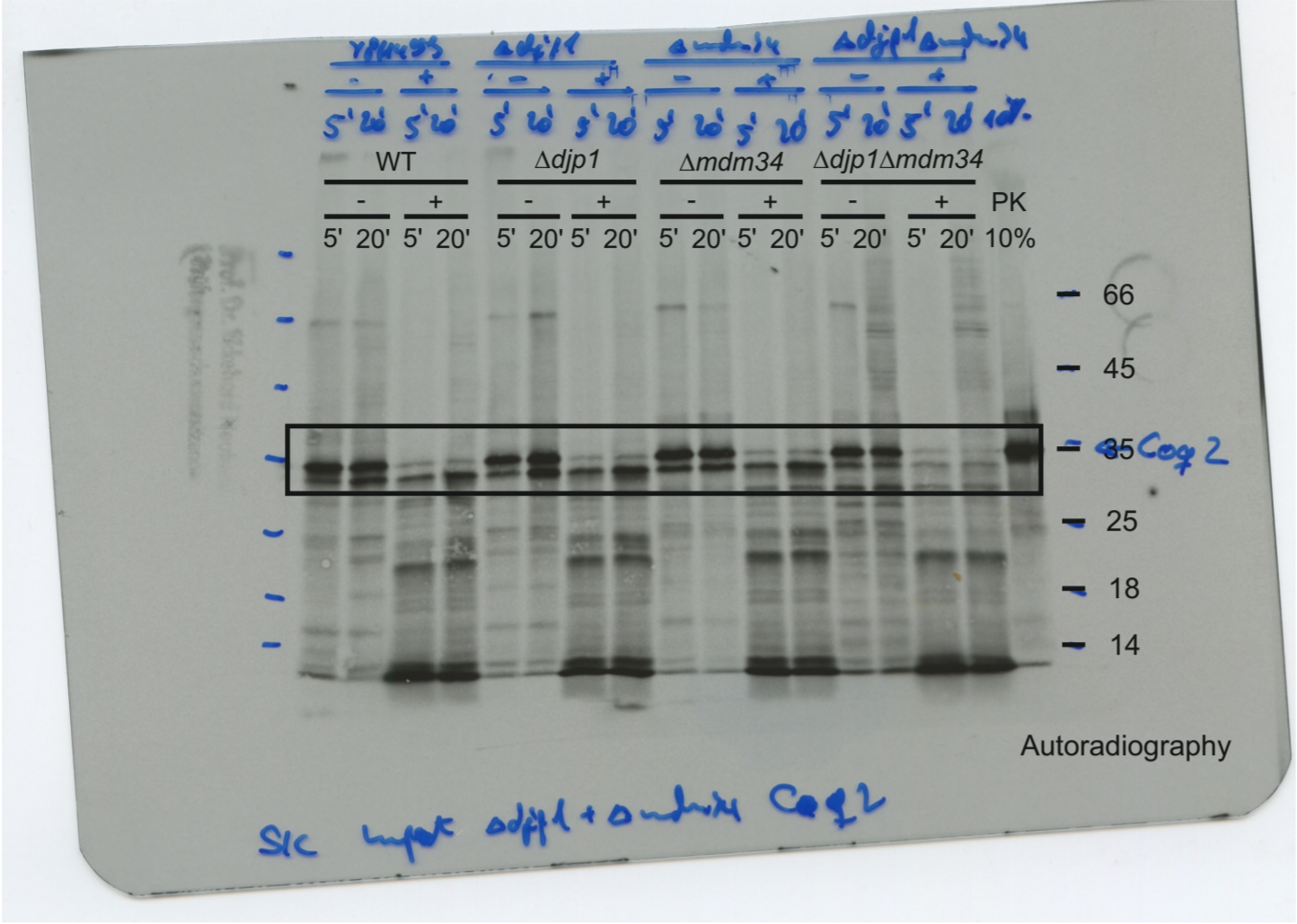

Figure EV1F

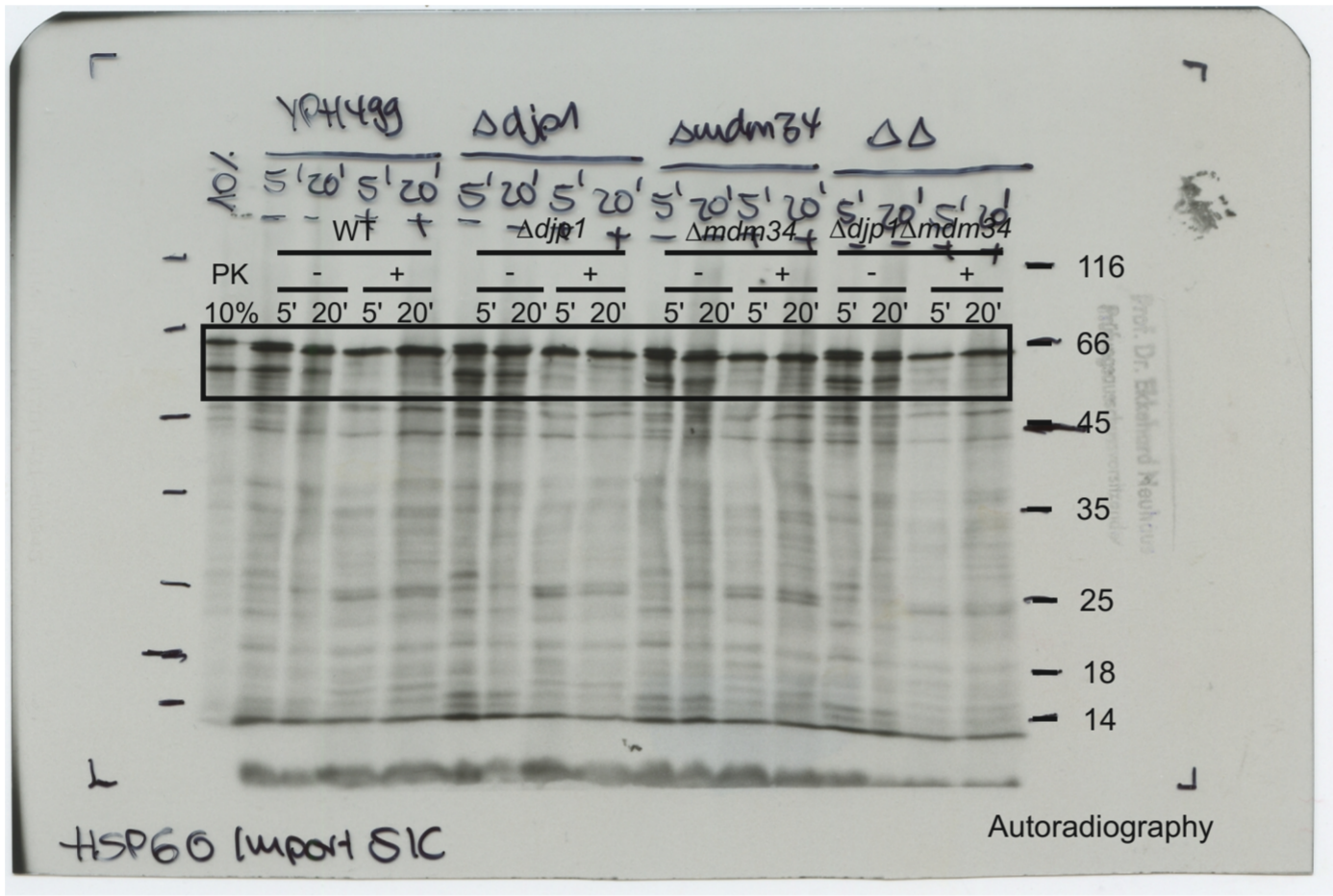

Figure EV1G

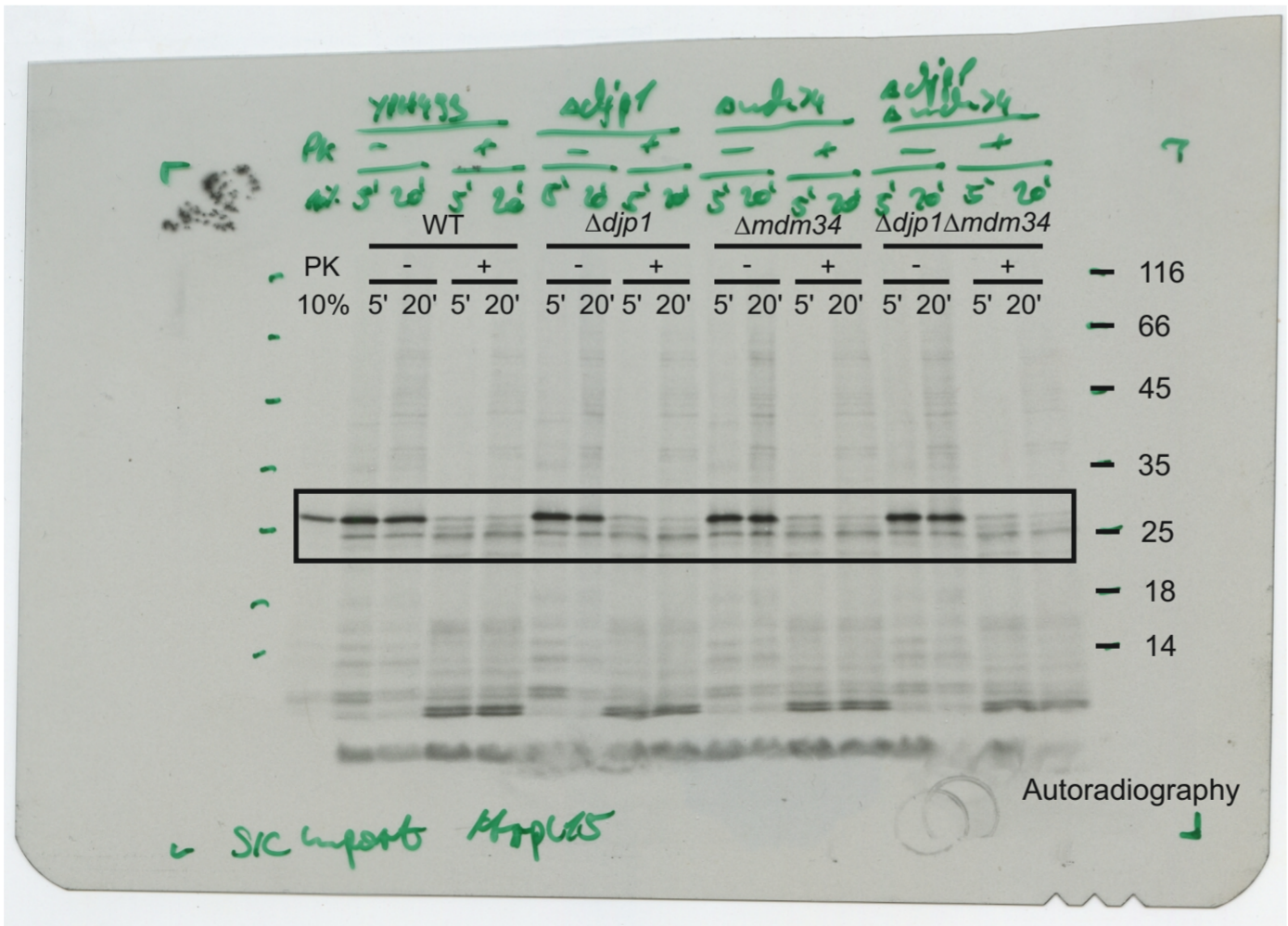

Supplement: Supplementary file 15 — Figure EV1-5 and Appendix S2 Source Data [file 44319_2024_113_MOESM15_ESM.zip › Koch_SourceData_FigEVall/EV1/EV1E-G.pdf]

Figure EV2C

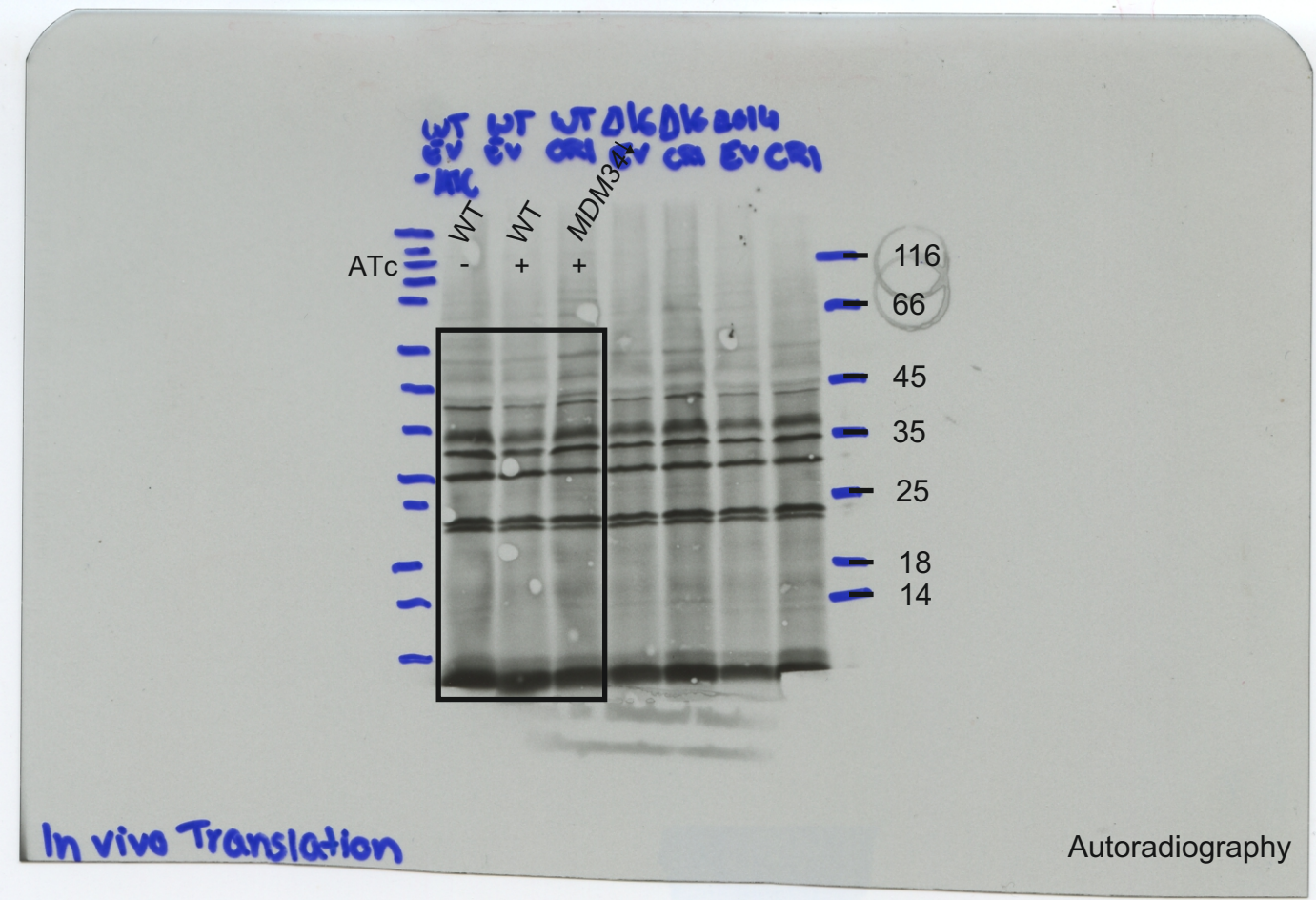

Supplement: Supplementary file 15 — Figure EV1-5 and Appendix S2 Source Data [file 44319_2024_113_MOESM15_ESM.zip › Koch_SourceData_FigEVall/EV2/EV2C.pdf]

Figure EV3A

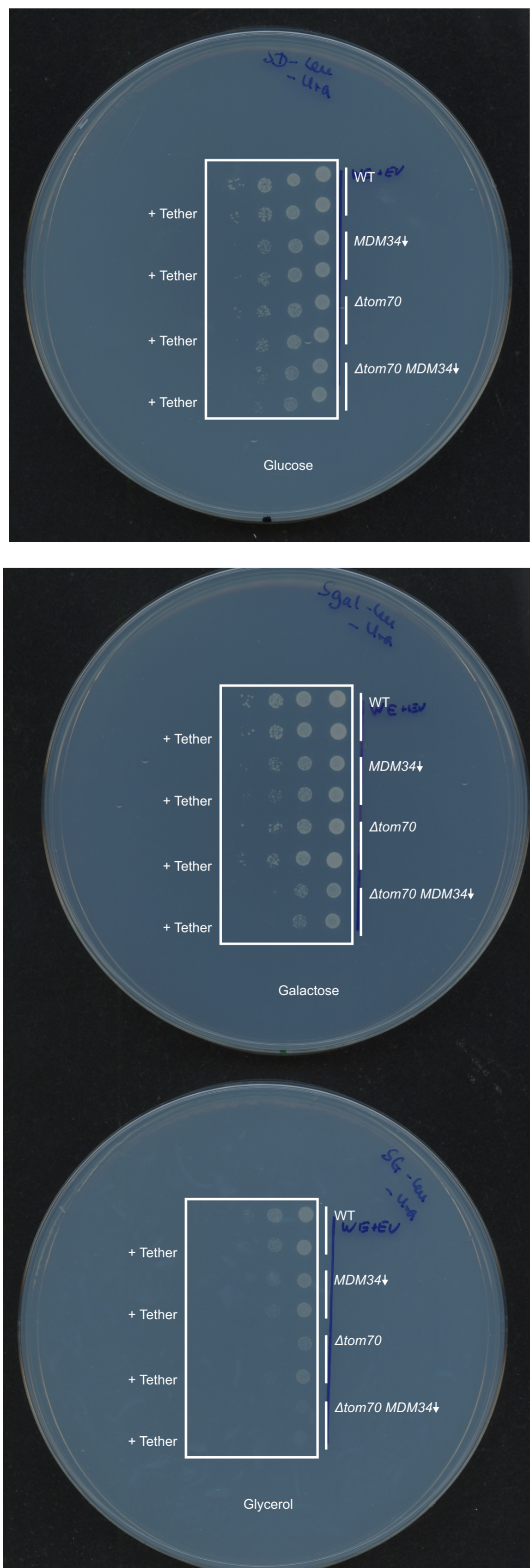

Supplement: Supplementary file 15 — Figure EV1-5 and Appendix S2 Source Data [file 44319_2024_113_MOESM15_ESM.zip › Koch_SourceData_FigEVall/EV3/EV3A.pdf]

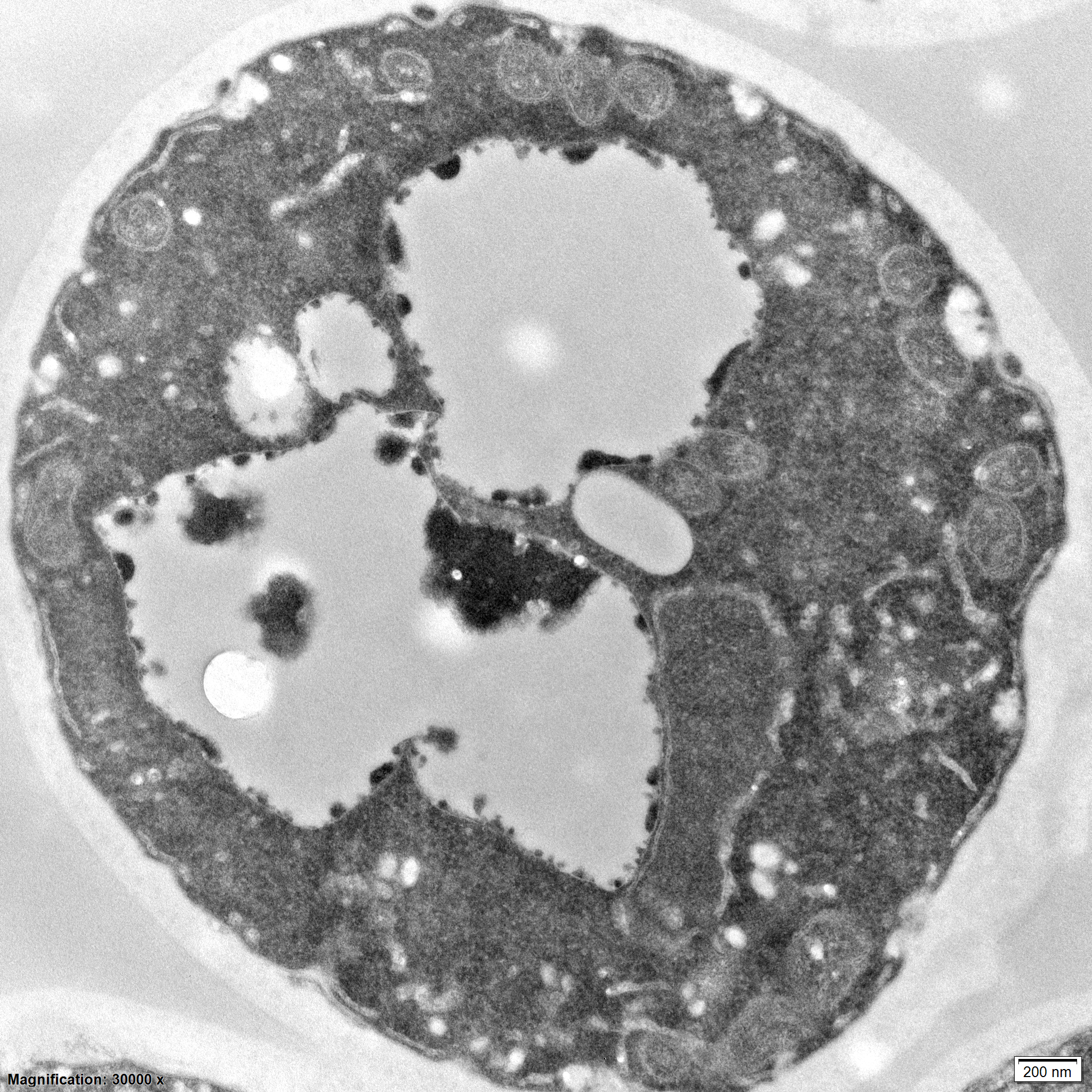

Supplement: Supplementary file 15 — Figure EV1-5 and Appendix S2 Source Data [file 44319_2024_113_MOESM15_ESM.zip › Koch_SourceData_FigEVall/EV4/EV4A/Dtom70_left.tif]

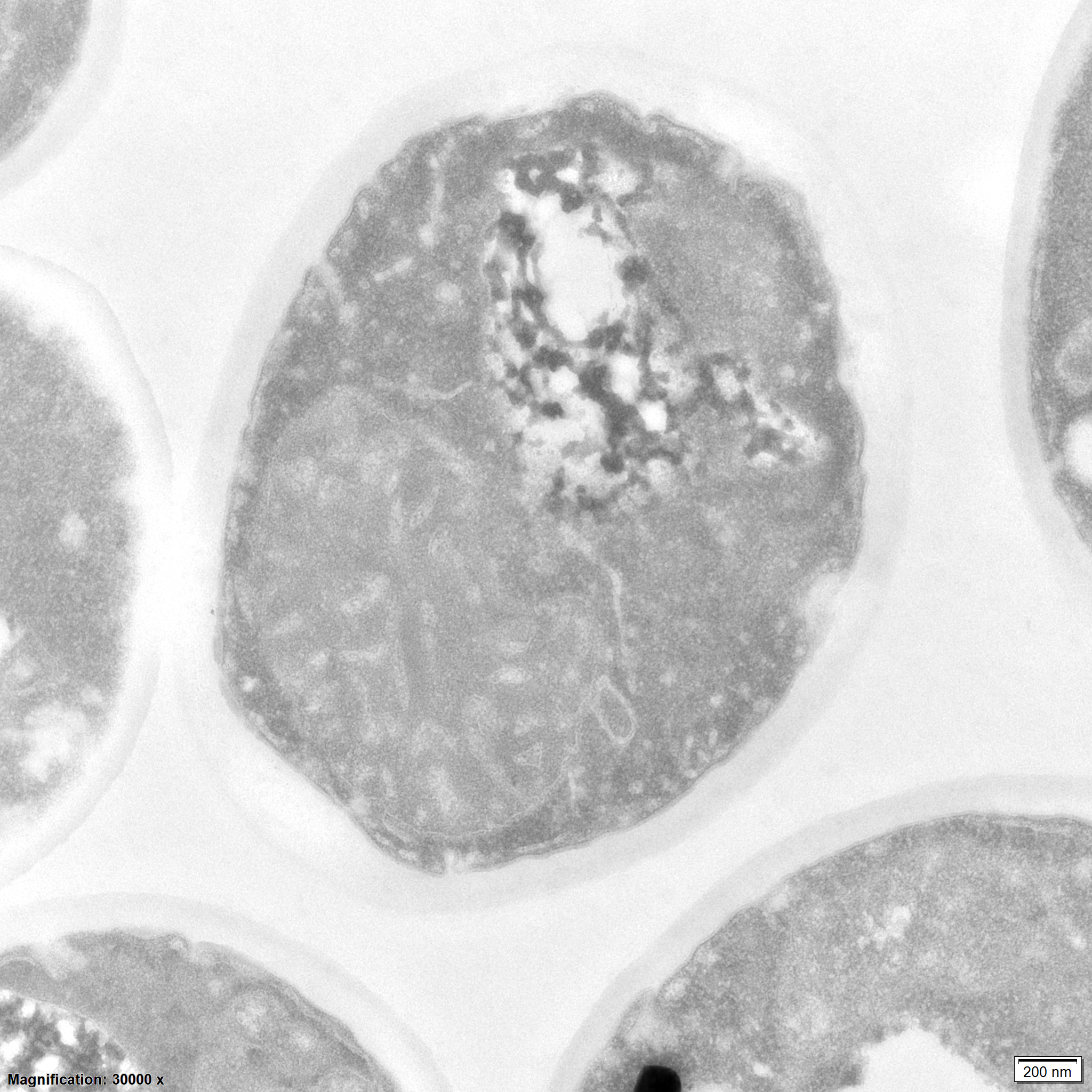

Supplement: Supplementary file 15 — Figure EV1-5 and Appendix S2 Source Data [file 44319_2024_113_MOESM15_ESM.zip › Koch_SourceData_FigEVall/EV4/EV4A/Dtom70_MDM34_left.tif]

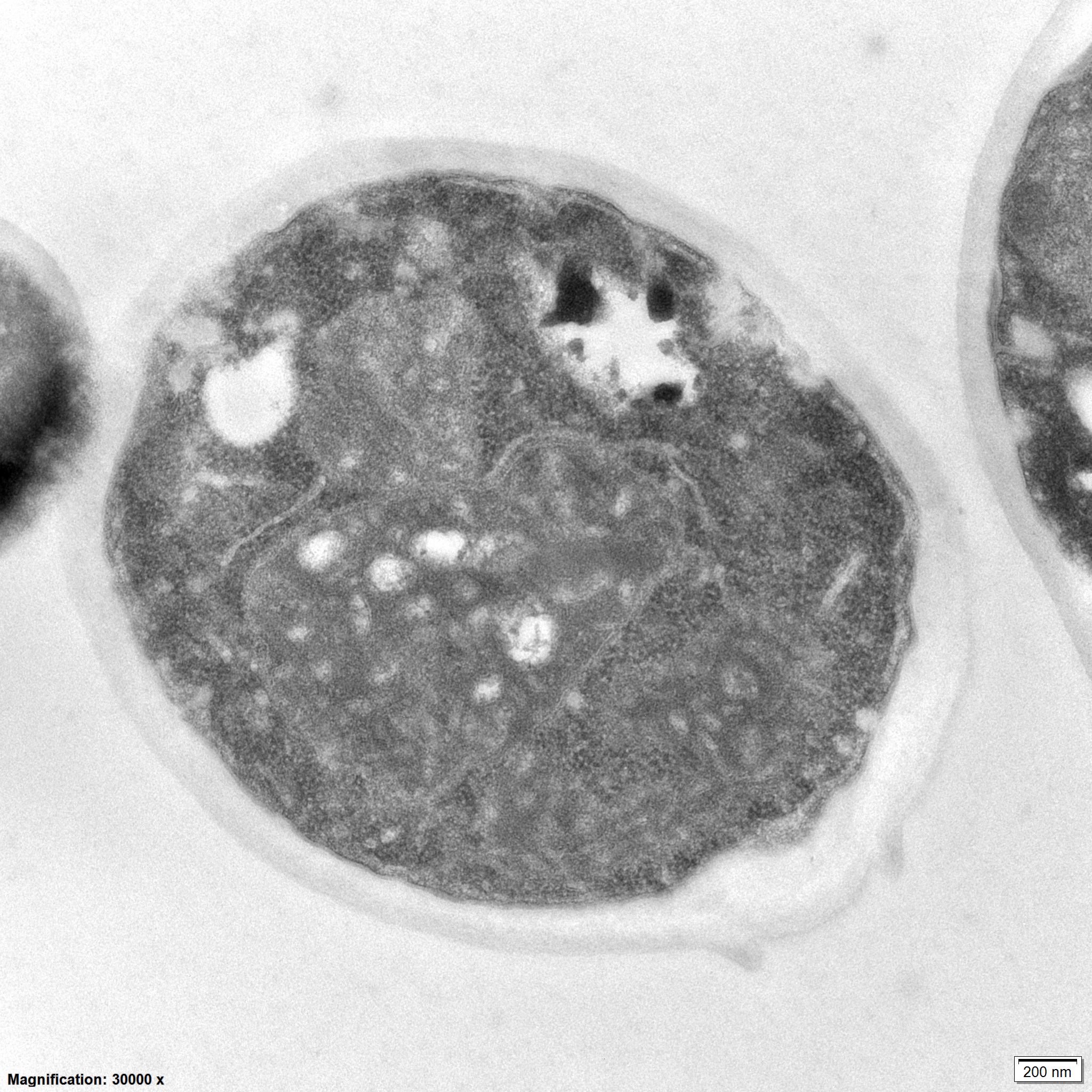

Supplement: Supplementary file 15 — Figure EV1-5 and Appendix S2 Source Data [file 44319_2024_113_MOESM15_ESM.zip › Koch_SourceData_FigEVall/EV4/EV4A/Dtom70_MDM34_middle_left.tif]

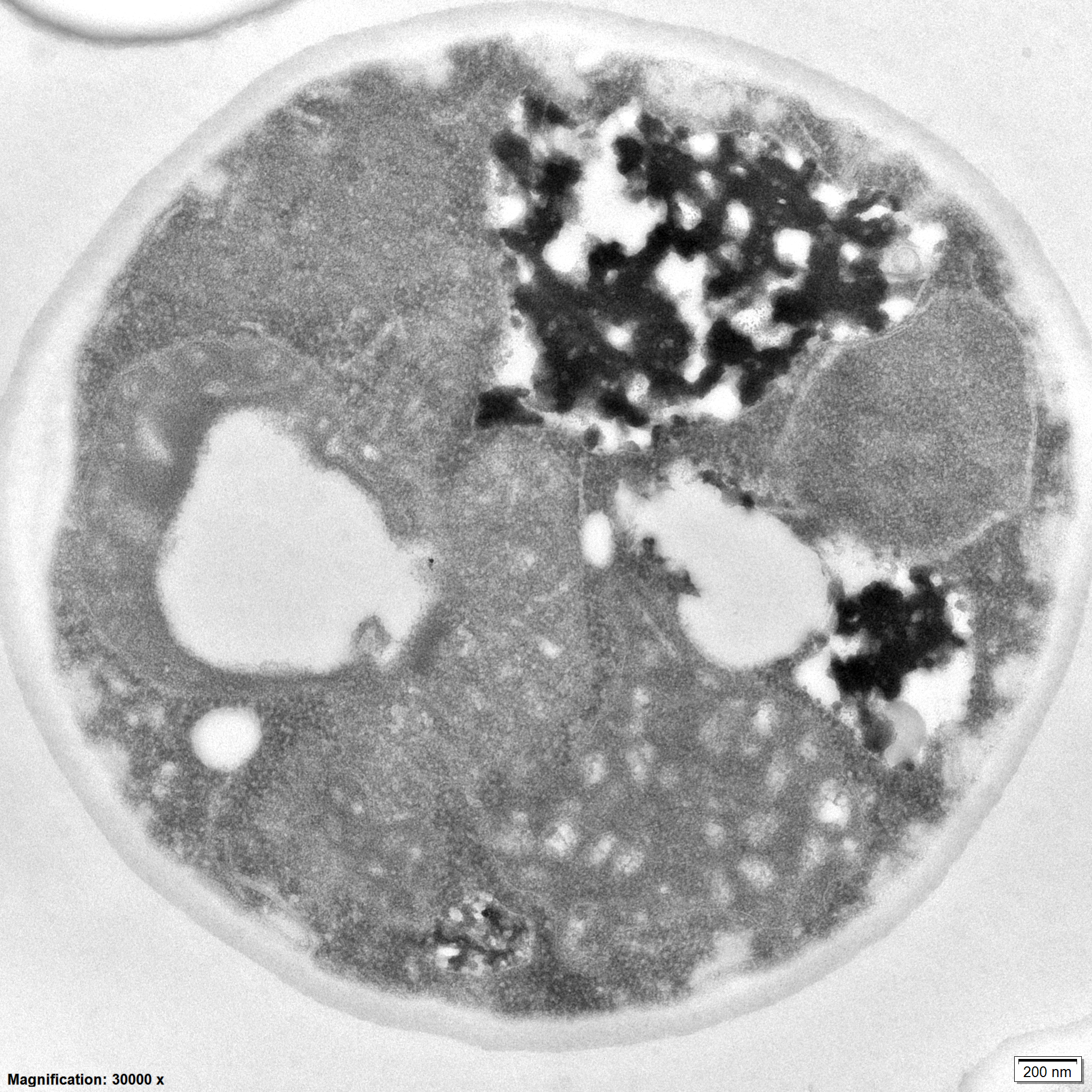

Supplement: Supplementary file 15 — Figure EV1-5 and Appendix S2 Source Data [file 44319_2024_113_MOESM15_ESM.zip › Koch_SourceData_FigEVall/EV4/EV4A/Dtom70_MDM34_middle_right.tif]

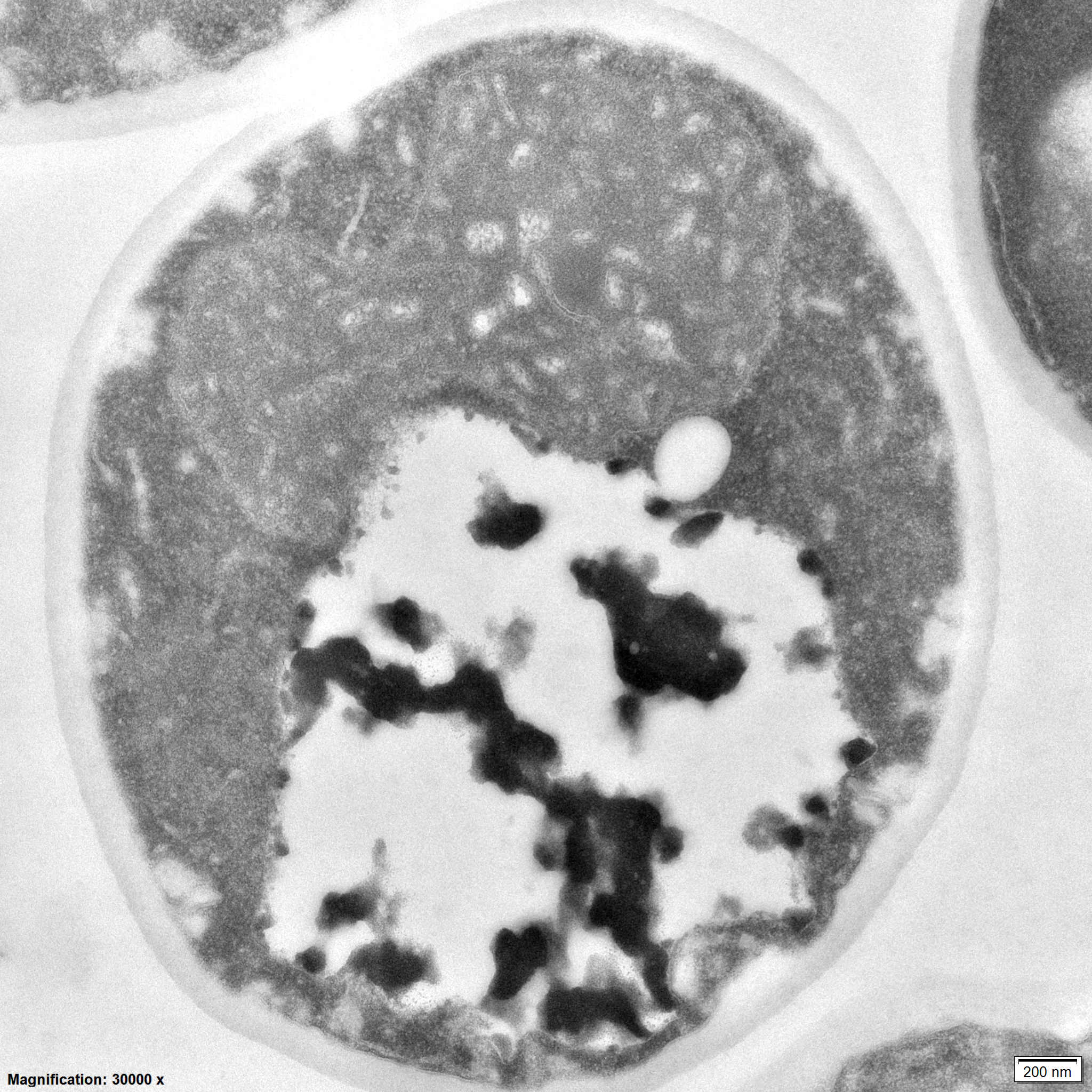

Supplement: Supplementary file 15 — Figure EV1-5 and Appendix S2 Source Data [file 44319_2024_113_MOESM15_ESM.zip › Koch_SourceData_FigEVall/EV4/EV4A/Dtom70_MDM34_right.tif]

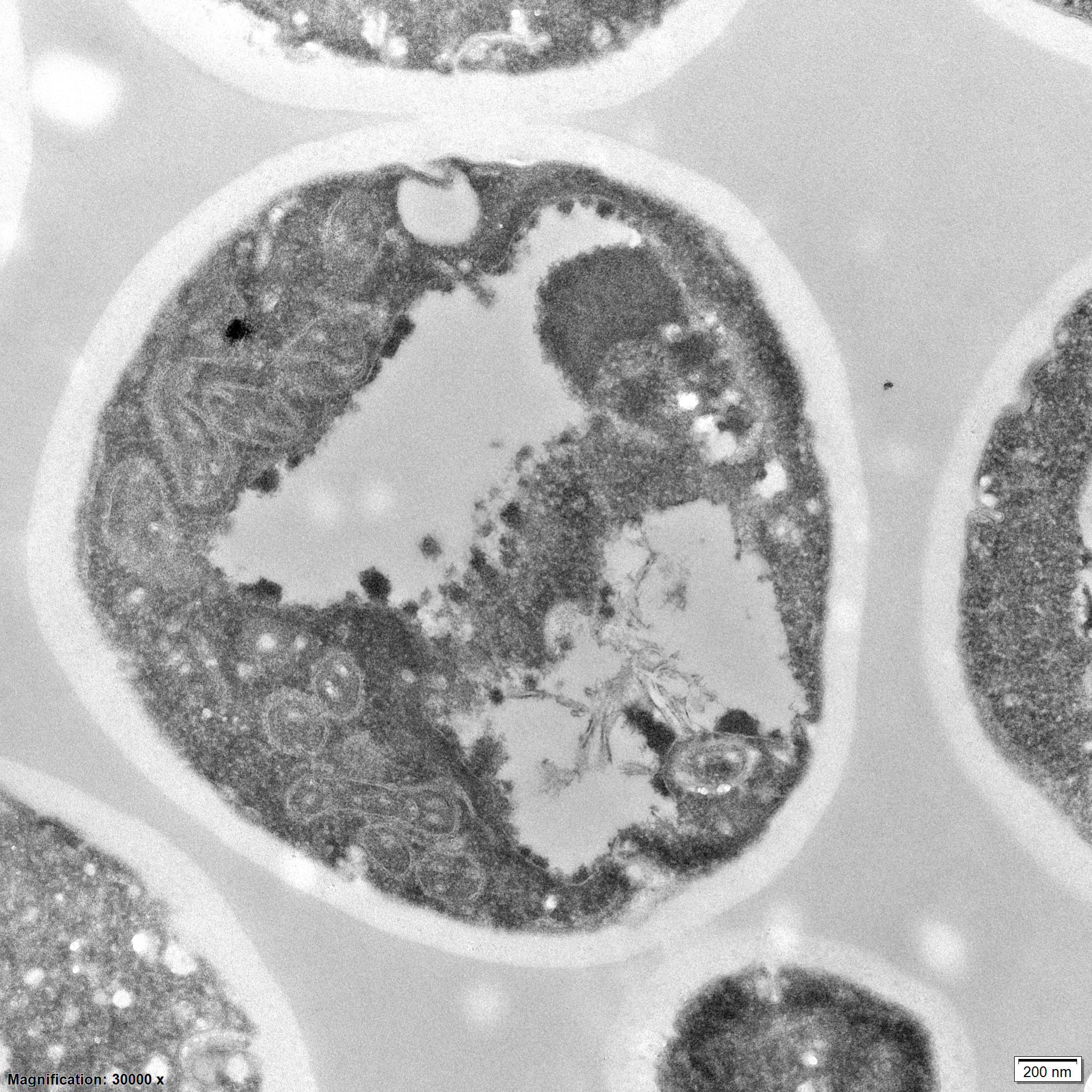

Supplement: Supplementary file 15 — Figure EV1-5 and Appendix S2 Source Data [file 44319_2024_113_MOESM15_ESM.zip › Koch_SourceData_FigEVall/EV4/EV4A/Dtom70_middle_left.tif]

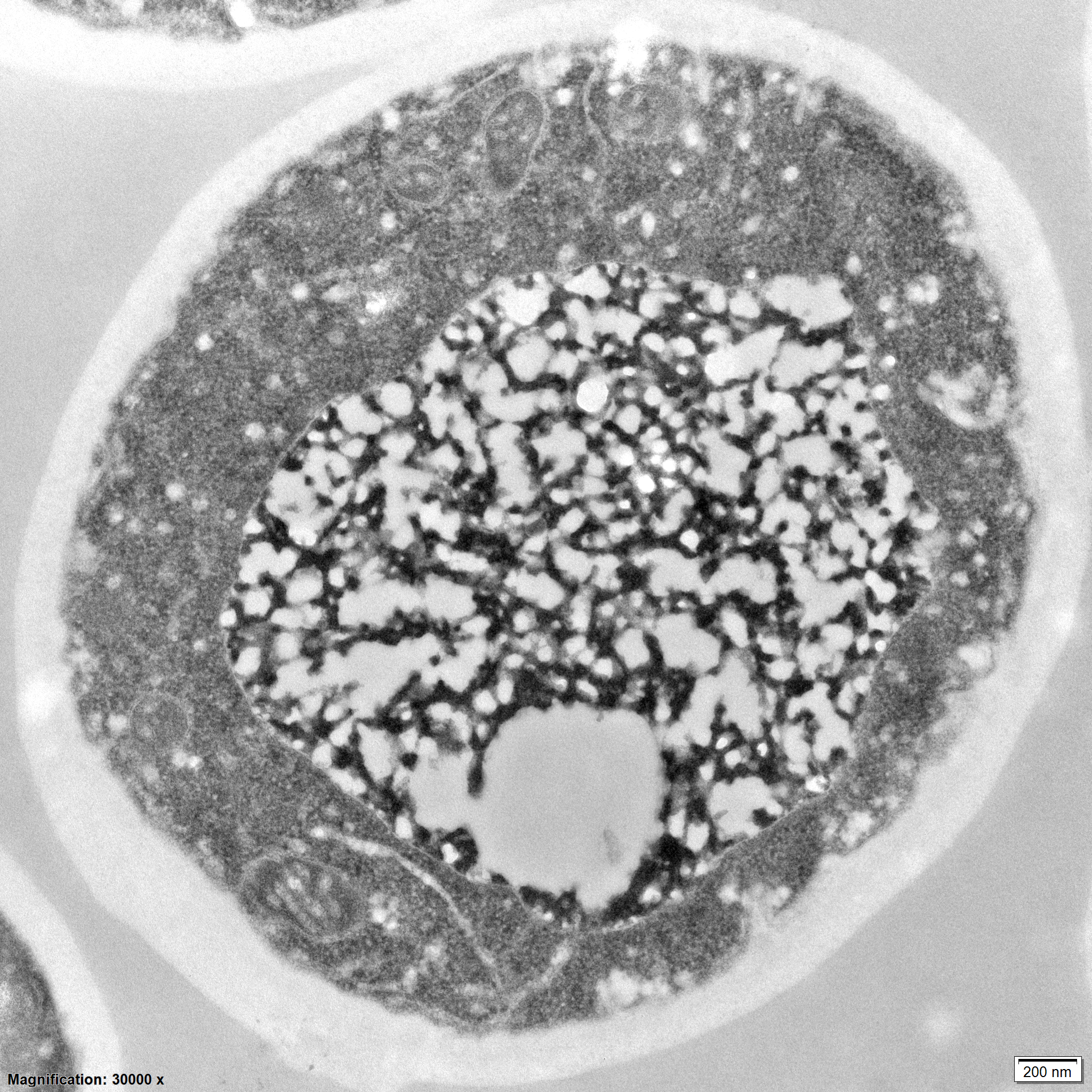

Supplement: Supplementary file 15 — Figure EV1-5 and Appendix S2 Source Data [file 44319_2024_113_MOESM15_ESM.zip › Koch_SourceData_FigEVall/EV4/EV4A/Dtom70_middle_right.tif]

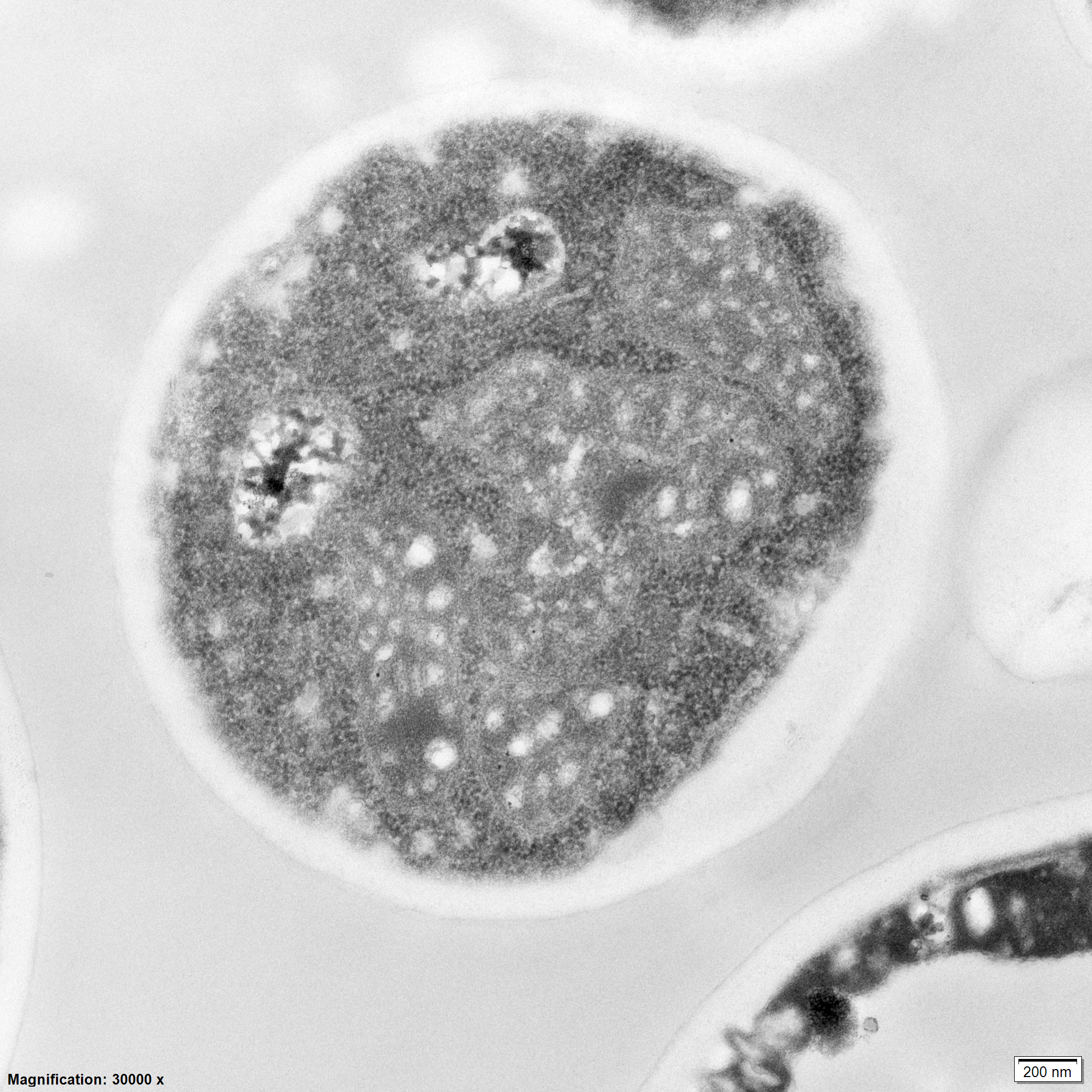

Supplement: Supplementary file 15 — Figure EV1-5 and Appendix S2 Source Data [file 44319_2024_113_MOESM15_ESM.zip › Koch_SourceData_FigEVall/EV4/EV4A/MDM34_left.tif]

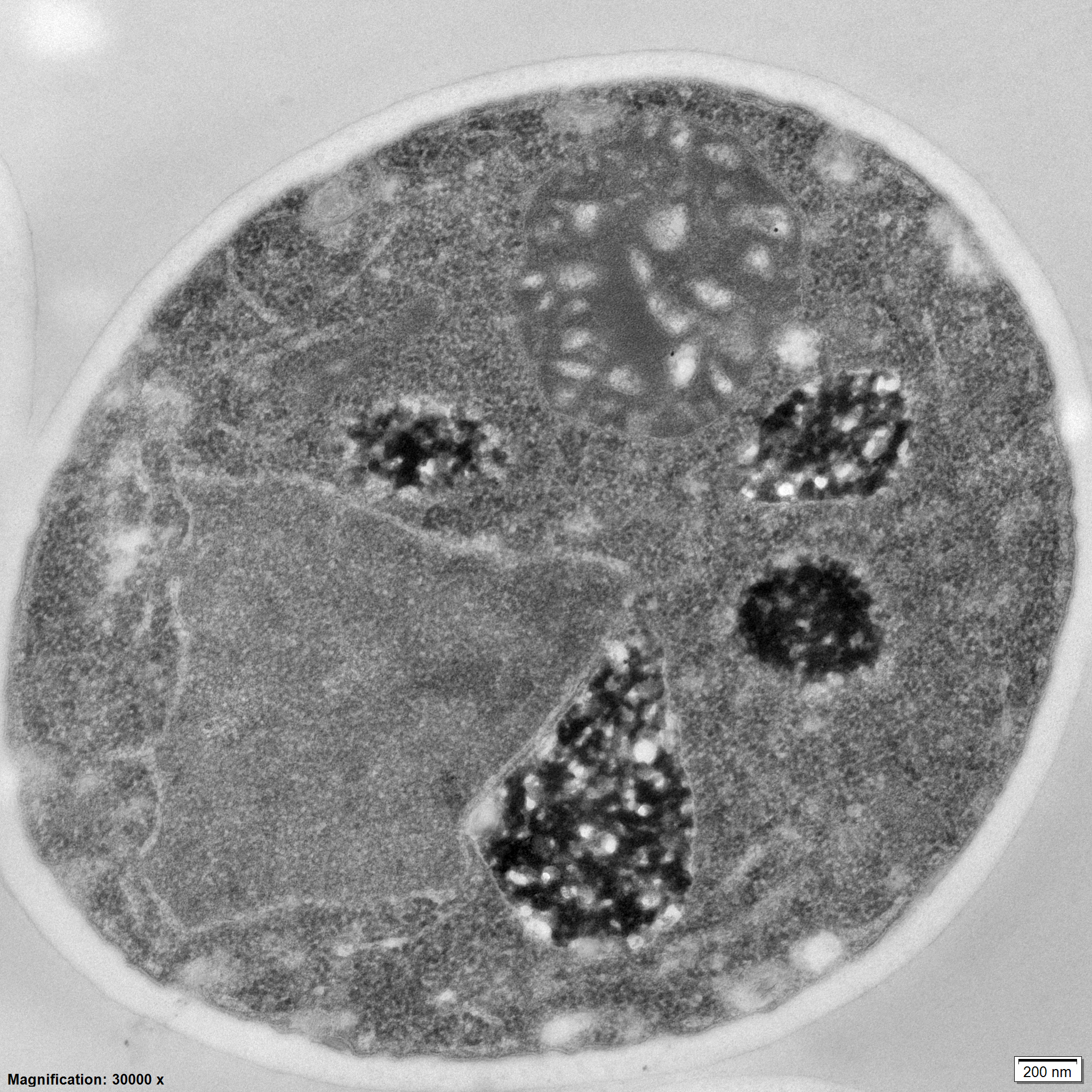

Supplement: Supplementary file 15 — Figure EV1-5 and Appendix S2 Source Data [file 44319_2024_113_MOESM15_ESM.zip › Koch_SourceData_FigEVall/EV4/EV4A/MDM34_middle_left.tif]

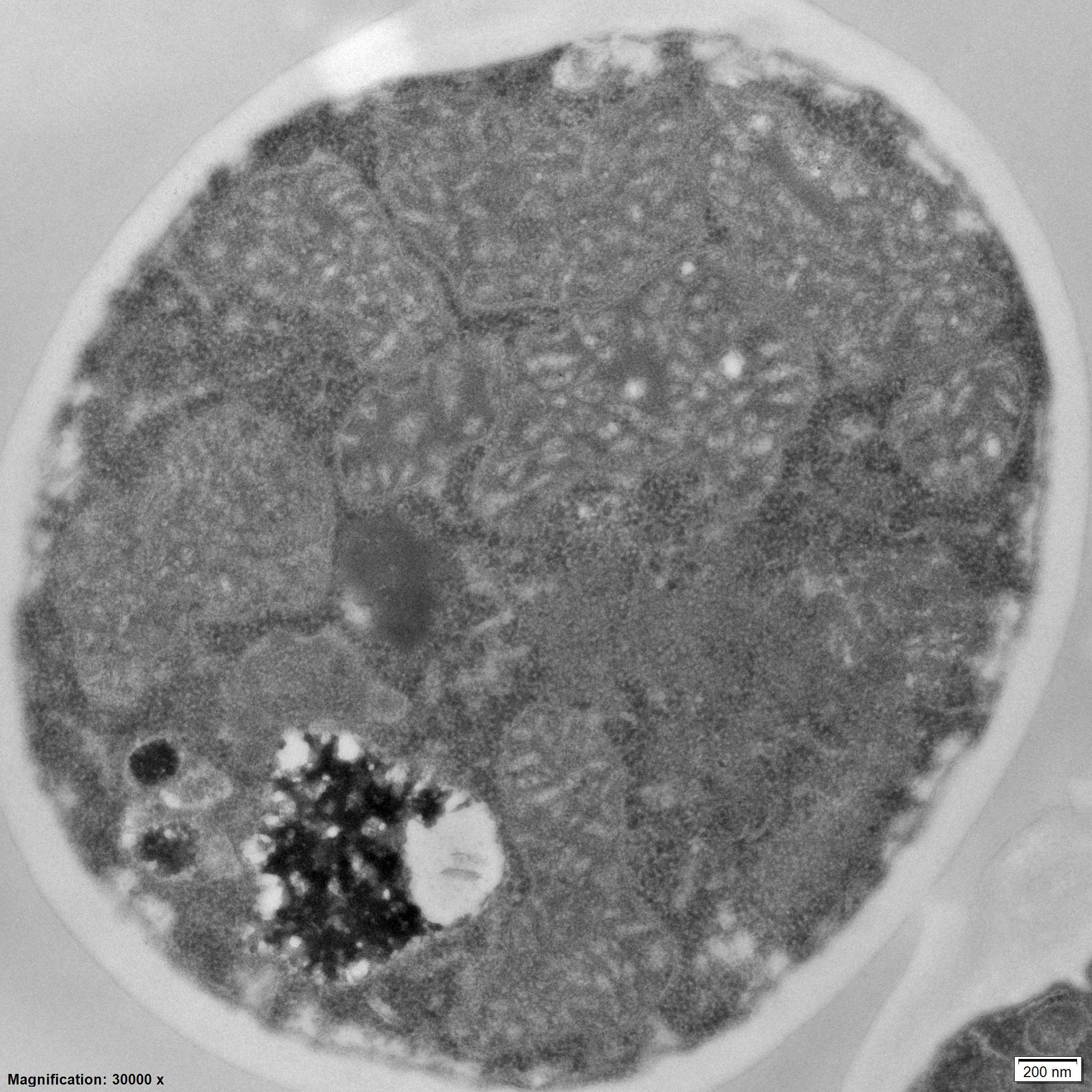

Supplement: Supplementary file 15 — Figure EV1-5 and Appendix S2 Source Data [file 44319_2024_113_MOESM15_ESM.zip › Koch_SourceData_FigEVall/EV4/EV4A/MDM34_middle_right.tif]

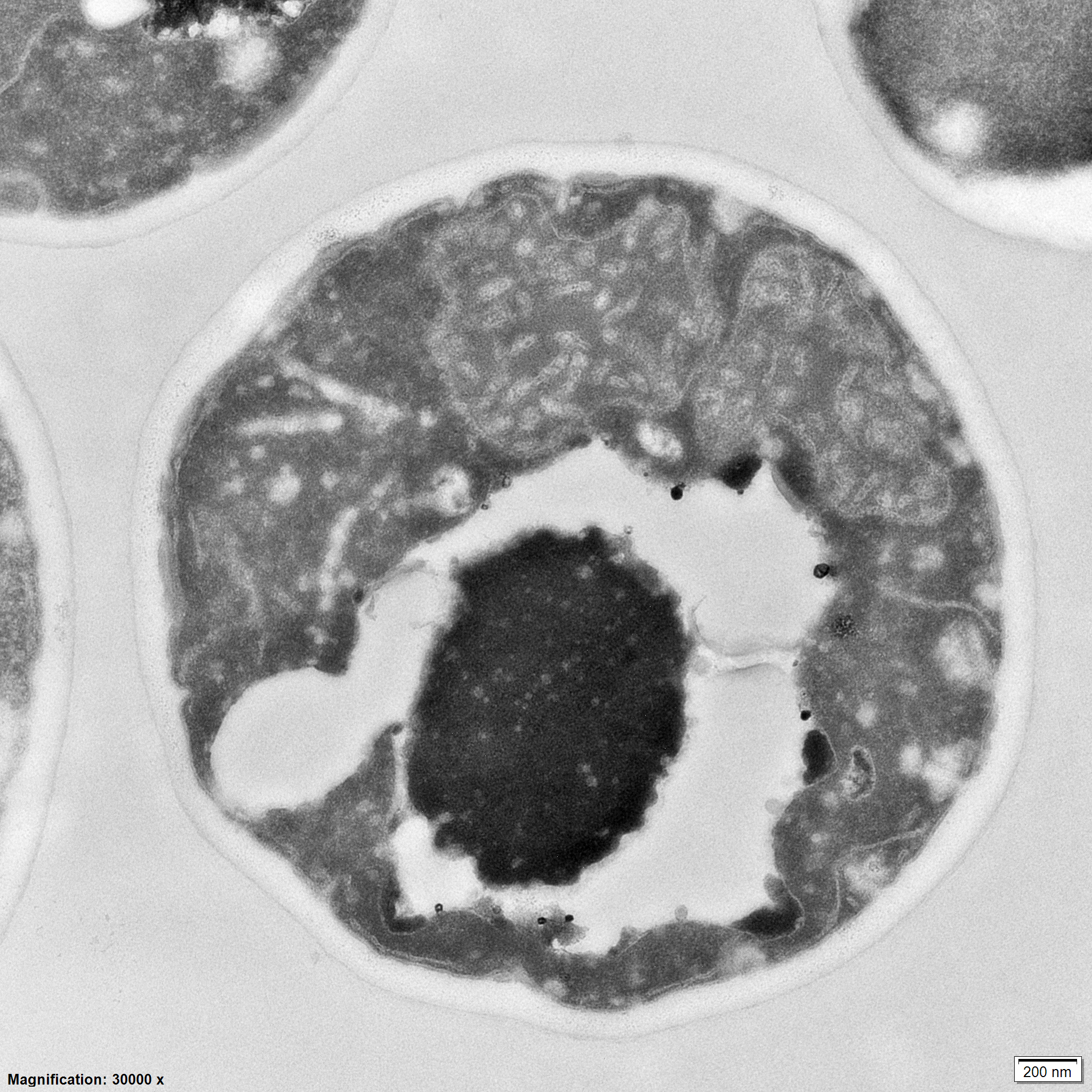

Supplement: Supplementary file 15 — Figure EV1-5 and Appendix S2 Source Data [file 44319_2024_113_MOESM15_ESM.zip › Koch_SourceData_FigEVall/EV4/EV4A/MDM34_right.tif]

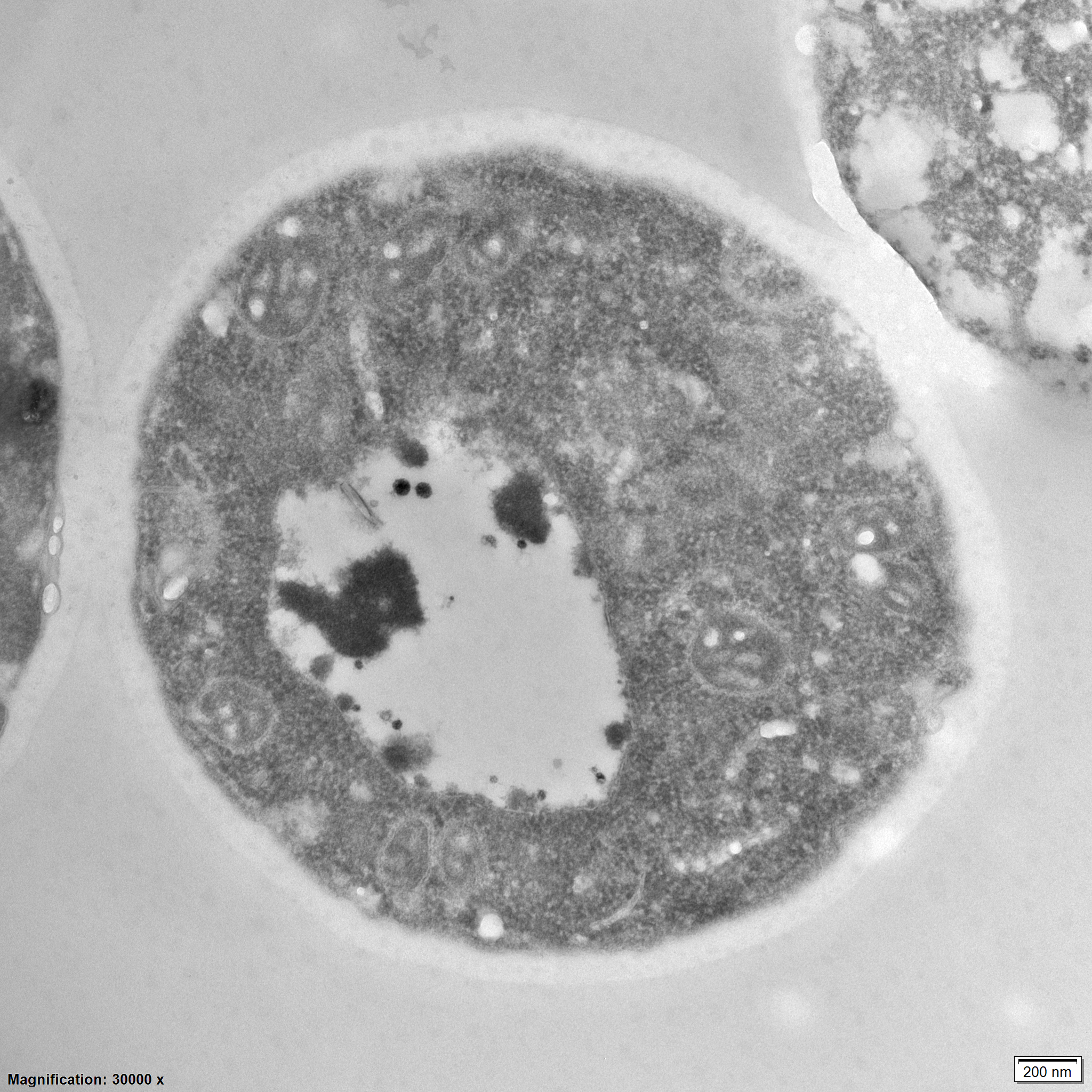

Supplement: Supplementary file 15 — Figure EV1-5 and Appendix S2 Source Data [file 44319_2024_113_MOESM15_ESM.zip › Koch_SourceData_FigEVall/EV4/EV4A/WT_left.tif]

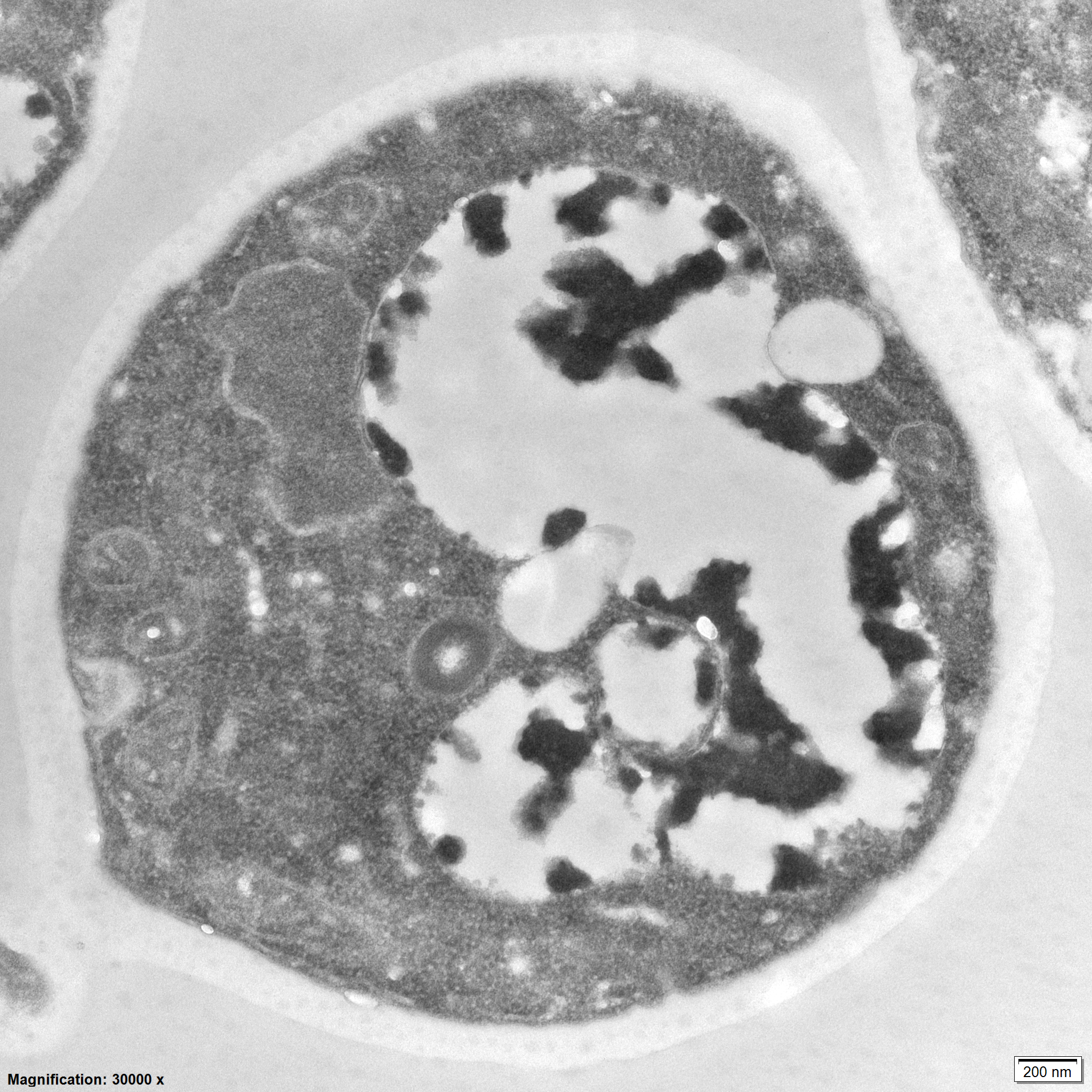

Supplement: Supplementary file 15 — Figure EV1-5 and Appendix S2 Source Data [file 44319_2024_113_MOESM15_ESM.zip › Koch_SourceData_FigEVall/EV4/EV4A/WT_middle_left.tif]

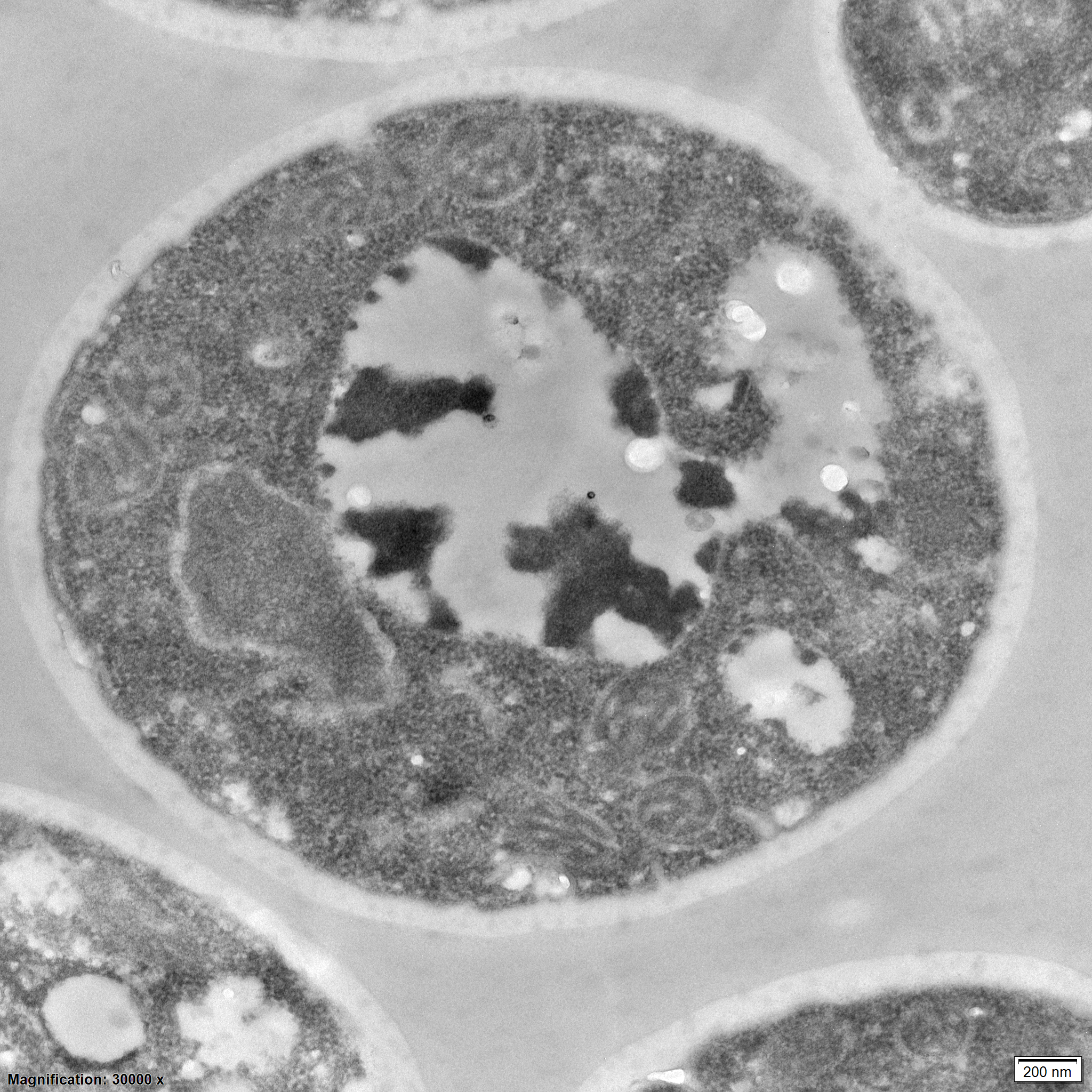

Supplement: Supplementary file 15 — Figure EV1-5 and Appendix S2 Source Data [file 44319_2024_113_MOESM15_ESM.zip › Koch_SourceData_FigEVall/EV4/EV4A/WT_middle_right.tif]

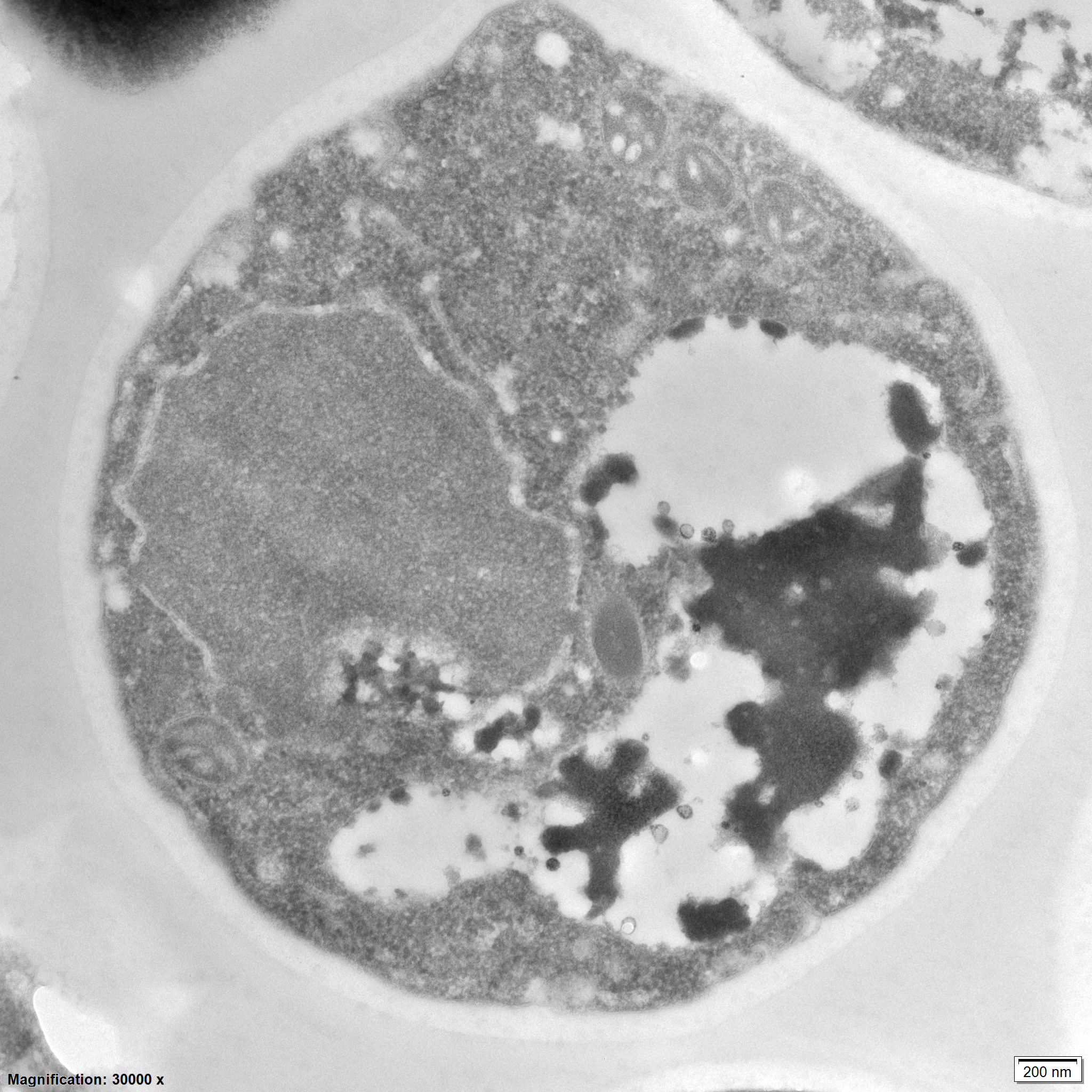

Supplement: Supplementary file 15 — Figure EV1-5 and Appendix S2 Source Data [file 44319_2024_113_MOESM15_ESM.zip › Koch_SourceData_FigEVall/EV4/EV4A/WT_right.tif]

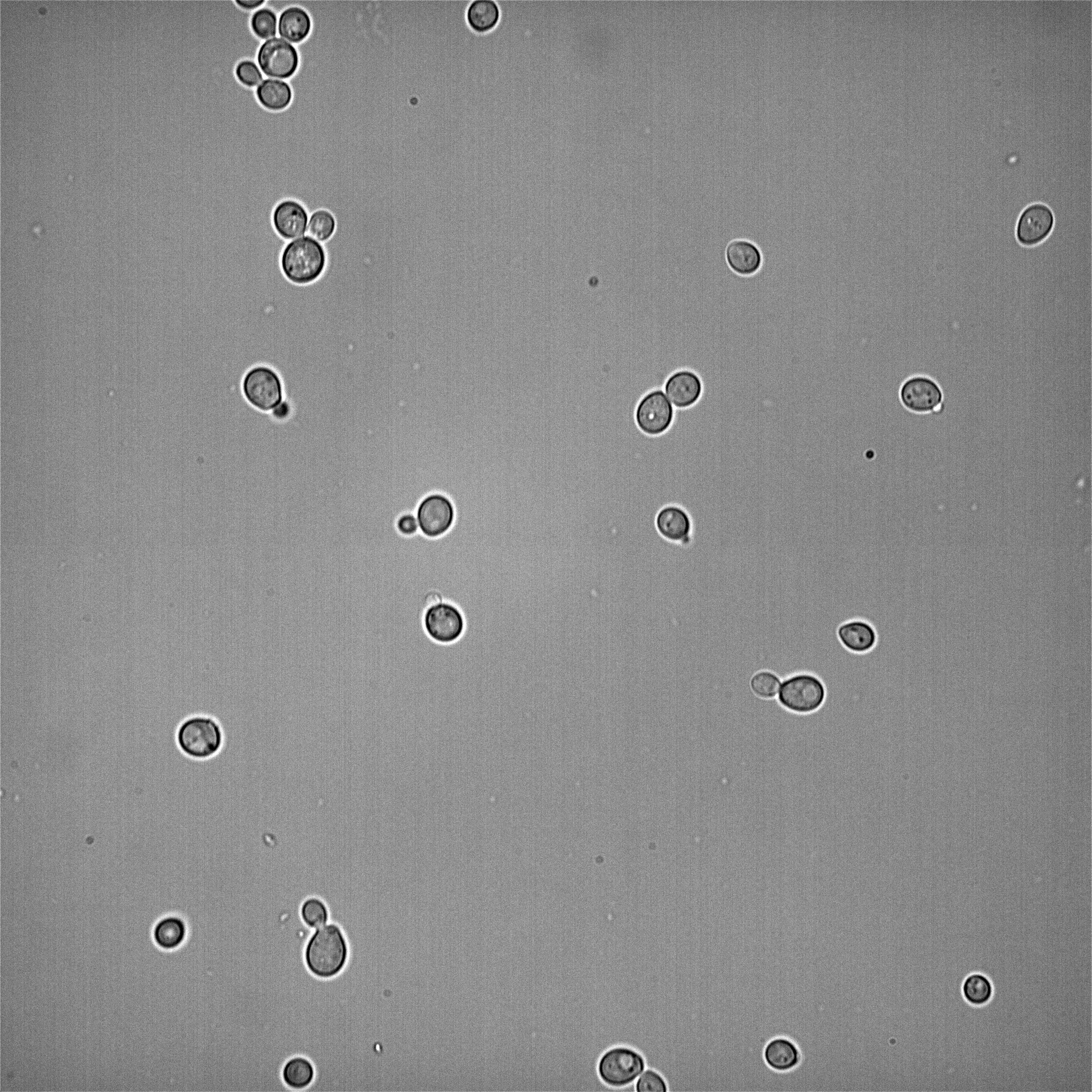

Supplement: Supplementary file 15 — Figure EV1-5 and Appendix S2 Source Data [file 44319_2024_113_MOESM15_ESM.zip › Koch_SourceData_FigEVall/EV4/EV4B/dtom70_BF.tif]

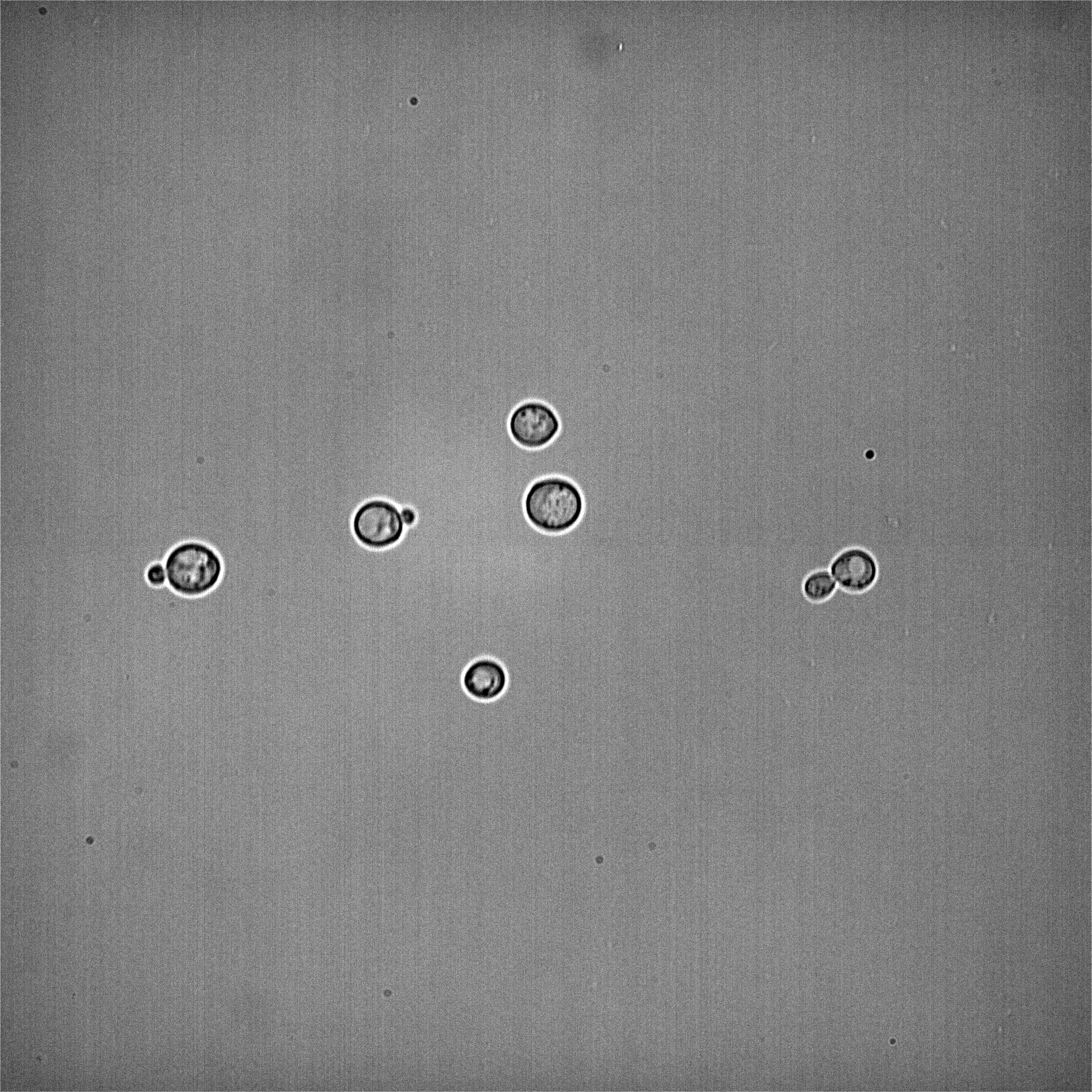

Supplement: Supplementary file 15 — Figure EV1-5 and Appendix S2 Source Data [file 44319_2024_113_MOESM15_ESM.zip › Koch_SourceData_FigEVall/EV4/EV4B/dtom70_MDM34_BF.tif]

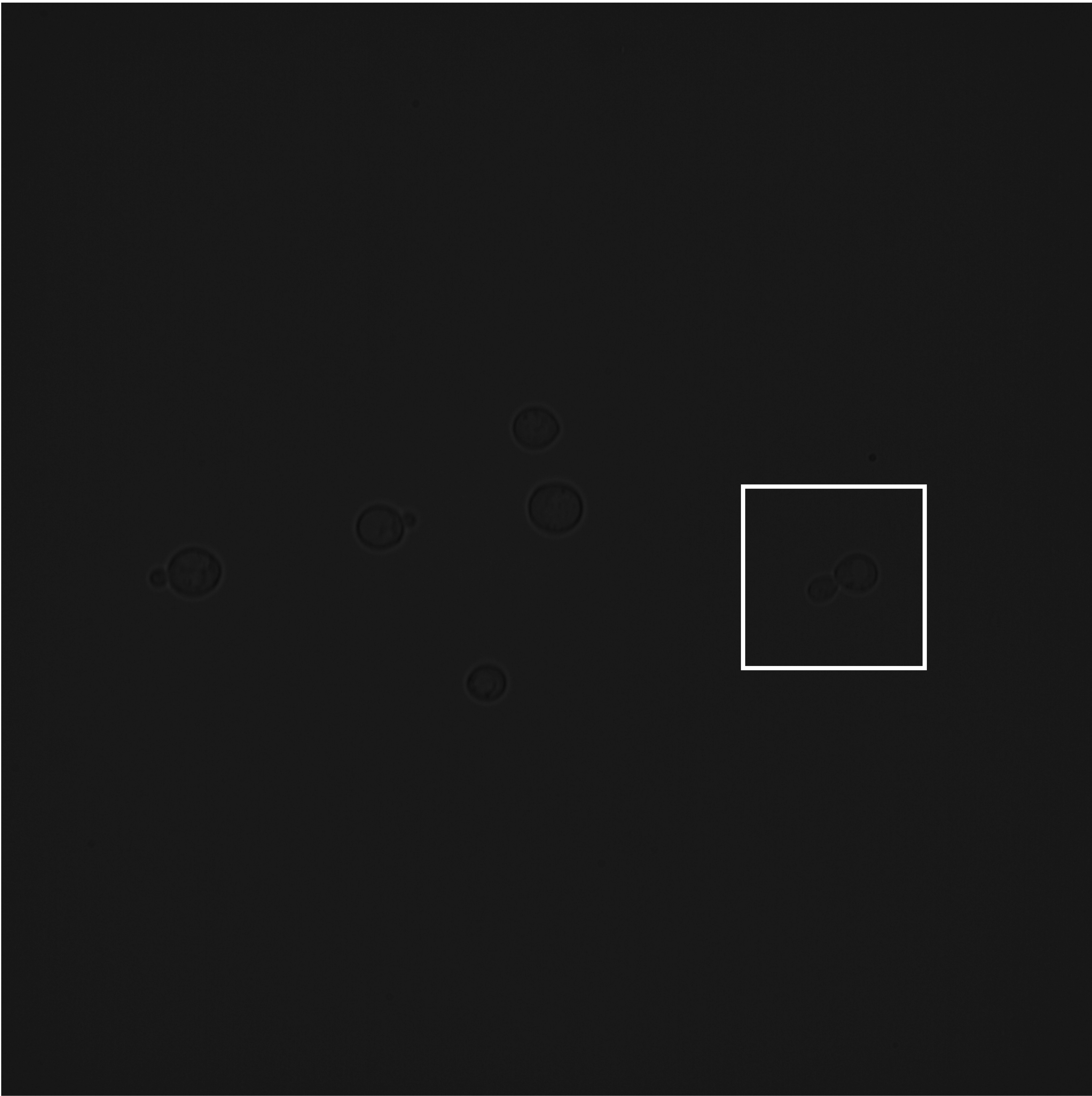

Supplement: Supplementary file 15 — Figure EV1-5 and Appendix S2 Source Data [file 44319_2024_113_MOESM15_ESM.zip › Koch_SourceData_FigEVall/EV4/EV4B/dtom70_MDM34_refrenceimage.pdf]

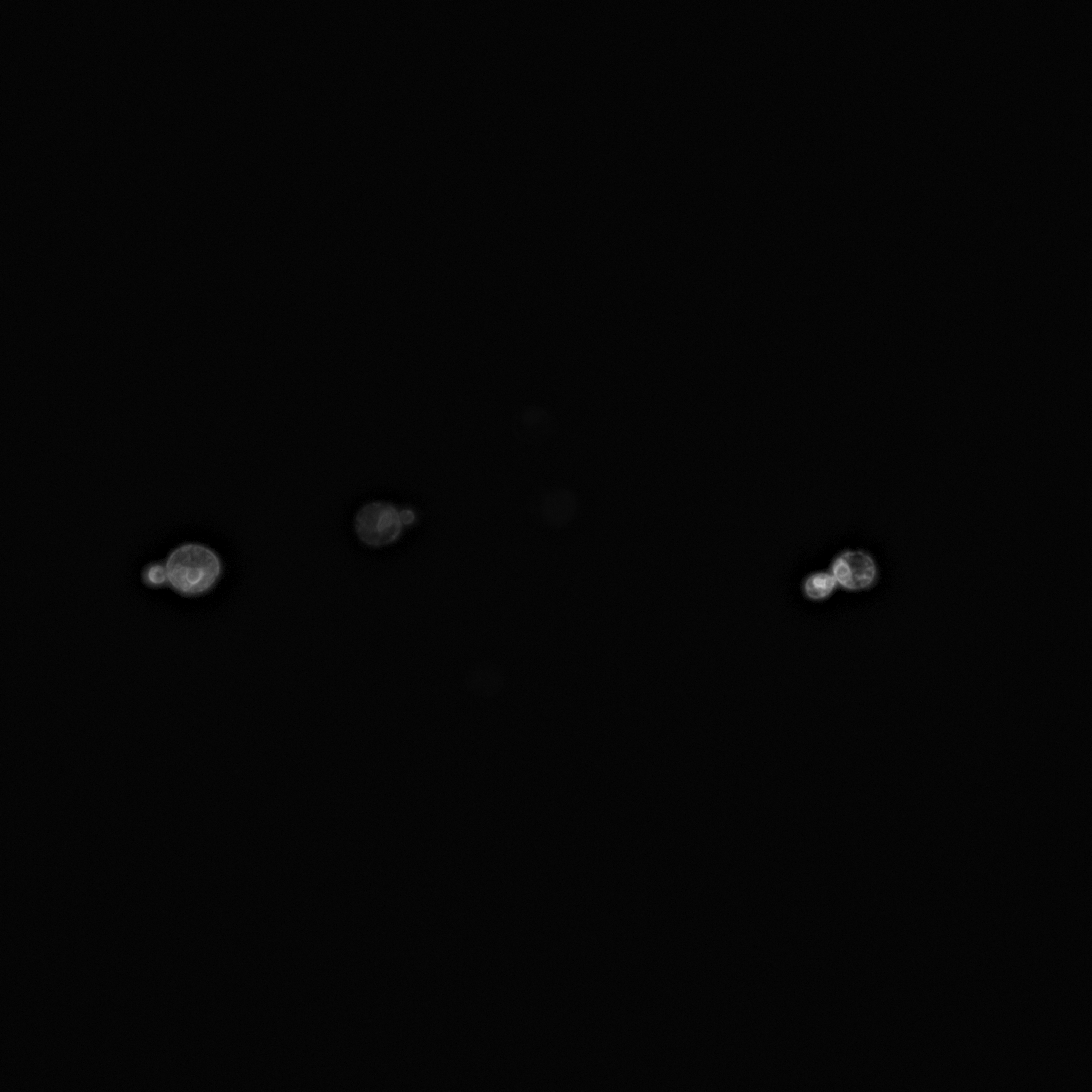

Supplement: Supplementary file 15 — Figure EV1-5 and Appendix S2 Source Data [file 44319_2024_113_MOESM15_ESM.zip › Koch_SourceData_FigEVall/EV4/EV4B/dtom70_MDM34_Scarlet.tif]

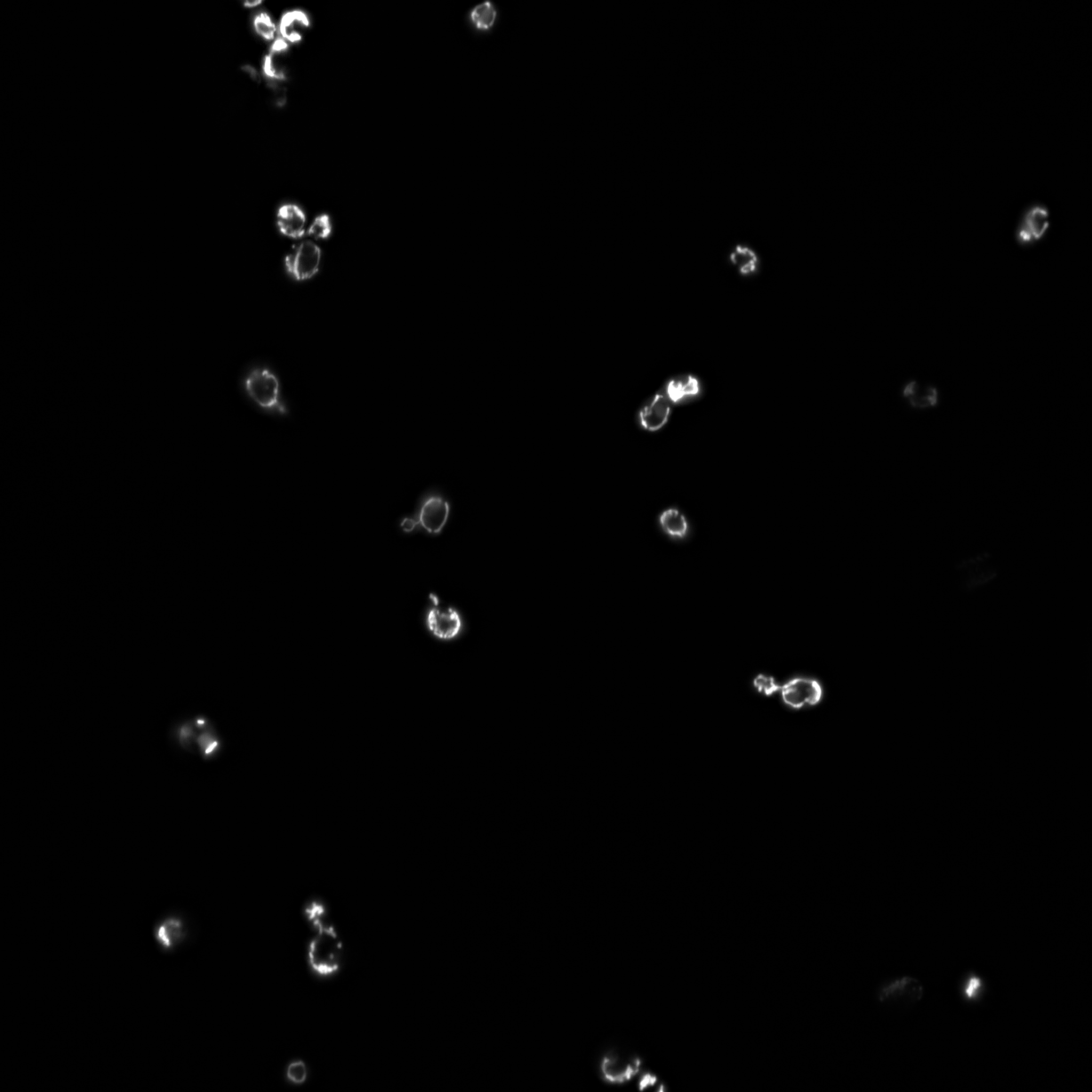

Supplement: Supplementary file 15 — Figure EV1-5 and Appendix S2 Source Data [file 44319_2024_113_MOESM15_ESM.zip › Koch_SourceData_FigEVall/EV4/EV4B/dtom70_NeonGreen.tif]

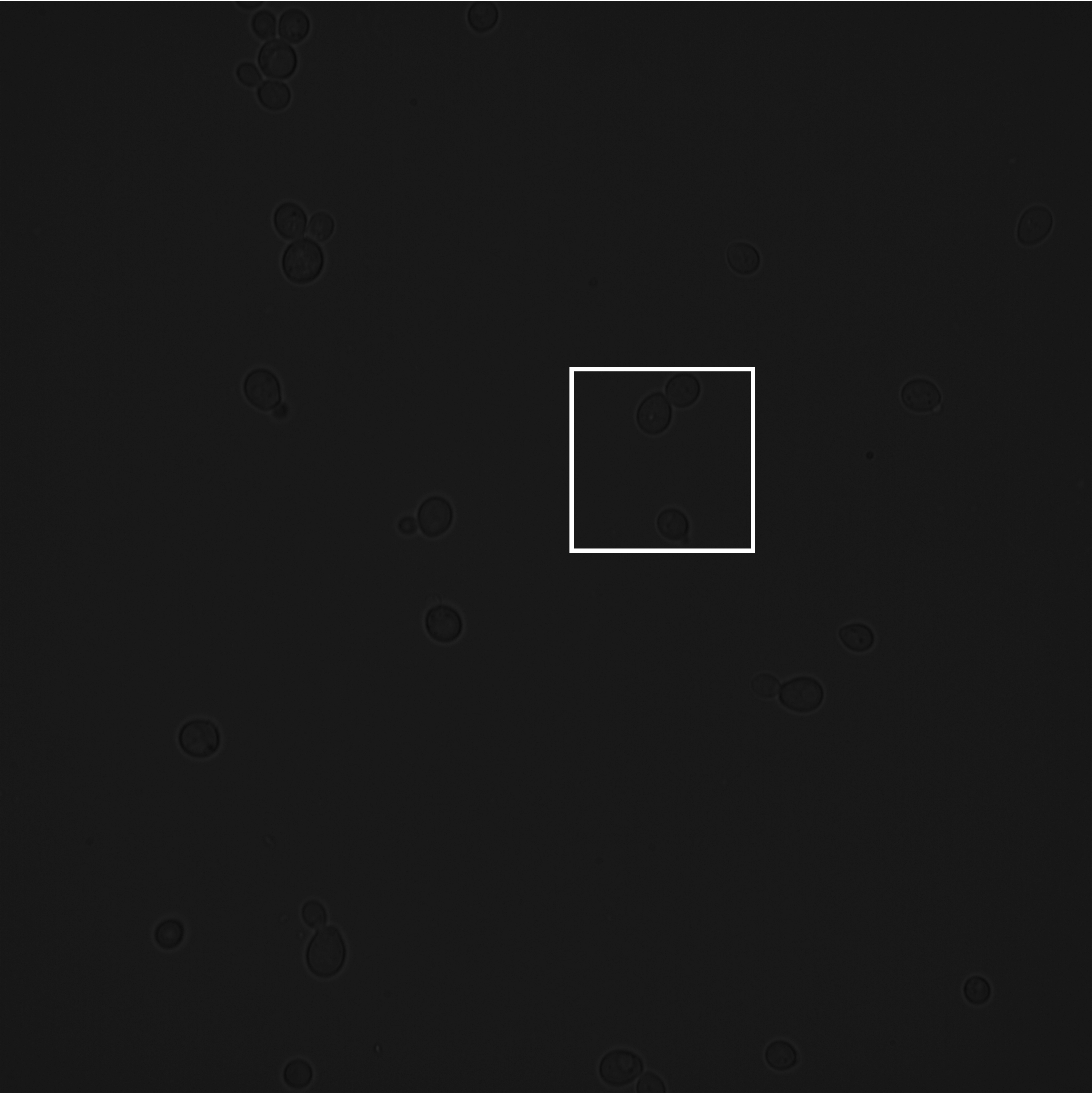

Supplement: Supplementary file 15 — Figure EV1-5 and Appendix S2 Source Data [file 44319_2024_113_MOESM15_ESM.zip › Koch_SourceData_FigEVall/EV4/EV4B/dtom70_refrenceimage.pdf]

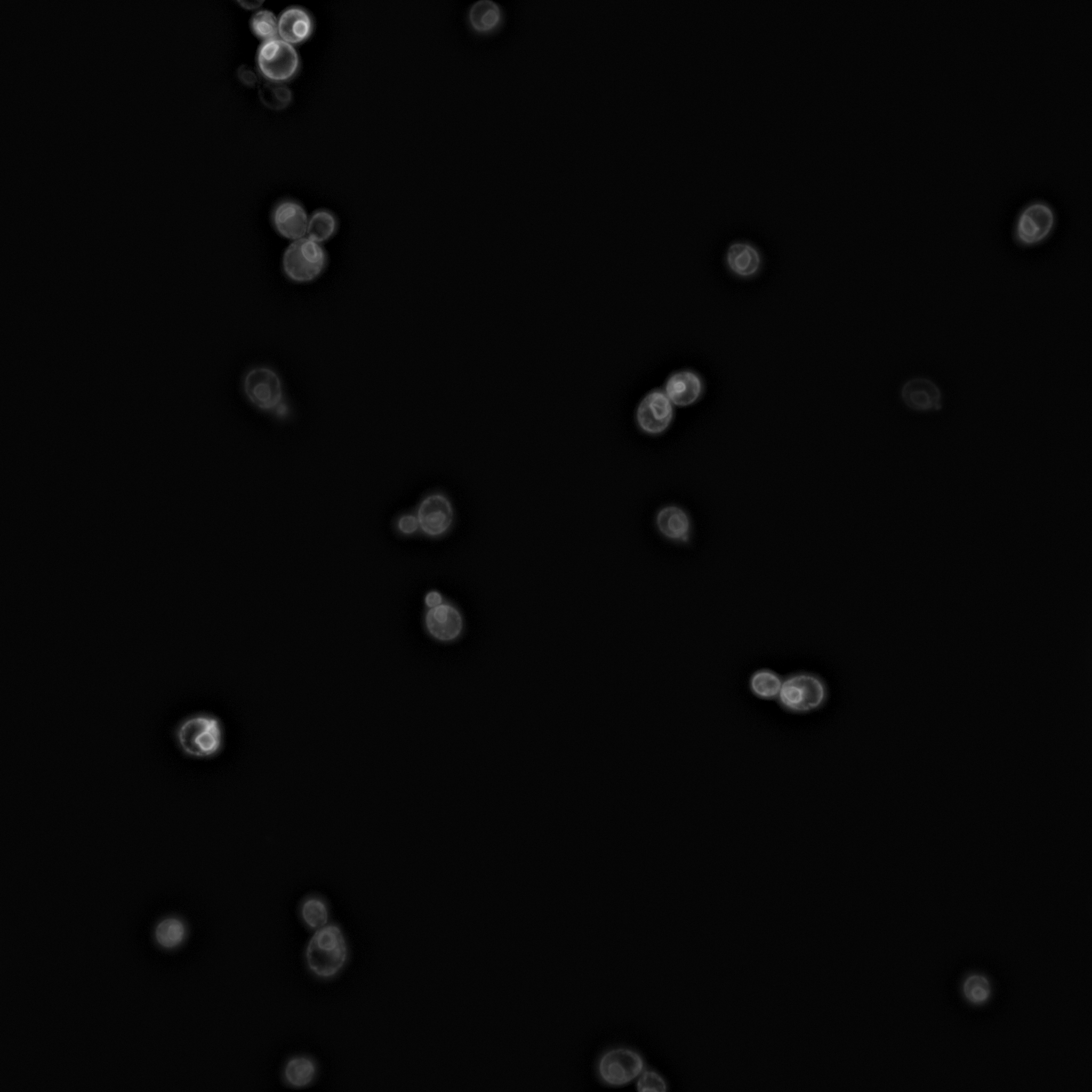

Supplement: Supplementary file 15 — Figure EV1-5 and Appendix S2 Source Data [file 44319_2024_113_MOESM15_ESM.zip › Koch_SourceData_FigEVall/EV4/EV4B/dtom70_Scarlet.tif]

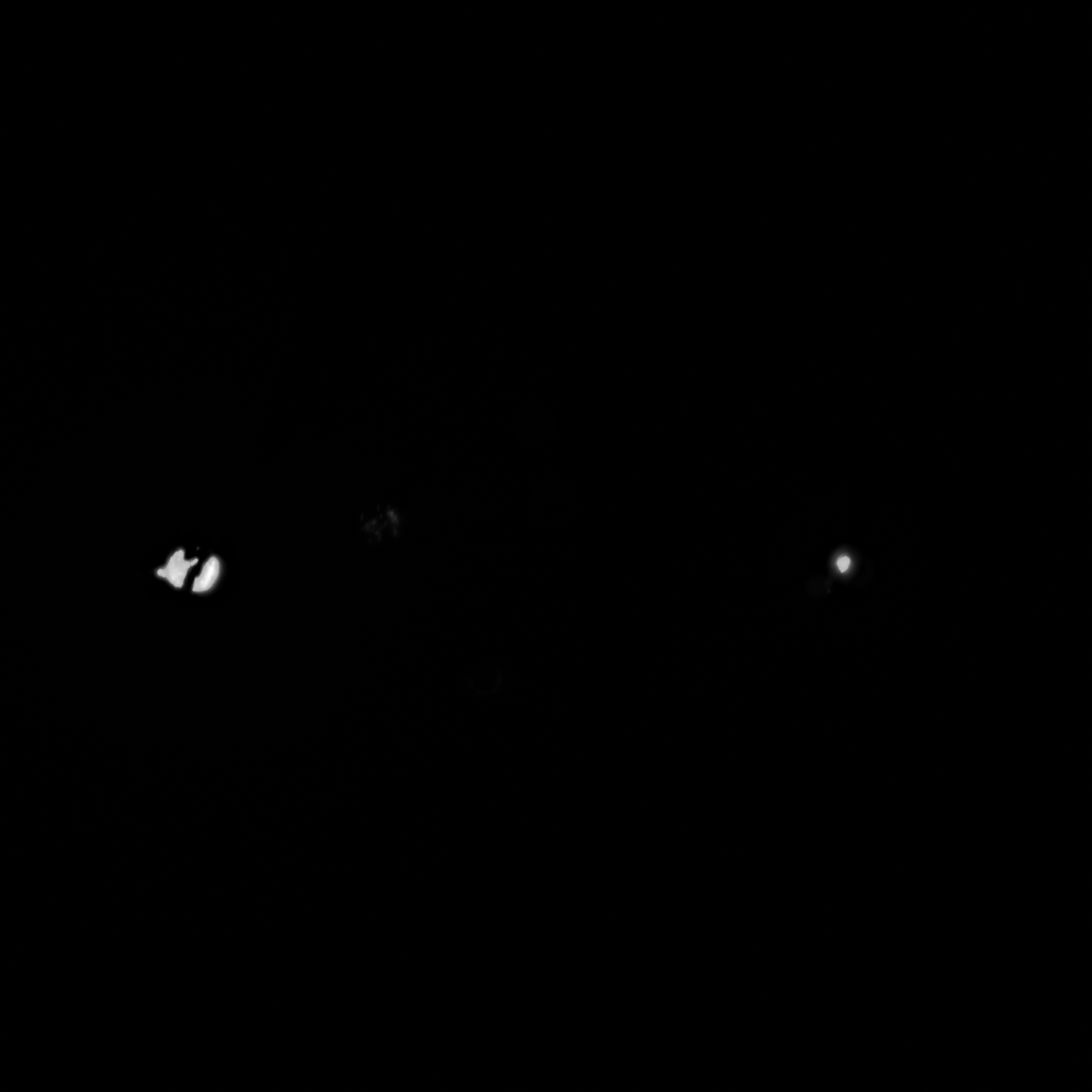

Supplement: Supplementary file 15 — Figure EV1-5 and Appendix S2 Source Data [file 44319_2024_113_MOESM15_ESM.zip › Koch_SourceData_FigEVall/EV4/EV4B/dtom_MDM34_NeonGreen.tif]

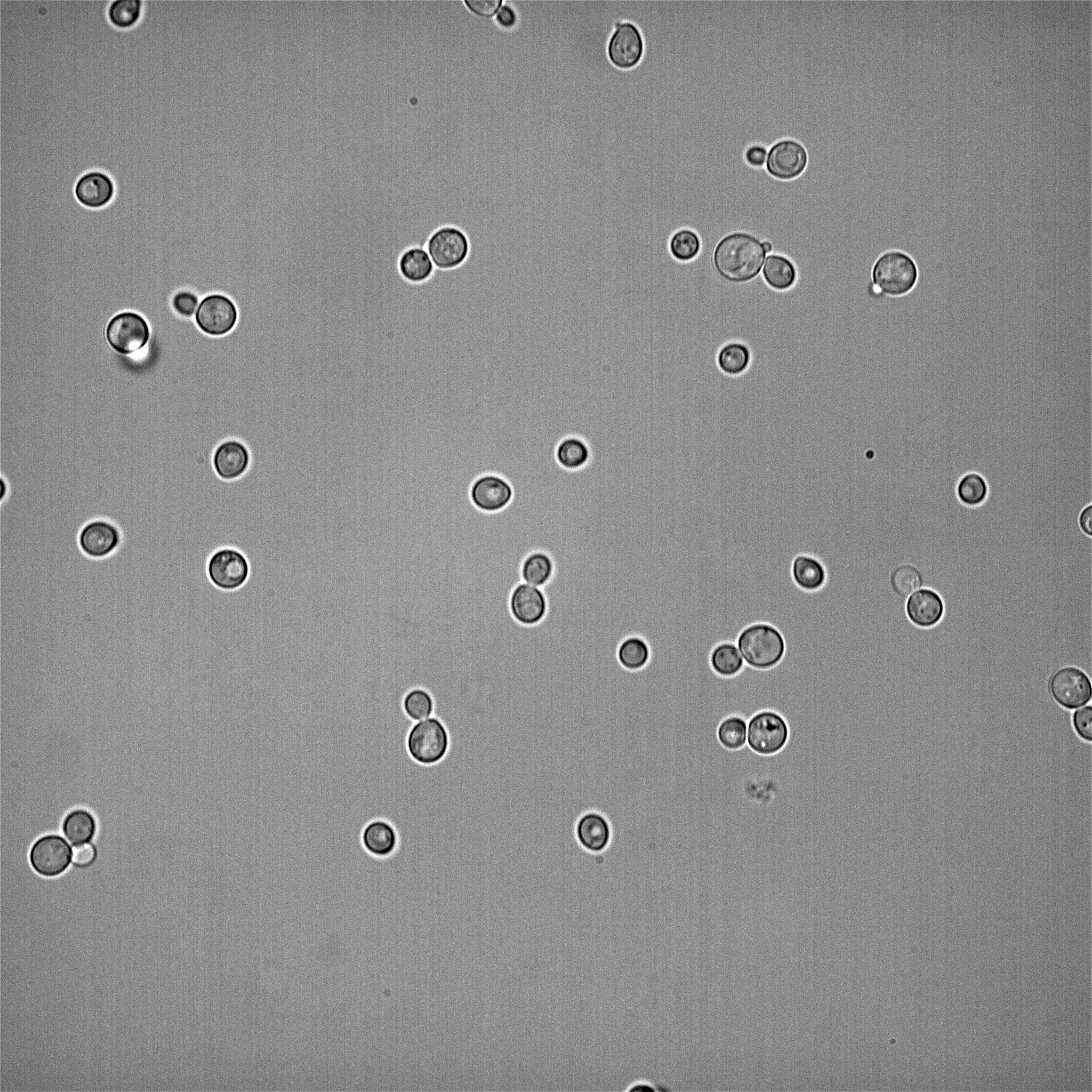

Supplement: Supplementary file 15 — Figure EV1-5 and Appendix S2 Source Data [file 44319_2024_113_MOESM15_ESM.zip › Koch_SourceData_FigEVall/EV4/EV4B/MDM34_BF.tif]

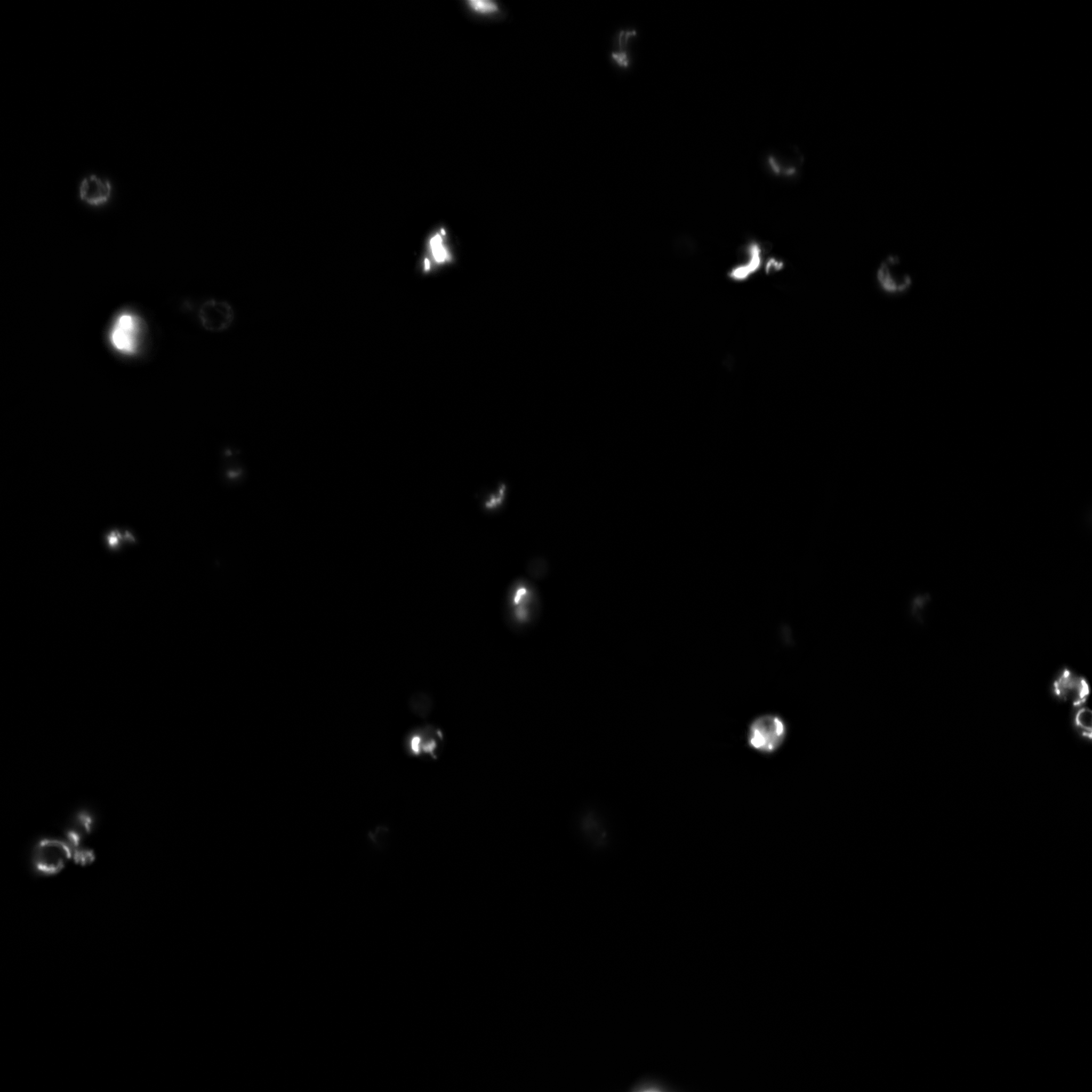

Supplement: Supplementary file 15 — Figure EV1-5 and Appendix S2 Source Data [file 44319_2024_113_MOESM15_ESM.zip › Koch_SourceData_FigEVall/EV4/EV4B/MDM34_NeonGreen.tif]

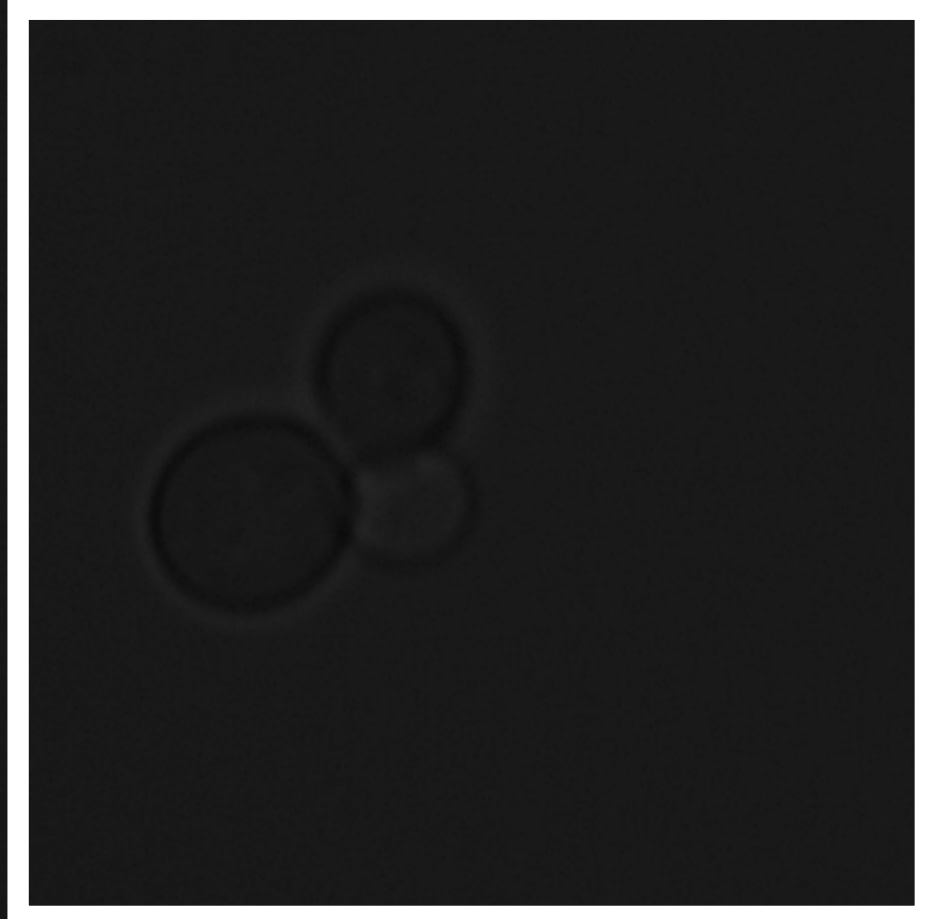

Supplement: Supplementary file 15 — Figure EV1-5 and Appendix S2 Source Data [file 44319_2024_113_MOESM15_ESM.zip › Koch_SourceData_FigEVall/EV4/EV4B/MDM34_refrenceimage.pdf]

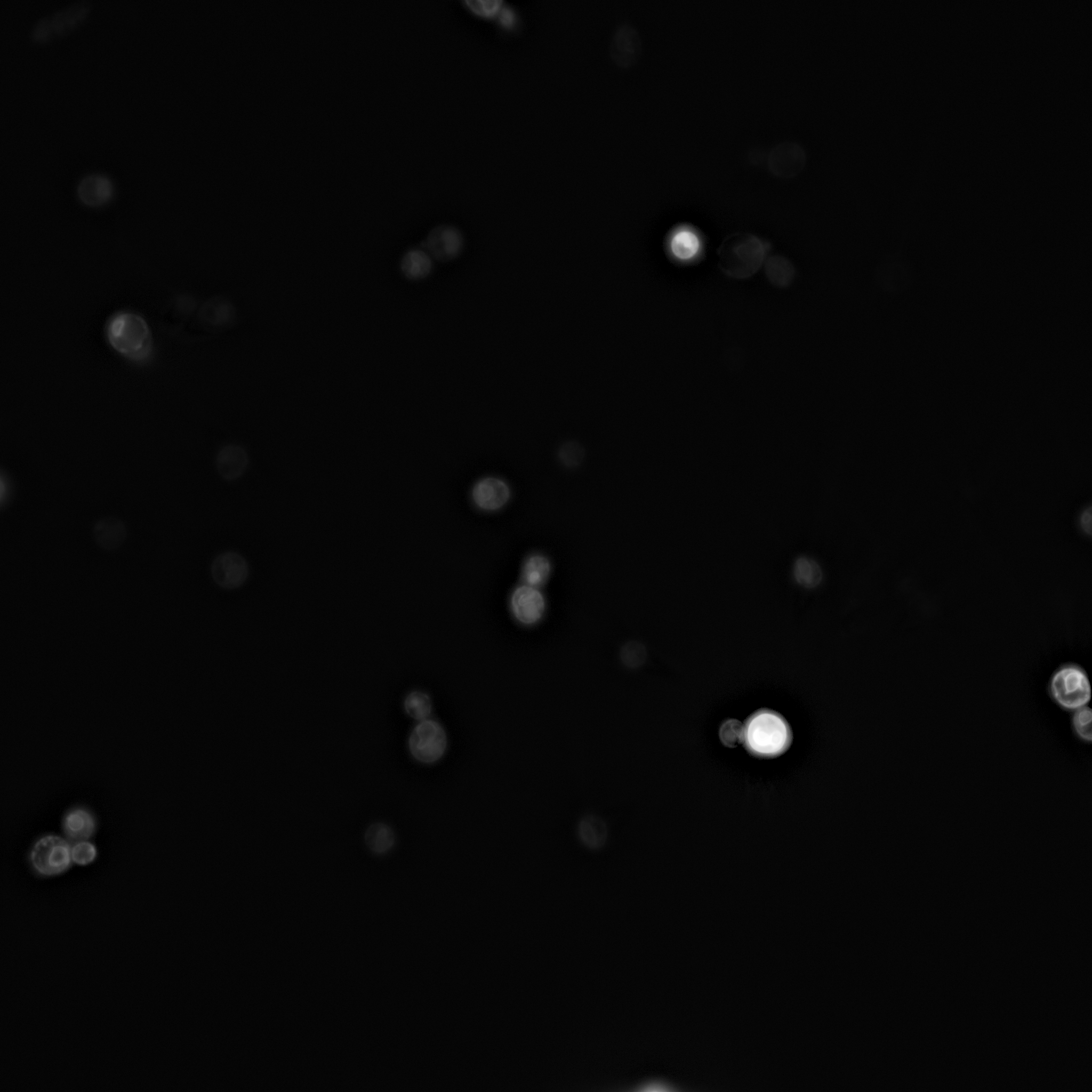

Supplement: Supplementary file 15 — Figure EV1-5 and Appendix S2 Source Data [file 44319_2024_113_MOESM15_ESM.zip › Koch_SourceData_FigEVall/EV4/EV4B/MDM34_Scarlet.tif]

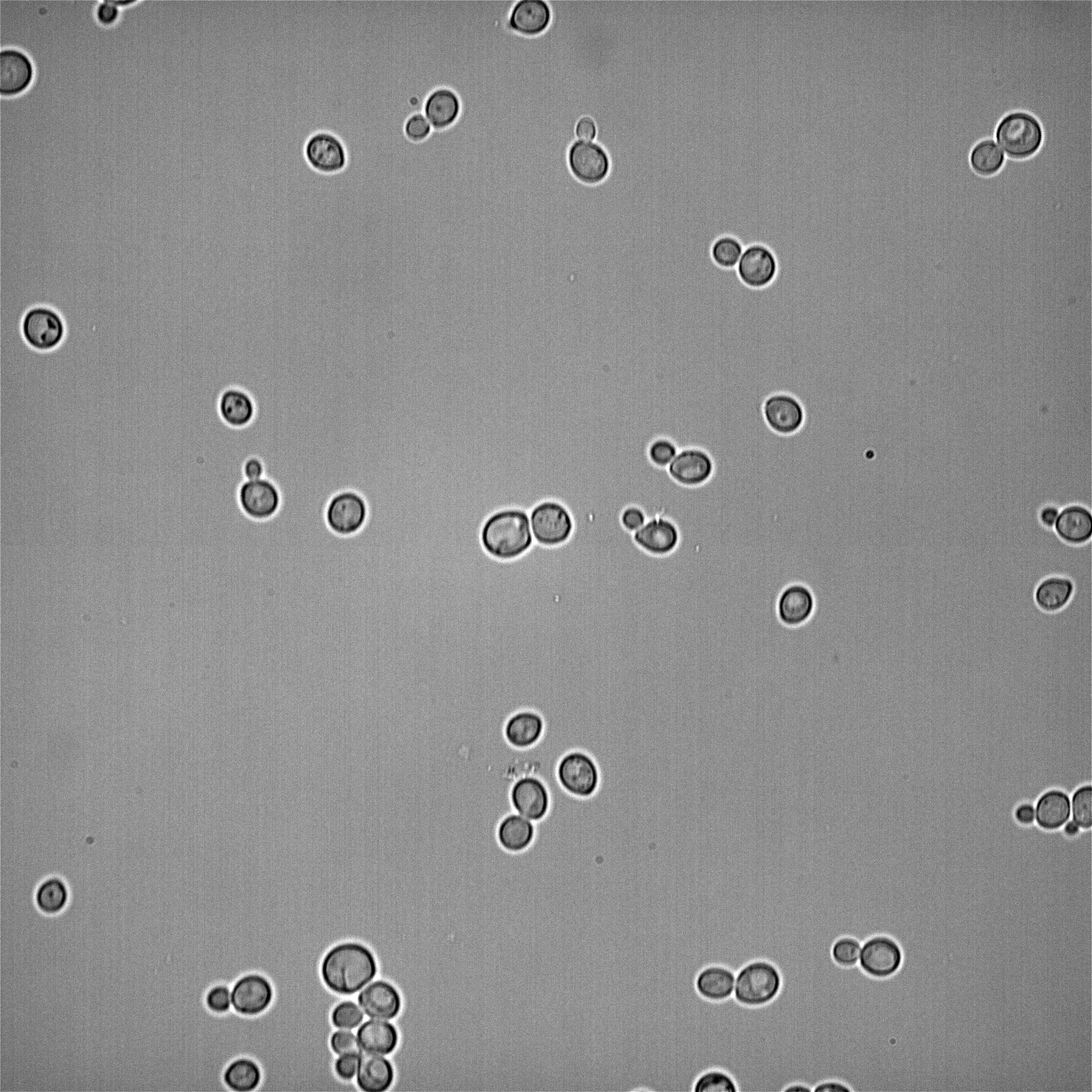

Supplement: Supplementary file 15 — Figure EV1-5 and Appendix S2 Source Data [file 44319_2024_113_MOESM15_ESM.zip › Koch_SourceData_FigEVall/EV4/EV4B/WT_BF.tif]

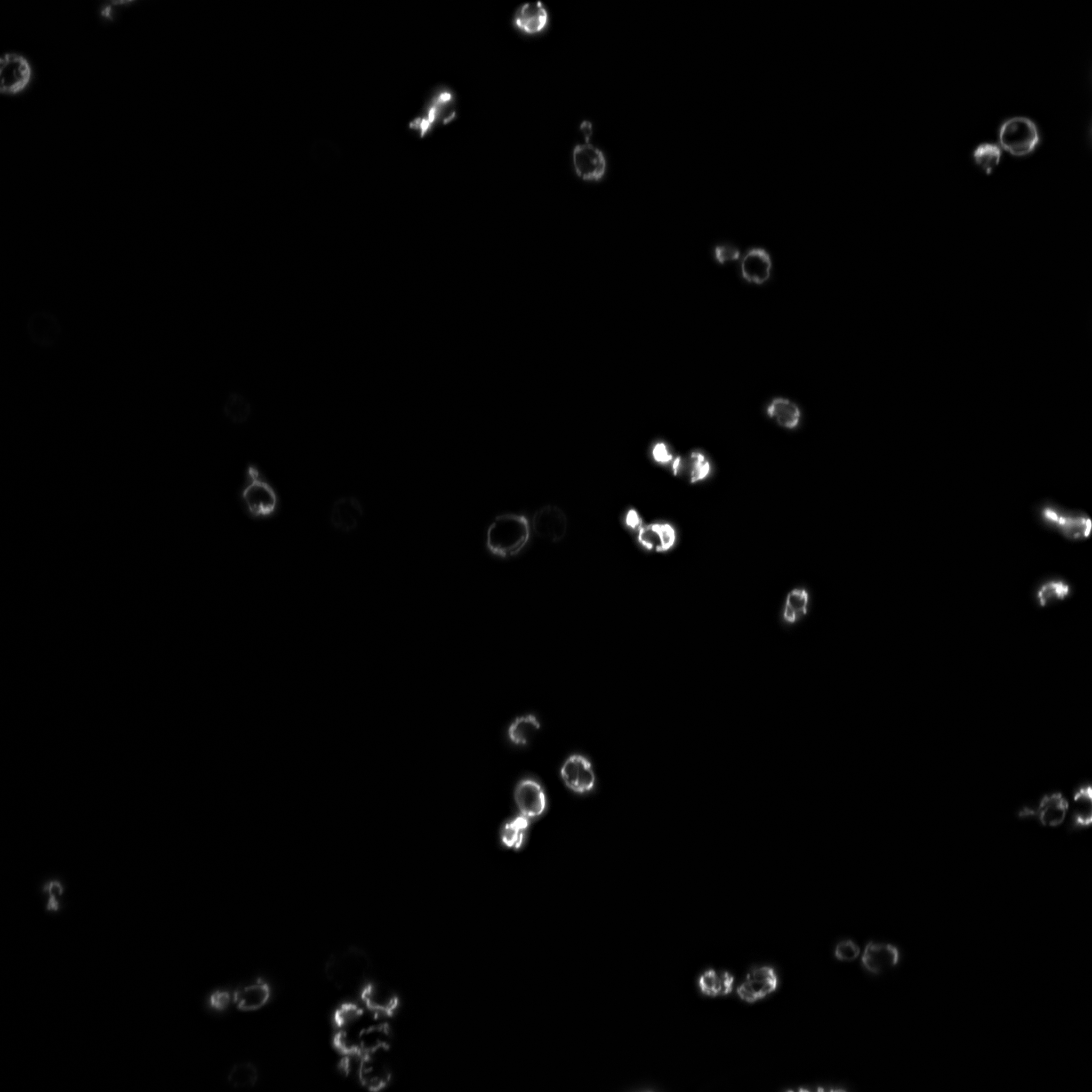

Supplement: Supplementary file 15 — Figure EV1-5 and Appendix S2 Source Data [file 44319_2024_113_MOESM15_ESM.zip › Koch_SourceData_FigEVall/EV4/EV4B/WT_NeonGreen.tif]

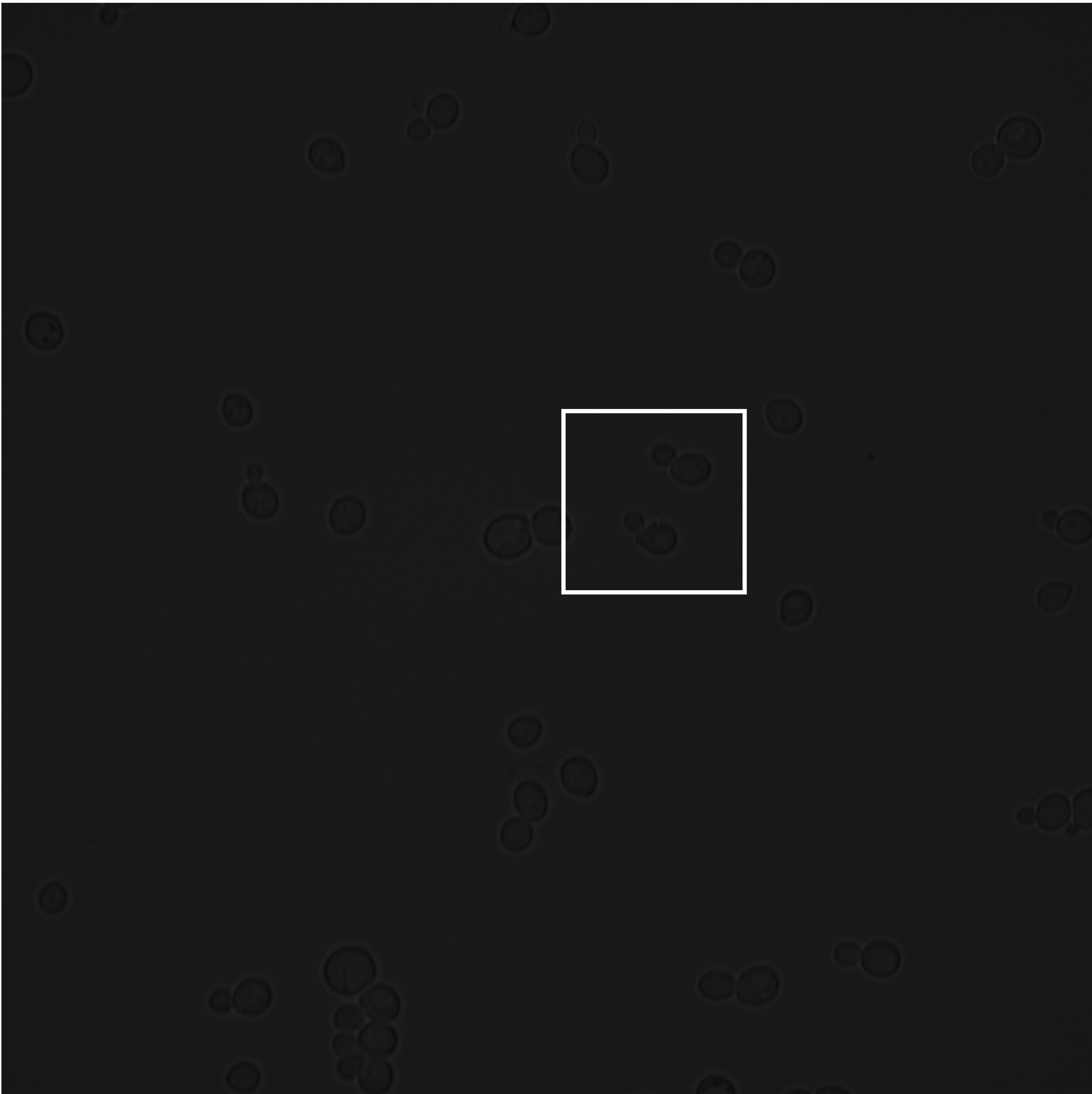

Supplement: Supplementary file 15 — Figure EV1-5 and Appendix S2 Source Data [file 44319_2024_113_MOESM15_ESM.zip › Koch_SourceData_FigEVall/EV4/EV4B/WT_refrenceimage.pdf]

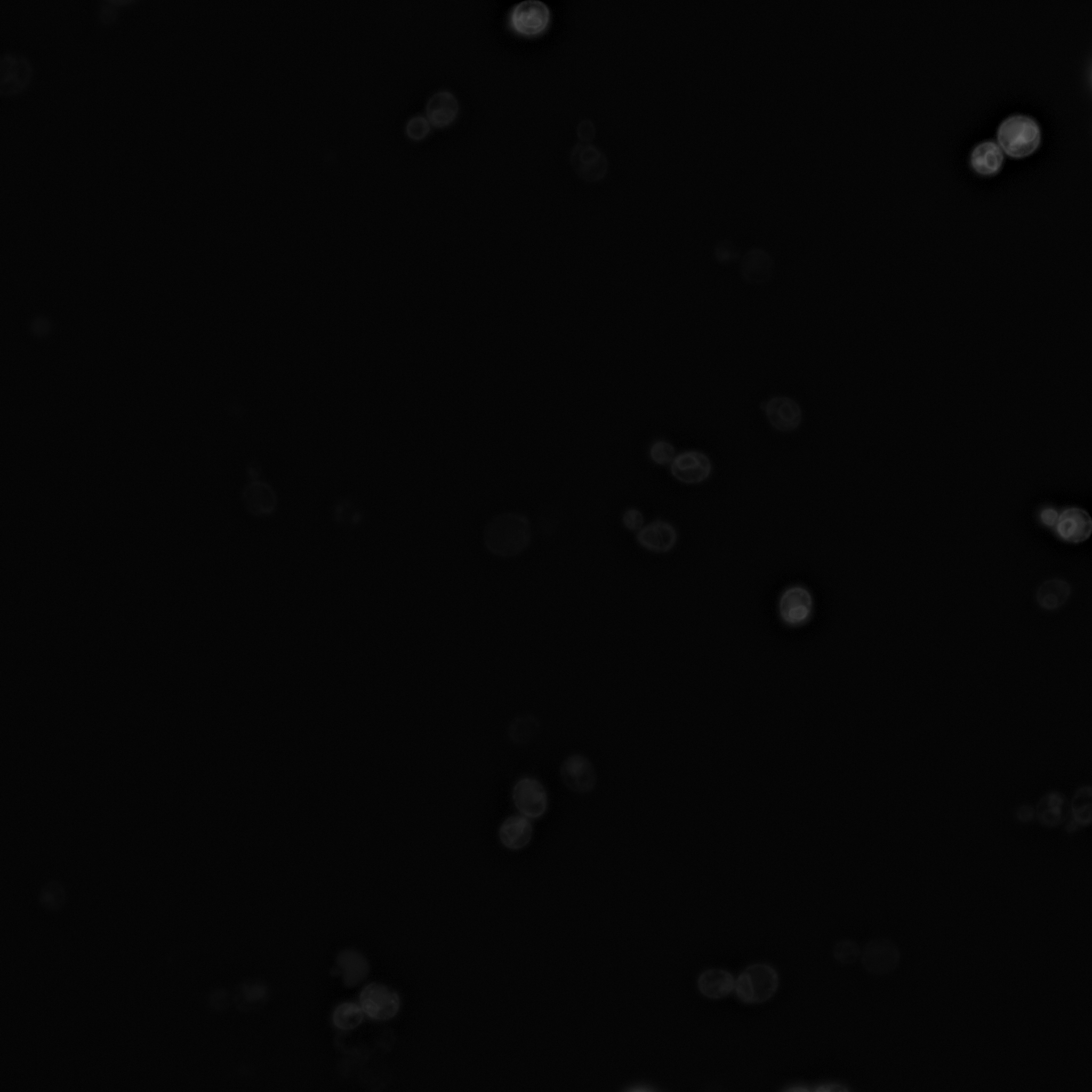

Supplement: Supplementary file 15 — Figure EV1-5 and Appendix S2 Source Data [file 44319_2024_113_MOESM15_ESM.zip › Koch_SourceData_FigEVall/EV4/EV4B/WT_Scarlet.tif]
