# Supplementary material for: The ER-SURF pathway uses ER-mitochondria contact sites for protein targeting to mitochondria
Source: EMBO Rep. 2024 Apr 2;25(4):24. doi: 10.1038/s44319-024-00113-w (PMC11014988; doi:10.1038/s44319-024-00113-w)

Figure 6A Cox5A_ΔTM

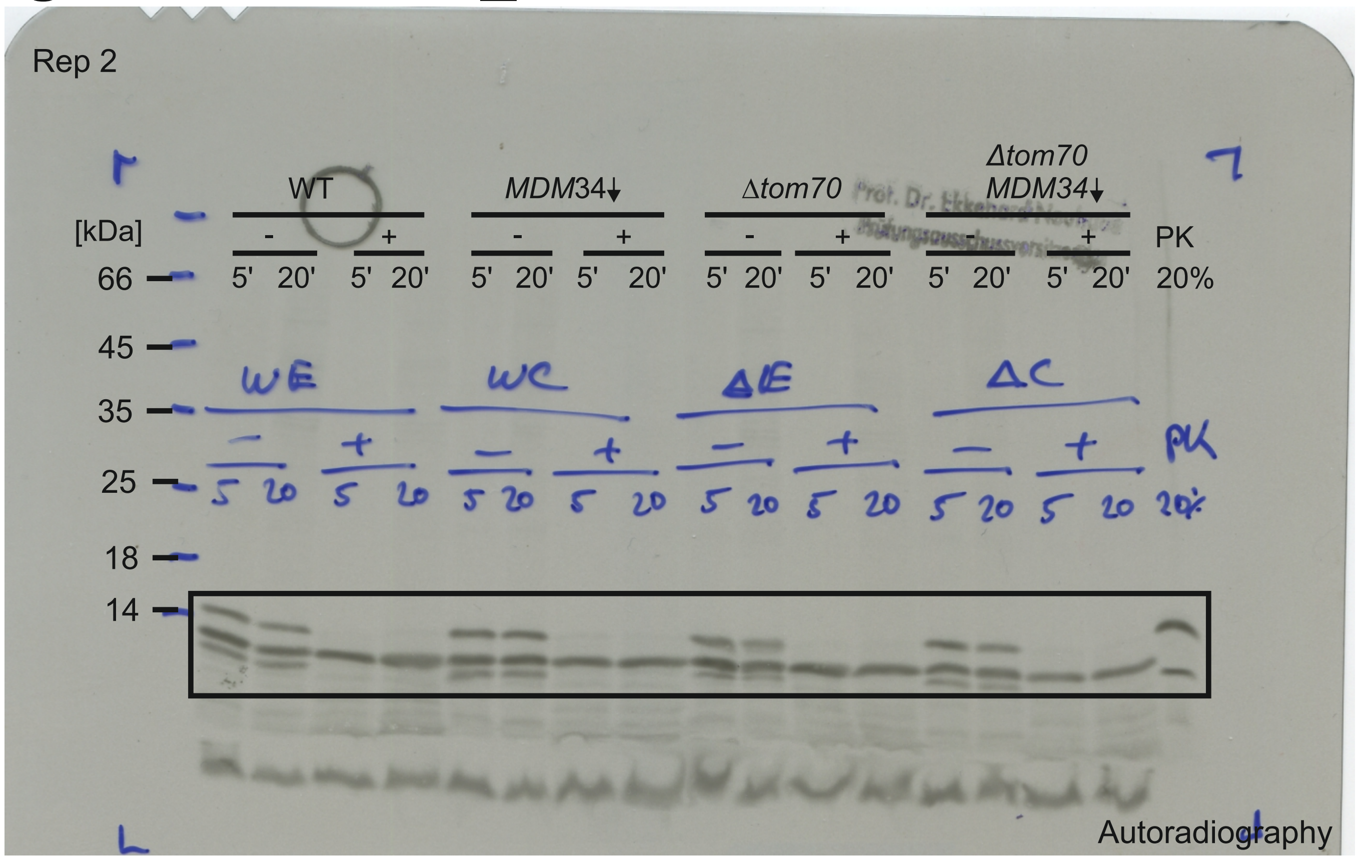

For quantification:

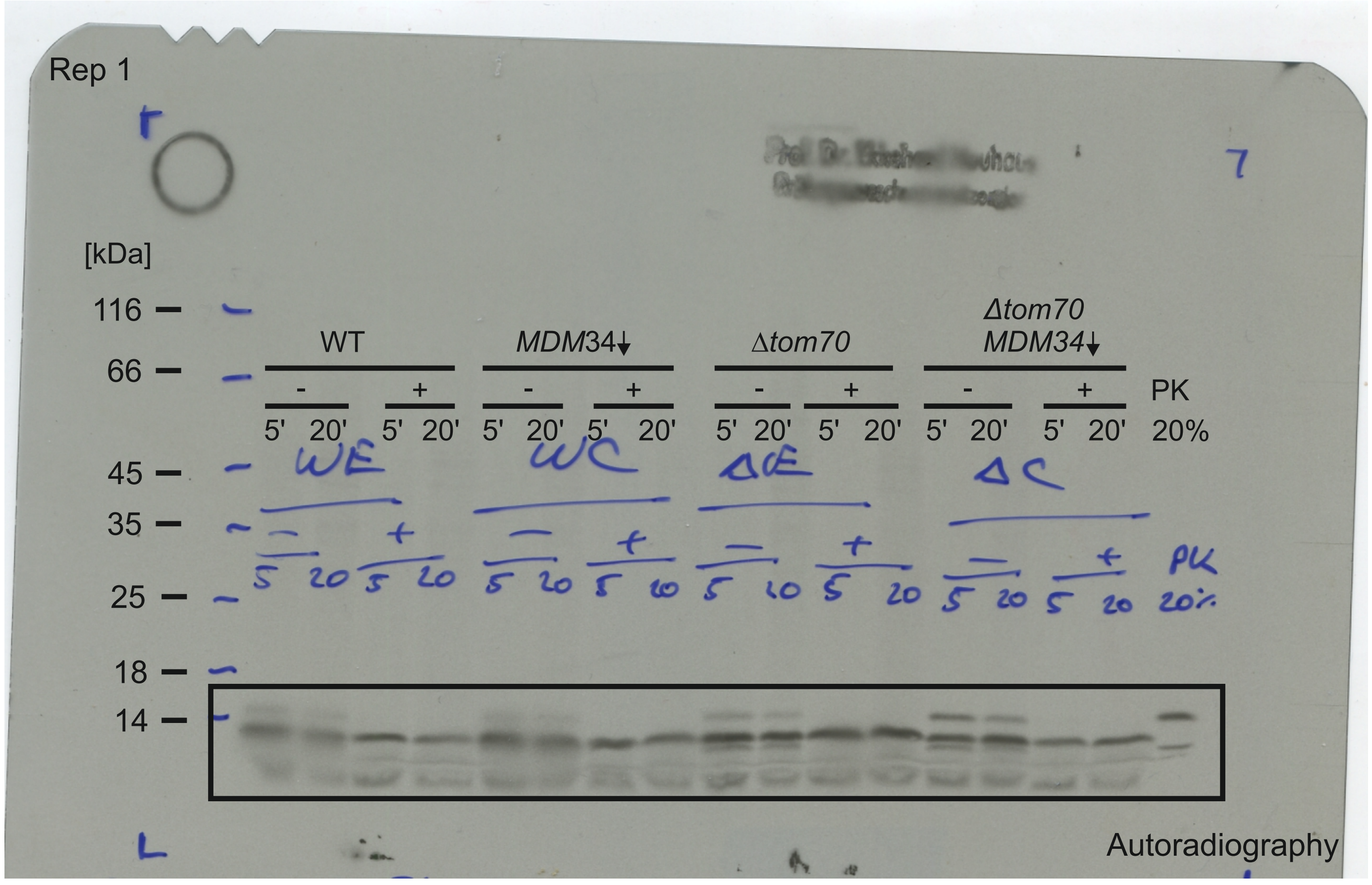

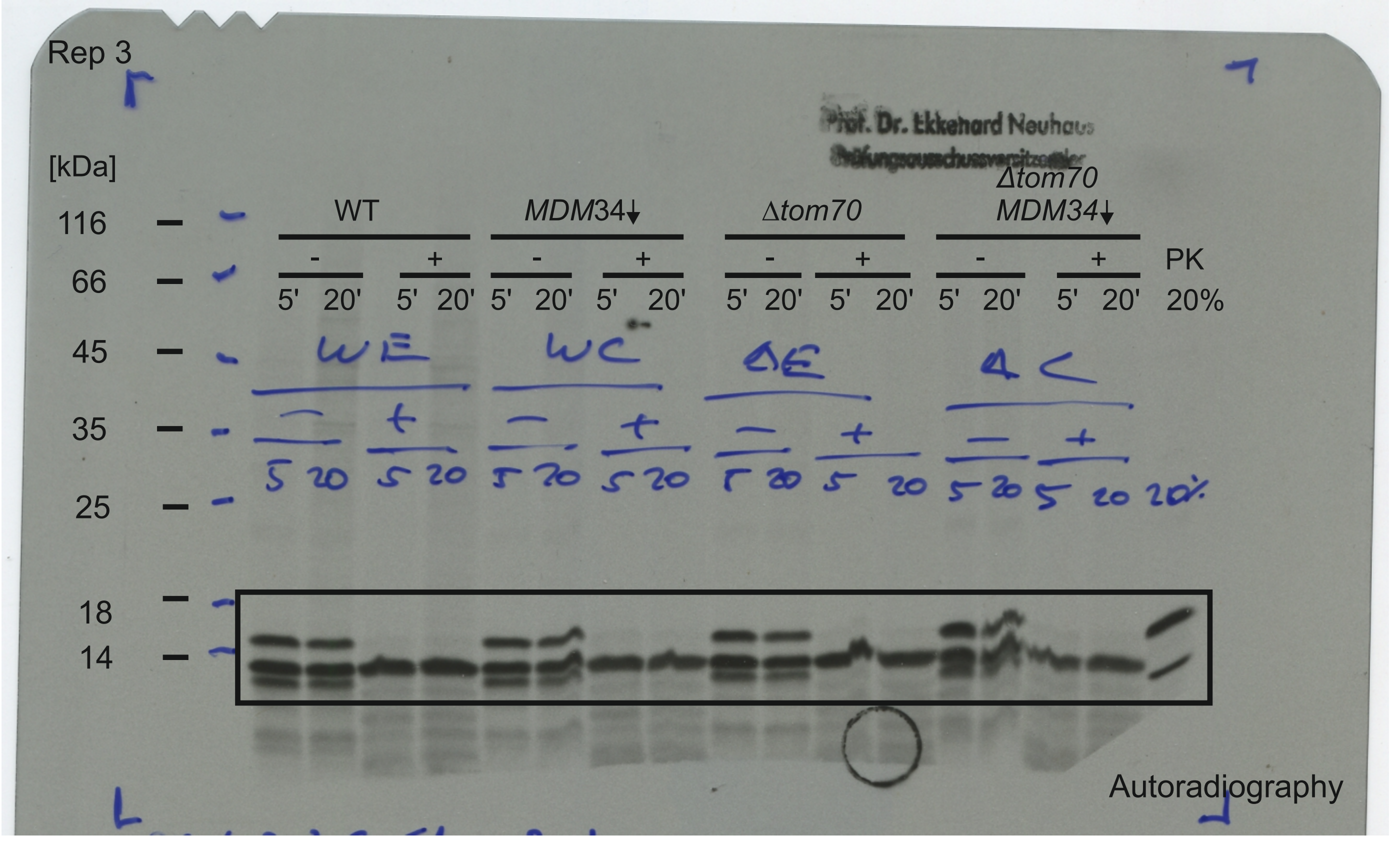

Supplement: Supplementary file 14 — Source data Fig. 6 [file 44319_2024_113_MOESM14_ESM.zip › Koch_SourceData_Fig6/6A-B/6A_Cox5A_DTM.pdf]
