# Supplementary material for: The ER-SURF pathway uses ER-mitochondria contact sites for protein targeting to mitochondria
Source: EMBO Rep. 2024 Apr 2;25(4):24. doi: 10.1038/s44319-024-00113-w (PMC11014988; doi:10.1038/s44319-024-00113-w)
Supplement: Supplementary file 17 — Expanded View Figures [file 44319_2024_113_MOESM17_ESM.pdf]

Expanded View Figures

Figure EV1. The ERMES contact site is critical for protein targeting via ER-SURF.

(**A**, **B**) The protein levels of Djp1 and of control proteins in wild type, Δ*djp1* and Δ*mdm34* strains were analyzed by Western blotting and quantified. Panel **B** shows the mean values and standard deviations of three biological replicates. Statistical difference was calculated with a student's *t*-test. Statistical significance was assigned as follows: ***p value <0.005. (**C**, **D**) Schematic representation of the Oxa1-Ura3 reporter assay. Normal import of this reporter leads to the depletion of Ura3 from the cytosol and uracil auxotrophy. Impaired import of this reporter restores uracil prototrophy and allows for growth on plates lacking uracil. Cells of the indicated strains were grown to log phase in glucose medium before tenfold serial dilutions were dropped onto plates containing or lacking uracil. (**E**–**G**) Radiolabeled proteins were synthesized in reticulocyte lysate in the presence of ^{35}S-methionine and incubated with semi-intact cells derived from the indicated strains. After 5 or 20 min, the cells were isolated, treated without or with proteinase K (PK) for 30 min on ice, and subjected to SDS-PAGE and autoradiography. 20% of the radioactive protein used per import reaction (time point) was loaded for comparison. Precursor and mature forms are indicated as pre and m. Source data are available online for this figure.

© The Author(s)

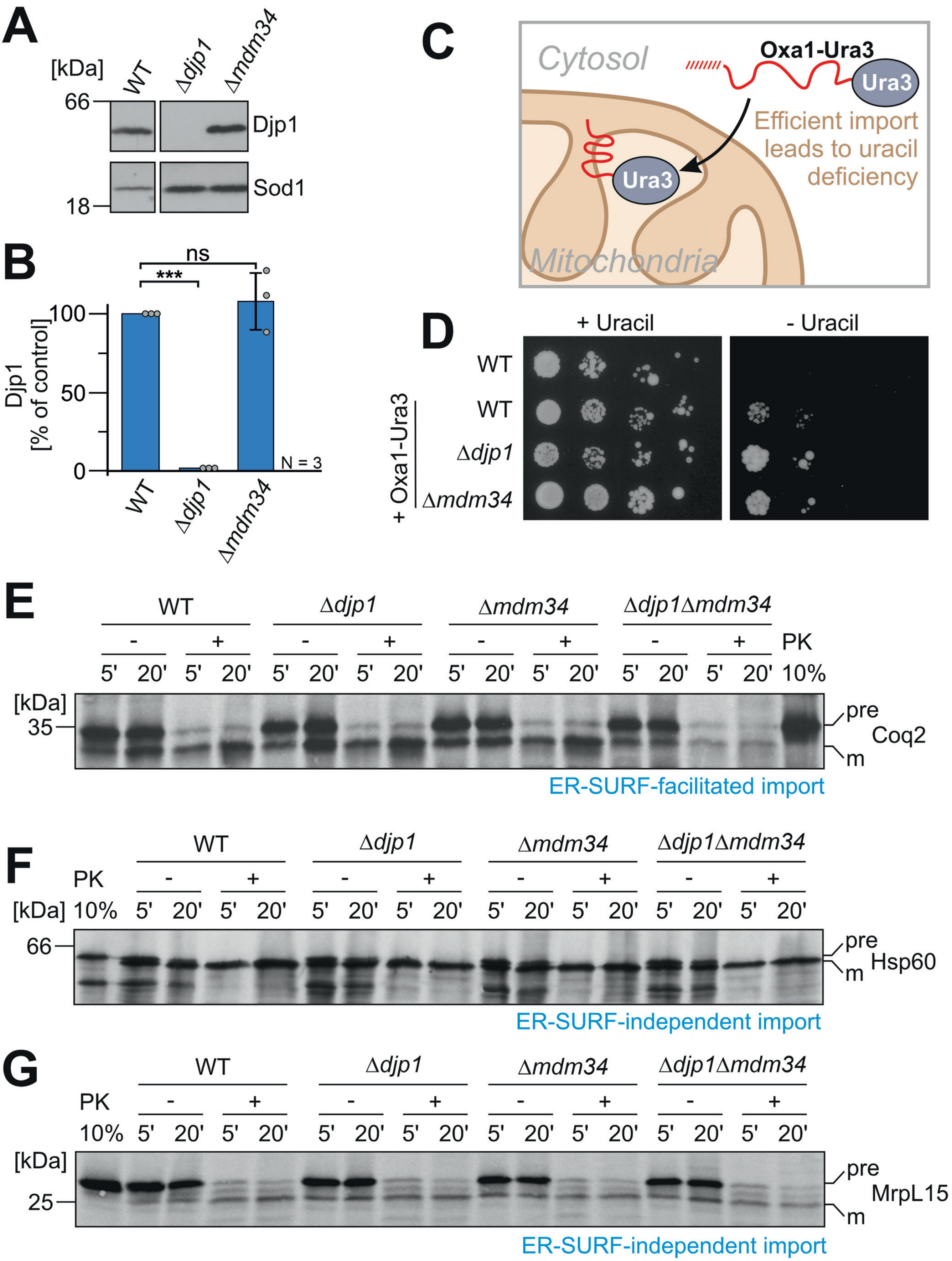
A
[kDa]
WT
Δdjp1
Δmdm34
66
18
Djp1
Sod1
B
ns
***
Djp1
[% of control]
100
50
0
N = 3
WT
Δdjp1
Δmdm34
C
Cytosol
Oxa1-Ura3
Ura3
Efficient import leads to uracil deficiency
Mitochondria
D
+ Uracil
- Uracil
WT
+ Oxa1-Ura3
Δdjp1
Δmdm34
E
WT
Δdjp1
Δmdm34
Δdjp1Δmdm34
PK
10%
5'
20'
[kDa]
35
pre
Coq2
m
ER-SURF-facilitated import
F
Hsp60
66
ER-SURF-independent import
G
25
MrpL15
ER-SURF-independent import

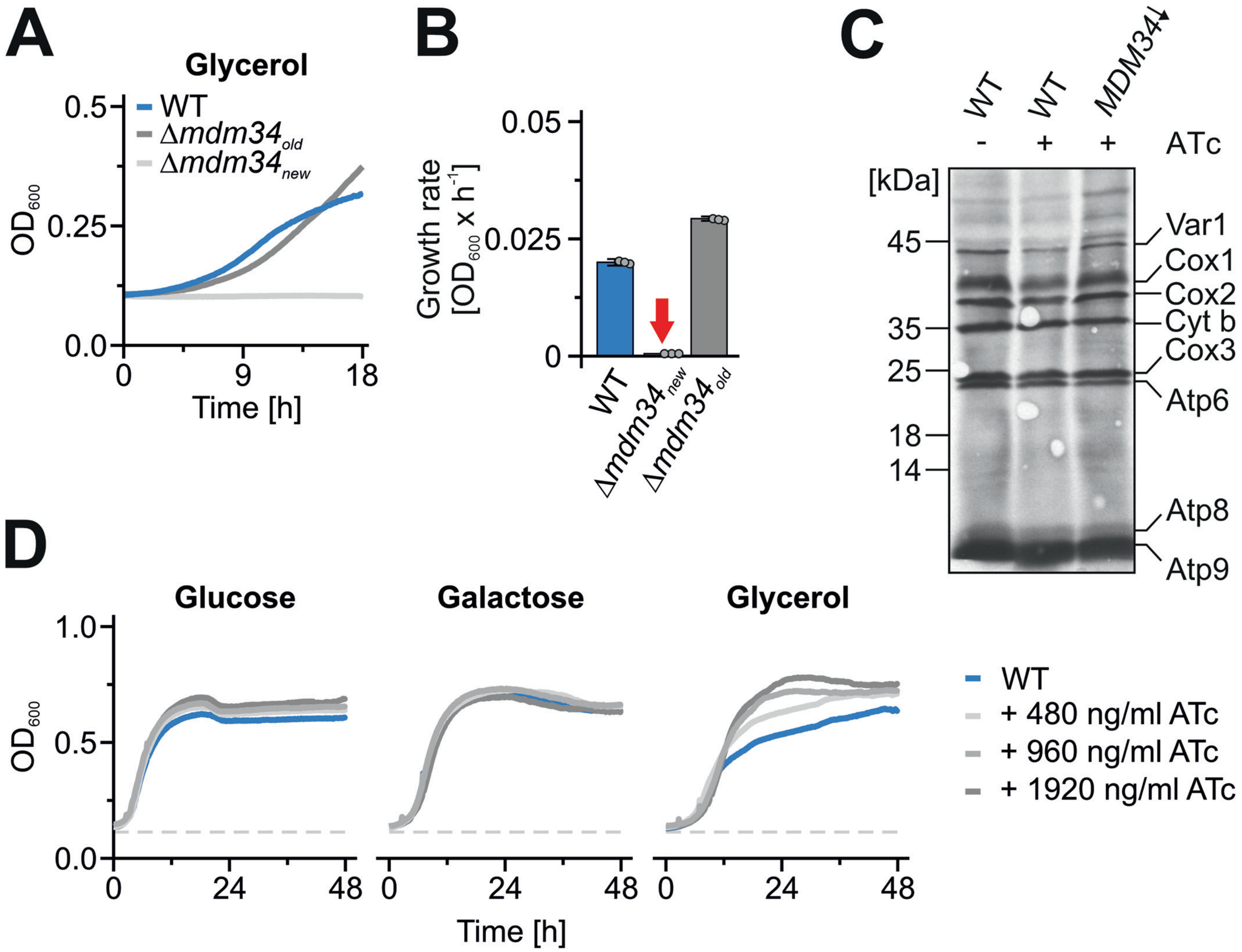

Figure EV2. The deletion of ERMES contact sites lead to transient growth defects.

(**A**, **B**) The indicated strains were grown in galactose medium to log phase and used to inoculate cultures in glycerol media. Cells were grown at 30 °C under constant agitation. Cell growth was continuously monitored. Growth rates were determined by calculation of the slope of the curve in the log phase. The graphs show the mean values of three technical replicates. (**C**) Indicated strains were grown in galactose medium and expression of Mdm34 was suppressed by the addition of ATc for 16 h. Mitochondrial translation products were radiolabeled for 15 min with ^{35}S-methionine in the presence of cycloheximide to inhibit cytosolic translation. Radiolabeled proteins were visualized by SDS-PAGE and autoradiography. (**D**) The indicated strains were grown in galactose medium to log phase and used to inoculate cultures in either glucose, galactose, or glycerol media containing varying concentrations of ATc. Cells were grown at 30 °C under constant agitation. Cell growth was continuously monitored. Source data are available online for this figure.

 © The Author(s)

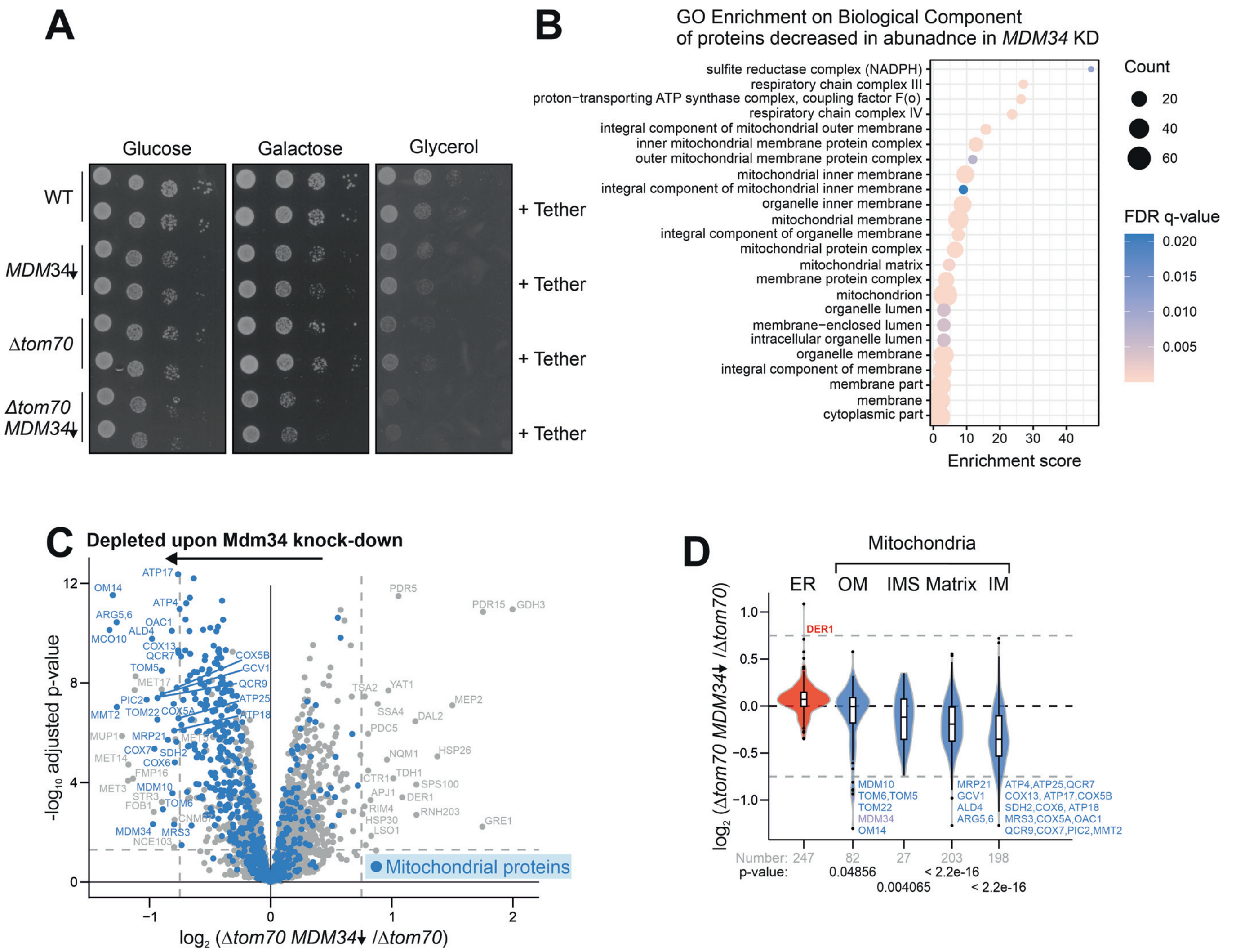

Figure EV3. Loss of ERMES and Tom70 leaves a strong footprint on the mitochondrial proteome.

(**A**) Cells of the indicated strains were grown to log phase in galactose medium and depletion of Mdm34 was induced by the addition of 960 ng/ml of ATc before tenfold serial dilutions were dropped onto plates with the indicated carbon sources. (**B**) The whole-cell proteomes of wild type and Mdm34-depleted cells were measured and further analyzed by gene ontology (GO) enrichment. Proteins with smaller than -0.5 $\log_2$ fold change were used as target set and analyzed by using the GOrilla tool (http://cbl-gorilla.cs.technion.ac.il/) with all quantified proteins as background. The top results with a false discovery rate [FDR] <5% are shown. (**C**) Comparison of the proteomes of Δ*tom70* and Mdm34-depleted cells 24 h after ATc addition. Mitochondrial proteins (Morgenstern et al, 2017) were indicated in blue. (**D**) The violin plot shows the ratio of protein abundance (log2-fold enrichment scores) in Δ*tom70* relative to Mdm34-depleted cells. Numbers below the different subclasses represent the number of proteins (*n*) within a given subclass. Boxes represent the data range from the first (Q1) to the third quartile (Q3), with the line in the middle representing the median. The minimum/maximum whisker values were calculated as Q1/Q3 ± 1.5 * interquartile range (IQR). Every data point outside is represented as a potential outlier in the form of a dot. Mitochondrial proteins, particularly those of the inner membrane, are significantly depleted. Statistical difference was calculated with a Kolmogorov–Smirnov test comparing the indicated subpopulations with all other proteins. The *p* values are shown as a measure of statistical significance. Source data are available online for this figure.

© The Author(s)

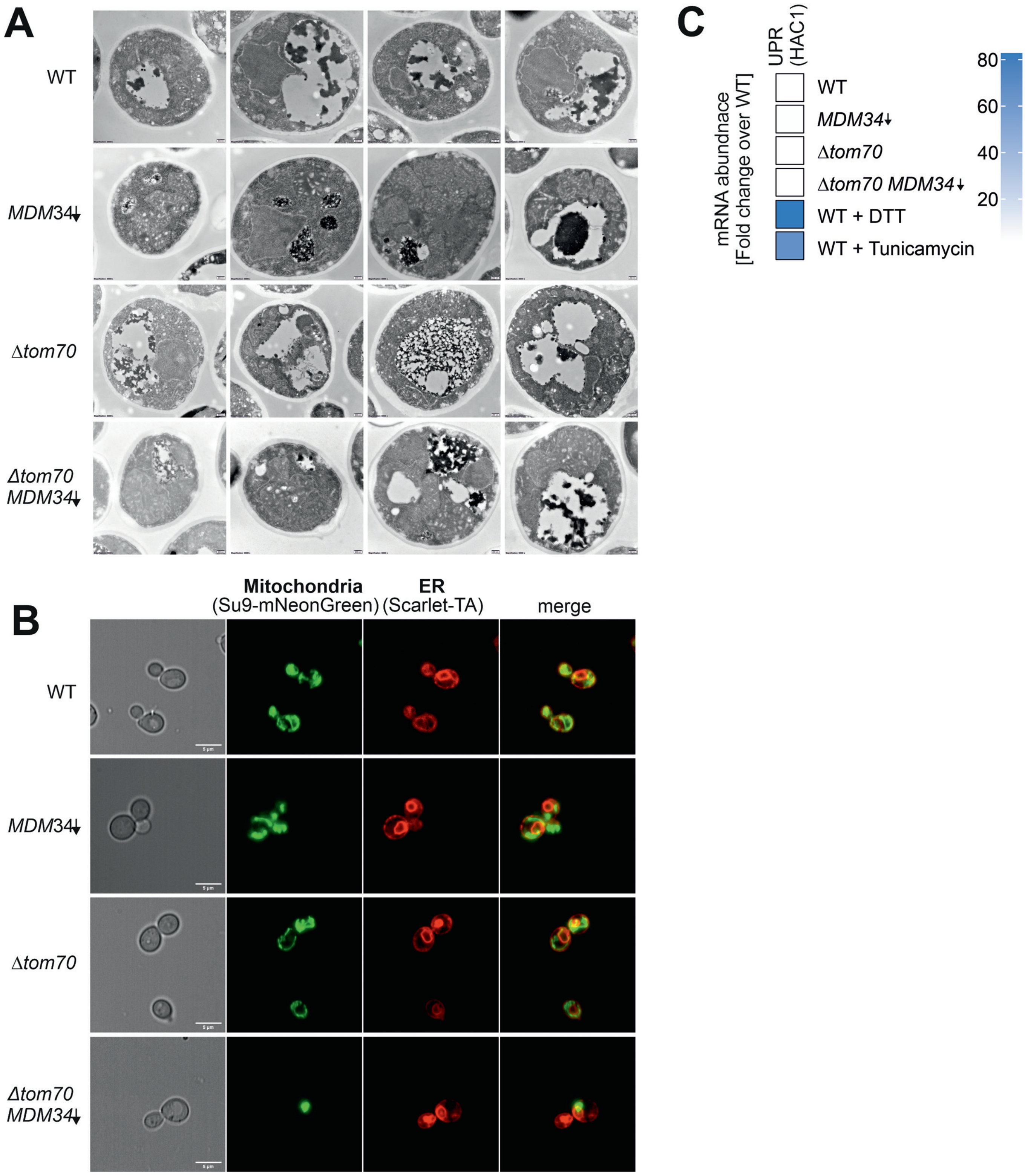
A
WT
MDM34↓
Δtom70
Δtom70 MDM34↓
B
Mitochondria (Su9-mNeonGreen)
ER (Scarlet-TA)
merge
WT
MDM34↓
Δtom70
Δtom70 MDM34↓
C
mRNA abundnace [Fold change over WT]
UPR (HAC1)
WT
MDM34↓
Δtom70
Δtom70 MDM34↓
WT + DTT
WT + Tunicamycin
80
60
40
20

 © The Author(s)

Figure EV4. Loss of ERMES strongly affects mitochondrial morphology.

(**A**) Yeast cells were grown to log phase in galactose medium before 960 ng/ml ATc was added for 16 h. Cells were embedded, cut into thin slices, and visualized by transmission electron microscopy. (**B**) The indicated strains expressing a matrix-targeted mNeonGreen (Su9-mNeonGreen) and an ER-targeted mScarlet (Scarlet-TA) were grown to log phase in galactose medium before 960 ng/ml ATc was added for 16 h. Afterwards, cells were harvested and imaged using a Leica Dmi8 Thunder imager. (**C**) Cells of the indicated strains were grown on galactose in the presence of ATc for 16 h. The induction of the unfolded protein response (UPR) was measured by qPCR based on *HAC1* splicing. Wild-type strains treated for 1 h with either DTT or tunicamycin served as positive controls. Source data are available online for this figure.

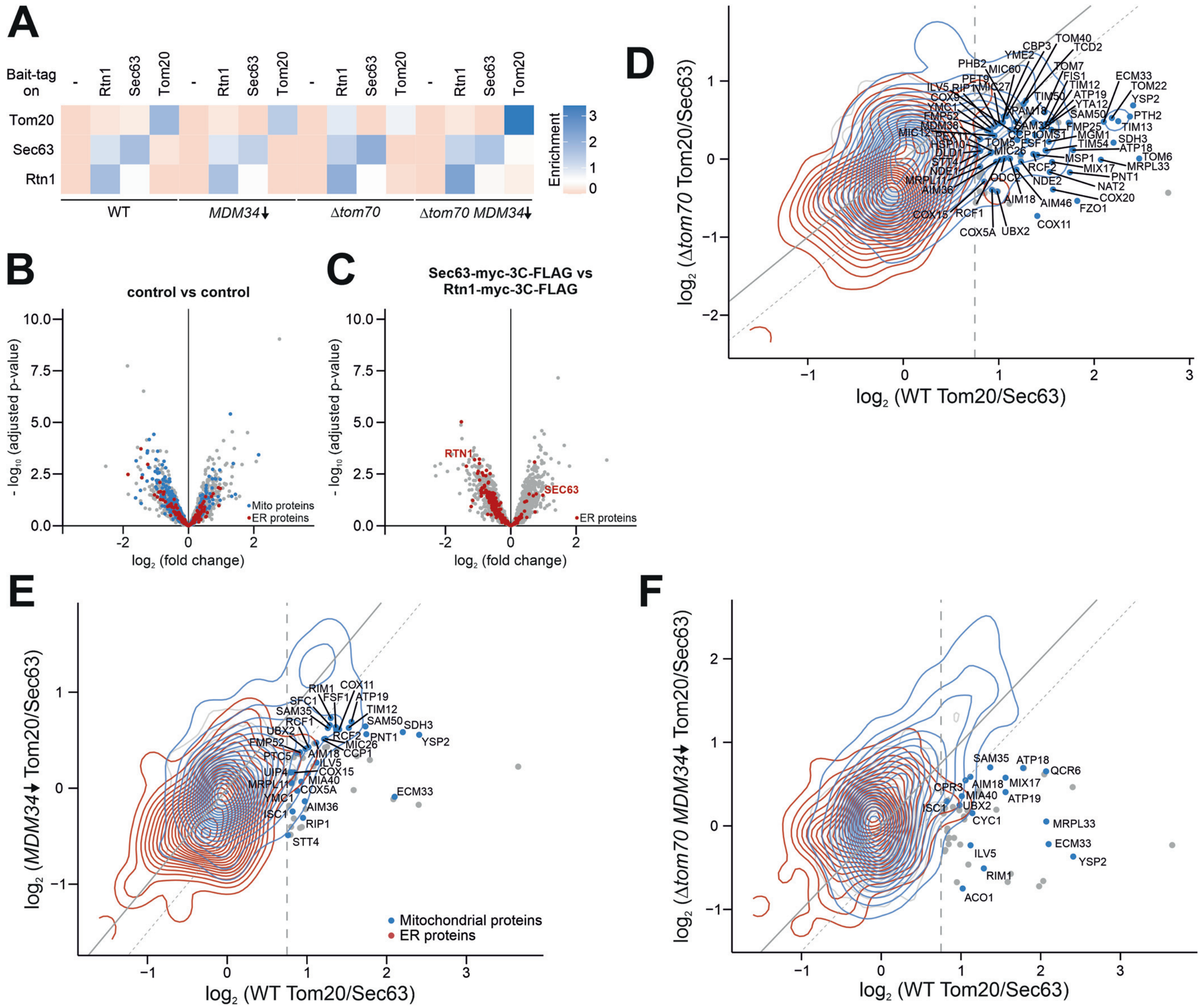

Figure EV5. Proteomic analysis of ERMES and Tom70-deficient cells identifies ER-SURF clients.

(**A**) Specific enrichment scores (difference in log2 fold change) for Tom20 and the ER proteins Sec63 and Rtn1 in the affinity-purified fractions were calculated to validate the selective recovery of the bait proteins. Data show mean values of three biological replicates. (**B**, **C**) Volcano plots of the proteomic data of the affinity-purified fractions. All measurements are based on three biological replicates and were processed as described in Materials and Methods. For the calculation of fold changes and *p* values, the limma package within the R programming language was used (Ritchie et al, 2015). (**D–F**) Correlation plot showing the log2 fold changes of the ER vs mitochondria fractions from the Sec63 and Tom20 affinity purification samples from wild type (x-axis) and Δ*tom70* (**D**), Mdm34-depleted (**E**), and Δ*tom70* Mdm34-depleted (**F**) (y-axis) cells. Samples on the diagonal were unaffected by Mdm34 depletion, whereas samples in the area indicated by the dashed line were classified as putative ER-SURF clients. The names of these mitochondrial proteins are shown.

 © The Author(s)
